# Rare Gold-Catalyzed 4-*exo*-*dig* Cyclization for Ring Expansion of Propargylic Aziridines toward Stereoselective (*Z*)-Alkylidene Azetidines, via Diborylalkyl Homopropargyl Amines

**DOI:** 10.1021/acs.orglett.4c02415

**Published:** 2024-09-02

**Authors:** Oriol Salvadó, Jorge Pérez-Ruíz, Alba Mesas, M. Mar Díaz-Requejo, Pedro J. Pérez, Elena Fernández

**Affiliations:** † Faculty of Chemistry, University Rovira i Virgili, 43007 Tarragona, Spain; ‡ Laboratorio de Catálisis Homogénea, Unidad Asociada al CSIC, Centro de Investigación en Química Sostenible (CIQSO) and Departamento de Química, Universidad de Huelva, 21007 Huelva, Spain

## Abstract

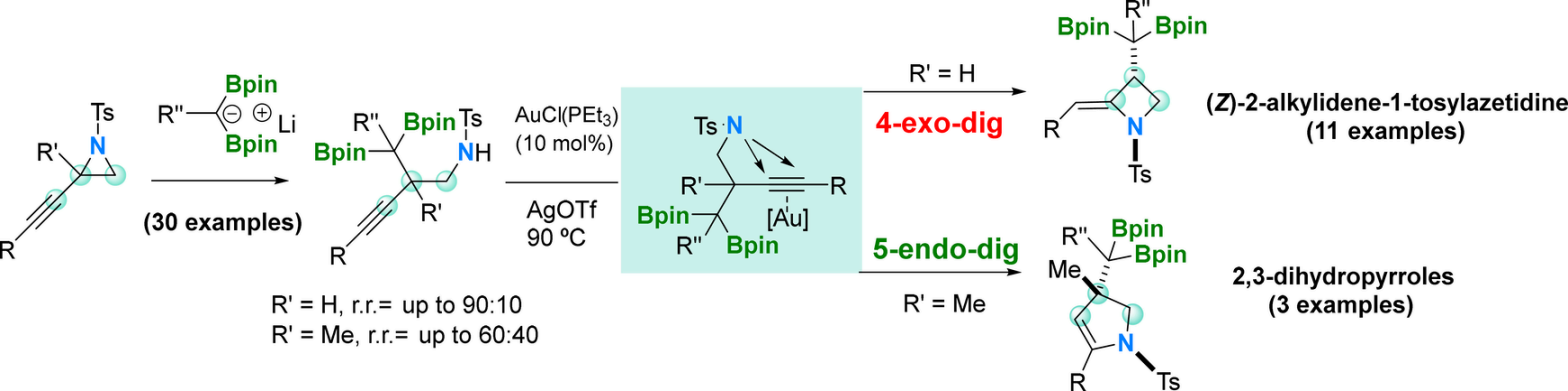

We report an uncommon 4-*exo*-*dig* cyclization of *N*-tosyl homopropargyl amines, catalyzed by [AuCl(PEt_3_)]/AgOTf, to prepare stereoselective (*Z*)-2-alkylidene-1-tosylazetidine compounds. The reaction outcome contrasts with the gold-catalyzed cyclization of *N*-tosyl homopropargyl amines containing a methyl group at the propargylic position that provides substituted 2,3-dihydropyrroles via a 5-*endo*-*dig* mechanism. The access to *N*-tosyl homopropargyl amines is possible by the regioselective nucleophilic attack of α-diboryl alkylidene lithium salts to propargylic aziridines.

Structural modifications of core scaffolds can be performed via skeletal editing strategies, considering subtle changes on the chemical space, avoiding *de novo* synthetic sequences.^1^ Changing the ring size in the core of a molecule can significantly impact its biological activity and, hence, speed up drug discovery objectives.^2^ With that in mind, reactions that break and rejoin atomic bonds by deleting, adding, or swapping atoms are considered a kind of convenient molecular surgery for molecular design.^3^ Ring expansion of *N*-tosylaziridines^4^ to azetidines is a challenging ring size manipulation and there has been very little examples that faced this problem and succeeded. Biocatalytic one-carbon ring expansion of aziridines, via [1,2]-Stevens rearrangement, is a valuable protocol that allows for the synthesis of azetidines, even with asymmetric induction (Scheme 1a).^5^ Alternative ring expansion of aziridines to azetidines employed phenacyl bromide derivatives via *in situ* generated ammonium ylides in a silica gel–water system (Scheme 1b).^6^ Visible light has also induced ring expansion of *N*-tosylaziridines with 1-bromo-1-nitroalkanes to afford 2-nitro azetidines with controlled regio- and diastereoselectivity (Scheme 1c).^7^ All of those attempts ran the fruitful ring expansion of *N*-tosylaziridines to azetidine synthesis, requiring the intermolecular interaction of aziridines with an external carbon. Here, we describe a new azetidine synthesis from propargyl aziridines, involving a rearrangement promoted by regioselective nucleophilic diborylalkylation ring opening, followed by a stereoselective Au-catalyzed ring-closing step, via a 4-*exo*-*dig* mechanism (Scheme 1d).

**Scheme 1:**
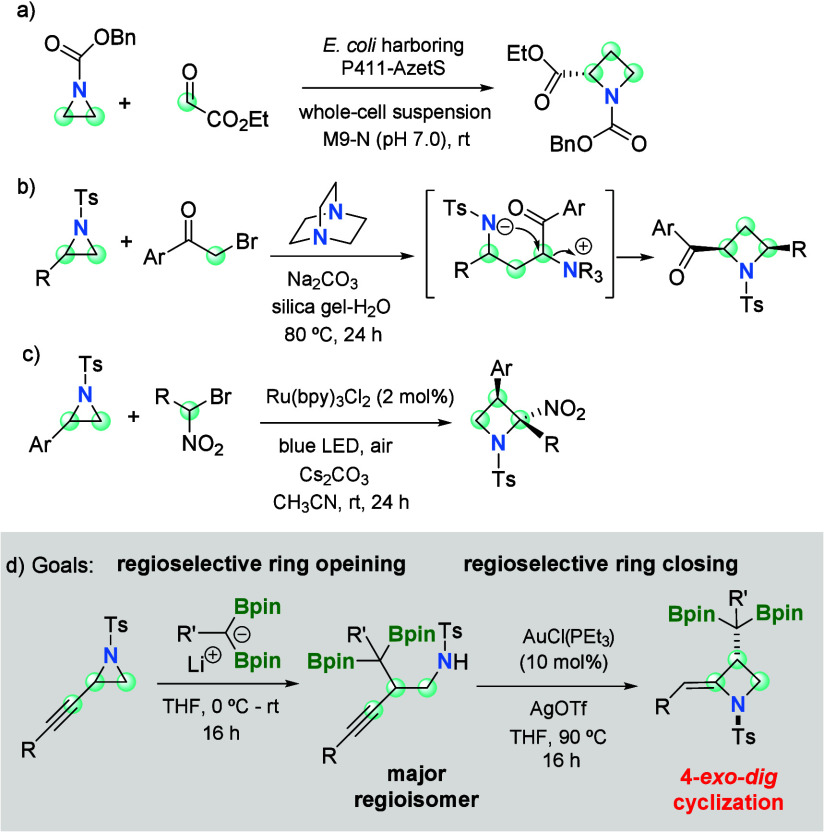
Ring Expansion Protocols To Transform Aziridines into Azetidines

The ability to precisely edit a four-membered heterocyclic ring from the corresponding propargylic aziridine not only represents an interesting ring expansion but also allows for the stereoselective formation of (*Z*)-2-alkylidene-1-tosylazetidine compounds that, to the best of our knowledge, are prepared for the first time in this work.

Our first goal is focused on the regioselective ring opening of propargylic aziridines with organoboron compounds. Whereas S_N_2 borylative ring opening of aziridines and vinyl aziridines to generate β-aminoboronate compounds are well-known processes,^8^ the borylative ring opening of propargylic aziridines is illustrated in one single example, providing the corresponding allenyl boronate product, following a preferred S_N_2′ process.^9^ Alternatively, the synthesis of γ-aminoboronic esters can be conducted via nucleophilic ring opening of aziridines^10^ and vinyl aziridines^11^ with α-borylcarbanions,^12^ although propargylic aziridines have never been explored toward this synthetic goal.^13^ To explore the regioselective nucleophilic attack of α-diborylcarbanions on propargylic aziridines, we selected bis(pinacolato)borylmethane **1a** to react with lithium diisopropylamide (LDA), in tetrahydrofuran (THF) at 0 °C, followed by the addition of 2-(phenylethynyl)-1-tosylaziridine (**2**) (Table 1).^14^

**Table 1:**
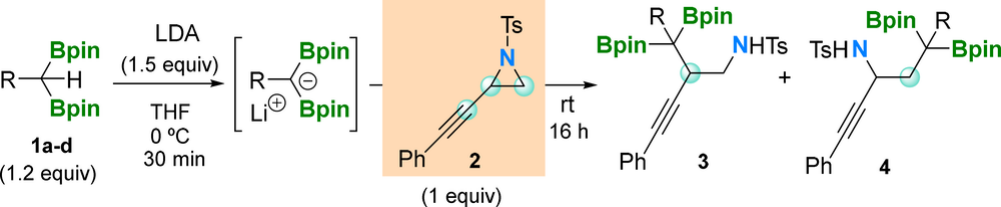

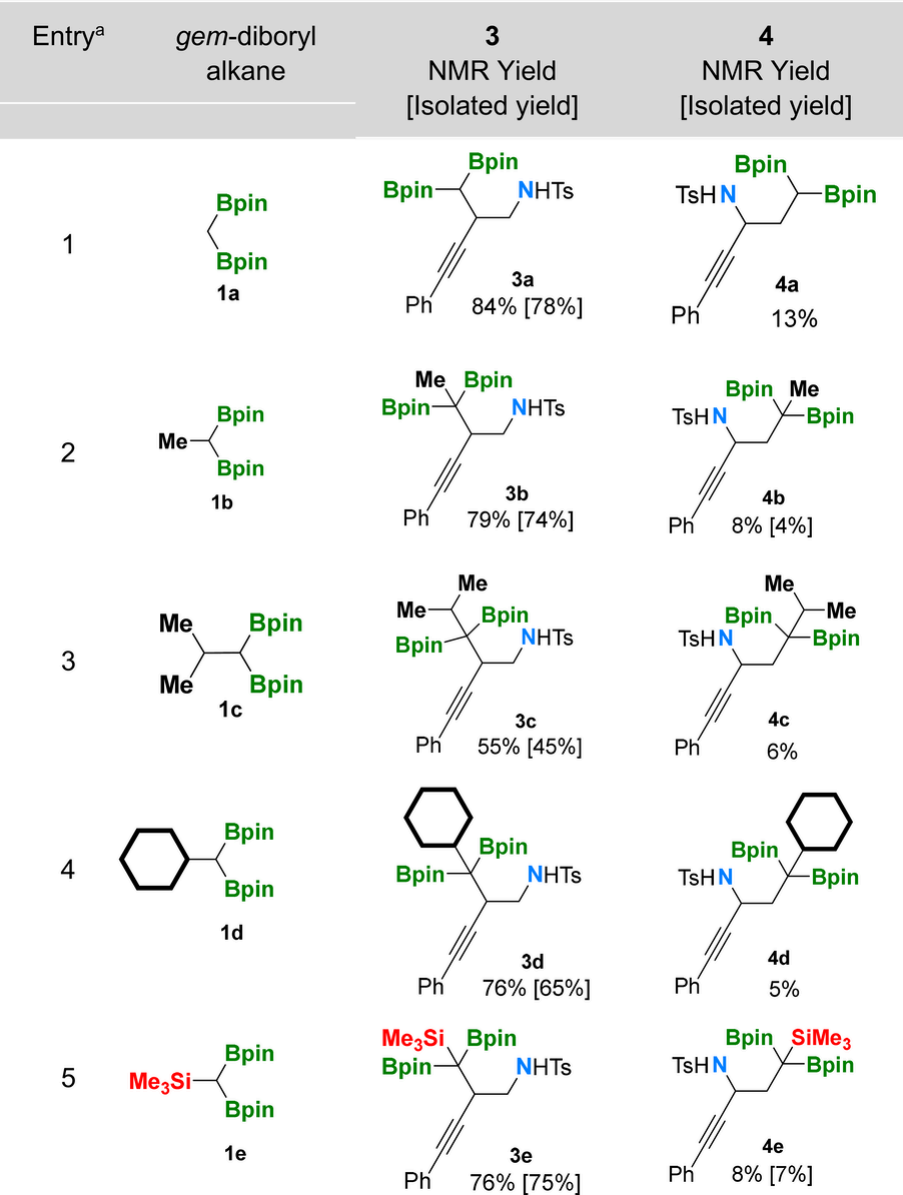
Regioselective Ring Opening of 2-(Phenylethynyl)-1-tosylaziridine (**2**) with α-Diboryl Alkylidene Lithium Salts^a^

a Reaction conditions: *gem*-diborylalkane (0.24 mmol, 1.2 equiv), LDA (0.3 mmol, 1.5 equiv), and THF at 0 °C for 30 min, followed by the addition of 2-(phenylethynyl)-1-tosylaziridine (**2**) (0.2 mmol) at room temperature for 16 h.

The transformation was quantitative after 16 h at room temperature with the formation of the major regioisomeric product **3a**, demonstrating that the diborylalkylation/ring opening took place at the most hindered position of aziridine, by virtue of the electronic properties of the adjacent triple bond (entry 1 in Table 1). The regioisomeric product **4a** was also observed by nuclear magnetic resonance (NMR) in a 13% yield. We generalized the regioselective trend for diborylalkylation/ring opening of compound **2**, even introducing steric hindrance at the diborylalkane reagent **1**, with R = Me (**1b**), R = ^*i*^Pr (**1c**), and R = Cy (**1d**). We proved the formation of products **3b**, **3c**, and **3d**, as major regioisomers, together with the formation of products **4b**, **4c**, and **4d** in <10% (entries 2–4 in Table 1). Interestingly, the most sterically hindered reagent **1e**, with R = SiMe_3_, reacted efficiently to synthesize the regioisomer **3e** in 75% isolated yield with 7% of the minor regioisomer **4e** (entry 5 in Table 1). This is in line with the stereoselective C–C bond formation when diborylsilylalkyl lithium salts react with vinyl epoxides^15^ for ring-opening reactions.

Having explored the viability of the regioselective diborylalkylation/ring opening of compound **2**, we selected reagents **1a**, **1b**, and **1e** to react with propargylic aziridines modified electronically,^14^ replacing the phenyl group by *p*-MeOC_6_H_4_ in compound **5** or *p*-ClC_6_H_4_ in compound **8** (Scheme 2).

**Scheme 2:**
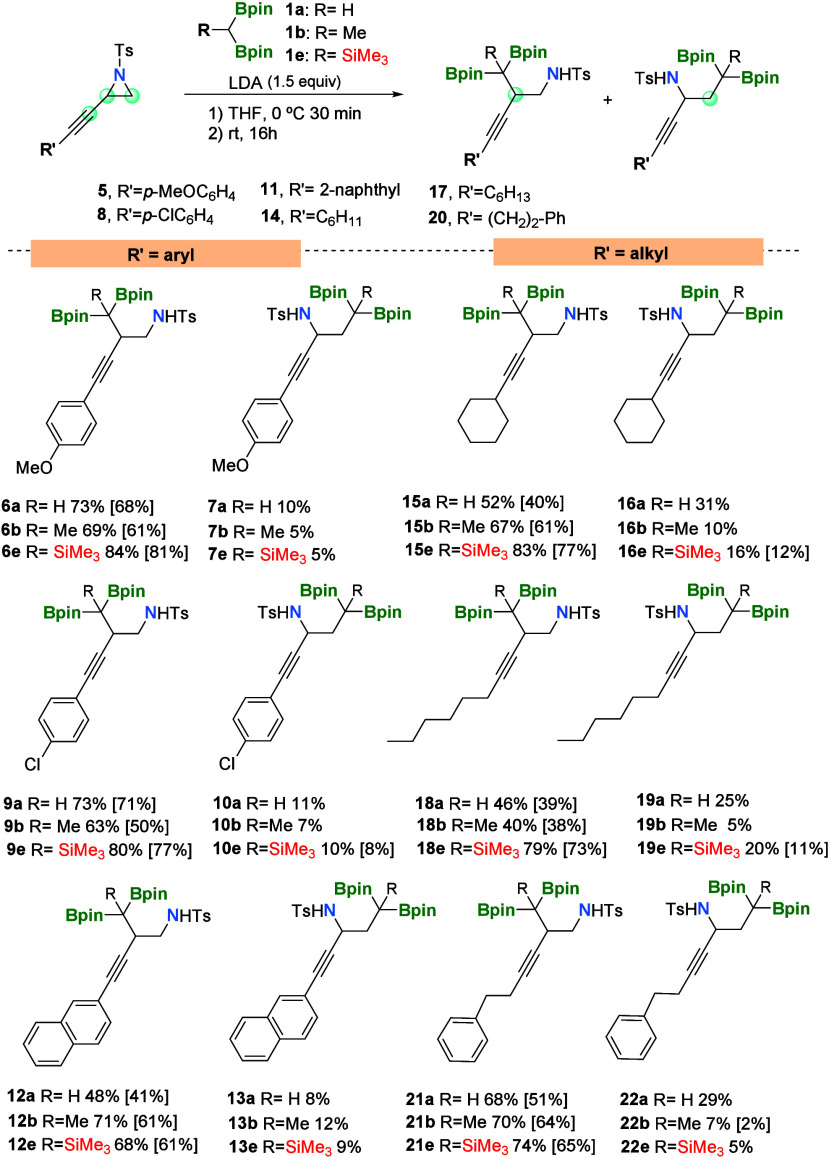
Substrate Scope on Nucleophilic Attack of α-Diborylcarbanions on Propargylic Aziridines Reaction conditions: *gem*-diborylalkane (0.24 mmol, 1.2 equiv), LDA (0.3 mmol, 1.5 equiv), and THF at 0 °C for 30 min, followed by the addition of 2-(phenylethynyl)-1-tosylaziridine (**2**) (0.2 mmol) at room temperature for 16 h.

We observed that electron-donating or electron-withdrawing properties on propargylic aziridines **5** and **8**, respectively, do not affect the reaction outcome when reacted with compounds **1a** and **1b**. The use of the more hindered diborylsilylalkyl lithium salts allowed for the isolation of the regioisomers **6e** and **9e** in 81 and 77% yields, respectively (Scheme 2). When propargylic aziridine **11**, with 2-naphthyl substituent, was employed as a substrate for diborylalkylation/ring opening with compounds **1a**, **1b**, and **1e**, we noticed that the major isomer was isolated in moderate yield (41% for compound **12a**, 61% for compound **12b**, and 61% for compound **12e**), presumably as a result of the steric hindrance on the substrate. Interestingly, when the substituent on propargylic aziridine **14** was the cyclohexyl group, products **15a**, **15b**, and **15e** were also formed as the preferred regioisomers (isolated yields of 40, 61, and 77%, respectively), despite the lack of aryl groups conjugated to the triple bond. A similar behavior was observed for the *n*-hexyl substituent of propargyl aziridine **17** that generated products **18a**, **18b**, and **18e** in 39, 38, and 73%, respectively. Eventually, propargylic aziridine **20**, with R′ = CH_2_–CH_2_–Ph, also favored the diborylalkylation/ring opening with reagents **1a**, **1b**, and **1e** on the most hindered position, producing products **21a**, **21b**, and **21e** in 51, 64, and 65% isolated yields, respectively (Scheme 2).

When propargylic aziridines contain a methyl group at the propargylic position (**23**, R′ = Ph; **26**, R′ = 2-naphthyl), the diborylalkylation/ring opening with reagent **1a** occurred without apparent regioselectivity (see products **24a** and **25a** in Scheme 3). A similar trend has been observed when the more hindered reagents **1b** or **1e** were employed in the diborylalkylation/ring opening of model substrate **23**. The diborylalkylation of the most hindered propargylic aziridine **26** with the most hindered reagent **1e** also produced both regioisomers **27e** and **28e** in similar isolated yields (Scheme 3).

**Scheme 3:**
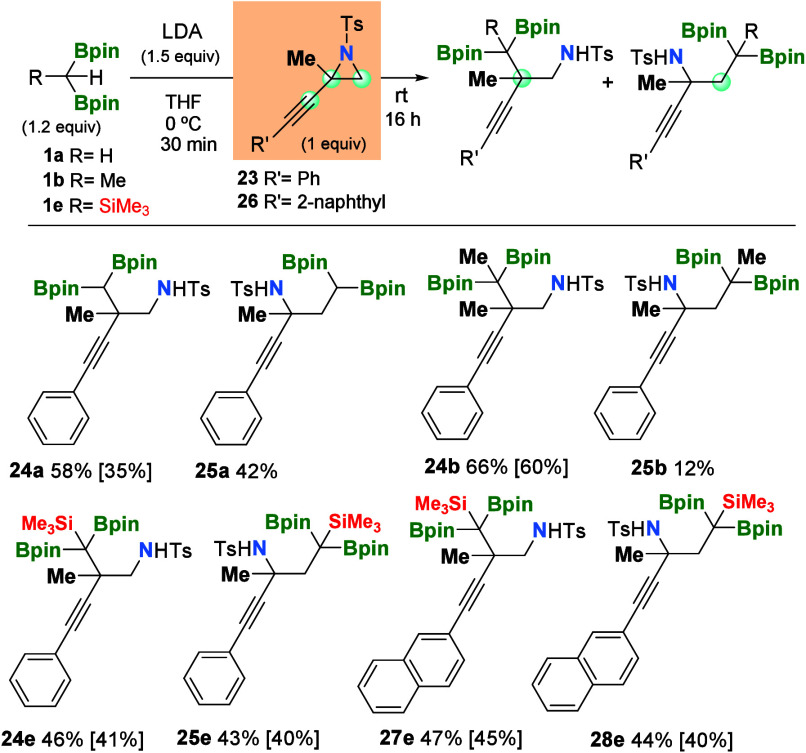
Nucleophilic Ring Opening of Me-Substituted Propargylic Aziridines with α-Diborylcarbanions Reaction conditions: *gem*-diborylalkane (0.24 mmol, 1.2 equiv), LDA (0.3 mmol, 1.5 equiv), and THF (1 mL) at 0 °C for 30 min, followed by the addition of propargyl aziridines (0.2 mmol) at room temperature for 16 h.

Remarkably, it seems that the electronic properties of propargylic carbon favor the formation of the vicinal quaternary centers in products **24a**/**24b**/**24e** and **27e**, despite the sterically hindered position. The formation of the propargylamines **25a**/**25b**/**25e** and **28e** also represents a straightforward access to valuable tetrasubstituted carbon centers with diverse polyfunctionality (Scheme 3).^16^

Considering the chemical structure of the *N*-tosyl homopropargyl amines prepared in this work, we planned to conduct the gold-catalyzed cyclization to synthesize heterocyclic compounds.^17^ We selected the complex [AuCl(PEt_3_)] (10 mol %), in the presence of AgOTf (10 mol %), as a scavenger of the Cl anion, because it is known that AgOTf does not catalyze this cyclization.^18^ We explored the gold-catalyzed cyclization on *N*-tosyl homopropargyl amine **24e**, and after 16 h at 90 °C, we isolated the corresponding substituted 2,3-dihydropyrrole **29e**, as a single diastereoisomer in 91% isolated yield (Scheme 4, top) suggesting a 5-*endo*-*dig* cyclization pathway. The configuration of the quaternary center was unequivocally assigned with the methyl group *cis* to the *N*-tosyl moiety, as confirmed by X-ray diffraction studies of compound **29e** (Scheme 4). The direct 5-*endo*-*dig* cyclization of the *N*-tosyl homopropargyl amine **24e** seems to follow the favored Baldwin’s rules for the synthesis of the heterocyclic dihydropyrrole ring.^20^

**Scheme 4:**
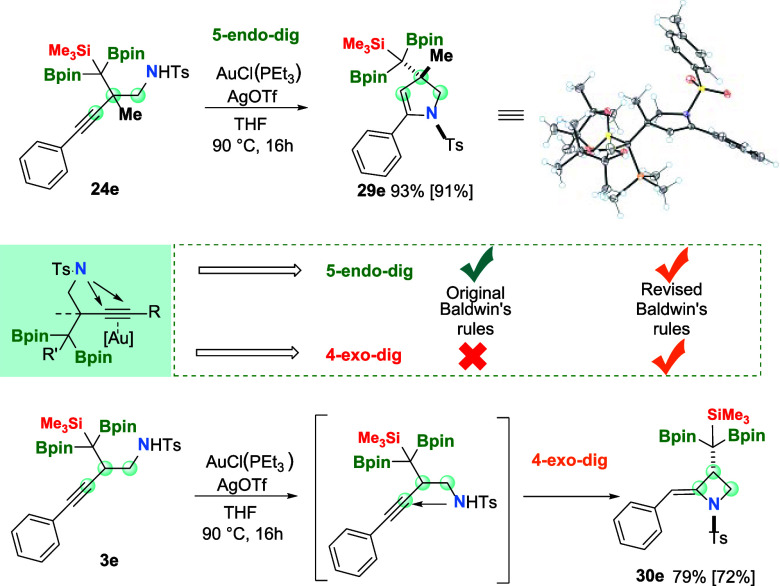
Au-Catalyzed Cyclization of *N*-Tosyl Homopropargyl Amines Reaction conditions: *N*-tosyl homopropargyl amine (0.1 mmol, 1 equiv), [AuCl(PEt_3_)] (0.01 mmol, 3.51 mg), AgOTf (0.01 mmol, 2.57 mg), and THF (1 mL) at 90 °C for 16 h.

However, when we conducted the gold-catalyzed cyclization of the analogous *N*-tosyl homopropargyl amine **3e**, the corresponding alkylidene azetidine **30e** was formed instead (Scheme 4, bottom). The formation of the four-membered ring alkylidene azetidine **30e** can be explained by the unlikely 4-*exo*-*dig* cyclization, which, on the basis of the acute angle formed by the interacting atoms, had been considered unfavorable by Baldwin rules.^21^ Because those rules have been comprehensively revisited, the mechanism for 4-*exo*-*dig* cyclization could be justified by a plausible obtuse angle of attack (Scheme 4).^22^ Scare examples of gold-catalyzed 4-*exo*-*dig* cyclization have been described,^23^ together with other catalytic or radical initiators for 4-*exo*-*dig* carbocyclization of alkynes.^24^ We extended the 5-*endo*-*dig* cyclization pathway for *N*-tosyl homopropargyl amines containing a methyl group at the propargylic position. In consequence, the cyclization of compounds **24b** and **27e**, provided, in both cases, 2,3-dihydropyrroles **29b** and **31e**, in moderate to high isolated yields (Scheme 5). In our attempt to demonstrate the feasibility of gold-catalyzed 4-*exo*-*dig* cyclization, we explored the gold-catalyzed cyclization of *N*-tosyl homopropargylamines **3b**, **9b**, and **12b**, generating the corresponding four-membered alkylidene azetidines **30b**, **32b**, and **33b** (Scheme 5). Electron-withdrawing substituents on the aryl group seem to have a beneficial influence on the cyclization, because product **32e** could be isolated in 93% yield, proceeding from *N*-tosyl homopropargyl amine **9e**, despite the steric hindrance associated with the SiMe_3_ group (Scheme 5). For these *N*-tosyl homopropargyl amines containing alkyl groups, instead of aryl groups, the formation of the corresponding alkylidene azetidines **34e** and **35e** was also feasible, noting that the unreacted substrate *N*-tosyl homopropargyl amine was isolated as the corresponding ketone as a consequence of the aqueous workup.

**Scheme 5:**
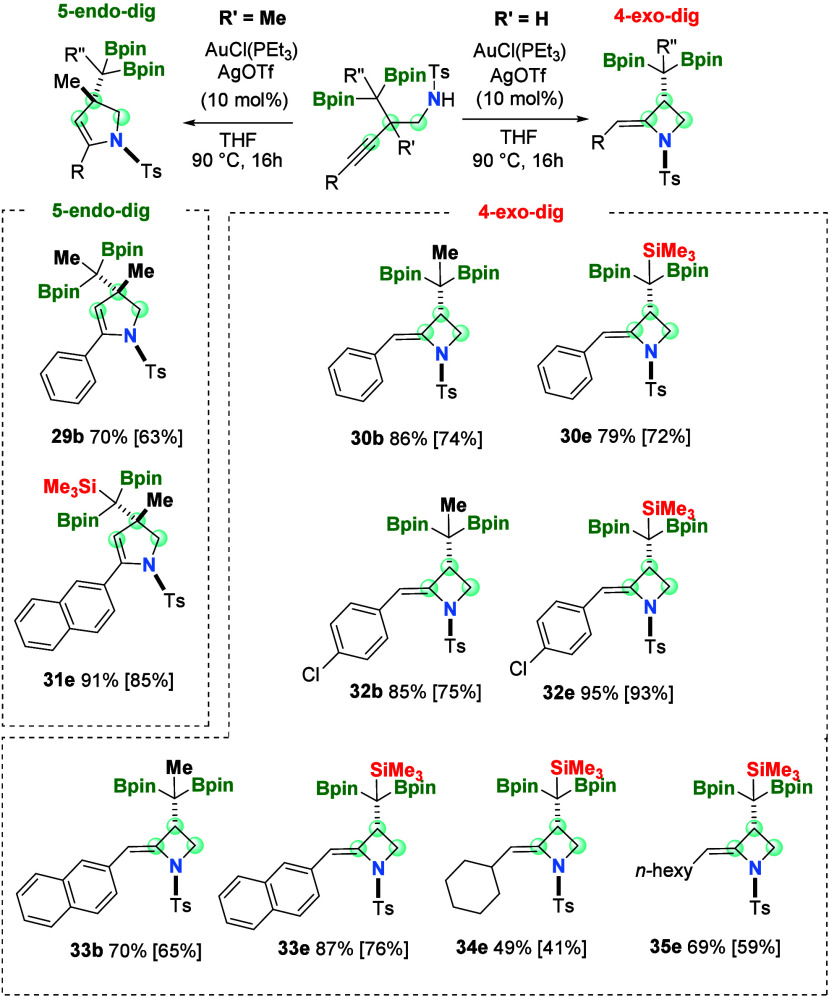
Substrate Scope for Au-Catalyzed 4-*exo*-*dig* Cyclization and 5-*endo*-*dig* Cyclization of *N*-Tosyl Homopropargyl Amines Reaction conditions: *N*-tosyl homopropargyl amine (0.1 mmol, 1 equiv), [AuCl(PEt_3_)] (0.01 mmol, 3.51 mg), AgOTf (0.01 mmol, 2.57 mg), and THF (1 mL) at 90 °C for 16 h.

Eventually, we explored the functionalization of the pending *gem*-diborylalkyl group, and toward this end, we prepared the substrate 1-tosyl-2-((4-(trifluoromethyl)phenyl)ethynyl)aziridine that was regioselectively converted into products **36a**, **36b**, and **36e** as favored regioisomers (Scheme 6). When we performed the gold-catalyzed cyclization of products **36a** and **36b**, the alkylidene azetidines **38a** and **38b** were exclusively formed and isolated in a moderate yield (Scheme 6). The functionalization was explored via base-mediated protodeborylation, generating product **39** in a high yield, as a mixture of 1:1 diastereoisomers (Scheme 6). The oxidation of the alkylidene azetidine **39** with NaBO_3_ allowed for the isolation of product **40** with a pending secondary alcohol, in a 1:1 mixture of diastereoisomers (Scheme 6). This is a straightforward access to alkylidene azetidines with a 1-hydroxyethan-1-ide pendant moiety that confers potential antibacterial activity to the heterocyclic four-membered ring, in combination with the structural alkylidene function on the C_4_ position with a (*Z*) stereochemistry that proved to have more beneficial biological activity than the (*E*) stereosiomer.^25^

**Scheme 6:**
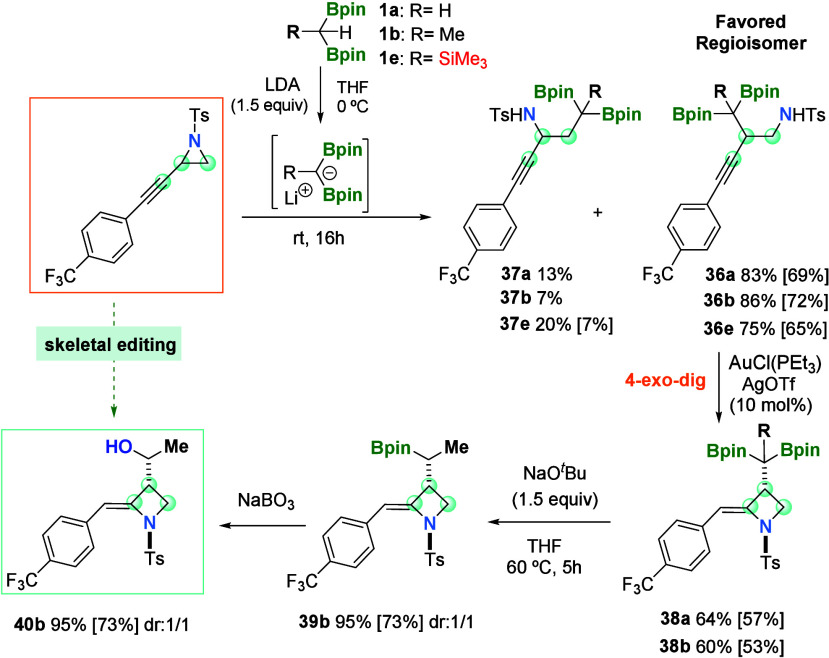
Strategic Synthesis of Alkylidene Azetidines with a 1-Hydroxyethan-1-ide Pendant Moiety Reaction conditions: *gem*-diborylalkane (0.24 mmol, 1.2 equiv), LDA (0.3 mmol, 1.5 equiv), and THF (1 mL) at 0 °C for 30 min, followed by the addition of propargyl aziridines (0.2 mmol) at room temperature for 16 h. For cyclization: *N*-tosyl homopropargyl amine (0.1 mmol, 1 equiv), [AuCl(PEt_3_)] (0.01 mmol, 3.51 mg), AgOTf (0.01 mmol, 2.57 mg), and THF (1 mL) at 90 °C for 16 h. For protodeborylation: NaO^*t*^Bu (1.5 equiv) at 60 °C for 5 h. For oxidation: NaBO_3_·H_2_O (0.3 mmol, 3 equiv) for 16 h.

We conclude that the ability to precisely edit a four-membered heterocyclic ring from the corresponding propargylic aziridine not only represents an interesting ring expansion but also allows for the stereoselective formation of (*Z*)-2-alkylidene-1-tosylazetidine compounds, prepared for the first time in this work, that can be functionalized toward alkylidene azetidines with 1-hydroxyethan-1-ide pendant moieties that are synthetic cores with potential antibacterial activity.

## Data Availability

The data underlying this study are available in the published article and its Supporting Information.

## Supporting Information Available

Experimental procedures, product characterization, NMR spectra, and X-ray single-crystal diffraction analysis for product **29e** (PDF)
