# Supplementary material for: Rare Gold-Catalyzed 4-exo-dig Cyclization for Ring Expansion of Propargylic Aziridines toward Stereoselective (Z)-Alkylidene Azetidines, via Diborylalkyl Homopropargyl Amines
Source: Org Lett. 2024 Sep 2;26(36):7535–40. doi: 10.1021/acs.orglett.4c02415 (PMC11406573; doi:10.1021/acs.orglett.4c02415)
Supplement: Supplementary file 1 — ol4c02415_si_001.pdf [file ol4c02415_si_001.pdf]

A rare gold-catalyzed 4-*exo-dig* cyclization for ring expansion of propargylic aziridines towards stereoselective (*Z*)-alkylidene azetidines, via diborylalkyl homopropargyl amines

Oriol Salvadó,[a] Jorge Pérez-Ruíz,[b] Alba Mesas,[a] M. Mar Díaz-Requejo,[b*] Pedro J. Pérez,[b*] Elena Fernández[a*]

[a]Faculty of Chemistry, University Rovira i Virgili, 43007 Tarragona, Spain.
[b]Laboratorio de Catálisis Homogénea, Unidad Asociada al CSIC, CIQSO-Centro de Investigación en Química Sostenible and Departamento de Química, Universidad de Huelva, 21007 Huelva, Spain.
mariaelena.fernandez@urv.cat, mmdiaz@dqcm.uhu.es, perez@dqcm.uhu.es

Contents

S1. General information

S2. Synthesis of 1,3-Enynes

S3. Synthesis of propargylic aziridines: general method for transfer reaction of PhI=NTs to 4-substituted enynes catalyzed by Tp^{Br3}Cu(NCMe)

S4. General procedure for ring-opening of propargylic aziridines with α-diborylalkylidene lithium salts

S5. General procedure for the cyclization of *N*-tosyl homopropargyl amines

S6. General procedure for protodeborylation of (*Z*)-2-alkylidene-1-tosylazetidines

S7. General procedure for oxidation of (*Z*)-2-alkylidene-1-tosylazetidines

S8. Characterization of new propargylic aziridines

S9. Characterization of *N*-tosyl homopropargyl amines and *N*-tosyl propargyl amines

S10. Characterization of substituted 2,3-dihydropyrroles

S11. Characterization of (*Z*)-2-alkylidene-1-tosylazetidines

S12. Characterization of 39b and 40b

S13. NMR spectra of propargylic aziridines

S14. NMR spectra of *N*-tosyl homopropargyl amines and *N*-tosyl propargyl amines

S15. NMR spectra of substituted 2,3-dihydropyrroles

S16. NMR spectra of (*Z*)-2-alkylidene-1-tosylazetidines

S17. NMR spectra of 39b and 40b

S18. X-ray single-crystal diffraction analysis for product 29e

S19. References

S1. General information

Solvents and reagents: Solvents and reagents were obtained from commercial suppliers and dried and/or purified (if needed) by standard procedures. Diboron reagents were purchased from Ally Chem and used without further purification. Commercially unavailable 1,3-enynes were obtained following the detailed procedures in this section. The metallic complex [Tp^{Br3}Cu(NCMe][S1] and the nitrene precursor (PhI=NTs)[S2] were synthetized by literature procedures. All reactions were conducted in an oven and flame-dried glassware under an inert atmosphere of argon, using Schlenk-type techniques. *Flash chromatography* was performed on standard silica gel (Merck Kieselgel 60 F254 400-630 mesh). *Thin layer chromatography* was performed on Merck Kieselgel 60 F254 which was developed using standard visualizing agents: UV fluorescence (254 and 366 nm) or potassium permanganate/Δ. *NMR spectra* were recorded at a Varian Goku 400 spectrometer, a Varian Mercury 400 spectrometer, Agilent 400 NMR spectrometer and Agilent 500DD2 spectrometer as solutions at 298 K and Bruker Advance III HD 500 MHz spectrometer at ambient temperature. ^{1}H NMR and $^{13}C\{^{1}H\}$ NMR chemical shifts (δ) are reported in ppm with the solvent resonance as the internal standard ($CHCl_3$: 7.26 ppm (^{1}H)) and ($CDCl_3$: 77.16 ppm (^{13}C). $^{11}B\{^{1}H\}$ NMR chemical shifts (δ) are reported in ppm relative to $(CH_3)_2O\cdots BF_3$. Data are reported as follows: chemical shift, multiplicity (s = singlet, d = doublet, t = triplet, q = quartet, hept = septuplet, br = broad, m = multiplet), coupling constants (Hz), and integration. *High-resolution mass spectra (HRMS)* were recorded using a 6210 Time of Flight (TOF) mass spectrometer from Agilent Technologies (Waldbronn, Germany) with an ESI interface and it was performed at the Servei de Recursos Científics i Tècnics (Universitat Rovira i Virgili, Tarragona) or using a BIOTOF II Time of Flight (TOF) mass spectrometer from Bruker with an APCI interface or EI interface and it was performed at the Unidade de Espectrometría de Masas e Proteómica (Universidade de Santiago de Compostela, Santiago de Compostela) and Centre for Research in Sustainable Chemistry (CIQSO) of the University of Huelva. GC-MS analyses were performed on an HP6890 gas chromatograph, and an Agilent Technologies 5973 Mass selective detector (Waldbronn, Germany) equipped with an achiral capillary column HP-5 (30m, 0.25mm i. d., 0.25μm thickness) using He as the carrier gas.

S2. Synthesis of 1,3-Enynes [S3]

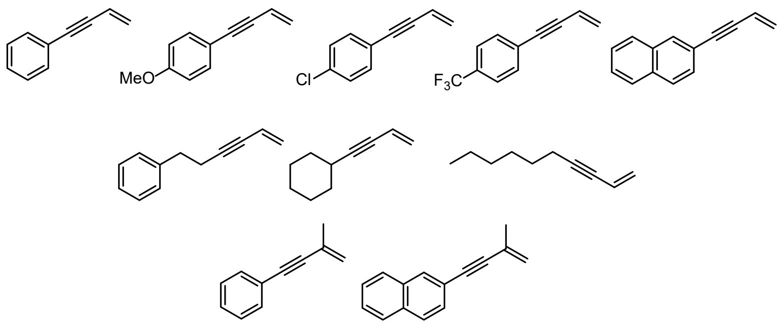

In a Schlenck tube, under inert atmosphere, $Pd(PPh_3)_4$ (0.077 mmol, 0.02 equiv.) was placed, followed by β-bromostyrene (3.84 mmol, 1 equiv., 1M solution in THF) diluted in piperidine (3.8 mL). The mixture was stirred for 5 min. and then the alkyne (3.84 mmol, 1 equiv.) and CuI (0.269 mmol, 0.07 equiv.) were added before stirring overnight. Then, the mixture was diluted with Et_2O (15 mL). The combined organic phases were washed with H_2O (20 mL), brine (20 mL), separated, dried with $MgSO_4$ and evaporated under vacuum. Purification was carried out by flash chromatography on silica gel (eluent: n-hexane).

S3. Synthesis of propargylic aziridines: general method for transfer reaction of PhI=NTs to 4-substituted enynes catalyzed by Tp^{Br3}Cu(NCMe).

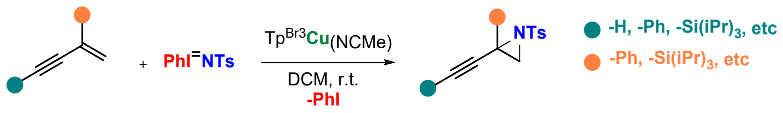

In a Schlenk tube, under Nitrogen atmosphere, the catalyst (0.02 mmol) was dissolved in dry/deoxygenated DCM (15 mL) and 1,3-enyne was added (1.2 mmol). After few minutes, PhI=NTs (0.4 mmol) was added to the reaction media in one portion, and the mixture was stirred at room temperature until no solid was observed in suspension (3-4 h). The solvent was removed under reduced pressure and the reaction crude was analyzed by NMR spectroscopy. The residue was purified through a silica gel column (eluent n-hexane/EtOAc).

S4. General procedure for ring-opening of propargylic aziridines with α-diborylalkylidene lithium salts

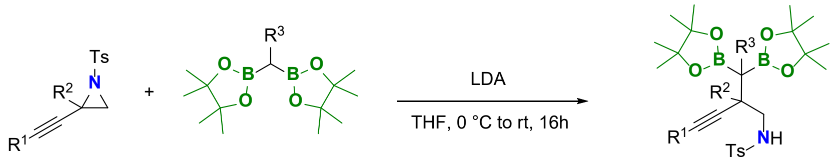

In an oven-dried Schlenk-type flask, equipped with a teflon screw cap and a magnetic stirring bar, 0.25 mmol of *gem*-diborylalkane was added (1.25 equiv). After flushing the flask with Ar for three times, 1.5 mL of dry THF was added at 0 °C. Then 0.3 mmol (1.5 equiv, 0.3 mL) of LDA (1M) in hexane was added at 0 °C. The reaction mixture was stirred for 30 minutes at 0 °C and after this period of time, 0.2 mmol (1 equiv) of the corresponding propargylic

aziridine and 0.5 mL of dry THF were added to the mixture to stir at 0 °C for 10 minutes. Finally, the reaction mixture was stirred at rt for 16 h. After this reaction time, the solvents were gently evaporated and the crude was purified using silica gel chromatography to afford the corresponding isolated product.

Specific example: (1 mmol)

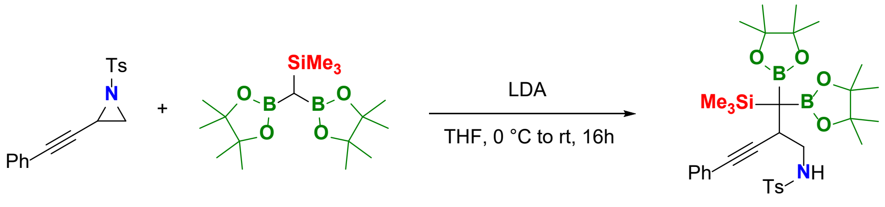

2 **1e** **3e**

In an oven-dried Schlenk-type flask, equipped with a teflon screw cap and a magnetic stirring bar, the *gem*-diborylalkane **1e** (1.25 mmol, 425 mg, 1.25 equiv) was added. After flushing the flask with Ar for three times, 7.5 mL of dry THF was added at 0 °C. Then 1.5 mmol (1.5 equiv, 1.5 mL) of LDA (1M) in hexane was added at 0 °C. The reaction mixture was stirred for 30 minutes at 0 °C and after this period of time, the propargylic aziridine **2** (1 mmol, 236 mg, 1 equiv) and 2.5 mL of dry THF were added to the mixture to stir at 0 °C for 10 minutes. Finally, the reaction mixture was stirred at rt for 16 h. After this reaction time, the solvents were gently evaporated and the crude was purified through flash chromatography using as eluent a mixture of pentane/diethyl ether (100:30). The product **3e** was isolated as a pale yellowish solid (478 mg, 75%).

S5. General procedure for the cyclization of *N*-tosyl homopropargyl amines

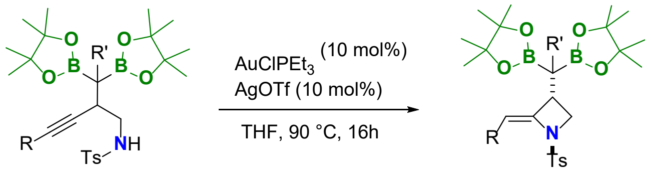

In an oven-dried Schlenk-type flask, equipped with a teflon screw cap and a magnetic stirring bar, 0.01 mmol of $AuClPEt_3$ (10 mol%, 3.51 mg) and 0.01 mmol of AgOTf (10 mol%, 2.57) were added in THF (1 mL). Then 0.1 mmol (1 equiv) of the corresponding *N*-tosyl homopropargyl amine was added at rt and the reaction mixture was stirred at 90 °C (using an oil bath) for 16 h. After the reaction time, the solvents were gently evaporated in a rotatory evaporator with a water bath at room temperature and the crude was purified using silica gel chromatography to afford the corresponding isolated product.

Specific example: (1 mmol)

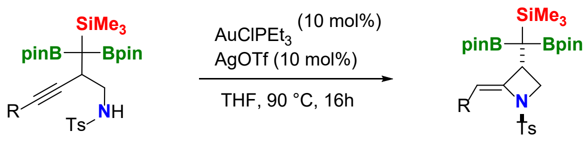

3e **30e**

In an oven-dried Schlenk-type flask, equipped with a teflon screw cap and a magnetic stirring bar, 0.10 mmol of $AuClPEt_3$ (10 mol%, 35.1 mg) and 0.1 mmol of AgOTf (10 mol%, 25.7) were added in THF (10 mL). Then 1 mmol (1 equiv, 637 mg) of the *N*-tosyl homopropargyl amine **3e** was added at rt and the reaction mixture was stirred at 90 °C (using an oil bath) for 16 h. After the reaction time, the solvents were gently evaporated in a rotatory evaporator with a water bath at room temperature and the crude was purified using silica gel chromatography and a mixture of pentane/diethyl ether (100:30) as eluent. The product **30e** was isolated as a pale yellowish solid (460 mg, 72%).

S6. General procedure for protodeborylation of (*Z*)-2-alkylidene-1-tosylazetidines

In an opened-air flask, charged with a magnetic stir bar, were added the corresponding (*Z*)-2-alkylidene-1-tosylazetidine (0.1 mmol, 1 equiv), NaO^tBu (0.15 mmol, 1.5 equiv) and THF (1 mL). The reaction was closed with a Teflon cap and the reaction was stirred at 60 ºC (using an oil bath) for 5h. After this period of time, the solvents were evaporated at the rotatory evaporator and the crude residue was purified by silica gel chromatography to obtain the corresponding protodeborylated product.

S7. General procedure for oxidation of (*Z*)-2-alkylidene-1-tosylazetidines

In an opened-air flask, charged with a magnetic stir bar, were added the corresponding (*Z*)-2-alkylidene-1-tosylazetidine (0.1 mmol, 1 equiv), $NaBO_3{\cdot}H_2O$ (0.5 mmol, 5 equiv), THF (2 mL) and distilled water (1 mL). The reaction was closed with a septum with a needle to avoid over pressures and was stirred for 16 h at room temperature. After this period of time, the mixture was extracted with Et_2O (3 x 15 mL), the organic layer was dried with anhydrous magnesium sulphate, filtered and the solvents were evaporated. The crude residue was purified by silica gel chromatography to obtain the corresponding product.

S8. Characterization of new propargylic aziridines.

2-Methyl-2-(naphthalen-2-ylethynyl)-1-tosylaziridine

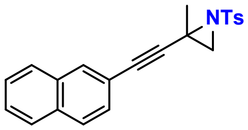

The product was purified by flash chromatography using as eluent a mixture of hexane/ethyl acetate (8:2). The product was isolated as a pale-yellow solid (116 mg, 84%).

^{1}H NMR (500 MHz, CDCl$_3$): δ = 7.99 (d, *J* = 1.48 Hz, 1H), 7.91 (d, *J* = 8.13 Hz, 2H), 7.79 (m, 3H), 7.49 (m, 3H), 7.31 (d, *J* = 8.02 Hz, 2H), 3.05 (s, 1H), 2.59 (s, 1H), 2.43 (s, 3H). 1.76 (s, 3H).

^{13}C NMR {^{1}H} (125 MHz, CDCl$_3$): δ =144.4, 136.7, 133.1, 132.9, 132.1, 129.6, 129.6, 128.4, 128.0, 128.0, 127.9, 127.8, 126.9, 126.6, 119.5, 86.4, 54.8, 42.0, 38.9, 23.8, 21.7.

HRMS (ESI) for **$C_{22}H_{19}NO_2SNa$[M+Na$^+$]:** calculated 384.1028, found = 384.1021.

2-((4-chlorophenyl)ethynyl)-1-tosylaziridine

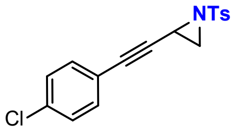

The product was purified by flash chromatography using as eluent a mixture of hexane/ethyl acetate (8:2). The product was isolated as a white solid (112 mg, 85%).

^{1}H NMR (500 MHz, CDCl$_3$): δ = 7.84 (d, *J* = 8.36 Hz, 2H), 7.32 (d, *J* = 8.07 Hz, 2H), 7.28 (d, *J* = 8.61 Hz, 2H), 7.21 (d, *J* = 8.51 Hz, 2H), 3.45 (dd, *J* = 7.07, 4.33 Hz, 1H), 2.81 (d, *J* = 7.04 Hz, 1H), 2.48 (d, *J* = 4.42 Hz, 1H), 2.40 (s, 3H).

^{13}C NMR {^{1}H} (125 MHz, CDCl$_3$): δ = 145.3, 135.1, 134.5, 133.4, 130.1, 128.9, 128.8, 128.2, 120.4, 85.0, 81.7, 35.0, 28.7, 21.8.

HRMS (ESI) for **$C_{17}H_{14}NO_2SNaCl$[M+Na$^+$]:** calculated 354.0300, found = 354.0329.

1-tosyl-2-((4-(trifluoromethyl)phenyl)ethynyl)aziridine

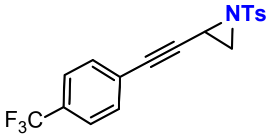

The product was purified by flash chromatography using as eluent a mixture hexane/ethyl acetate (8:2). The product was isolated as a pale-yellow solid (111 mg, 76%).

^{1}H NMR (500 MHz, CDCl$_3$): δ = 7.87 (d, *J* = 8.28 Hz, 2H), 7.55 (d, *J* = 8.26 Hz, 2H), 7.50 (d, *J* = 8.18 Hz, 2H), 7.37 (d, *J* = 8.03 Hz, 2H), 3.49 (dd, *J* = 7.04, 4.32 Hz, 1H), 2.85 (d, *J* = 7.07 Hz, 1H), 2.52 (d, *J* = 4.33 Hz, 1H), 2.46 (s, 3H).

^{13}C NMR {^{1}H} (125 MHz, CDCl$_3$): δ = 145.3, 134.4, 132.3, 130.7 (q, *J* = 32.73 Hz), 130.0, 128.2, 125.6, 125.3 (q, *J* = 3.79 Hz), 123.8 (q, *J* = 271.76 Hz), 86.3, 81.2, 34.9, 28.4, 21.8.

^{19}F NMR {^{1}H} (470 MHz, CDCl$_3$): -62.95.

HRMS (ESI) for **$C_{18}H_{14}F_3NO_2SNa$[M+Na$^+$]:** calculated 388.0589, found = 388.0562.

S9. Characterization of *N*-tosyl homopropargyl amines and *N*-tosyl propargyl amines

N-(2-(bis(4,4,5,5-tetramethyl-1,3,2-dioxaborolan-2-yl)methyl)-4-phenylbut-3-yn-1-yl)-4-methylbenzenesulfonamide (3a)

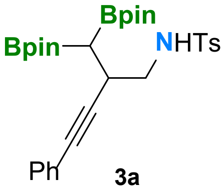

3a

The product was purified by flash chromatography using as eluent a mixture of pentane/diethyl ether (100:30). The product was isolated as a pale yellowish solid (88 mg, 78%).

^{1}H NMR (400 MHz, $CDCl_3$): δ = 7.76 (d, *J* = 8.3 Hz, 2H), 7.36 – 7.19 (m, 7H), 4.97 (m,1H), 3.31 – 3.22 (m, 1H), 3.13 – 2.96 (m, 2H), 2.39 (s, 3H), 1.22 (s, 6H), 1.20 (s, 6H), 1.19 (s, 6H), 1.14 (s, 6H), 1.11 (d, *J* = 9.8 Hz, 1H).

^{13}C NMR {^{1}H} (125 MHz, $CDCl_3$): δ = 143.2, 137.1, 131.7, 129.7, 128.2, 127.9, 127.3, 123.3, 91.4, 83.7, 83.6, 82.5, 48.1, 29.6, 24.9, 24.9, 24.5, 24.5, 21.6

^{11}B NMR (128.3 MHz, $CDCl_3$): δ 32.7.

HRMS (ESI) for **$C_{30}H_{42}B_2NO_6S^+$[M+H]$^+$:** calculated: 566.2918, found: 566.2924.

N-(2-(1,1-bis(4,4,5,5-tetramethyl-1,3,2-dioxaborolan-2-yl)ethyl)-4-phenylbut-3-yn-1-yl)-4-methylbenzenesulfonamide (3b)

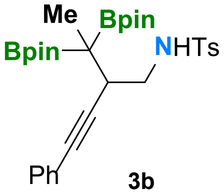

3b

The product was purified by flash chromatography using as eluent a mixture of pentane/diethyl ether (100:30). The product was isolated as a pale brownish solid (86 mg, 74%).

^{1}H NMR (400 MHz, $CDCl_3$): δ = 7.80 – 7.73 (m, 2H), 7.34 – 7.28 (m, 2H), 7.27 – 7.23 (m, 5H), 5.03 (dd, J = 8.2, 3.3 Hz, 1H), 3.26 (dd, J = 10.1, 8.2 Hz, 1H), 3.16 – 3.03 (m, 2H), 2.39 (s, 3H), 1.22 (s, 6H), 1.21 (s, 6H), 1.18 (s, 12H), 1.15 (s, 3H).

^{13}C NMR {^{1}H} (125 MHz, $CDCl_3$): δ = 143.3, 137.2, 131.8, 129.7, 128.3, 127.9, 127.4, 123.5, 90.3, 83.8, 83.7, 83.3, 45.2, 35.6, 24.9, 24.9, 24.8, 24.6, 21.6, 12.9.

^{11}B NMR (128.3 MHz, $CDCl_3$): δ 32.6.

HRMS (ESI) for **$C_{31}H_{44}B_2NO_6S$[M+H$^+$]$^+$:** calculated: 580.3076, found: 580.3081

4-methyl-N-(4-methyl-2-(phenylethynyl)-3,3-bis(4,4,5,5-tetramethyl-1,3,2-dioxaborolan-2-yl)pentyl)benzenesulfonamide (3c)

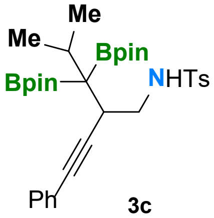

3c

The product was purified by flash chromatography using as eluent a mixture of pentane/diethyl ether (100:30). The product was isolated as a pale yellowish solid (55 mg, 45%).

^{1}H NMR (400 MHz, $CDCl_3$): δ = 7.79 (d, *J* = 8.3 Hz, 2H), 7.33 – 7.18 (m, 7H), 5.28 – 5.21 (m, 1H), 3.55 – 3.45 (m, 1H), 3.30 – 3.20 (m, 1H), 2.93 – 2.88 (m, 1H), 2.37 (s, 3H), 2.22 – 2.12 (m, 1H), 1.20 (s, 6H), 1.19 (s, 6H), 1.17 (s, 6H), 1.16 (s, 6H), 1.00 (d, *J* = 6.9 Hz, 3H), 0.90 (d, *J* = 6.8 Hz, 3H).

^{13}C NMR {^{1}H} (125 MHz, CDCl$_3$): δ = 143.0, 137.8, 131.6, 129.5, 128.2, 127.8, 127.4, 123.8, 91.1, 83.5, 83.3, 83.2, 46.1, 33.9, 29.9, 25.0, 24.9, 24.9, 24.7, 21.7, 21.5, 21.0.

^{11}B NMR (128.3 MHz, CDCl$_3$): δ 32.6.

HRMS (ESI) for **$C_{33}H_{48}B_2NO_6S^+$[M+H$^+$]$^+$:** calculated: 608.3380, found: 608.3394.

_N_-(2-(cyclohexylbis(4,4,5,5-tetramethyl-1,3,2-dioxaborolan-2-yl)methyl)-4-phenylbut-3-yn-1-yl)-4-methylbenzenesulfonamide (3d)

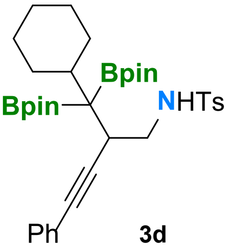

3d

The product was purified by flash chromatography using as eluent a mixture of pentane/diethyl ether (100:30). The product was isolated as a pale yellowish solid (84.4 mg, 65%).

^{1}H NMR (400 MHz, CDCl$_3$): δ 7.80 (d, J = 8.3 Hz, 2H), 7.32 – 7.26 (m, 5H), 7.24 (d, J = 8.1 Hz, 2H), 5.35 – 5.30 (m, 1H), 3.54 – 3.40 (m, 1H), 3.24 (ddd, J = 12.0, 9.2, 2.6 Hz, 1H), 2.98 (dd, J = 9.3, 4.6 Hz, 1H), 2.38 (s, 3H), 1.85 – 1.53 (m, 10H), 1.27 – 1.25 (m, 1H), 1.21 (s, 6H), 1.20 (s, 6H), 1.17 (s, 6H), 1.17 (s, 6H).

^{13}C NMR {^{1}H} (125 MHz, CDCl$_3$): δ = 143.0, 137.7, 131.6, 129.6, 128.2, 127.7, 127.4, 123.9, 91.6, 83.5, 83.4, 83.2, 45.9, 40.5, 33.1, 31.6, 31.4, 27.5, 27.3, 26.9, 25.0, 25.0, 24.9, 24.7, 21.5.

^{11}B NMR (128.3 MHz, CDCl$_3$): δ 34.4.

HRMS (ESI) for **$C_{36}H_{52}B_2NO_6S^+$[M+H$^+$]$^+$:** calculated: 648.3696 , found: 648.3712.

_N_-(2-(Bis(4,4,5,5-tetramethyl-1,3,2-dioxaborolan-2-yl)(trimethylsilyl)methyl)-4-phenylbut-3-yn-1-yl)-4-methylbenzenesulfonamide (3e)

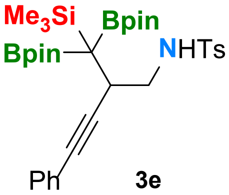

3e

The product was purified by flash chromatography using as eluent a mixture of pentane/diethyl ether (100:30). The product was isolated as a pale yellowish solid (96 mg, 75%).

^{1}H NMR (400 MHz, CDCl$_3$): δ 7.81 (d, J = 8.3 Hz, 2H), 7.28 (m, 5H), 7.25 – 7.20 (m, 2H), 5.45 (dd, J = 8.9, 3.0 Hz, 1H), 3.53 – 3.49 (m, 1H), 3.44 – 3.32 (m, 1H), 2.86 – 2.80 (m, 1H), 2.38 (s, 3H), 1.24 (s, 6H), 1.19 (s, 6H), 1.18 (s, 6H), 1.16 (s, 6H), 0.09 (s, 9H).

^{13}C NMR {^{1}H} (125 MHz, CDCl$_3$): δ = 143.0, 138.1, 131.5, 129.5, 128.3, 127.8, 127.4, 123.8, 92.3, 83.2, 82.9, 47.3, 31.7, 25.5, 25.3, 24.9, 24.8, 24.4, 21.5, 0.0.

^{11}B NMR (128.3 MHz, CDCl$_3$): δ 32.4.

HRMS (ESI) for **$C_{33}H_{50}B_2NO_6SSi^+$[M+H]$^+$:** calculated: 638.3334, found: 638.3321.

4-methyl-*N*-(1-phenyl-5,5-bis(4,4,5,5-tetramethyl-1,3,2-dioxaborolan-2-yl)hex-1-yn-3-yl)benzenesulfonamide (4b)

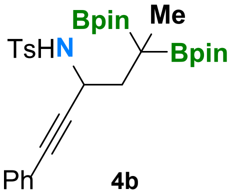

4b

The product was purified by flash chromatography using as eluent a mixture of pentane/diethyl ether (100:30). The product was isolated as a pale yellowish solid (8 mg, 4%).

^{1}H NMR (400 MHz, CDCl$_3$): δ = 7.77 (d, *J* = 8.3 Hz, 2H), 7.23 – 7.14 (m, 5H), 7.00 – 6.92 (m, 2H), 5.12 (d, *J* = 9.3 Hz, 1H), 4.41 (dd, *J* = 11.2, 9.3 Hz, 1H), 2.44 – 2.38 (m, 1H), 2.23 (s, 3H), 1.91 (dd, *J* = 14.6, 4.5 Hz, 1H), 1.33 (s, 6H), 1.31 (s, 6H), 1.21 (s, 12H), 1.18 (s, 3H).

^{13}C NMR {^{1}H} (125 MHz, CDCl$_3$): δ = 143.2, 138.0, 131.5, 129.4, 128.0, 127.9, 127.8, 122.7, 88.5, 84.0, 83.8, 83.4, 44.0, 39.1, 24.8, 24.8, 24.7, 24.7, 21.4, 14.8.

^{11}B NMR (128.3 MHz, CDCl$_3$): δ = 34.5

HRMS (ESI) for **$C_{31}H_{44}B_2NO_6S[M+H^+]^+$:** calculated: 580.3076, found: 580.3081

4-methyl-*N*-(1-phenyl-5,5-bis(4,4,5,5-tetramethyl-1,3,2-dioxaborolan-2-yl)-5-(trimethylsilyl)pent-1-yn-3-yl)benzenesulfonamide (4e)

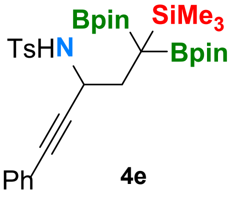

4e

The product was purified by flash chromatography using as eluent a mixture of pentane/diethyl ether (100:30). The product was isolated as a pale yellowish solid (9 mg, 7%).

^{1}H NMR (400 MHz, CDCl$_3$): δ = 7.80 – 7.72 (m, 2H), 7.25 – 7.15 (m, 3H), 7.15 – 7.10 (m, 2H), 7.01 – 6.95 (m, 2H), 6.11 (d, J = 8.3 Hz, 1H), 4.93 (ddd, J = 9.9, 8.3, 5.9 Hz, 1H), 2.24 (s, 3H), 2.15 – 2.01 (m, 2H), 1.33 (s, 6H), 1.30 (s, 6H), 1.28 (s, 6H), 1.26 (s, 6H), 0.13 (s, 9H).

^{13}C NMR {^{1}H} (125 MHz, CDCl$_3$): δ =142.6, 139.0, 131.6, 129.2, 127.9, 127.9, 127.5, 123.0, 89.4, 84.1, 83.6, 83.2, 46.0, 34.1, 25.8, 25.6, 24.8, 24.3, 21.4, -1.2.

^{11}B NMR (128.3 MHz, CDCl$_3$): δ = 34.1

HRMS (ESI) for **$C_{33}H_{50}B_2NO_6SSi^+[M+H]^+$:** calculated: 638.3334, found: 638.3338

***N*-(2-(bis(4,4,5,5-tetramethyl-1,3,2-dioxaborolan-2-yl)methyl)-4-(4-methoxyphenyl)but-3-yn-1-yl)-4-methylbenzenesulfonamide (6a)**

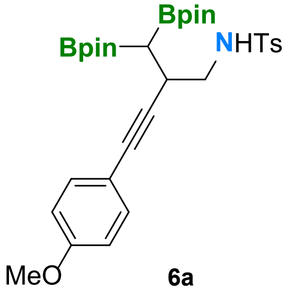

6a

The product was purified by flash chromatography using as eluent a mixture of pentane/diethyl ether (100:30). The product was isolated as a pale yellowish solid (82 mg, 68%).

^{1}H NMR (400 MHz, CDCl$_3$): δ 7.75 (d, *J* = 8.2 Hz, 2H), 7.27 – 7.21 (m, 4H), 6.83 – 6.75 (m, 2H), 5.00 – 4.92 (m, 1H), 3.79 (s, 3H), 3.29 – 3.22 (m, 1H), 3.10 – 2.95 (m, 2H), 2.40 (s, 3H), 1.21 (s, 6H), 1.19 (s, 6H), 1.12 (s, 12H), 1.10 (d, *J* = 9.9 Hz, 1H).

^{13}C NMR {^{1}H} (125 MHz, CDCl$_3$): δ = 159.3, 143.2, 137.2, 133.1, 129.7, 127.3, 115.5, 113.8, 89.9, 83.7, 83.6, 82.4, 55.3, 48.2, 29.6, 24.9, 24.5, 24.5, 21.6.

^{11}B NMR (128.3 MHz, $CDCl_3$): δ 31.6.

HRMS (ESI) for **$C_{31}H_{44}B_2NO_7S^+$[M+H]$^+$:** calculated: 596.3030, found: 596.3031.

N-(2-(1,1-bis(4,4,5,5-tetramethyl-1,3,2-dioxaborolan-2-yl)ethyl)-4-(4-methoxyphenyl)but-3-yn-1-yl)-4-methylbenzenesulfonamide (6b)

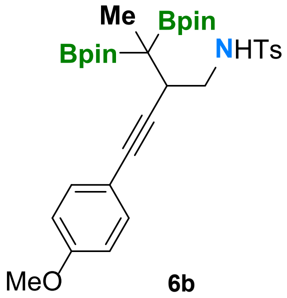

6b

The product was purified by flash chromatography using as eluent a mixture of pentane/diethyl ether (100:30). The product was isolated as a pale yellowish solid (75.2 mg, 61%).

^1H NMR (400 MHz, $CDCl_3$): δ 7.77 (d, *J* = 8.3 Hz, 2H), 7.28 – 7.26 (m, 2H), 7.26 – 7.23 (m, 2H), 6.80 (d, *J* = 8.7 Hz, 2H), 5.02 (dd, *J* = 8.4, 3.0 Hz, 1H), 3.80 (s, 3H), 3.26 (ddd, *J* = 10.1, 8.4, 4.7 Hz, 1H), 3.14 – 3.01 (m, 2H), 2.41 (s, 3H), 1.23 (s, 6H), 1.22 (s, 6H), 1.19 (s, 12H), 1.15 (s, 3H).

^{13}C NMR {^1H} (125 MHz, $CDCl_3$): δ = 159.3, 143.2, 137.2, 133.1, 129.6, 127.4, 115.7, 113.8, 88.6, 83.7, 83.6, 83.0, 55.3, 45.1, 35.5, 24.9, 24.8, 24.7, 24.6, 21.6, 12.8.

^{11}B NMR (128.3 MHz, $CDCl_3$): δ 32.9.

HRMS (ESI) for **$C_{32}H_{46}B_2NO_7S^+$[M+H$^+$]$^+$:** calculated: 610.3176 , found: 610.3181.

N-(2-(bis(4,4,5,5-tetramethyl-1,3,2-dioxaborolan-2-yl)(trimethylsilyl)methyl)-4-(4-methoxyphenyl)but-3-yn-1-yl)-4-methylbenzenesulfonamide (6e)

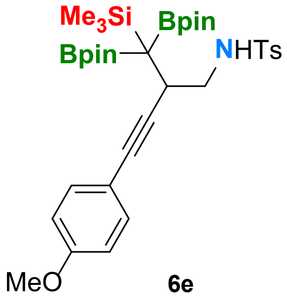

6e

The product was purified by flash chromatography using as eluent a mixture of pentane/diethyl ether (100:30). The product was isolated as a pale yellowish solid (108 mg, 81%).

^1H NMR (400 MHz, $CDCl_3$): δ 7.83 – 7.77 (m, 2H), 7.28 – 7.18 (m, 4H), 6.84 – 6.76 (m, 2H), 5.46 (dd, J = 9.1, 3.0 Hz, 1H), 3.78 (s, 3H), 3.49 (ddd, J = 12.2, 9.1, 3.0 Hz, 1H), 3.37 (ddd, J = 12.2, 9.1, 4.9 Hz, 1H), 2.81 (dd, J = 9.1, 4.9 Hz, 1H), 2.37 (s, 3H), 1.22 (s, 6H), 1.18 (s, 6H), 1.17 (s, 6H), 1.16 (s, 6H), 0.08 (s, 9H).

^{13}C NMR {^1H} (125 MHz, $CDCl_3$): δ = 159.2, 142.9, 138.1, 132.7, 129.4, 127.3, 115.9, 113.8, 90.6, 83.1, 83.0, 82.8, 55.3, 47.2, 31.6, 25.4, 25.2, 24.8, 24.3, 21.5, 0.0.

^{11}B NMR (128.3 MHz, $CDCl_3$): δ 31.9.

HRMS (ESI) for **$C_{34}H_{52}B_2NO_7SSi$ [M+H]$^+$:** calculated: 668.3427, found: 668.3423.

N-(2-(bis(4,4,5,5-tetramethyl-1,3,2-dioxaborolan-2-yl)methyl)-4-(4-chlorophenyl)but-3-yn-1-yl)-4-methylbenzenesulfonamide (9a)

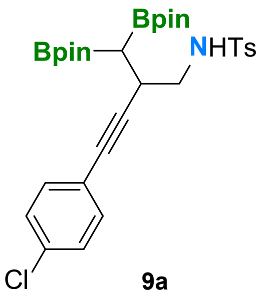

9a

The product was purified by flash chromatography using as eluent a mixture of pentane/diethyl ether (100:30). The product was isolated as a pale yellowish solid (85.2 mg, 71%).

^{1}H NMR (400 MHz, $CDCl_3$): δ 77.78 (d, *J* = 8.3 Hz, 2H), 7.30 – 7.26 (m, 6H), 5.01 – 4.92 (m, 1H), 3.34 – 3.24 (m, 1H), 3.13 – 3.01 (m, 2H), 2.42 (s, 3H), 1.24 (s, 6H), 1.22 (s, 6H), 1.22 (s, 6H), 1.21 (s, 6H), 1.14 (d, *J* = 9.8 Hz, 1H).

^{13}C NMR {^{1}H} (125 MHz, $CDCl_3$): δ = 143.3, 137.3, 133.9, 132.9, 129.7, 128.6, 127.3, 121.9, 92.7, 83.8, 83.6, 81.5, 48.0, 30.4, 29.7, 25.0, 24.9, 24.8, 24.5, 21.6.

^{11}B NMR (128.3 MHz, $CDCl_3$): δ 34.0.

HRMS (ESI) for **$C_{30}H_{41}B_2ClNO_6S^+$[M+H$^+$]$^+$:** calculated: 600.2535 , found: 600.2518.

N-(2-(1,1-bis(4,4,5,5-tetramethyl-1,3,2-dioxaborolan-2-yl)ethyl)-4-(4-chlorophenyl)but-3-yn-1-yl)-4-methylbenzenesulfonamide (9b)

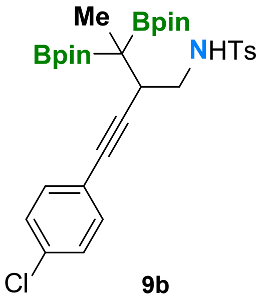

9b

The product was purified by flash chromatography using as eluent a mixture of pentane/diethyl ether (100:30). The product was isolated as a pale yellowish solid (62 mg, 50%).

^{1}H NMR (400 MHz, $CDCl_3$): δ 7.79 (d, 2H), 7.32 – 7.21 (m, 6H), 5.07 – 5.00 (m, 1H), 3.33 – 3.22 (m, 1H), 3.18 – 3.06 (m, 2H), 2.43 (s, 3H), 1.25 (s, 6H), 1.24 (s, 6H), 1.20 (s, 12H), 1.16 (s, 3H).

^{13}C NMR {^{1}H} (125 MHz, $CDCl_3$): δ = 143.2, 137.2, 133.9, 133.0, 129.7, 128.6, 127.3, 122.0, 91.5, 83.8, 83.6, 82.1, 45.0, 35.6, 24.9, 24.8, 24.7, 24.6, 21.6, 12.9.

^{11}B NMR (128.3 MHz, $CDCl_3$): δ 34.9.

HRMS (ESI) for **$C_{31}H_{43}B_2ClNO_6S^+$[M+H$^+$]$^+$:** calculated: 614.2680 , found: 614.2677.

N-(2-(bis(4,4,5,5-tetramethyl-1,3,2-dioxaborolan-2-yl)(trimethylsilyl)methyl)-4-(4-chlorophenyl)but-3-yn-1-yl)-4-methylbenzenesulfonamide (9e)

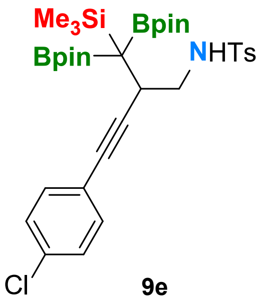

9e

The product was purified by flash chromatography using as eluent a mixture of pentane/diethyl ether (100:30). The product was isolated as a pale yellowish solid (104 mg, 77%).

^{1}H NMR (400 MHz, $CDCl_3$): δ 7.83 – 7.76 (m, 2H), 7.26 – 7.16 (m, 6H), 5.45 (dd, J = 8.9, 3.3 Hz, 1H), 3.49 (ddd, J = 12.3, 8.9, 3.3 Hz, 1H), 3.37 (ddd, J = 12.3, 8.9, 4.8 Hz, 1H), 2.85 (dd, J = 8.9, 4.8 Hz, 1H), 2.38 (s, 3H), 1.22 (s, 6H), 1.18 (s, 6H), 1.18 (s, 6H), 1.15 (s, 6H), 0.08 (s, 9H).

^{13}C NMR {^{1}H} (125 MHz, CDCl$_3$): δ = 143.0, 138.1, 133.7, 132.7, 129.5, 128.6, 127.3, 122.3, 93.6, 83.2, 83.0, 82.1, 47.3, 31.8, 25.5, 25.3, 24.9, 24.4, 21.5, 0.0.

^{11}B NMR (128.3 MHz, CDCl$_3$): δ 32.1.

HRMS (ESI) for **$C_{33}H_{49}B_2ClNO_6SSi$ [M+H]$^+$:** calculated: 672.2924, found: 672.2929.

***N*-(2-(bis(4,4,5,5-tetramethyl-1,3,2-dioxaborolan-2-yl)(trimethylsilyl)methyl)-4-(4-chlorophenyl)but-3-yn-1-yl)-4-methylbenzenesulfonamide (10e)**

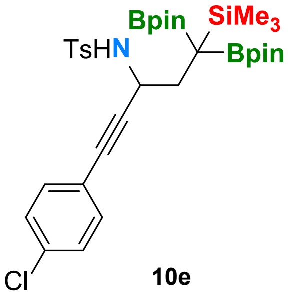

10e

The product was purified by flash chromatography using as eluent a mixture of pentane/diethyl ether (100:30). The product was isolated as a pale yellowish solid (11 mg, 8%).

^{1}H NMR (400 MHz, CDCl$_3$): δ = 7.78 – 7.71 (m, 2H), 7.20 – 7.10 (m, 4H), 6.94 – 6.87 (m, 2H), 6.15 (d, J = 8.1 Hz, 1H), 4.92 (ddd, J = 10.3, 8.1, 5.5 Hz, 1H), 2.26 (s, 3H), 2.10 – 2.00 (m, 2H), 1.32 (s, 6H), 1.29 (s, 6H), 1.27 (s, 6H), 1.26 (s, 6H), 0.12 (s, 9H).

^{13}C NMR {^{1}H} (125 MHz, CDCl$_3$): δ = 142.6, 139.1, 133.9, 132.9, 129.2, 128.2, 127.5, 121.5, 90.5, 83.7, 83.2, 83.1, 46.0, 34.0, 25.8, 25.6, 24.8, 24.3, 21.4, -1.2

^{11}B NMR (128.3 MHz, CDCl$_3$): δ = 34.0

HRMS (ESI) for **$C_{33}H_{49}B_2ClNO_6SSi$ [M+H]$^+$:** calculated: 672.2924, found: 672.2931.

***N*-(2-(bis(4,4,5,5-tetramethyl-1,3,2-dioxaborolan-2-yl)methyl)-4-(naphthalen-2-yl)but-3-yn-1-yl)-4-methylbenzenesulfonamide (12a)**

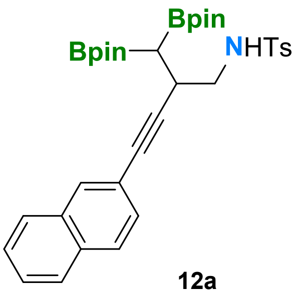

12a

The product was purified by flash chromatography using as eluent a mixture of pentane/diethyl ether (100:30). The product was isolated as a pale yellowish solid (51 mg, 41%).

^{1}H NMR (400 MHz, CDCl$_3$): δ 7.87 – 7.70 (m, 6H), 7.52 – 7.42 (m, 2H), 7.36 (dd, *J* = 8.5, 1.6 Hz, 1H), 7.25 (d, *J* = 8.3 Hz, 2H), 5.02 (dd, *J* = 7.3, 4.8 Hz, 1H), 3.32 (ddd, *J* = 11.5, 7.0, 4.4 Hz, 1H), 3.20 – 3.01 (m, 2H), 2.37 (s, 3H), 1.23 (s, 6H), 1.22 (s, 6H), 1.20 (d, *J* = 1.3 Hz, 12H), 1.17 (d, *J* = 9.9 Hz, 1H).

^{13}C NMR {^{1}H} (125 MHz, CDCl$_3$): δ = 143.2, 137.3, 133.0, 132.7, 131.4, 129.8, 129.7, 128.7, 127.9, 127.8, 127.7, 127.7, 127.3, 127.2, 126.6, 126.5, 120.7, 91.9, 83.8, 83.6, 82.9, 48.1, 29.8, 25.0, 25.0, 24.5, 24.5, 21.6.

^{11}B NMR (128.3 MHz, CDCl$_3$): δ 33.6.

HRMS (ESI) for **$C_{34}H_{44}B_2NO_6S^+$[M+H]$^+$:** calculated: 616.3082, found: 616.3083.

***N*-(2-(1,1-bis(4,4,5,5-tetramethyl-1,3,2-dioxaborolan-2-yl)ethyl)-4-(naphthalen-2-yl)but-3-yn-1-yl)-4-methylbenzenesulfonamide (12b)**

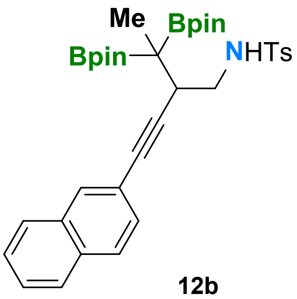

12b

The product was purified by flash chromatography using as eluent a mixture of pentane/diethyl ether (100:30). The product was isolated as a pale yellowish solid (73 mg, 61%).

^{1}H NMR (400 MHz, CDCl$_3$): δ 7.89 – 7.68 (m, 6H), 7.55 – 7.42 (m, 2H), 7.36 (dd, *J* = 8.5, 1.6 Hz, 1H), 7.25 (d, *J* = 7.8 Hz, 2H), 5.06 (dd, *J* = 8.4, 3.3 Hz, 1H), 3.30 (ddd, *J* = 10.3, 8.2, 5.0 Hz, 1H), 3.21 – 3.08 (m, 2H), 2.37 (s, 3H), 1.23 (s, 6H), 1.23 (s, 6H), 1.19 (s, 3H), 1.19 (s, 12H).

^{13}C NMR {^{1}H} (125 MHz, CDCl$_3$): δ = 143.2, 137.2, 133.1, 132.7, 131.4, 129.7, 128.8, 127.9, 127.8, 127.7, 127.4, 126.5, 120.8, 90.7, 83.8, 83.7, 83.6, 45.1, 35.7, 24.9, 24.8, 24.7, 24.6, 21.6, 12.9.

^{11}B NMR (128.3 MHz, CDCl$_3$): δ 33.5.

HRMS (ESI) for **$C_{35}H_{46}B_2NO_6S^+$[M+H$^+$]$^+$:** calculated: 630.3226, found: 630.3247.

***N*-(2-(bis(4,4,5,5-tetramethyl-1,3,2-dioxaborolan-2-yl)(trimethylsilyl)methyl)-4-(naphthalen-2-yl)but-3-yn-1-yl)-4-methylbenzenesulfonamide (12e)**

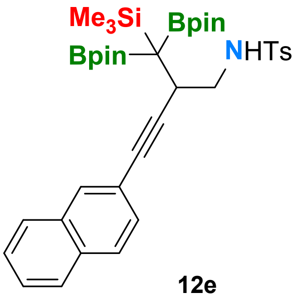

12e

The product was purified by flash chromatography using as eluent a mixture of pentane/diethyl ether (100:30). The product was isolated as a pale yellowish solid (84 mg, 61%).

^{1}H NMR (400 MHz, CDCl$_3$): δ 7.85 – 7.72 (m, 6H), 7.53 – 7.43 (m, 2H), 7.35 (m, 1H), 7.28 – 7.20 (m, 2H), 5.52 (dd, J = 8.9, 3.1 Hz, 1H), 3.56 (ddd, J = 12.4, 9.0, 3.1 Hz, 1H), 3.42 (ddd, J = 12.4, 8.9, 4.9 Hz, 1H), 2.90 (dd, J = 9.0, 4.9 Hz, 1H), 2.35 (s, 3H), 1.25 (s, 6H), 1.21 (s, 6H), 1.20 (s, 6H), 1.18 (s, 6H), 0.12 (s, 9H).

^{13}C NMR {^{1}H} (125 MHz, CDCl$_3$): δ = 143.0, 138.2, 133.1, 132.6, 131.1, 129.5, 128.5, 127.9, 127.8, 127.7, 127.4, 126.5, 126.5, 121.2, 92.8, 83.5, 83.2, 83.0, 47.3, 31.8, 25.5, 25.3, 24.9, 24.4, 21.5, 0.1

^{11}B NMR (128.3 MHz, CDCl$_3$): δ 34.3.

HRMS (ESI) for **$C_{37}H_{52}B_2NO_6SSi$ [M+H]$^+$:** calculated: 688.3474, found: 688.3478.

N-(2-(bis(4,4,5,5-tetramethyl-1,3,2-dioxaborolan-2-yl)methyl)-4-cyclohexylbut-3-yn-1-yl)-4-methylbenzenesulfonamide (15a)

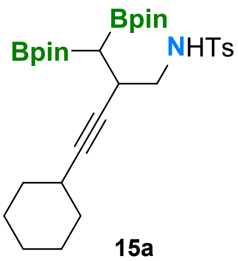

15a

The product was purified by flash chromatography using as eluent a mixture of pentane/diethyl ether (100:30). The product was isolated as a pale yellowish solid (46 mg, 40%).

^{1}H NMR (400 MHz, CDCl$_3$): δ 7.75 (d, *J* = 8.4 Hz, 2H), 7.28 (d, *J* = 8.3 Hz, 2H), 4.85 (dd, *J* = 7.4, 4.3 Hz, 1H), 3.20 – 3.11 (m, 1H), 2.91 – 2.77 (m, 2H), 2.42 (s, 3H), 2.26 (m,1H), 1.74 – 1.44 (m, 10H), 1.21 (s, 6H), 1.21 (s, 6H), 1.19 (s, 6H), 1.17 (s, 6H), 0.98 (d, *J* = 9.9 Hz, 1H).

^{13}C NMR {^{1}H} (125 MHz, CDCl$_3$): δ = 143.1, 137.3, 129.7, 127.3, 87.0, 83.6, 83.5, 81.5, 65.1, 48.3, 37.4, 33.0, 30.4, 29.1, 28.9, 26.0, 24.9, 24.6, 24.5, 21.6, 18.5.

^{11}B NMR (128.3 MHz, CDCl$_3$): δ 33.3.

HRMS (ESI) for **$C_{30}H_{48}B_2NO_6S^+$[M+H$^+$]$^+$:** calculated: 572.3383, found: 572.3379.

N -(2-(1,1-bis(4,4,5,5-tetramethyl-1,3,2-dioxaborolan-2-yl)ethyl)-4-cyclohexylbut-3-yn-1-yl)-4-methylbenzenesulfonamide (15b)

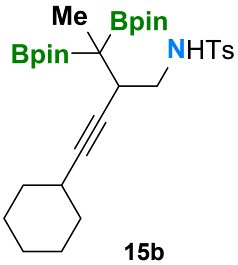

15b

The product was purified by flash chromatography using as eluent a mixture of pentane/diethyl ether (100:30). The product was isolated as a pale yellowish solid (72 mg, 61%).

^{1}H NMR (400 MHz, CDCl$_3$): δ 7.75 (d, *J* = 8.3 Hz, 2H), 7.28 (d, *J* = 8.2 Hz, 2H), 4.89 (dd, *J* = 8.6, 3.0 Hz, 1H), 3.15 (ddd, *J* = 11.0, 8.7, 5.7 Hz, 1H), 2.96 – 2.81 (m, 2H), 2.42 (s, 3H), 2.30 – 2.22 (m, 1H), 1.38 – 1.20 (m, 10H), 1.19 (s, 12H), 1.19 (s, 6H), 1.18 (s, 6H), 1.05 (s, 3H).

^{13}C NMR {^{1}H} (125 MHz, CDCl$_3$): δ = 143.1, 137.3, 129.6, 127.4, 87.8, 83.6, 83.4, 79.9, 45.3, 34.8, 33.1, 33.0, 29.2, 26.0, 25.0, 24.9, 24.8, 24.7, 24.6, 21.6, 12.7.

^{11}B NMR (128.3 MHz, CDCl$_3$): δ 34.8.

HRMS (ESI) for **$C_{31}H_{50}B_2NO_6S^+$[M+H]$^+$:** calculated: 586.3550, found: 586.3539.

N-(2-(bis(4,4,5,5-tetramethyl-1,3,2-dioxaborolan-2-yl)(trimethylsilyl)methyl)-4-cyclohexylbut-3-yn-1-yl)-4-methylbenzenesulfonamide (15e)

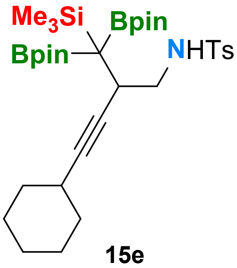

15e

The product was purified by flash chromatography using as eluent a mixture of pentane/diethyl ether (100:30). The product was isolated as a pale yellowish solid (99 mg, 77%).

^{1}H NMR (400 MHz, CDCl$_3$): δ 7.78 (d, J = 8.3 Hz, 2H), 7.26 (d, J = 8.0 Hz, 2H), 5.32 – 5.24 (m, 1H), 3.41 – 3.30 (m, 1H), 3.30 – 3.20 (m, 1H), 2.56 – 2.48 (m, 1H), 2.40 (s, 3H), 2.27 – 2.18 (m, 1H), 1.77 – 1.60 (m, 5H), 1.57 – 1.45 (m, 1H), 1.39 – 1.23 (m, 4H), 1.21 (s, 6H), 1.17 (s, 6H), 1.16 (s, 6H), 1.15 (s, 6H), 0.03 (s, 9H).

^{13}C NMR {^{1}H} (125 MHz, $CDCl_3$): δ = 142.9, 138.2, 129.4, 127.4, 87.5, 83.0, 82.7, 81.8, 47.3, 33.0, 33.0, 30.8, 29.3, 26.0, 25.5, 25.2, 24.9, 24.5, 21.5, 0.1

^{11}B NMR (128.3 MHz, $CDCl_3$): δ 32.3.

HRMS (ESI) for **$C_{33}H_{56}B_2NO_6SSi$ $[M+H]^+$:** calculated: 644.3779, found: 644.3790.

N-(1-cyclohexyl-5,5-bis(4,4,5,5-tetramethyl-1,3,2-dioxaborolan-2-yl)-5-(trimethylsilyl)pent-1-yn-3-yl)-4-methylbenzenesulfonamide (16e)

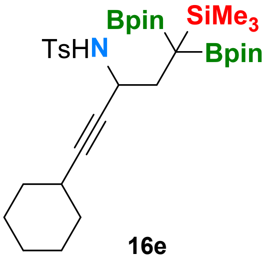

16e

The product was purified by flash chromatography using as eluent a mixture of pentane/diethyl ether (100:30). The product was isolated as a pale yellowish solid (15 mg, 12%).

^{1}H NMR (400 MHz, $CDCl_3$): δ = 7.74 (d, J = 8.3 Hz, 2H), 7.24 (d, J = 8.3 Hz, 2H), 6.01 (d, J = 7.8 Hz, 1H), 4.73 – 4.62 (m, 1H), 2.39 (s, 3H), 1.96 – 1.90 (m, 2H), 1.92 – 1.81 (m, 1H), 1.59 – 1.40 (m, 6H), 1.32 (s, 6H), 1.29 (s, 6H), 1.27 (s, 6H), 1.25 (s, 6H), 1.22 – 1.08 (m, 2H), 0.98 (tt, J = 11.0, 3.2 Hz, 2H), 0.11 (s, 9H).

^{13}C NMR {^{1}H} (125 MHz, $CDCl_3$): δ = 142.2, 139.6, 129.1, 127.5, 88.1, 83.6, 83.1, 79.8, 45.6, 34.7, 32.5, 32.5, 29.2, 25.9, 25.6, 25.3, 24.7, 24.2, 21.5, -1.2

^{11}B NMR (128.3 MHz, $CDCl_3$): δ = 33.2

HRMS (ESI) for **$C_{33}H_{56}B_2NO_6SSi$ $[M+H]^+$:** calculated: 644.3779, found: 644.3778.

N -(2-(bis(4,4,5,5-tetramethyl-1,3,2-dioxaborolan-2-yl)methyl)dec-3-yn-1-yl)-4-methylbenzenesulfonamide (18a)

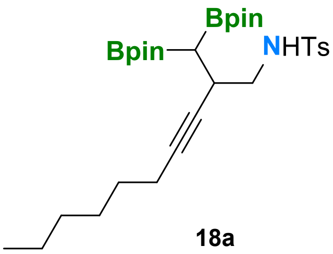

18a

The product was purified by flash chromatography using as eluent a mixture of pentane/diethyl ether (100:30). The product was isolated as a pale yellowish solid (44.8 mg, 39%).

^{1}H NMR (400 MHz, $CDCl_3$): δ 7.74 (d, *J* = 8.3 Hz, 2H), 7.27 (d, *J* = 8.3 Hz, 2H), 4.86 (dd, *J* = 7.4, 4.5 Hz, 1H), 3.16 (ddd, *J* = 10.9, 7.4, 4.7 Hz, 1H), 2.93 – 2.72 (m, 2H), 2.41 (s, 3H), 2.06 (t, *J* = 7.1, 2H), 1.46 – 1.36 (m, 2H), 1.35 – 1.23 (m, 6H), 1.21 (s, 6H), 1.21 (s, 6H), 1.19 (s, 6H), 1.17 (s, 6H), 0.99 (d, *J* = 10.2 Hz, 1H), 0.88 (t, *J* = 7.0 Hz, 3H).

^{13}C NMR {^{1}H} (125 MHz, $CDCl_3$): δ = 143.1, 137.3, 129.6, 127.3, 83.6, 83.5, 82.8, 81.6, 48.3, 31.5, 29.0, 28.9, 28.7, 24.9, 24.9, 24.6, 24.5, 22.6, 21.6, 18.8, 14.2.

^{11}B NMR (128.3 MHz, $CDCl_3$): δ 33.8.

HRMS (ESI) for **$C_{30}H_{50}B_2NO_6S^+$$[M+H^+]^+$:** calculated: 574.3595 , found: 574.3552.

N-(2-(1,1-bis(4,4,5,5-tetramethyl-1,3,2-dioxaborolan-2-yl)ethyl)dec-3-yn-1-yl)-4-methylbenzenesulfonamide (18b)

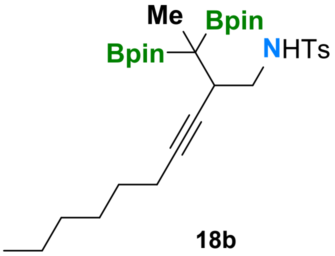

18b

The product was purified by flash chromatography using as eluent a mixture of pentane/diethyl ether (100:30). The product was isolated as a pale yellowish solid (51 mg, 38%).

^{1}H NMR (400 MHz, CDCl$_3$): δ 7.75 (d, *J* = 8.3 Hz, 2H), 7.29 – 7.26 (m, 2H), 4.91 (dd, *J* = 8.6, 3.0 Hz, 1H), 3.14 (ddd, *J* = 11.0, 8.7, 6.0 Hz, 1H), 2.97 – 2.80 (m, 2H), 2.41 (s, 3H), 2.06 (t, *J* = 7.0 Hz, 2H), 1.47 – 1.34 (m, 2H), 1.35 – 1.22 (m, 6H), 1.20 (s, 6H), 1.19 (s, 6H), 1.19 (s, 12H), 1.05 (s, 3H), 0.88 (t, *J* = 7.0 Hz, 3H).

^{13}C NMR {^{1}H} (125 MHz, CDCl$_3$): δ = 143.1, 137.3, 129.6, 127.4, 83.6, 83.5, 83.4, 80.1, 45.2, 34.8, 31.5, 29.0, 28.7, 24.8, 24.8, 24.7, 24.6, 22.6, 21.6, 18.8, 14.2, 12.6.

^{11}B NMR (128.3 MHz, CDCl$_3$): δ 34.7.

HRMS (ESI) for **$C_{31}H_{52}B_2NO_6S^+$[M+H$^+$]$^+$:** calculated: 588.3706 , found: 588.3711.

N-(2-(bis(4,4,5,5-tetramethyl-1,3,2-dioxaborolan-2-yl)(trimethylsilyl)methyl)dec-3-yn-1-yl)-4-methylbenzenesulfonamide (18e)

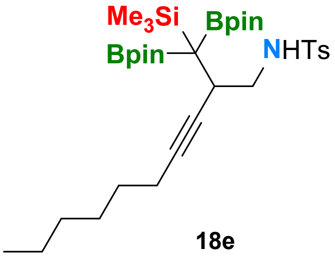

18e

The product was purified by flash chromatography using as eluent a mixture of pentane/diethyl ether (100:30). The product was isolated as a pale yellowish solid (94 mg, 73%).

^{1}H NMR (400 MHz, CDCl$_3$): δ 7.79 (d, *J* = 8.3 Hz, 2H), 7.28 – 7.24 (m, 2H), 5.29 (dd, *J* = 9.3, 2.5 Hz, 1H), 3.37 (ddd, *J* = 12.0, 9.4, 2.6 Hz, 1H), 3.27 (ddd, *J* = 12.1, 9.4, 4.9 Hz, 1H), 2.53 (ddt, *J* = 9.3, 4.6, 2.1 Hz, 1H), 2.40 (s, 3H), 1.56 (d, *J* = 5.2 Hz, 1H), 1.45 – 1.22 (m, 8H), 1.21 (s, 6H), 1.17 (s, 6H), 1.16 (s, 6H), 1.15 (s, 6H), 0.88 (t, *J* = 5.2 Hz, 3H), 0.03 (s, 9H).

^{13}C NMR {^{1}H} (125 MHz, CDCl$_3$): δ = 142.9, 138.3, 129.4, 127.4, 83.3, 83.1, 82.7, 82.0, 47.3, 31.5, 31.0, 28.9, 28.8, 25.5, 25.1, 24.9, 24.4, 22.7, 21.5, 18.9, 14.2.

^{11}B NMR (128.3 MHz, CDCl$_3$): δ 33.8.

HRMS (ESI) for **$C_{33}H_{58}B_2NO_6SSi^+$[M+H$^+$]$^+$:** calculated: 646.3935, found: 646.3943.

N-(1,1-bis(4,4,5,5-tetramethyl-1,3,2-dioxaborolan-2-yl)-1-(trimethylsilyl)undec-4-yn-3-yl)-4-methylbenzenesulfonamide (19e)

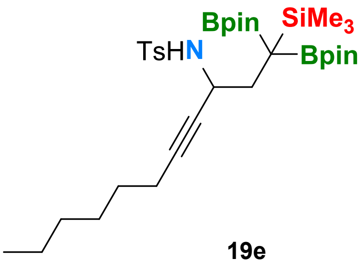

19e

The product was purified by flash chromatography using as eluent a mixture of pentane/diethyl ether (100:30). The product was isolated as a pale yellowish solid (27 mg, 11%).

^{1}H NMR (400 MHz, CDCl$_3$): δ 7.74 (d, *J* = 8.3 Hz, 2H), 7.25 – 7.21 (m, 2H), 6.03 (d, *J* = 7.8 Hz, 1H), 4.68 (m, 1H), 2.39 (s,

3H), 1.96 – 1.90 (m, 2H), 1.76 – 1.70 (m, 2H), 1.59 – 1.40 (m, 6H), 1.28 (s, 6H), 1.27 (s, 6H), 1.25 (s, 6H), 1.17 (s, 6H), 0.89 (t, *J* = 7.2 Hz, 4H), 0.10 (s, 9H)

^{13}C NMR {^{1}H} (125 MHz, CDCl$_3$): δ = 142.2, 139.5, 129.0, 127.5, 84.3, 83.6, 83.1, 80.0, 45.7, 34.5, 31.4, 28.8, 28.5, 25.9, 25.6, 24.7, 24.2, 22.6, 21.5, 18.8, 14.1.

^{11}B NMR (128.3 MHz, CDCl$_3$): δ 33.4.

HRMS (ESI) for **$C_{33}H_{58}B_2NO_6SSi^+$[M+H$^+$]$^+$:** calculated: 646.3935, found: 646.3943.

_N_ -(2-(bis(4,4,5,5-tetramethyl-1,3,2-dioxaborolan-2-yl)methyl)-6-phenylhex-3-yn-1-yl)-4-methylbenzenesulfonamide (21a)

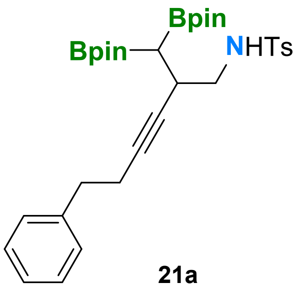

21a

The product was purified by flash chromatography using as eluent a mixture of pentane/diethyl ether (100:30). The product was isolated as a pale yellowish solid (60.8 mg, 51%).

^{1}H NMR (400 MHz, CDCl$_3$): δ 77.71 (d, 2H), 7.32 – 7.27 (m, 3H), 7.26 – 7.16 (m, 4H), 4.71 (dd, *J* = 7.5, 4.5 Hz, 1H), 3.18 – 3.10 (m, 1H), 2.88 – 2.77 (m, 2H), 2.73 (t, *J* = 7.5 Hz, 2H), 2.41 (s, 3H), 2.37 (t, *J* = 7.6 Hz, 2H), 1.21 (s, 6H), 1.20 (s, 12H), 1.17 (s, 6H), 0.99 (d, *J* = 10.1 Hz, 1H).

^{13}C NMR {^{1}H} (125 MHz, CDCl$_3$): δ = 143.1, 140.8, 137.4, 129.6, 128.6, 128.5, 127.3, 126.5, 83.6, 83.5, 82.7, 81.7, 48.2, 35.3, 29.0, 24.9, 24.9, 24.5, 24.5, 21.6, 21.1.

^{11}B NMR (128.3 MHz, CDCl$_3$): δ 33.3.

HRMS (ESI) for **$C_{32}H_{46}B_2NO_6S^+$[M+H$^+$]$^+$:** calculated: 594.3226 , found: 594.3239.

***N*-(2-(1,1-bis(4,4,5,5-tetramethyl-1,3,2-dioxaborolan-2-yl)ethyl)-6-phenylhex-3-yn-1-yl)-4-methylbenzenesulfonamide (21b)**

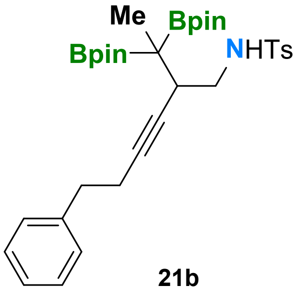

21b

The product was purified by flash chromatography using as eluent a mixture of pentane/diethyl ether (100:30). The product was isolated as a pale yellowish solid (78 mg, 64%).

^{1}H NMR (400 MHz, CDCl$_3$): δ 7.72 (d, 2H), 7.32 – 7.27 (m, 4H), 7.23 – 7.15 (m, 3H), 4.80 (dd, *J* = 8.7, 3.0 Hz, 1H), 3.19 – 3.08 (m, 1H), 2.95 – 2.79 (m, 2H), 2.74 (t, *J* = 7.5 Hz, 2H), 2.41 (s, 3H), 2.37 (t, *J* = 7.6 Hz, 2H), 1.20 (s, 6H), 1.19 (s, 12H), 1.18 (s, 6H), 1.03 (s, 3H).

^{13}C NMR {^{1}H} (125 MHz, CDCl$_3$): δ = 143.1, 140.8, 137.3, 129.6, 128.5, 128.5, 127.3, 126.4, 83.6, 83.5, 82.3, 81.1, 45.2, 35.4, 34.9, 24.8, 24.8, 24.7, 24.6, 21.6, 21.0, 12.6

^{11}B NMR (128.3 MHz, CDCl$_3$): δ 34-7.

HRMS (ESI) for **$C_{33}H_{48}B_2NO_6S^+$[M+H$^+$]$^+$:** calculated: 608.3383 , found: 608.3372.

N-(2-(bis(4,4,5,5-tetramethyl-1,3,2-dioxaborolan-2-yl)(trimethylsilyl)methyl)-6-phenylhex-3-yn-1-yl)-4-methylbenzenesulfonamide (21e)

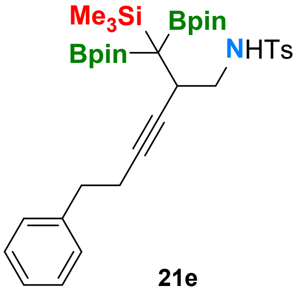

21e

The product was purified by flash chromatography using as eluent a mixture of pentane/diethyl ether (100:30). The product was isolated as a pale yellowish solid (86 mg, 65%).

^{1}H NMR (400 MHz, $CDCl_3$): δ 7.73 (d, *J* = 8.2 Hz, 2H), 7.31 (dd, *J* = 8.0, 6.7 Hz, 2H), 7.25 – 7.18 (m, 5H), 5.10 (dd, *J* = 9.4, 2.7 Hz, 1H), 3.44 – 3.20 (m, 2H), 2.84 – 2.64 (m, 2H), 2.48 (bs, 1H), 2.39 (s, 3H), 2.38 – 2.30 (m, 2H), 1.21 (s, 6H), 1.18 (s, 6H), 1.16 (s, 6H), 1.16 (s, 6H), 0.02 (s, 9H).

^{13}C NMR {^{1}H} (125 MHz, $CDCl_3$): δ =142.8, 140.9, 138.4, 129.4, 128.6, 128.4, 127.3, 126.5, 83.1, 83.0, 82.8, 82.3, 47.2, 35.3, 31.1, 25.5, 25.2, 24.9, 24.4, 21.5, 21.1, 0.0.

^{11}B NMR ($CDCl_3$, 128.3 MHz) δ 33.9.

HRMS (ESI) for **$C_{35}H_{54}B_2NO_6SSi^+$[M+H]$^+$:** calculated: 666.3634, found: 666.3635.

4-methyl-N-(8-phenyl-2,2-bis(4,4,5,5-tetramethyl-1,3,2-dioxaborolan-2-yl)oct-5-yn-4-yl)benzenesulfonamide (22b)

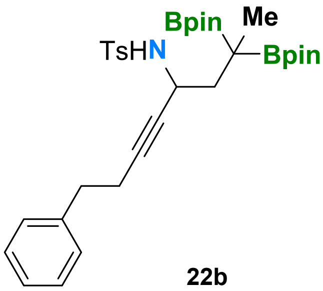

22b

The product was purified by flash chromatography using as eluent a mixture of pentane/diethyl ether (100:30). The product was isolated as a pale yellowish solid (8 mg, 2%).

^{1}H NMR (400 MHz, $CDCl_3$): δ 7.73 (d, *J* = 8.3 Hz, 2H), 7.32 – 7.27 (m, 2H), 7.24 – 7.17 (m, 3H), 7.12 – 7.04 (m, 2H), 4.99 (d, *J* = 9.2 Hz, 1H), 4.15 (tdd, *J* = 9.2, 4.4, 2.3 Hz, 1H), 2.48 – 2.40 (m, 3H), 2.37 (s, 3H), 2.10 (dd, *J* = 14.6, 11.3 Hz, 1H), 2.02 – 1.96 (m, 2H), 1.77 (dd, *J* = 14.6, 4.5 Hz, 1H), 1.31 (s, 6H), 1.29 (s, 6H), 1.22 (s, 12H), 1.11 (s, 3H).

^{13}C NMR {^{1}H} (125 MHz, $CDCl_3$): δ = 142.8, 140.8, 138.3, 129.2, 128.4, 128.4, 127.9, 126.4, 83.8, 83.6, 83.4, 80.0, 43.6, 39.4, 34.9, 25.4, 24.8, 24.8, 24.7, 24.7, 21.6, 20.9, 14.7.

^{11}B NMR (128.3 MHz, $CDCl_3$): δ 34.6.

HRMS (ESI) for **$C_{33}H_{48}B_2NO_6S^+$[M+H$^+$]$^+$:** calculated: 608.3383 , found: 608.3372.

N-(2-(bis(4,4,5,5-tetramethyl-1,3,2-dioxaborolan-2-yl)methyl)-2-methyl-4-phenylbut-3-yn-1-yl)-4-methylbenzenesulfonamide (24a)

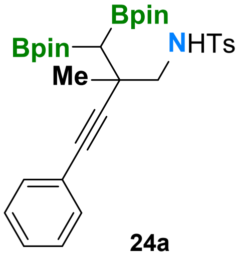

24a

The product was purified by flash chromatography using as eluent a mixture of pentane/diethyl ether (100:30). The product was isolated as a pale yellowish solid (41.2 mg, 35%).

^{1}H NMR (400 MHz, $CDCl_3$): δ 7.76 (d, *J* = 8.3 Hz, 2H), 7.33 – 7.28 (m, 2H), 7.26 – 7.22 (m, 5H), 5.39 (t, *J* = 6.2 Hz, 1H), 3.29 – 3.14 (m, 2H), 2.39 (s, 3H), 1.41 (s, 3H), 1.28 (s, 1H), 1.20 (s, 12H), 1.19 (s, 6H), 1.19 (s, 6H).

^{13}C NMR {^{1}H} (125 MHz, CDCl$_3$): δ = 143.0, 137.5, 131.7, 129.6, 128.3, 128.2, 127.8, 127.3, 123.6, 95.4, 83.5, 83.4, 81.8, 52.6, 35.1, 26.3, 24.9, 24.9, 24.5, 21.6, 15.4.

^{11}B NMR (128.3 MHz, CDCl$_3$): δ 32.1.

HRMS (ESI) for **$C_{31}H_{44}B_2NO_6S^+$[M+H]$^+$:** calculated: 580.3081, found: 580.3082.

N-(2-(1,1-bis(4,4,5,5-tetramethyl-1,3,2-dioxaborolan-2-yl)ethyl)-2-methyl-4-phenylbut-3-yn-1-yl)-4-methylbenzenesulfonamide (24b)

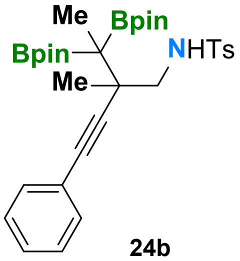

24b

The product was purified by flash chromatography using as eluent a mixture of pentane/diethyl ether (100:30). The product was isolated as a pale yellowish solid (71.2 mg, 60%).

^{1}H NMR (400 MHz, CDCl$_3$): δ 7.76 (d, *J* = 8.3 Hz, 2H), 7.36 – 7.29 (m, 2H), 7.28 – 7.23 (m, 5H), 5.41 (dd, *J* = 7.4, 5.1 Hz, 1H), 3.40 – 3.22 (m, 2H), 2.40 (s, 3H), 1.37 (s, 3H), 1.21 (s, 3H), 1.19 (s, 12H), 1.17 (s, 6H), 1.17 (s, 6H).

^{13}C NMR {^{1}H} (125 MHz, CDCl$_3$): δ = 142.8, 137.4, 131.6, 129.4, 128.0, 127.7, 127.2, 123.4, 95.3, 83.4, 83.3, 81.7, 52.4, 34.9, 26.2, 24.8, 24.4, 21.4.

^{11}B NMR (128.3 MHz, CDCl$_3$): δ 32.9.

HRMS (ESI) for **$C_{32}H_{46}B_2NO_6S^+$[M+H]$^+$:** calculated: 594.3238, found: 594.3246.

N-(2-(bis(4,4,5,5-tetramethyl-1,3,2-dioxaborolan-2-yl)(trimethylsilyl)methyl)-2-methyl-4-phenylbut-3-yn-1-yl)-4-methylbenzenesulfonamide (24e)

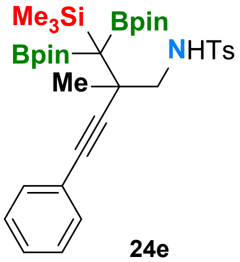

24e

The product was purified by flash chromatography using as eluent a mixture of pentane/diethyl ether (100:30). The product was isolated as a pale yellowish solid (60 mg, 41%).

^{1}H NMR (400 MHz, CDCl$_3$): δ 7.78 – 7.73 (m, 2H), 7.36 – 7.30 (m, 2H), 7.29 – 7.12 (m, 5H), 5.32 (dd, J = 8.8, 3.6 Hz, 1H), 3.49 (dd, J = 11.4, 8.8 Hz, 1H), 3.29 (dd, J = 11.4, 3.6 Hz, 1H), 2.40 (s, 3H), 1.47 (s, 3H), 1.18 (s, 12H), 1.13 (s, 6H), 1.12 (s, 6H), 0.21 (s, 9H).

^{13}C NMR {^{1}H} (125 MHz, CDCl$_3$): δ = 142.9, 137.4, 131.4, 129.5, 128.2, 127.7, 127.3, 123.9, 96.3, 83.1, 83.0, 82.9, 52.7, 37.8, 26.8, 25.0, 24.9, 24.9, 24.9, 21.6, 2.7.

^{11}B NMR (128.3 MHz, CDCl$_3$): δ 33.4

HRMS (ESI) for **$C_{34}H_{52}B_2NO_6SSi$ [M+H]$^+$:** calculated: 652.3465, found: : 652.3478.

***N*-(1-cyclohexyl-3-methyl-5,5-bis(4,4,5,5-tetramethyl-1,3,2-dioxaborolan-2-yl)-5-(trimethylsilyl)pent-1-yn-3-yl)-4-methylbenzenesulfonamide (25e)**

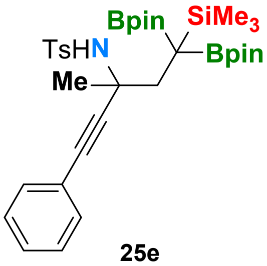

25e

The product was purified by flash chromatography using as eluent a mixture of pentane/diethyl ether (100:30). The product was isolated as a pale yellowish solid (52 mg, 40%).

^{1}H NMR (400 MHz, $CDCl_3$): δ 7.83 – 7.79 (m, 2H), 7.34 – 7.29 (m, 2H), 7.24 – 7.17 (m, 5H), 7.11 (s, 1H), 2.37 (s, 3H), 2.25 (d, J = 14.1 Hz, 1H), 2.12 (d, J = 14.1 Hz, 1H), 1.68 (s, 3H), 1.26 (s, 6H), 1.24 (s, 6H), 1.06 (s, 12H), 0.13 (s, 9H).

^{13}C NMR {^{1}H} (125 MHz, $CDCl_3$): δ = 142.6, 139.5, 132.7, 129.4, 128.1, 127.9, 127.7, 123.1, 92.6, 86.5, 83.5, 83.0, 56.3, 44.5, 28.6, 26.1, 26.0, 25.0, 24.5, 21.5, -1.1.

^{11}B NMR (128.3 MHz, $CDCl_3$): δ 32.5.

HRMS (ESI) for **$C_{34}H_{52}B_2NO_6SSi$ [M+H]$^+$:** calculated: 652.3465, found: : 652.3465.

***N*-(2-(bis(4,4,5,5-tetramethyl-1,3,2-dioxaborolan-2-yl)(trimethylsilyl)methyl)-2-methyl-4-(naphthalen-2-yl)but-3-yn-1-yl)-4-methylbenzenesulfonamide (27e)**

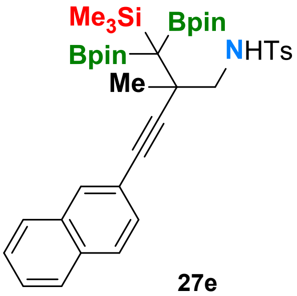

27e

The product was purified by flash chromatography using as eluent a mixture of pentane/diethyl ether (100:30). The product was isolated as a pale yellowish solid (63 mg, 45%).

^{1}H NMR (400 MHz, $CDCl_3$): δ 7.85 – 7.82 (m, 1H), 7.82 – 7.74 (m, 4H), 7.73 (s, 1H), 7.52 – 7.42 (m, 2H), 7.39 (dd, J = 8.4, 1.6 Hz, 1H), 7.26 (dd, J = 8.4, 0.8 Hz, 2H), 5.39 (dd, J = 8.8, 3.6 Hz, 1H), 3.53 (dd, J = 11.4, 8.8 Hz, 1H), 3.34 (dd, J = 11.4, 3.6 Hz, 1H), 2.39 (s, 3H), 1.52 (s, 3H), 1.19 (s, 12H), 1.15 (s, 6H), 1.13 (s, 6H), 0.24 (s, 9H).

^{13}C NMR {^{1}H} (125 MHz, $CDCl_3$): δ = 142.9, 137.4, 133.1, 132.6, 131.0, 129.5, 128.5, 127.8, 127.7, 127.4, 126.5, 126.4, 121.3, 96.7, 83.4, 83.0, 83.0, 52.7, 38.0, 26.8, 25.0, 25.0, 24.9, 24.9, 21.5, 2.7.

^{11}B NMR (128.3 MHz, $CDCl_3$): δ 32.7

HRMS (ESI) for **$C_{38}H_{54}B_2NO_6SSi$ [M+H]$^+$:** calculated: 702.3619, found: 702.3635.

4-methyl-*N*-(3-methyl-1-(naphthalen-2-yl)-5,5-bis(4,4,5,5-tetramethyl-1,3,2-dioxaborolan-2-yl)-5-(trimethylsilyl)pent-1-yn-3-yl)benzenesulfonamide (28e)

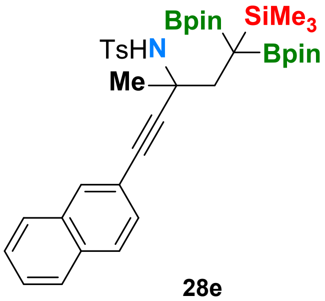

28e

The product was purified by flash chromatography using as eluent a mixture of pentane/diethyl ether (100:40). The product was isolated as a pale yellowish solid (52 mg, 40%).

^{1}H NMR (400 MHz, CDCl$_3$): δ δ 7.92 (d, J = 1.6 Hz, 1H), 7.83 (d, J = 8.2 Hz, 2H), 7.81 – 7.75 (m, 2H), 7.67 (d, J = 8.6 Hz, 1H), 7.47 (dd, J = 6.3, 3.3 Hz, 2H), 7.34 (dd, J = 8.6, 1.6 Hz, 1H), 7.23 (d, J = 8.2 Hz, 2H), 7.13 (s, 1H), 2.39 (s, 3H), 2.29 (d, J = 14.1 Hz, 1H), 2.15 (d, J = 14.1 Hz, 1H), 1.72 (s, 3H), 1.26 (s, 6H), 1.24 (s, 6H), 1.04 (s, 6H), 1.03 (s, 6H), 0.16 (s, 9H).

^{13}C NMR {^{1}H} (125 MHz, CDCl$_3$): δ = 142.7, 139.5, 132.8, 132.8, 132.8, 129.6, 129.4, 128.0, 127.9, 127.8, 127.2, 126.6, 126.4, 120.5, 93.0, 86.9, 83.5, 83.0, 56.4, 44.6, 28.6, 26.3, 26.1, 25.0, 24.5, 21.6, -1.1.

^{11}B NMR (128.3 MHz, CDCl$_3$): δ 33.1.

HRMS (ESI) for **$C_{38}H_{54}B_2NO_6SSi$ [M+H]$^+$:** calculated: 702.3619, found: 702.3622.

***N*-(2-(bis(4,4,5,5-tetramethyl-1,3,2-dioxaborolan-2-yl)methyl)-4-(4-(trifluoromethyl)phenyl)but-3-yn-1-yl)-4-methylbenzenesulfonamide (36a)**

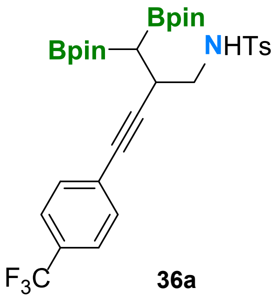

36a

The product was purified by flash chromatography using as eluent a mixture of pentane/diethyl ether (100:30). The product was isolated as a pale yellowish solid (88 mg, 69%).

^{1}H NMR (400 MHz, CDCl$_3$): δ 7.76 (d, J = 8.3 Hz, 2H), 7.52 (d, J = 8.5 Hz, 2H), 7.40 (d, J = 8.3 Hz, 2H), 7.26 (d, J = 8.5 Hz, 2H), 5.00 – 4.93 (m, 1H), 3.34 – 3.22 (m, 1H), 3.17 – 3.01 (m, 2H), 2.39 (s, 3H), 1.22 (s, 6H), 1.20 (s, 6H), 1.20 (s, 6H), 1.19 (s, 6H), 1.13 (d, J = 9.8 Hz, 1H).

^{13}C NMR {^{1}H} (125 MHz, CDCl$_3$): δ = 143.3, 137.3, 131.9, 131.9, 129.8, 129.7, 127.3, 127.2, 125.2 (q, J = 4.0 Hz), 94.5, 83.8, 83.7, 81.3, 47.9, 29.8, 24.9, 24.9, 24.5, 21.5.

^{11}B NMR (128.3 MHz, CDCl$_3$): δ 32.2.

^{19}F NMR {^{1}H} (376.5 MHz, CDCl$_3$): δ -62.78.

HRMS (ESI) for **$C_{31}H_{41}B_2F_3NO_6S^+$[M+H]$^+$:** calculated: 634.2798, found: 634.2799.

N-(2-(bis(4,4,5,5-tetramethyl-1,3,2-dioxaborolan-2-yl)methyl)-4-(4-(trifluoromethyl)phenyl)but-3-yn-1-yl)-4-methylbenzenesulfonamide (36b)

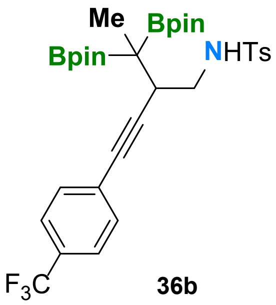

36b

The product was purified by flash chromatography using as eluent a mixture of pentane/diethyl ether (100:30). The product was isolated as a pale yellowish solid (94 mg, 72%).

^{1}H NMR (400 MHz, $CDCl_3$): δ 7.76 (d, J = 8.5 Hz, 2H), 7.52 (d, J = 8.0 Hz, 2H), 7.40 (d, J = 8.5 Hz, 2H), 7.26 (d, J = 8.0 Hz, 2H), 5.06 – 4.98 (m, 1H), 3.32 – 3.18 (m, 1H), 3.18 – 3.06 (m, 2H), 2.39 (s, 3H), 1.22 (s, 6H), 1.21 (s, 6H), 1.18 (s, 12H), 1.15 (s, 3H).

^{13}C NMR {^{1}H} (125 MHz, $CDCl_3$): δ = 143.4, 137.3, 132.1, 129.8, 127.4, 125.35 (q, J = 3.8 Hz), 93.4, 84.0, 83.8, 82.1, 45.1, 35.9, 25.0, 24.9, 24.8, 24.7, 21.7, 13.1.

^{11}B NMR (128.3 MHz, $CDCl_3$): δ 34.5.

^{19}F NMR {^{1}H} (376.5 MHz, $CDCl_3$): δ -62.75.

HRMS (ESI) for **$C_{32}H_{43}B_2F_3NO_6S^+$[M+H]$^+$:** calculated: 648.2955, found: 648.2956.

N-(2-(bis(4,4,5,5-tetramethyl-1,3,2-dioxaborolan-2-yl)(trimethylsilyl)methyl)-4-(4-(trifluoromethyl)phenyl)but-3-yn-1-yl)-4-methylbenzenesulfonamide (36e)

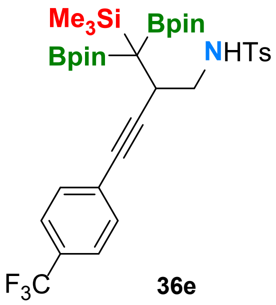

36e

The product was purified by flash chromatography using as eluent a mixture of pentane/diethyl ether (100:30). The product was isolated as a pale yellowish solid (92 mg, 65%).

^{1}H NMR (400 MHz, $CDCl_3$): δ7.79 (d, J = 8.4 Hz, 2H), 7.53 (d, J = 9.4 Hz, 2H), 7.37 (d, J = 8.4 Hz, 2H), 7.23 (d, J = 9.4 Hz, 2H), 5.47 (dd, J = 8.8, 3.4 Hz, 1H), 3.58 – 3.46 (m, 1H), 3.44 – 3.33 (m, 1H), 2.90 (dd, J = 8.8, 4.9 Hz, 1H), 2.37 (s, 3H), 1.23 (s, 6H), 1.19 (s, 12H), 1.16 (s, 6H), 0.10 (s, 9H).

^{13}C NMR {^{1}H} (125 MHz, $CDCl_3$): δ = 143.1, 138.1, 131.7, 129.5, 127.3, 125.29 (q, J = 4.0 Hz), 95.4, 83.3, 83.1, 81.9, 47.3, 31.9, 25.5, 25.3, 24.9, 24.4, 21.5, 0.0.

^{11}B NMR (128.3 MHz, $CDCl_3$): δ 34.3.

^{19}F NMR {^{1}H} (376.5 MHz, $CDCl_3$): δ -62.75.

HRMS (ESI) for **$C_{34}H_{49}B_2F_3NO_6SSi^+$[M+H]$^+$:** calculated: 706.3195, found: 706.3193.

N-(5,5-bis(4,4,5,5-tetramethyl-1,3,2-dioxaborolan-2-yl)-1-(4-(trifluoromethyl)phenyl)-5-(trimethylsilyl)pent-1-yn-3-yl)-4-methylbenzenesulfonamide (37e)

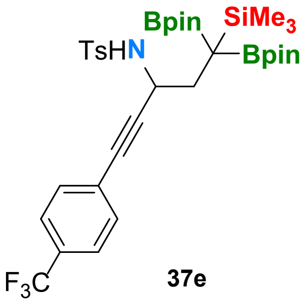

37e

The product was purified by flash chromatography using as eluent a mixture of pentane/diethyl ether (100:30). The product was isolated as a pale yellowish solid (10 mg, 7%).

^{1}H NMR (400 MHz, CDCl$_3$): δ = 7.75 (d, J = 8.3 Hz, 2H), 7.44 (d, J = 8.0 Hz, 2H), 7.12 (d, J = 8.3 Hz, 2H), 7.08 (d, *J* = 8.0 Hz, 2H), 6.20 (d, J = 8.0 H, 1H), 5.01 – 4.90 (m, 1H), 2.23 (s, 3H), 2.15 – 2.01 (m, 2H), 1.33 (s, 6H), 1.30 (s, 6H), 1.28 (s, 6H), 1.26 (s, 6H), 0.13 (s, 9H).

^{13}C NMR {^{1}H} (125 MHz, CDCl$_3$): δ = 142.6, 139.1, 131.8, 129.2, 127.5, 124.8 (q, J = 3.6 Hz), 92.1, 83.7, 83.2, 82.9, 45.9, 33.9, 25.8, 25.6, 24.8, 24.3, 21.4, -1.2

^{11}B NMR (128.3 MHz, CDCl$_3$): δ = 33.8

^{19}F NMR {^{1}H} (376.5 MHz, CDCl$_3$): δ -62.83.

HRMS (ESI) for **$C_{34}H_{49}B_2F_3NO_6SSi^+$[M+H]$^+$:** calculated: 706.3195, found: 706.3198.

S10. Characterization of substituted 2,3-dihydropyrroles

3-(bis(4,4,5,5-tetramethyl-1,3,2-dioxaborolan-2-yl)(trimethylsilyl)methyl)-3-methyl-5-phenyl-1-tosyl-2,3-dihydro-1*H*-pyrrole (29e)

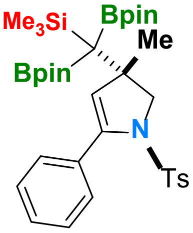

29e

The product was purified by flash chromatography using as eluent a mixture of pentane/diethyl ether (100:30). The product was isolated as a pale yellowish solid (59 mg, 91%).

^{1}H NMR (400 MHz, CDCl$_3$): δ 7.53 – 7.46 (m, 4H), 7.36 – 7.29 (m, 3H), 7.29 – 7.24 (m, 2H), 5.52 (s, 1H), 4.17 (d, J = 12.8 Hz, 1H), 3.83 (d, J = 12.8 Hz, 1H), 2.41 (s, 3H), 1.17 (s, 6H), 1.15 (s, 6H), 1.14 (s, 6H), 1.10 (s, 6H), 0.64 (s, 3H), 0.10 (s, 9H).

^{13}C NMR {^{1}H} (125 MHz, CDCl$_3$): δ = 143.2, 139.9, 134.6, 133.8, 129.3, 128.5, 128.2, 128.2, 127.5, 82.8, 82.7, 65.2, 47.6, 29.8, 25.1, 25.1, 25.0, 24.9, 21.6, 2.4.

^{11}B NMR (128.3 MHz, CDCl$_3$): δ 33.0.

HRMS (ESI) for **$C_{34}H_{52}B_2NO_6SSi$ [M+H]$^+$:** calculated: 652.3470, found: 652.3476.

3-(1,1-bis(4,4,5,5-tetramethyl-1,3,2-dioxaborolan-2-yl)ethyl)-3-methyl-5-phenyl-1-tosyl-2,3-dihydro-1H-pyrrole (29b)

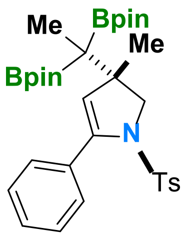

29b

The product was purified by flash chromatography using as eluent a mixture of pentane/diethyl ether (100:30). The product was isolated as a pale yellowish solid (56 mg, 63%).

^{1}H NMR (400 MHz, CDCl$_3$): δ 7.50 (dd, *J* = 7.8, 1.7 Hz, 4H), 7.36 – 7.29 (m, 3H), 7.24 (d, *J* = 1.7 Hz, 2H), 5.38 (s, 1H), 4.09 (d, *J* = 12.5 Hz, 1H), 3.66 (d, *J* = 12.3 Hz, 1H), 2.40 (s, 3H), 1.26 (s, 3H), 1.15 (s, 6H), 1.15 (s, 6H), 1.13 (s, 12H), 0.90 (s, 3H).

^{13}C NMR {^{1}H} (125 MHz, CDCl$_3$): δ = 143.3, 141.3, 134.2, 133.7, 129.3, 128.5, 128.3, 128.2, 127.5, 125.5, 83.1, 83.0, 62.5, 47.7, 29.8, 25.6, 24.8, 24.7, 24.7, 24.6, 21.6, 14.1.

^{11}B NMR (128.3 MHz, CDCl$_3$): δ 34.5.

HRMS (ESI) for **$C_{32}H_{46}B_2NO_6S^+$[M+H$^+$]$^+$:** calculated: 594.3226, found: 594.3242.

3-(bis(4,4,5,5-tetramethyl-1,3,2-dioxaborolan-2-yl)(trimethylsilyl)methyl)-3-methyl-5-(naphthalen-2-yl)-1-tosyl-2,3-dihydro-1*H*-pyrrole (31e)

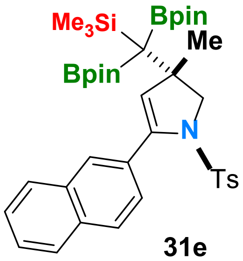

31e

The product was purified by flash chromatography using as eluent a mixture of pentane/diethyl ether (100:30). The product was isolated as a pale yellowish solid (59 mg, 85%).

^{1}H NMR (400 MHz, CDCl$_3$): δ 7.90 (s, 1H), 7.88 – 7.81 (m, 2H), 7.79 (d, J = 8.5 Hz, 1H), 7.69 (dd, J = 8.6, 1.7 Hz, 1H), 7.56 – 7.51 (m, 2H), 7.51 – 7.45 (m, 2H), 7.25 (d, J = 8.5 Hz, 2H), 5.67 (s, 1H), 4.25 (d, J = 13.6 Hz, 1H), 3.88 (d, J = 13.6 Hz, 1H), 2.40 (s, 3H), 1.18 (s, 6H), 1.16 (s, 6H), 1.14 (s, 6H), 1.11 (s, 6H), 0.69 (s, 3H), 0.12 (s, 9H)

^{13}C NMR {^{1}H} (125 MHz, CDCl$_3$): δ = 143.3, 139.9, 134.6, 133.4, 133.0, 131.6, 129.4, 128.2, 128.2, 127.8, 126.9, 126.7, 126.1, 126.0, 82.8, 82.7, 65.2, 47.8, 29.9, 29.5, 25.1, 25.1, 24.9, 24.9, 21.6, 2.4.

^{11}B NMR (128.3 MHz, CDCl$_3$): δ 32.0.

HRMS (ESI) for **$C_{38}H_{54}B_2NO_6SSi^+$[M+H]$^+$:** calculated: 702.3635, found: 702.3635.

S11. Characterization of (*Z*)-2-alkylidene-1-tosylazetidines

(*Z*)-2-benzylidene-3-(1,1-bis(4,4,5,5-tetramethyl-1,3,2-dioxaborolan-2-yl)ethyl)-1-tosylazetidine (30b)

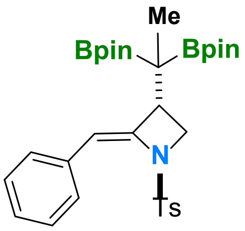

30b

The product was purified by flash chromatography using as eluent a mixture of pentane/diethyl ether (100:30). The product was isolated as a pale yellowish solid (65 mg, 74%).

^{1}H NMR (400 MHz, $CDCl_3$): δ 7.50 – 7.46 (m, 4H), 7.35 – 7.30 (m, 3H), 7.24 (d, *J* = 8.1 Hz, 2H), 5.13 (d, *J* = 2.3 Hz, 1H), 4.09 (dd, *J* = 12.1, 10.0 Hz, 1H), 3.62 (dd, *J* = 12.2, 8.8 Hz, 1H), 3.02 (ddd, *J* = 10.1, 8.7, 2.3 Hz, 1H), 2.41 (s, 3H), 1.18 (s, 12H), 1.16 (s, 6H), 1.15 (s, 6H), 0.75 (s, 3H).

^{13}C NMR {^{1}H} (125 MHz, $CDCl_3$): δ = 144.1, 143.4, 133.8, 133.7, 133.3, 129.4, 128.4, 128.3, 127.6, 121.1, 83.4, 83.3, 55.0, 44.0, 24.8, 24.8, 24.6, 24.6, 21.6, 12.3.

^{11}B NMR (128.3 MHz, $CDCl_3$): δ 33.9.

HRMS (ESI) for **$C_{31}H_{44}B_2NO_6S^+$[M+H$^+$]$^+$:** calculated: 580.3070 , found: 580.3072.

(*Z*)-2-benzylidene-3-(bis(4,4,5,5-tetramethyl-1,3,2-dioxaborolan-2-yl)(trimethylsilyl)methyl)-1-tosylazetidine (30e)

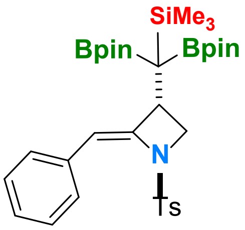

30e

The product was purified by flash chromatography using as eluent a mixture of pentane/diethyl ether (100:30). The product was isolated as a pale yellowish solid (92 mg, 72%).

^{1}H NMR (400 MHz, $CDCl_3$): δ 7.59 (d, *J* = 8.3 Hz, 2H), 7.51 (dd, *J* = 8.0, 1.6 Hz, 2H), 7.36 – 7.28 (m, 3H), 7.27 (d, *J* = 0.8 Hz, 1H), 7.25 (s, 1H), 5.38 (d, *J* = 2.0 Hz, 1H), 4.23 (dd, *J* = 12.1, 8.3 Hz, 1H), 3.86 (dd, *J* = 12.1, 11.3 Hz, 1H), 2.62 – 2.45 (m, 1H), 2.42 (s, 3H), 1.16 (s, 6H), 1.14 (s, 6H), 1.09 (s, 6H), 1.05 (s, 6H), -0.03 (s, 9H).

^{13}C NMR {^{1}H} (125 MHz, $CDCl_3$): δ = 143.5, 142.9, 134.3, 134.1, 129.2, 128.2, 128.1, 127.8, 127.5, 125.9, 82.8, 82.6, 59.3, 40.7, 25.3, 25.3, 25.1, 25.0, 24.8, 24.6, 21.6, 0.0.

^{11}B NMR (128.3 MHz, $CDCl_3$): δ 32.5.

HRMS (ESI) for **$C_{33}H_{50}B_2NO_6SSi^+$[M+H$^+$]$^+$:** calculated: 638.3309, found: 638.3323.

(*Z*)-3-(1,1-bis(4,4,5,5-tetramethyl-1,3,2-dioxaborolan-2-yl)ethyl)-2-(4-chlorobenzylidene)-1-tosylazetidine (32b)

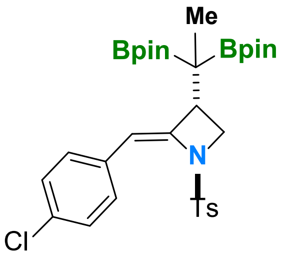

32b

The product was purified by flash chromatography using as eluent a mixture of pentane/diethyl ether (100:30). The product was isolated as a pale yellowish solid (68 mg, 75%).

^{1}H NMR (400 MHz, CDCl$_3$): δ 7.48 – 7.46 (m, 2H), 7.43 – 7.40 (m, 2H), 7.29 (d, *J* = 8.1 Hz, 2H), 7.25 (d, *J* = 8.1 Hz, 2H), 5.15 (d, *J* = 2.3 Hz, 1H), 4.09 (dd, *J* = 12.1, 10.0 Hz, 1H), 3.62 (dd, *J* = 12.2, 8.8 Hz, 1H), 2.96 (ddd, *J* = 10.1, 8.7, 2.3 Hz, 1H), 2.42 (s, 3H), 1.17 (s, 12H), 1.16 (s, 6H), 1.15 (s, 6H), 0.75 (s, 3H).

^{13}C NMR {^{1}H} (125 MHz, CDCl$_3$): δ = 143.7, 143.0, 134.1, 133.6, 129.5, 129.4, 128.2, 128.0, 127.1, 121.8, 83.4, 83.3, 55.1, 44.1, 24.8, 24.8, 24.6, 24.6, 21.7, 12.5.

^{11}B NMR (128.3 MHz, CDCl$_3$): δ 34.3.

HRMS (ESI) for **$C_{31}H_{43}B_2ClNO_6S^+$[M+H$^+$]$^+$:** calculated: 614.2680 , found: 614.2686.

(*Z*)-3-(bis(4,4,5,5-tetramethyl-1,3,2-dioxaborolan-2-yl)(trimethylsilyl)methyl)-2-(4-chlorobenzylidene)-1-tosylazetidine (32e)

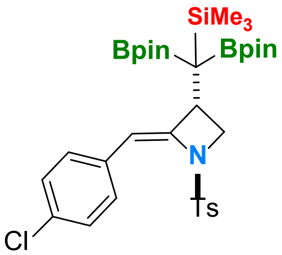

32e

The product was purified by flash chromatography using as eluent a mixture of pentane/diethyl ether (100:30). The product was isolated as a pale yellowish solid (63 mg, 93%).

^{1}H NMR (400 MHz, CDCl$_3$): δ 7.58 (d, J = 8.4 Hz, 2H), 7.45 (d, J = 8.5 Hz, 2H), 7.29 (d, J = 8.6 Hz, 2H), 7.28 – 7.24 (m, 2H), 5.39 (d, J = 2.0 Hz, 1H), 4.21 (dd, J = 12.2, 8.1 Hz, 1H), 3.88 – 3.78 (dd, J = 11.0, 8.1 Hz, 1H), 2.49 (ddd, J = 11.3, 8.1, 2.0 Hz, 1H), 2.41 (s, 3H), 1.15 (s, 6H), 1.13 (s, 6H), 1.08 (s, 6H), 1.04 (s, 6H), -0.04 (s, 9H).

^{13}C NMR {^{1}H} (125 MHz, CDCl$_3$): δ = 143.7, 141.8, 134.1, 133.8, 132.6, 129.3, 128.6, 128.1, 128.1, 126.7, 82.8, 82.6, 59.3, 40.8, 25.1, 25.0, 24.8, 24.5, 21.6, 0.1.

^{11}B NMR (128.3 MHz, CDCl$_3$): δ 32.4.

HRMS (ESI) for **$C_{33}H_{49}B_2ClNO_6SSi$ [M+H]$^+$:** calculated: 672.2918, found: 672.2931.

(*Z*)-3-(1,1-bis(4,4,5,5-tetramethyl-1,3,2-dioxaborolan-2-yl)ethyl)-2-(naphthalen-2-ylmethylene)-1-tosylazetidine (33b)

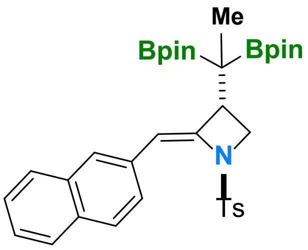

33b

The product was purified by flash chromatography using as eluent a mixture of pentane/diethyl ether (100:30). The product was isolated as a pale yellowish solid (62 mg, 65%).

^{1}H NMR (400 MHz, $CDCl_3$): δ 7.66 – 7.54 (m, 5H), 7.43 (dd, *J* = 8.6, 1.8 Hz, 1H), 7.29 – 7.27 (m, 1H), 7.25 – 7.22 (m, 2H), 7.03 – 6.98 (m, 2H), 5.03 (d, *J* = 2.3 Hz, 1H), 3.93 (dd, *J* = 12.2, 10.0 Hz, 1H), 3.45 (dd, *J* = 12.2, 8.8 Hz, 1H), 2.87 – 2.72 (m, 2H), 2.18 (s, 3H), 0.96 (s,12H), 0.94 (s, 6H), 0.93 (s, 6H), 0.56 (s, 3H).

^{13}C NMR {^{1}H} (125 MHz, $CDCl_3$): δ = 144.1, 143.6, 133.7, 133.4, 133.0, 131.2, 129.4, 128.3, 128.2, 127.8, 127.0, 126.7, 126.6, 126.2, 126.1, 121.8, 83.4, 83.3, 55.1, 44.2, 24.8, 24.8, 24.6, 24.6, 21.6, 12.4.

^{11}B NMR (128.3 MHz, $CDCl_3$): δ 33.7.

HRMS (ESI) for **$C_{35}H_{46}B_2NO_6S^+$[M+H]$^+$:** calculated: 630.3238, found: 630.3236.

(*Z*)-3-(bis(4,4,5,5-tetramethyl-1,3,2-dioxaborolan-2-yl)(trimethylsilyl)methyl)-2-(naphthalen-2-ylmethylene)-1-tosylazetidine (33e)

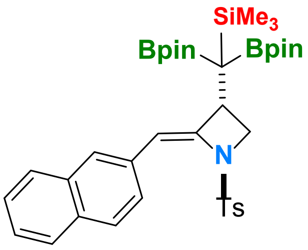

33e

The product was purified by flash chromatography using as eluent a mixture of pentane/diethyl ether (100:30). The product was isolated as a pale yellowish solid (52 mg, 76%).

^{1}H NMR (400 MHz, $CDCl_3$): δ 7.93 (d, J = 1.9 Hz, 1H), 7.90 – 7.74 (m, 3H), 7.68 (dd, J = 8.4, 1.9 Hz, 1H), 7.62 (d, J = 8.4 Hz, 2H), 7.53 – 7.38 (m, 2H), 7.27 (d, J = 7.6 Hz, 2H), 5.52 (d, J = 2.1 Hz, 1H), 4.29 (dd, J = 12.1, 8.3 Hz, 1H), 3.91 (dd, J = 12.1, 10.3 Hz,, 1H), 2.59 (ddd, J = 10.6, 8.3, 2.1 Hz, 1H), 2.43 (s, 3H), 1.17 (s, 6H), 1.15 (s, 6H), 1.09 (s, 6H), 1.05 (s, 6H), -0.01 (s, 9H).

^{13}C NMR {^{1}H} (125 MHz, $CDCl_3$): δ = 143.3, 142.7, 134.2, 133.2, 132.9, 131.5, 129.1, 128.1, 128.0, 127.6, 127.0, 126.5, 125.9, 125.8, 125.7, 82.6, 82.4, 59.2, 40.7, 24.9, 24.8, 24.7, 24.6, 24.4, 21.4, -0.0.

^{11}B NMR (128.3 MHz, $CDCl_3$): δ 34.1.

HRMS (ESI) for **$C_{37}H_{52}B_2NO_6SSi$ [M+H]$^+$:** calculated: 688.3474, found: 688.3478.

(*Z*)-3-(bis(4,4,5,5-tetramethyl-1,3,2-dioxaborolan-2-yl)(trimethylsilyl)methyl)-2-(cyclohexylmethylene)-1-tosylazetidine (34e)

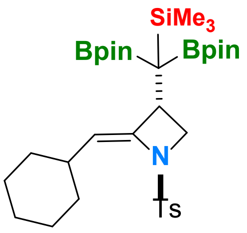

34e

The product was purified by flash chromatography using as eluent a mixture of pentane/diethyl ether (100:30). The product was isolated as a pale yellowish solid (27 mg, 41%).

^{1}H NMR (400 MHz, CDCl$_3$): δ 7.74 – 7.67 (m, 2H), 7.26 – 7.20 (m, 2H), 3.88 (dd, J = 12.0, 7.6 Hz, 1H), 3.30 (dd, J = 12.0, 11.5 Hz, 1H), 2.76 – 2.65 (m, 1H), 2.46 – 2.36 (m, 1H), 2.39 (s, 3H), 2.15 – 1.96 (m, 3H), 1.82 – 1.69 (m, 1H), 1.63 – 1.38 (m, 7H), 1.20 (s, 6H), 1.15 (s, 6H), 1.12 (s, 6H), 1.10 (s, 6H), -0.13 (s, 9H).

^{13}C NMR {^{1}H} (125 MHz, CDCl$_3$): δ = 143.3, 137.0, 132.8, 130.2, 129.5, 127.9, 82.7, 82.4, 55.9, 34.9, 34.5, 31.9, 31.6, 27.8, 27.7, 26.7, 25.3, 25.1, 24.8, 24.6, 21.6, 0.0.

^{11}B NMR (128.3 MHz, CDCl$_3$): δ 33.9

HRMS (ESI) for **$C_{33}H_{56}B_2NO_6SSi$ [M+H]$^+$:** calculated: 644.3804, found: 644.3790.

(*Z*)-3-(bis(4,4,5,5-tetramethyl-1,3,2-dioxaborolan-2-yl)(trimethylsilyl)methyl)-2-heptylidene-1-tosylazetidine (35e)

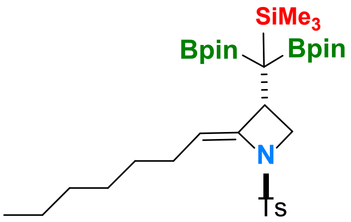

35e

The product was purified by flash chromatography using as eluent a mixture of pentane/diethyl ether (100:30). The product was isolated as a pale yellowish solid (69 mg, 59%).

^{1}H NMR (400 MHz, CDCl$_3$): δ 7.73 (d, J = 8.3 Hz, 2H), 7.24 (d, J = 8.3 Hz, 2H), 5.51 (dd, J = 2.8, 1.4 Hz, 1H), 3.93 (dd, J = 9.1, 6.5 Hz, 1H), 3.53 (dd, J = 11.0, 9.1 Hz, 1H), 2.49 (dd, J = 11.0, 6.5 Hz, 1H), 2.40 (s, 3H), 2.29 – 2.10 (m, 2H), 2.02 – 1.78 (m, 2H), 1.34 – 1.19 (m, 6H), 1.18 (s, 6H), 1.16 (s, 6H), 1.15 (s, 6H), 1.12 (s, 6H), 0.84 (t, J = 7.3 Hz, 3H), 0.06 (s, 9H).

^{13}C NMR {^{1}H} (125 MHz, CDCl$_3$): δ = 143.1, 138.4, 135.7, 129.2, 127.8, 125.6, 108.1, 82.7, 82.6, 56.1, 35.5, 35.0, 31.3, 30.4, 29.8, 28.8, 25.2, 25.1, 24.7, 24.6, 22.7, 21.6, 14.2, 0.2.

^{11}B NMR (128.3 MHz, CDCl$_3$): δ 32.7

HRMS (ESI) for **$C_{33}H_{58}B_2NO_6SSi^+$[M+H]$^+$:** calculated: 646.3947, found: 646.3947.

(*Z*)-3-(bis(4,4,5,5-tetramethyl-1,3,2-dioxaborolan-2-yl)methyl)-1-tosyl-2-(4-(trifluoromethyl)benzylidene)azetidine (38a)

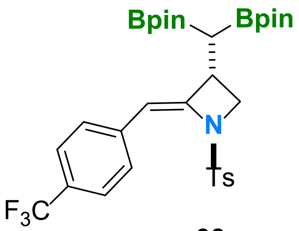

38a

The product was purified by flash chromatography using as eluent a mixture of pentane/diethyl ether (100:30). The product was isolated as a pale yellowish solid (36 mg, 57%).

^{1}H NMR (400 MHz, touene-d^8): δ 7.59 – 7.51 (m, 4H), 7.35 (d, J = 8.5 Hz, 2H), 6.77 (d, J = 8.1 Hz, 2H), 5.57 (d, J = 2.5 Hz, 1H), 4.46 (dd, J = 12.7, 9.2 Hz, 1H), 3.79 (dd, J = 12.7, 7.8 Hz, 1H), 3.05 –

2.93 (m, 1H), 1.90 (s, 3H), 1.02 (s, 6H), 1.01 (s, 6H), 0.97 (s, 6H), 0.96 (s, 6H), 0.40 (d, J = 10.3 Hz, 1H).

^{13}C NMR {^{1}H} (125 MHz, touene-d^{8}): δ =143.0, 142.6, 134.6, 129.1, 128.8, 128.0, 125.7, 82.8, 82.7, 59.3, 38.7, 30.0, 24.5, 24.4, 24.0, 20.7.

^{11}B NMR (128.3 MHz, touene-d^{8}): δ 33.4

^{19}F NMR {^{1}H} (376.5 MHz, touene-d^{8}): δ -62.2.

HRMS (ESI) for **$C_{31}H_{41}B_2F_3NO_6S^+$[M+H]$^+$:** calculated: 634.2798, found: 634.2800.

(*Z*)-3-(1,1-bis(4,4,5,5-tetramethyl-1,3,2-dioxaborolan-2-yl)ethyl)-1-tosyl-2-(4-(trifluoromethyl)benzylidene)azetidine (38b)

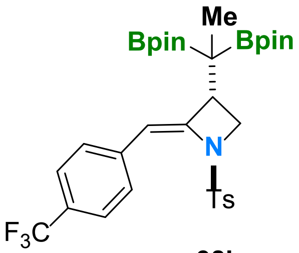

38b

The product was purified by flash chromatography using as eluent a mixture of pentane/diethyl ether (100:30). The product was isolated as a pale yellowish solid (37 mg, 53%).

^{1}H NMR (400 MHz, touene-d^{8}): δ 7.57 – 7.50 (m, 4H), 7.36 (d, J = 8.3 Hz, 2H), 6.72 (d, J = 8.1 Hz, 2H), 5.50 (d, J = 2.4 Hz, 1H), 4.48 (dd, J = 12.3, 9.5 Hz, 1H), 4.01 (dd, J = 12.3, 9.5 Hz, 1H), 3.17 (t, J = 9.5, 2.4 Hz, 1H), 1.88 (s, 3H), 1.06 (s, 3H), 1.00 (s, 6H), 0.99 (s, 6H), 0.98 (s, 6H), 0.97 (s, 6H).

^{13}C NMR {^{1}H} (125 MHz, touene-d^{8}): δ =143., 142.8, 134.7, 129.0, 128.8, 128.3, 128.1, 124.4, 123.1, 82.9, 82.8, 55.5, 44.7, 30.0, 24.3, 24.2, 24.1, 24.1, 20.6, 13.3.

^{11}B NMR (128.3 MHz, touene-d^{8}): δ 33.4

^{19}F NMR {^{1}H} (376.5 MHz, touene-d^{8}): δ -62.2.

HRMS (ESI) for **$C_{32}H_{43}B_2F_3NO_6S^+$[M+H]$^+$:** calculated: 648.2955, found: 648.2957.

S12. Characterization of 39b and 40b

(*Z*)-3-(1-(4,4,5,5-tetramethyl-1,3,2-dioxaborolan-2-yl)ethyl)-1-tosyl-2-(4-(trifluoromethyl)benzylidene)azetidine (39b)

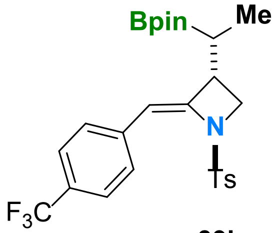

39b

The product was purified by flash chromatography using as eluent a mixture of pentane/diethyl ether (100:30). The product was isolated as a pale yellowish solid in a dr: 1/1 (34 mg, 73%).

^{1}H NMR (400 MHz, CDCl$_3$): δ 7.67 – 7.55 (m, 8H), 7.54 – 7.48 (m, 4H), 7.30 – 7.26 (m, 4H), 5.53 (d, J = 2.4 Hz, 1H), 5.47 (d, J = 2.5 Hz, 1H), 4.23 – 4.05 (m, 2H), 3.72 – 3.56 (m, 2H), 2.56 – 2.43 (m, 2H), 2.43 (s, 6H), 1.20 (s, 6H), 1.19 (s, 6H), 1.18 (s, 6H), 1.17 (s, 6H), 0.83 – 0.77 (m, 6H), 0.73 – 0.59 (m, 2H).

^{13}C NMR {^{1}H} (125 MHz, CDCl$_3$): δ = 144.1, 144.0, 143.1, 143.0, 136.9, 136.9, 133.6, 133.6, 129.6, 128.1, 128.1, 128.1, 128.1, 125.6, 124.9 (q, J = 3.8 Hz), 124.3, 123.7, 122.9, 83.4, 83.4, 56.9, 56.3, 43.9, 43.8, 30.4, 29.8, 24.9, 24.8, 24.8, 21.7, 21.7, 12.9, 12.8.

^{11}B NMR (128.3 MHz, CDCl$_3$): δ 33.8

^{19}F NMR {^{1}H} (376.5 MHz, CDCl$_3$): δ -62.58, -62.57.

HRMS (ESI) for **$C_{26}H_{32}BF_3NO_4S^+$[M+H]$^+$:** calculated: 522.2096, found: 522.2094.

(*Z*)-1-(1-tosyl-2-(4-(trifluoromethyl)benzylidene)azetidin-3-yl)ethan-1-ol (40b)

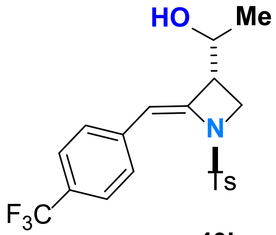

40b

The product was purified by flash chromatography using as eluent a mixture of pentane/diethyl ether (100:30). The product was isolated as a pale yellowish oil in a dr: 1/1 (21 mg, 73%).

^{1}H NMR (400 MHz, CDCl$_3$): δ 7.73 – 7.55 (m, 8H), 7.56 – 7.47 (m, 4H), 7.32 – 7.24 (m, 4H), 5.58 (d, J = 2.5 Hz, 1H), 5.31 (d, J = 2.5 Hz, 1H), 4.16 – 4.02 (m, 2H), 3.94 (dd, J = 12.8, 8.7 Hz, 1H), 3.66 (dd, J = 12.8, 8.7 Hz, 1H), 3.38 – 3.27 (m, 1H), 3.31 – 3.20 (m, 1H), 2.57 – 2.46 (m, 2H), 2.44 (s, 6H), 1.25 (s, 2H), 1.08 (d, J = 2.1 Hz, 3H), 1.06 (d, J = 2.1 Hz, 3H).

^{13}C NMR {^{1}H} (125 MHz, CDCl$_3$): δ = 144.4, 144.3, 133.5, 133.3, 129.7, 129.6, 128.3, 128.2, 128.1, 128.1, 124.9, 120.1, 119.4, 69.9, 69.7, 53.7, 53.6, 49.3, 49.1, 21.7, 21.7, 21.6, 21.4.

^{11}B NMR (128.3 MHz, CDCl$_3$): δ 33.8

^{19}F NMR {^{1}H} (376.5 MHz, CDCl$_3$): δ -62.79, -62.80.

S13. NMR spectra of propargylic aziridines

2-Methyl-2-(naphthalen-2-ylethynyl)-1-tosylaziridine

^{1}H NMR (500 MHz, $CDCl_3$)

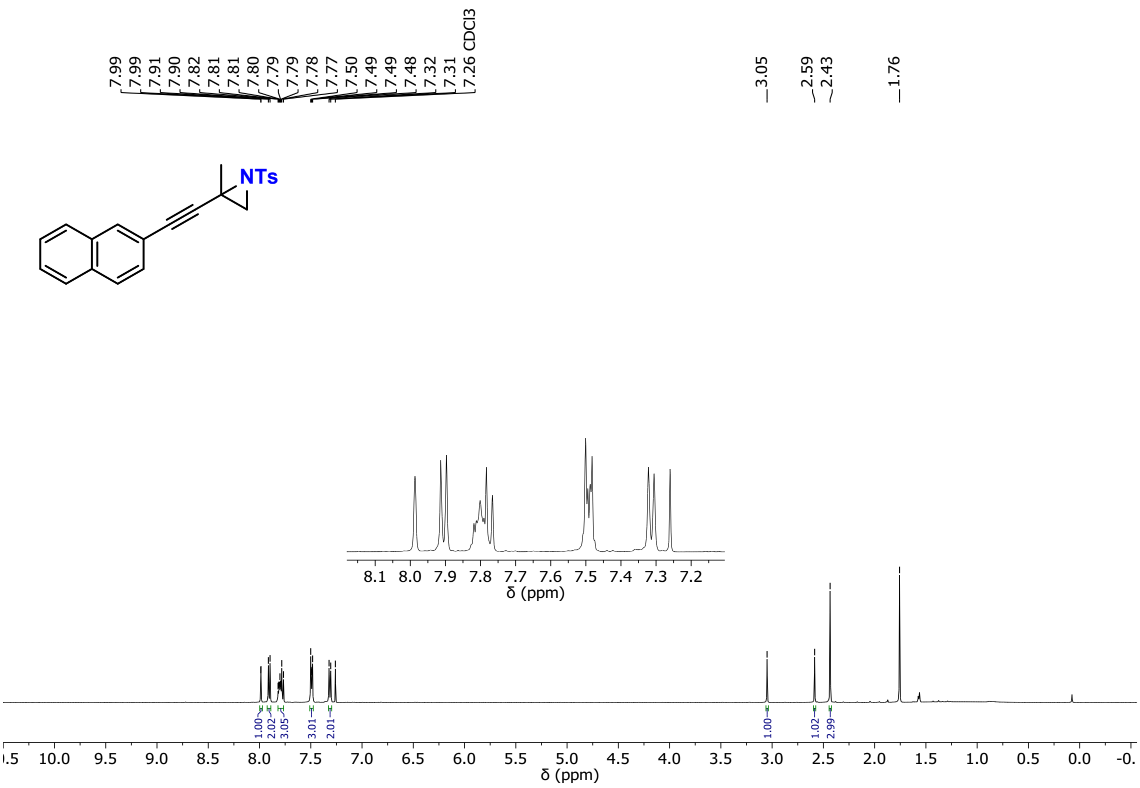

^{13}C NMR {^{1}H} (125 MHz, $CDCl_3$)

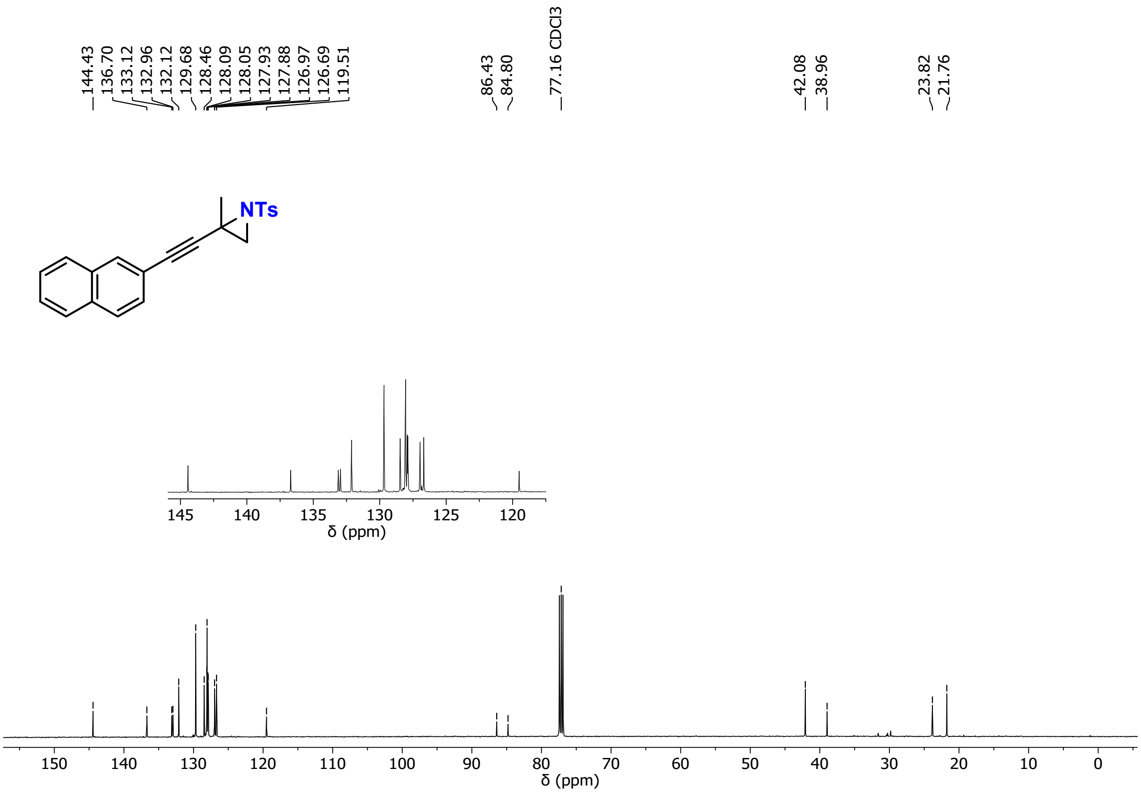

2-((4-chlorophenyl)ethynyl)-1-tosylaziridine

^{1}H NMR (500 MHz, $CDCl_3$)

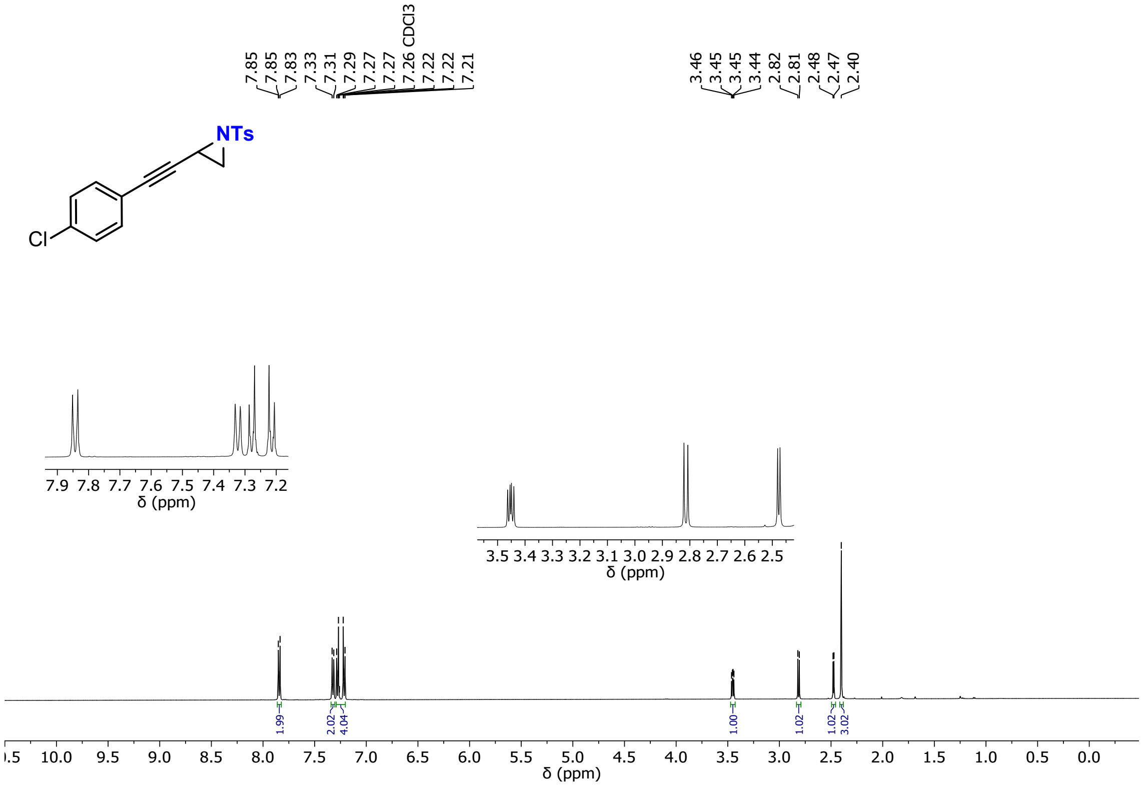

^{13}C NMR {[1]H} (125 MHz, $CDCl_3$)

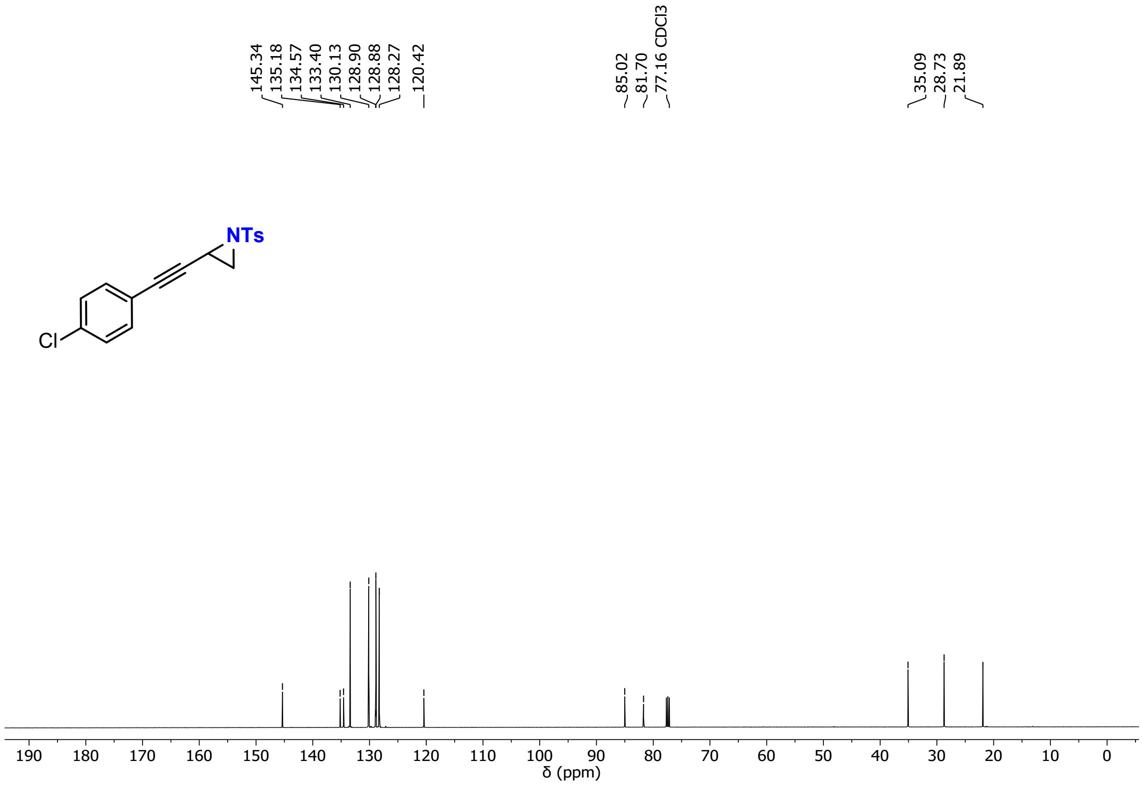

1-tosyl-2-((4-(trifluoromethyl)phenyl)ethynyl)aziridine

^{1}H NMR (500 MHz, $CDCl_3$)

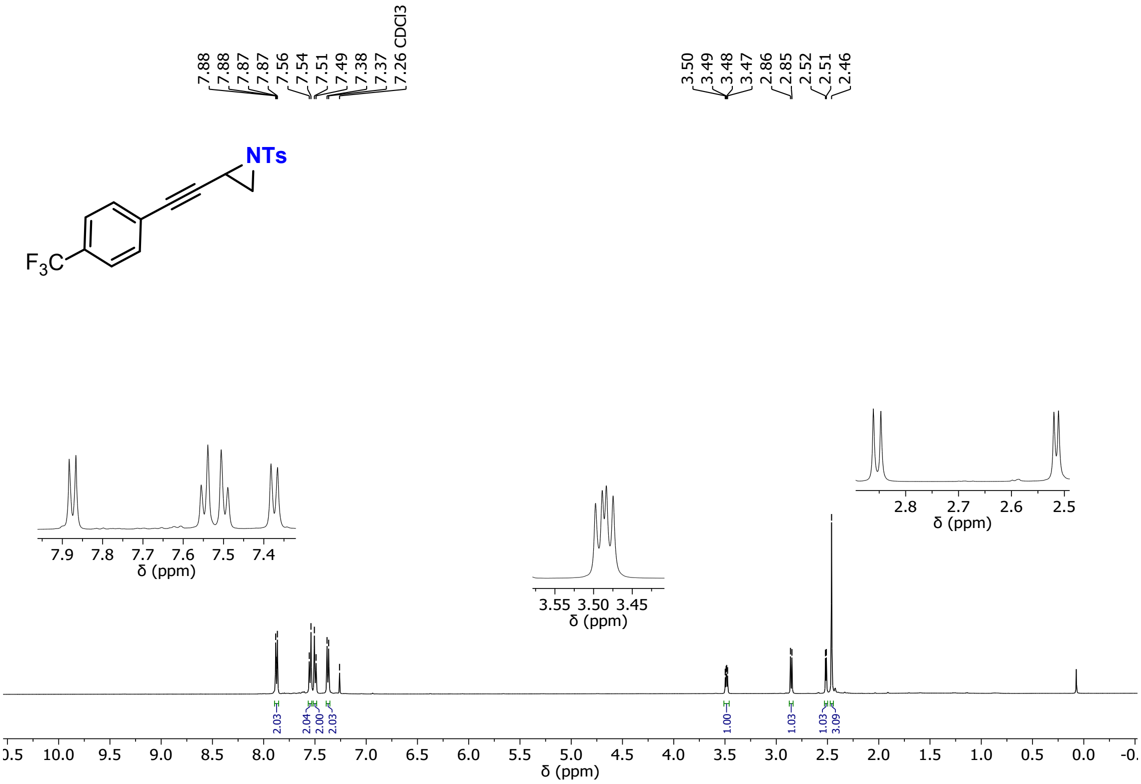

^{13}C NMR {^{1}H} (125 MHz, $CDCl_3$)

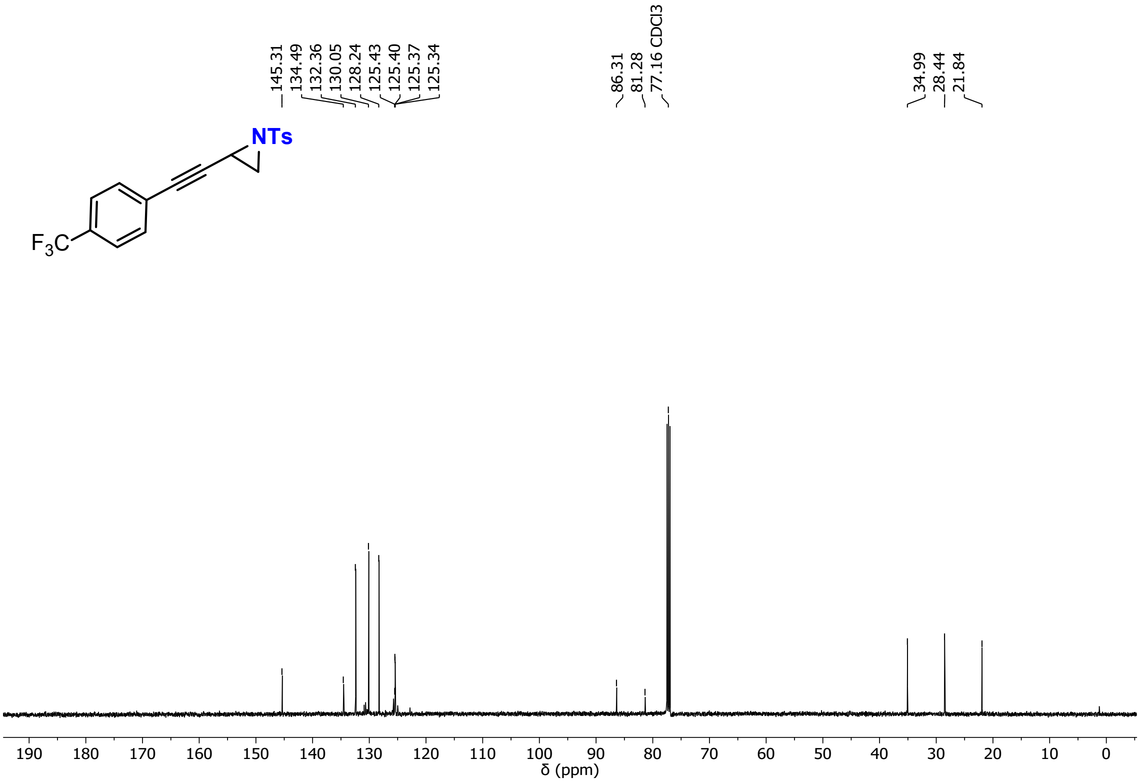

^{19}F NMR {^{1}H} (470 MHz, $CDCl_3$)

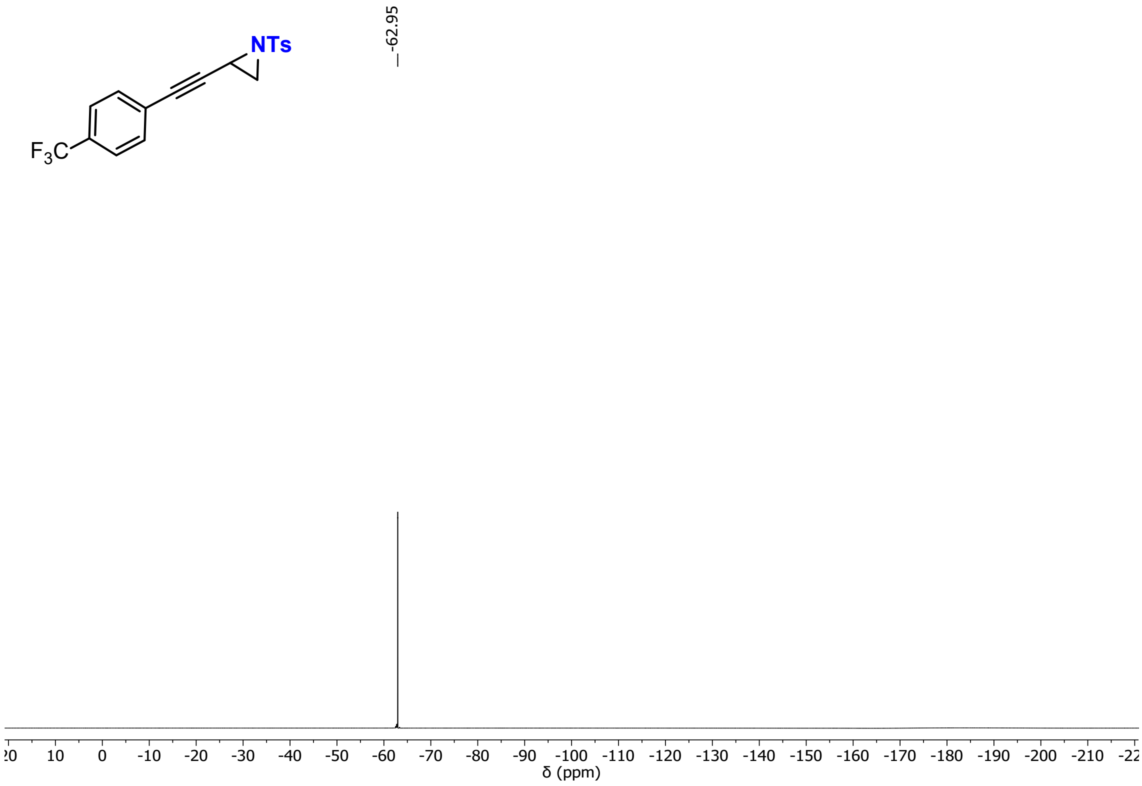

S14.NMR spectra of *N*-tosyl homopropargyl amines and *N*-tosyl propargyl amines.

N-(2-(bis(4,4,5,5-tetramethyl-1,3,2-dioxaborolan-2-yl)methyl)-4-phenylbut-3-yn-1-yl)-4-methylbenzenesulfonamide (3a)

^{1}H NMR (400 MHz, $CDCl_3$)

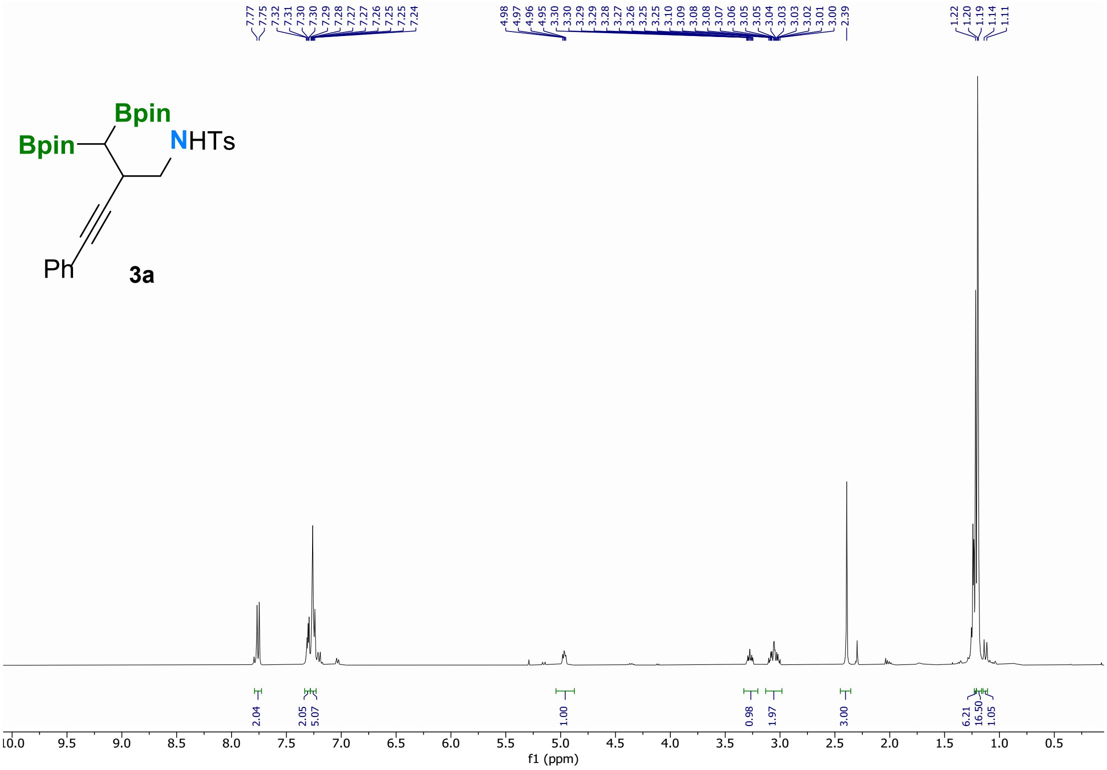

^{13}C NMR {^{1}H} (125 MHz, $CDCl_3$)

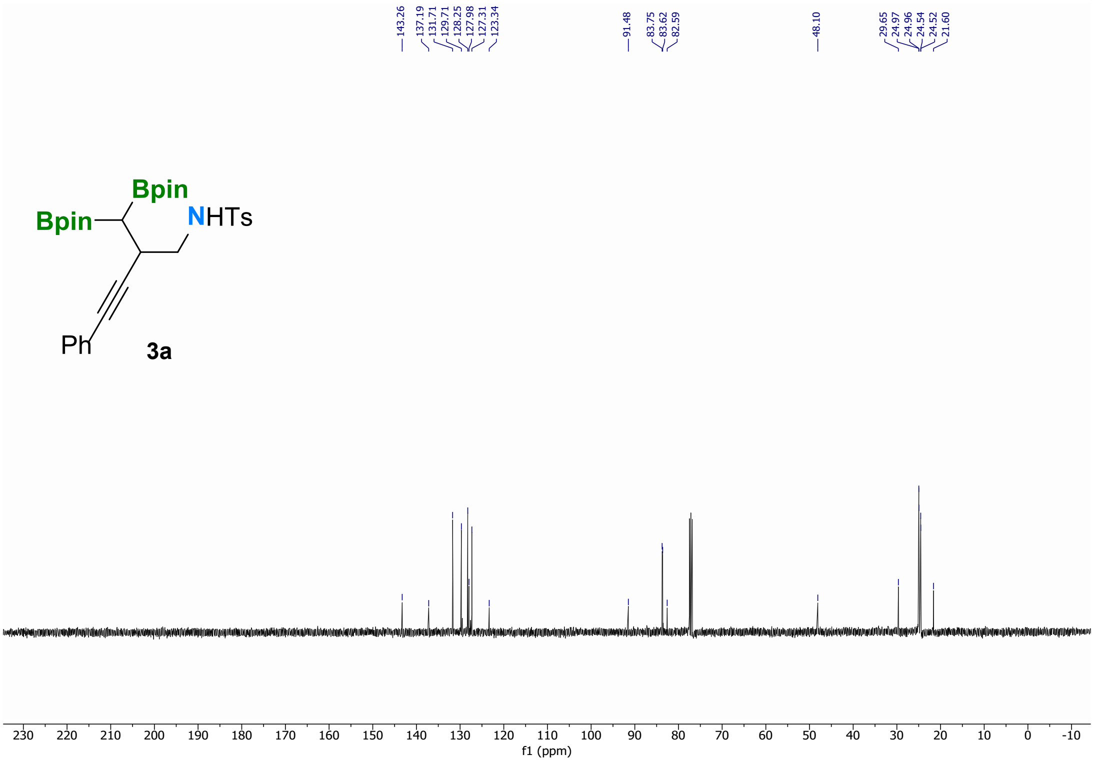

^{11}B NMR (128.3 MHz, $CDCl_3$)

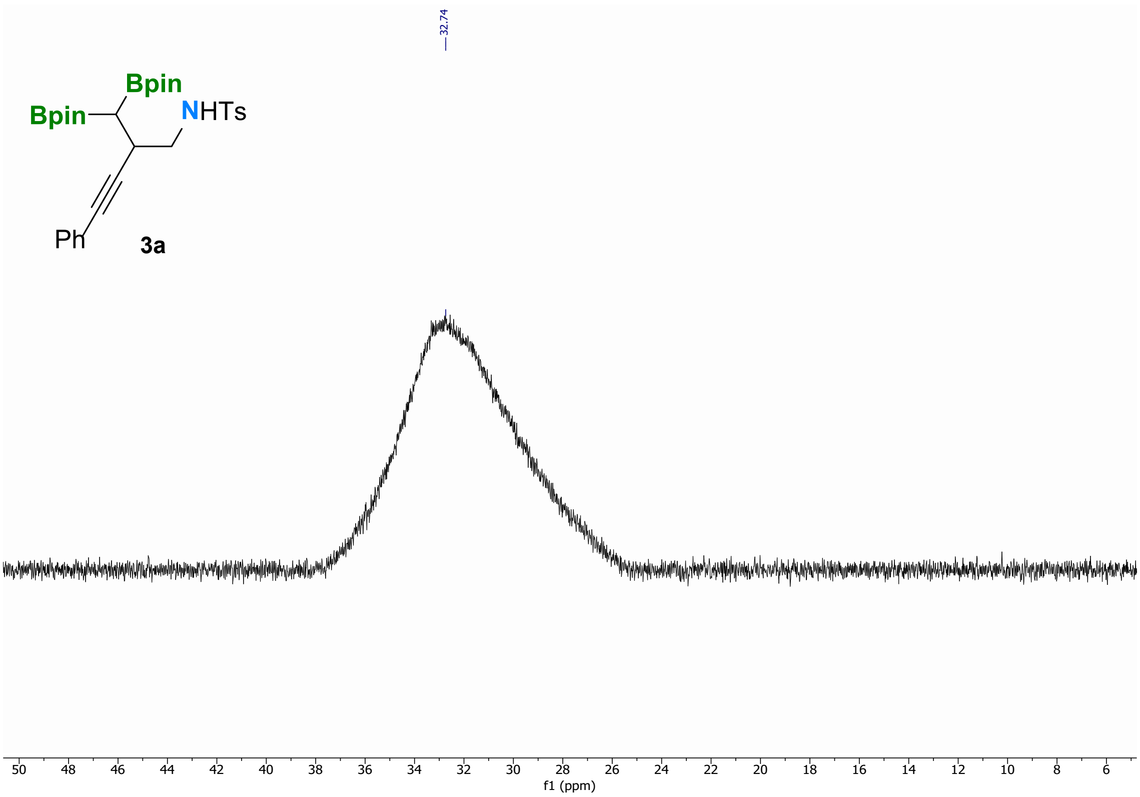

***N*-(2-(1,1-bis(4,4,5,5-tetramethyl-1,3,2-dioxaborolan-2-yl)ethyl)-4-phenylbut-3-yn-1-yl)-4-methylbenzenesulfonamide (3b)**

^{1}H NMR (400 MHz, $CDCl_3$)

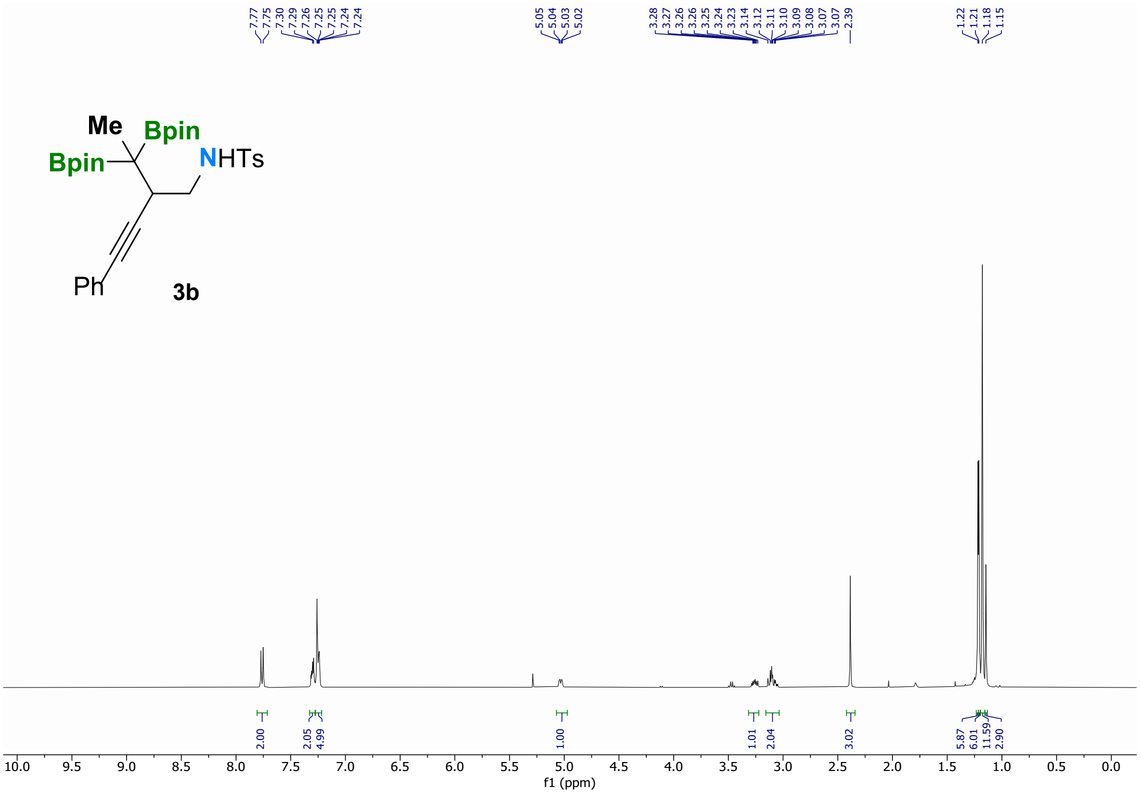

^{13}C NMR {^{1}H} (125 MHz, $CDCl_3$)

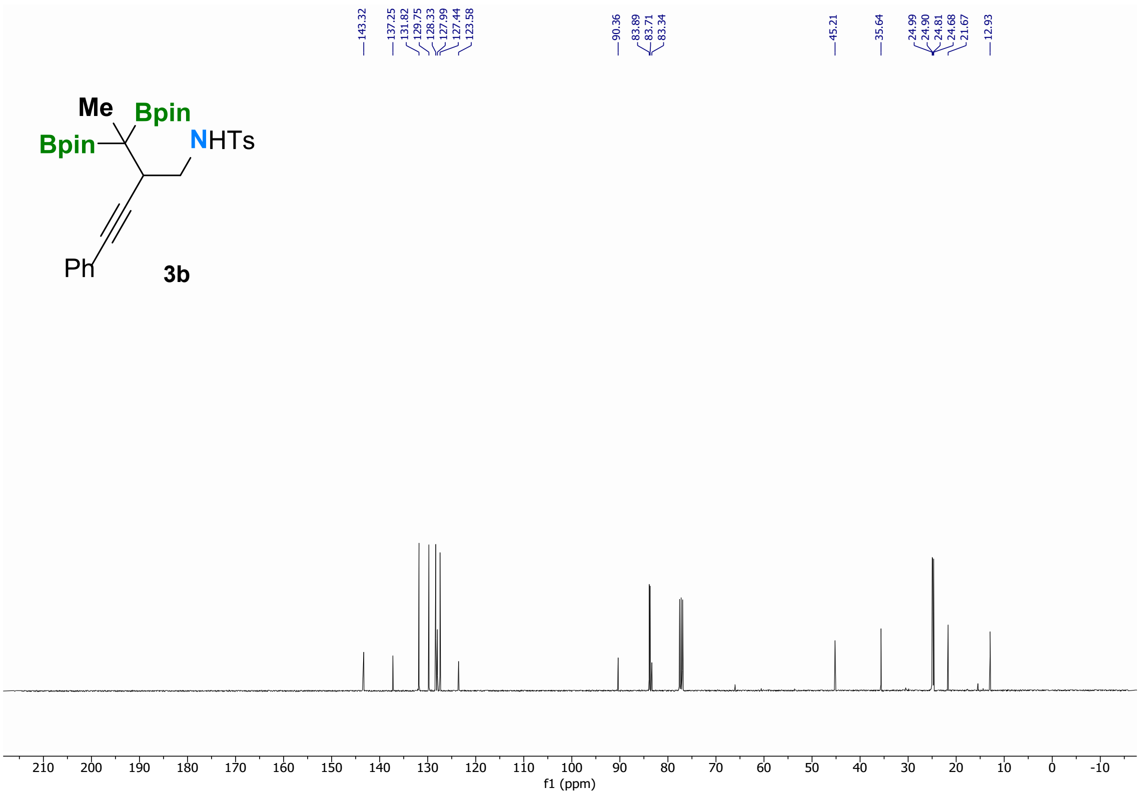

^{11}B NMR (128.3 MHz, $CDCl_3$)

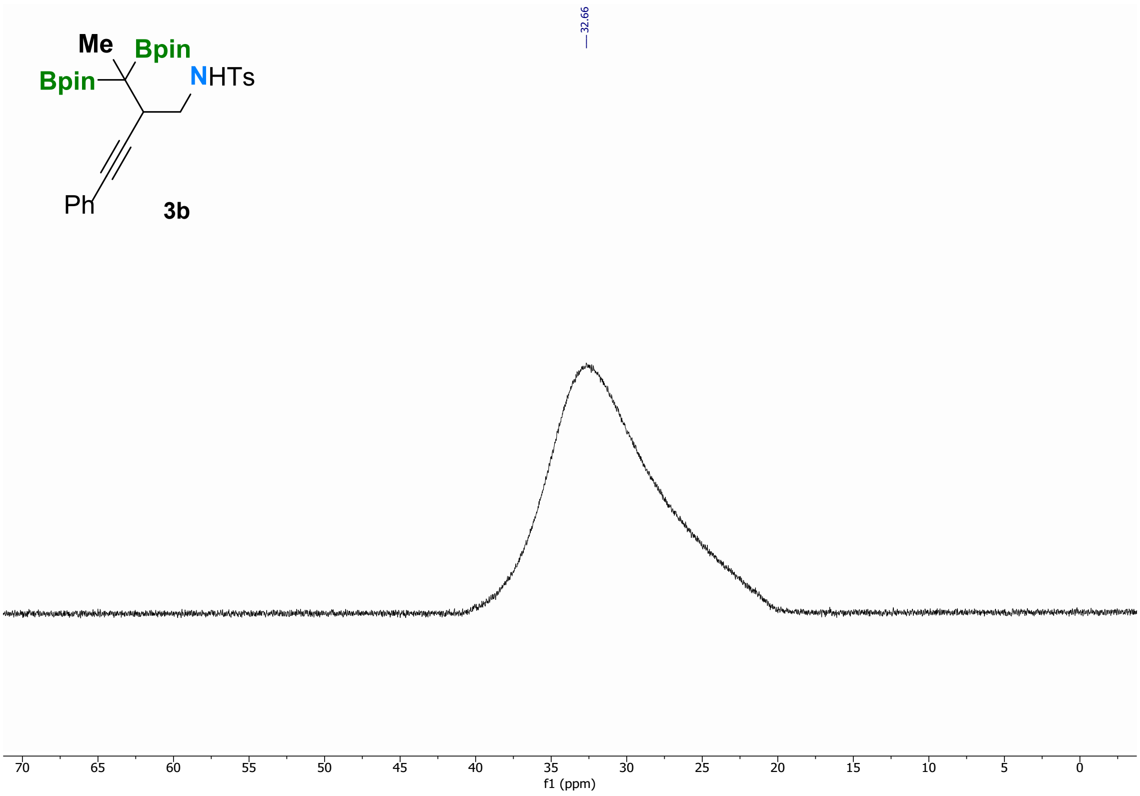

4-methyl-N-(4-methyl-2-(phenylethynyl)-3,3-bis(4,4,5,5-tetramethyl-1,3,2-dioxaborolan-2-yl)pentyl)benzenesulfonamide (3c)

^{1}H NMR (400 MHz, $CDCl_3$)

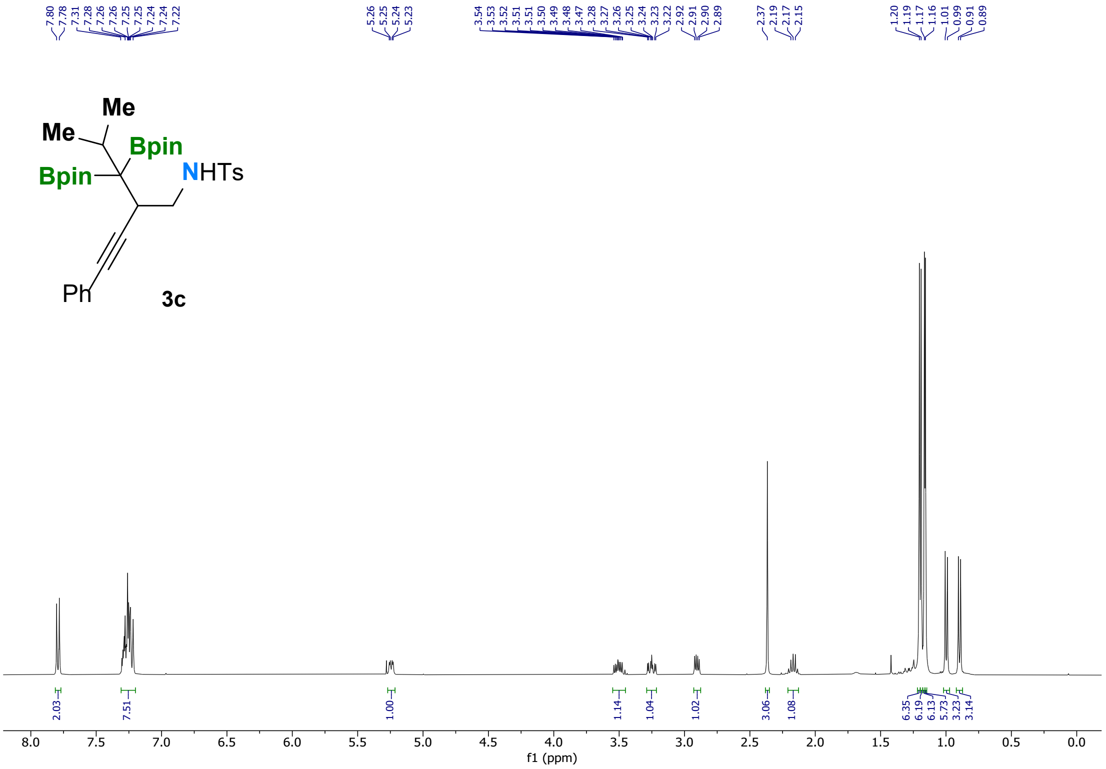

^{13}C NMR {^{1}H} (125 MHz, $CDCl_3$)

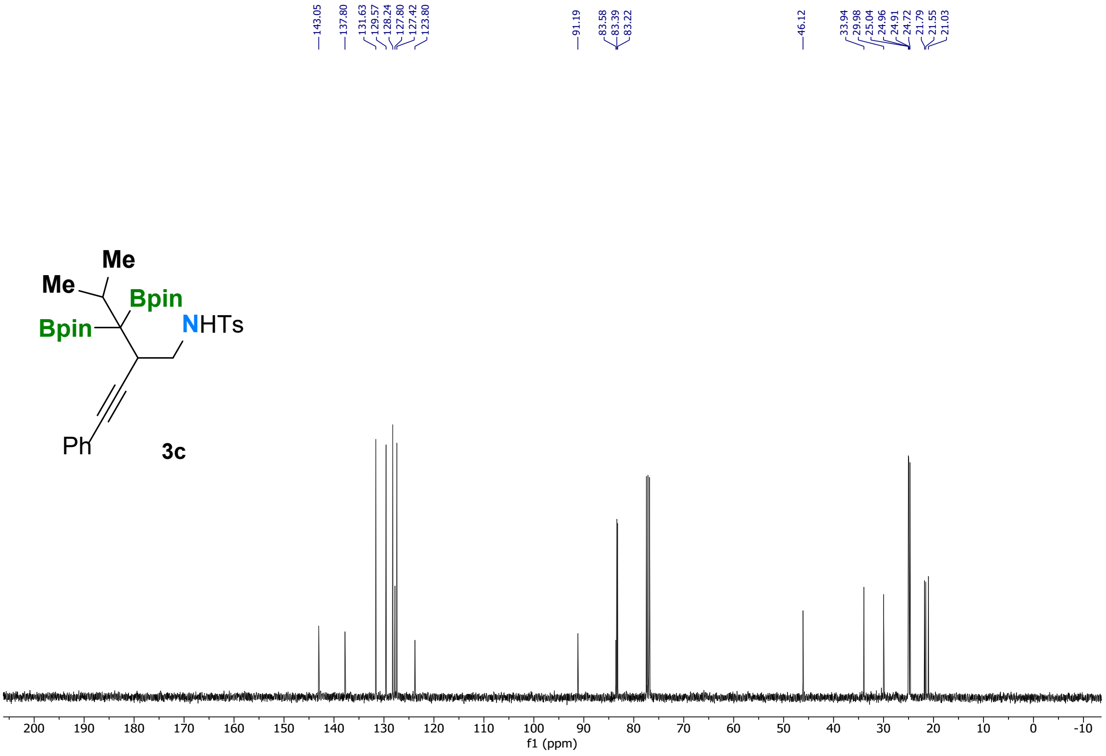

^{11}B NMR (128.3 MHz, CDCl$_3$)

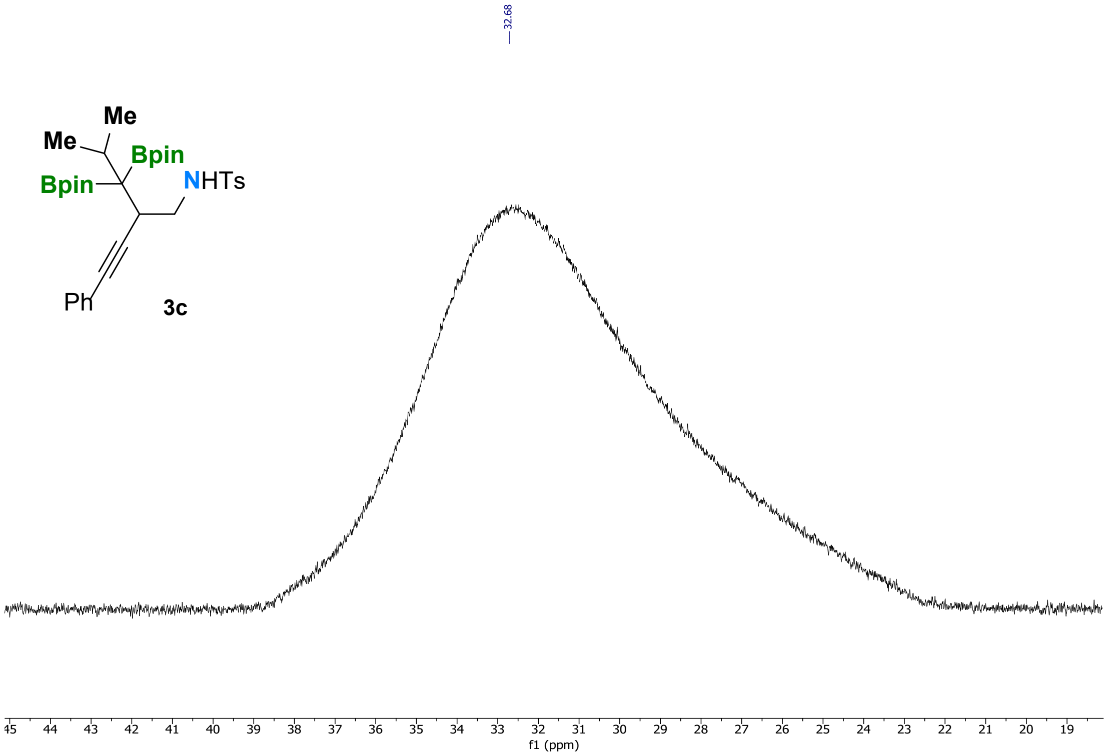

N-(2-(cyclohexylbis(4,4,5,5-tetramethyl-1,3,2-dioxaborolan-2-yl)methyl)-4-phenylbut-3-yn-1-yl)-4-methylbenzenesulfonamide (3d)

^{1}H NMR (400 MHz, CDCl$_3$)

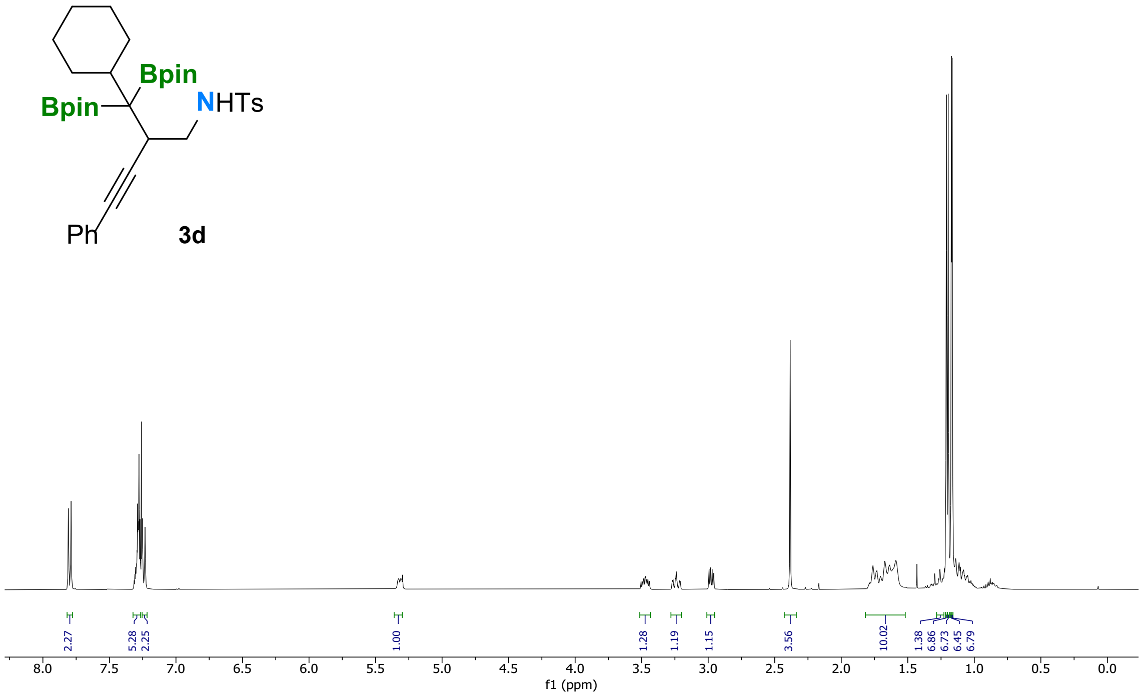

^{13}C NMR {^{1}H} (125 MHz, $CDCl_3$)

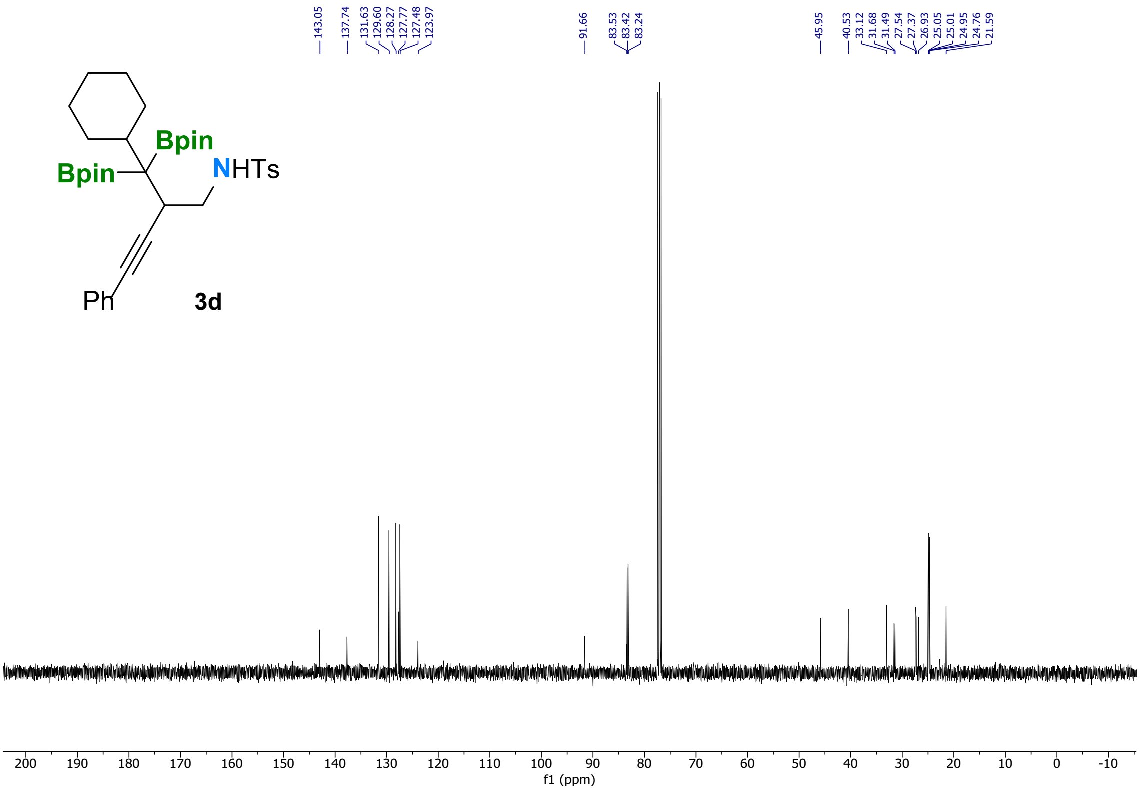

^{11}B NMR (128.3 MHz, $CDCl_3$)

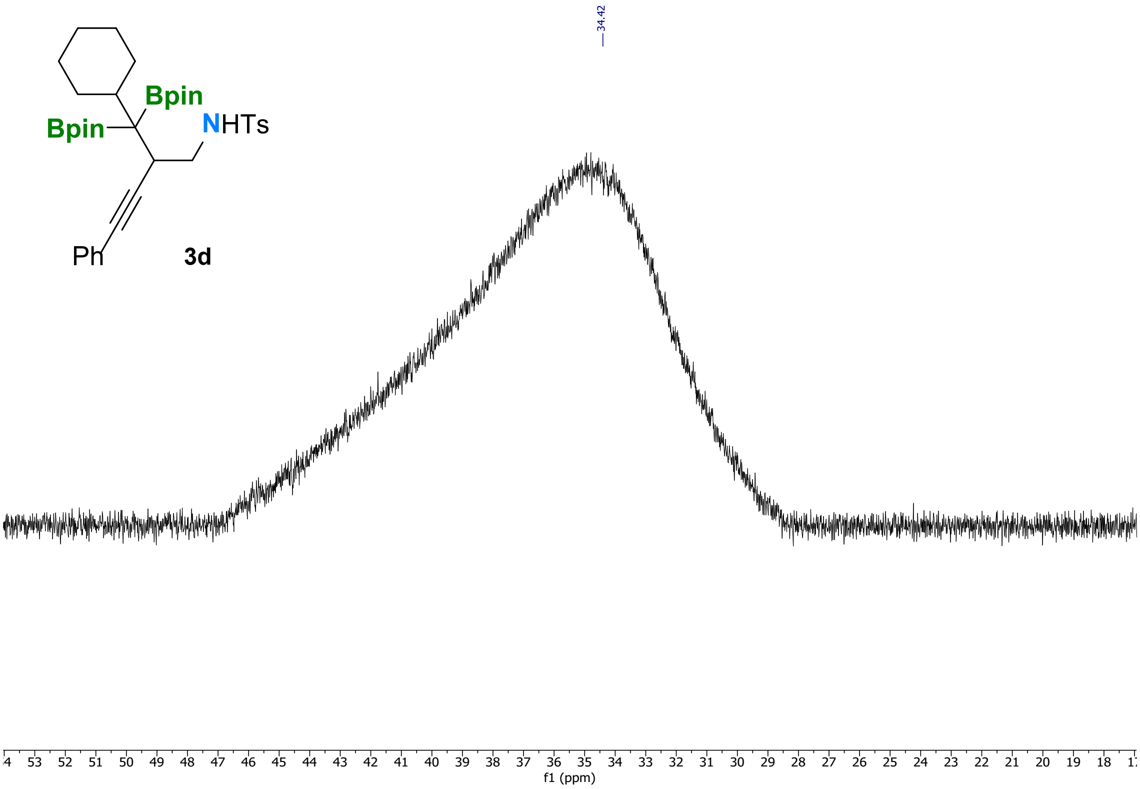

N-(2-(Bis(4,4,5,5-tetramethyl-1,3,2-dioxaborolan-2-yl)(trimethylsilyl)methyl)-4-phenylbut-3-yn-1-yl)-4-methylbenzenesulfonamide (3e)

^{1}H NMR (400 MHz, $CDCl_3$)

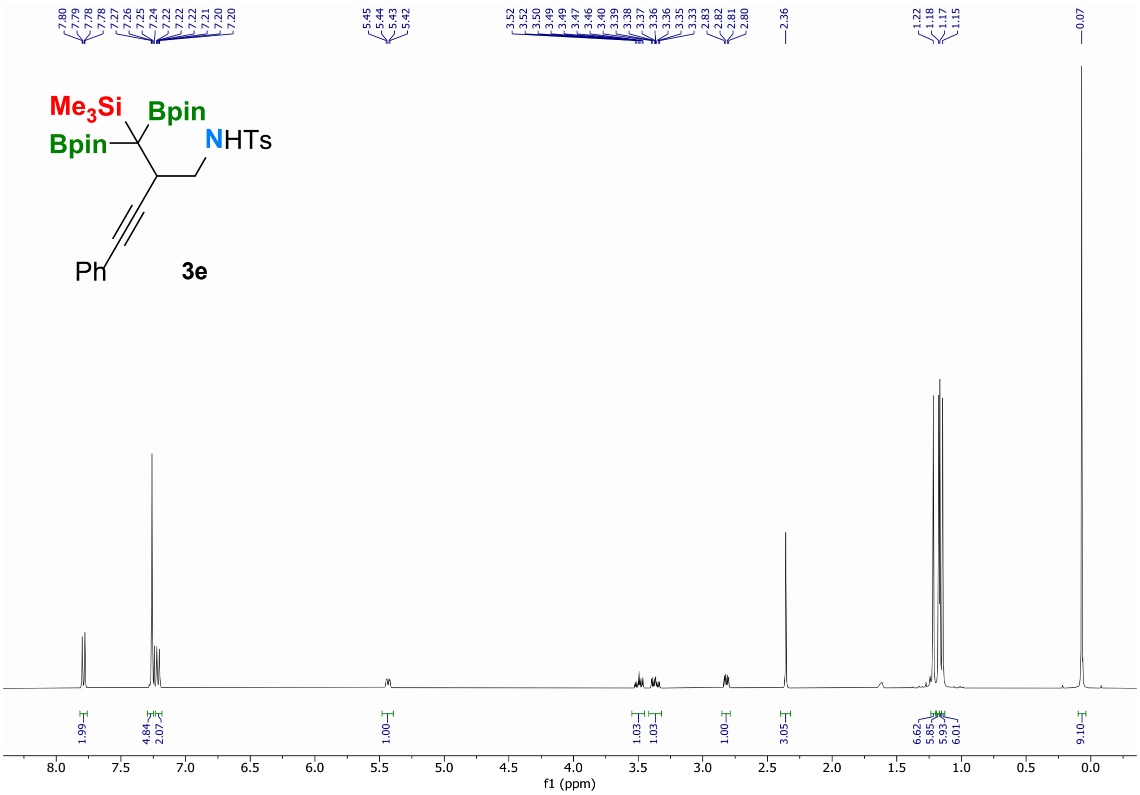

^{13}C NMR {^{1}H} (125 MHz, $CDCl_3$)

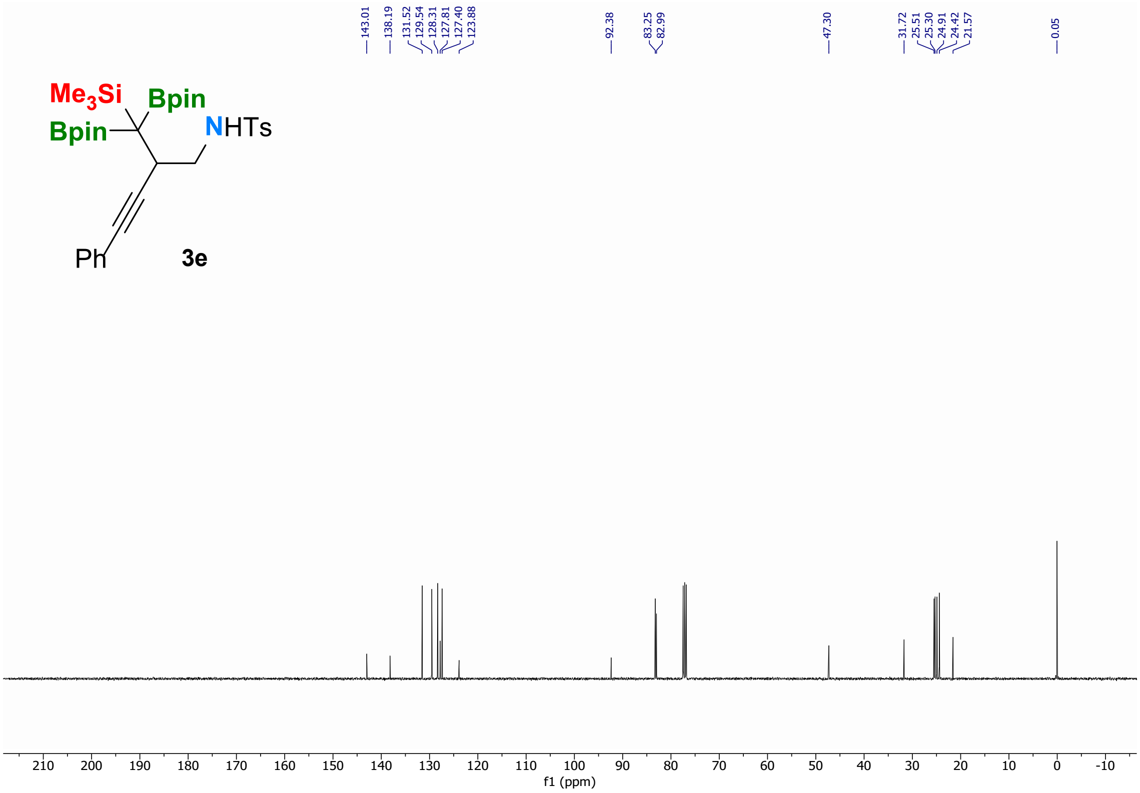

^{11}B NMR (128.3 MHz, CDCl$_3$)

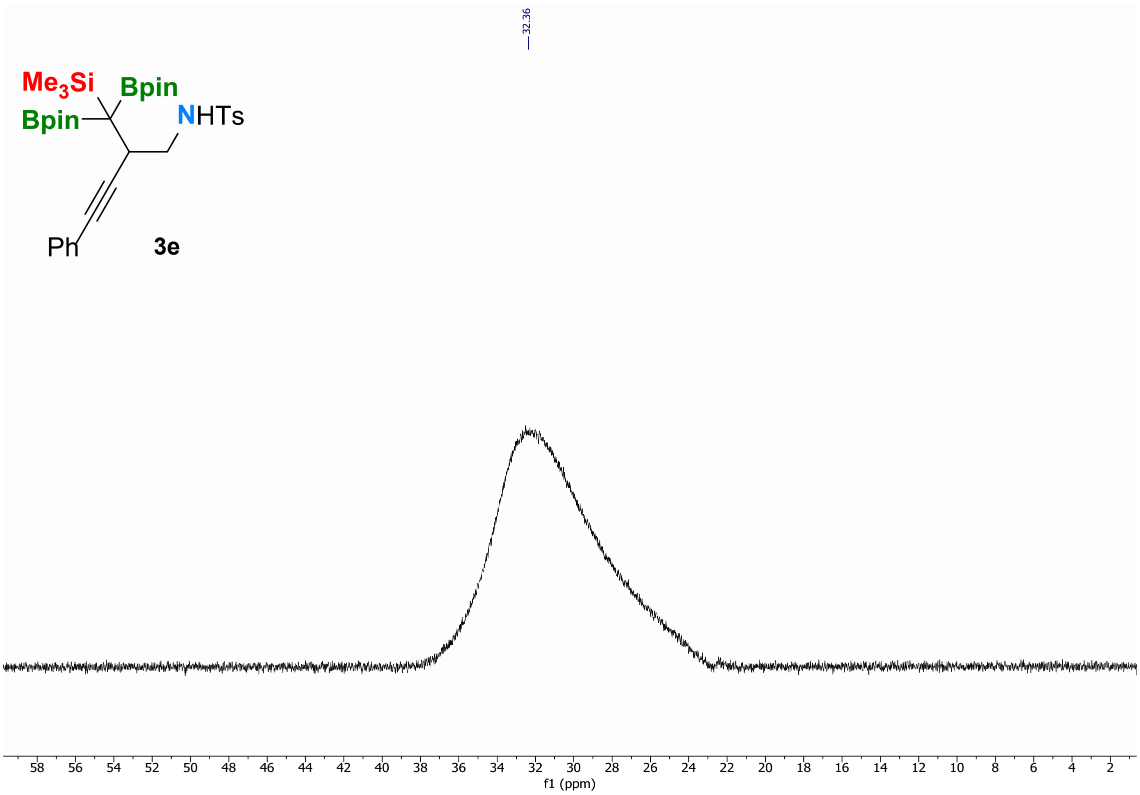

4-methyl-*N*-(1-phenyl-5,5-bis(4,4,5,5-tetramethyl-1,3,2-dioxaborolan-2-yl)hex-1-yn-3-yl)benzenesulfonamide (4b)

^{1}H NMR (400 MHz, CDCl$_3$)

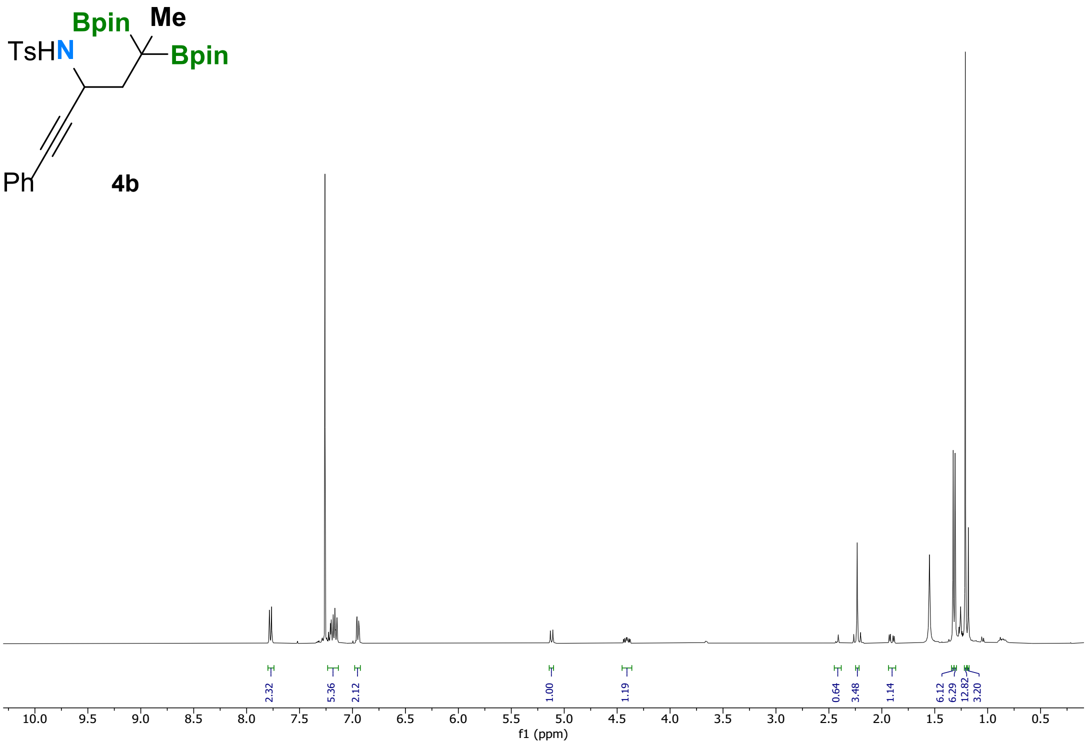

^{13}C NMR {^{1}H} (125 MHz, CDCl$_3$)

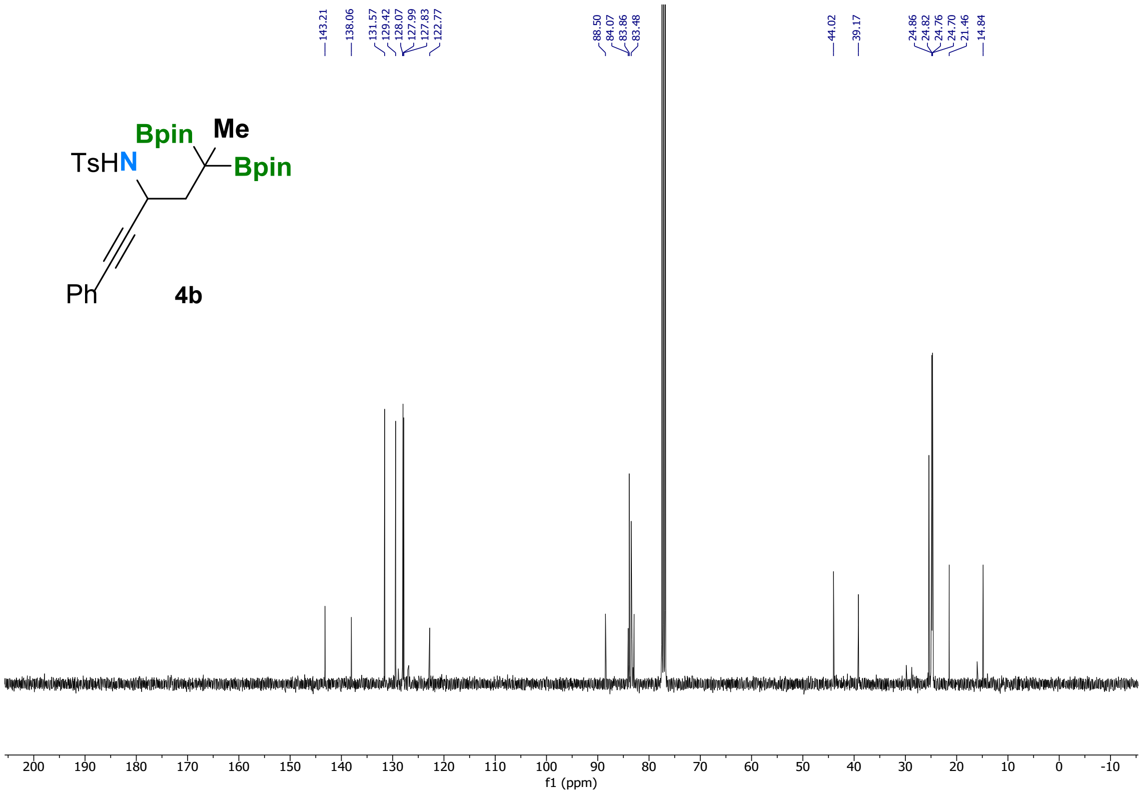

^{11}B NMR (128.3 MHz, $CDCl_3$)

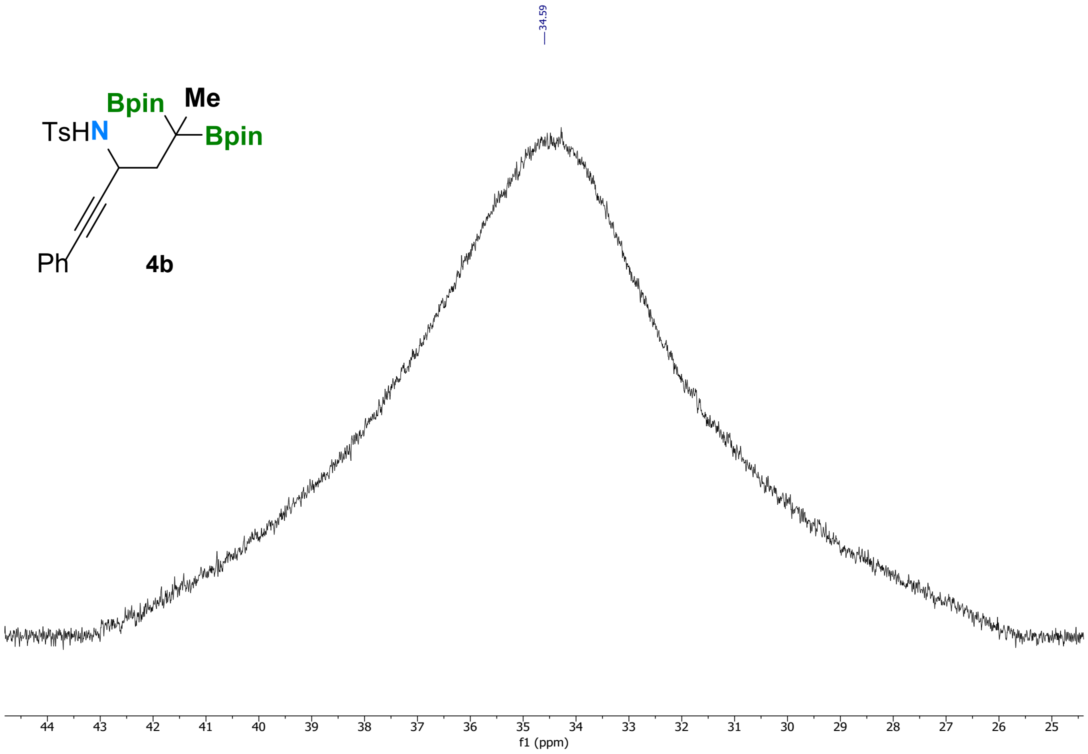

4-methyl-*N*-(1-phenyl-5,5-bis(4,4,5,5-tetramethyl-1,3,2-dioxaborolan-2-yl)-5-(trimethylsilyl)pent-1-yn-3-yl)benzenesulfonamide (4e)

^{1}H NMR (400 MHz, $CDCl_3$)

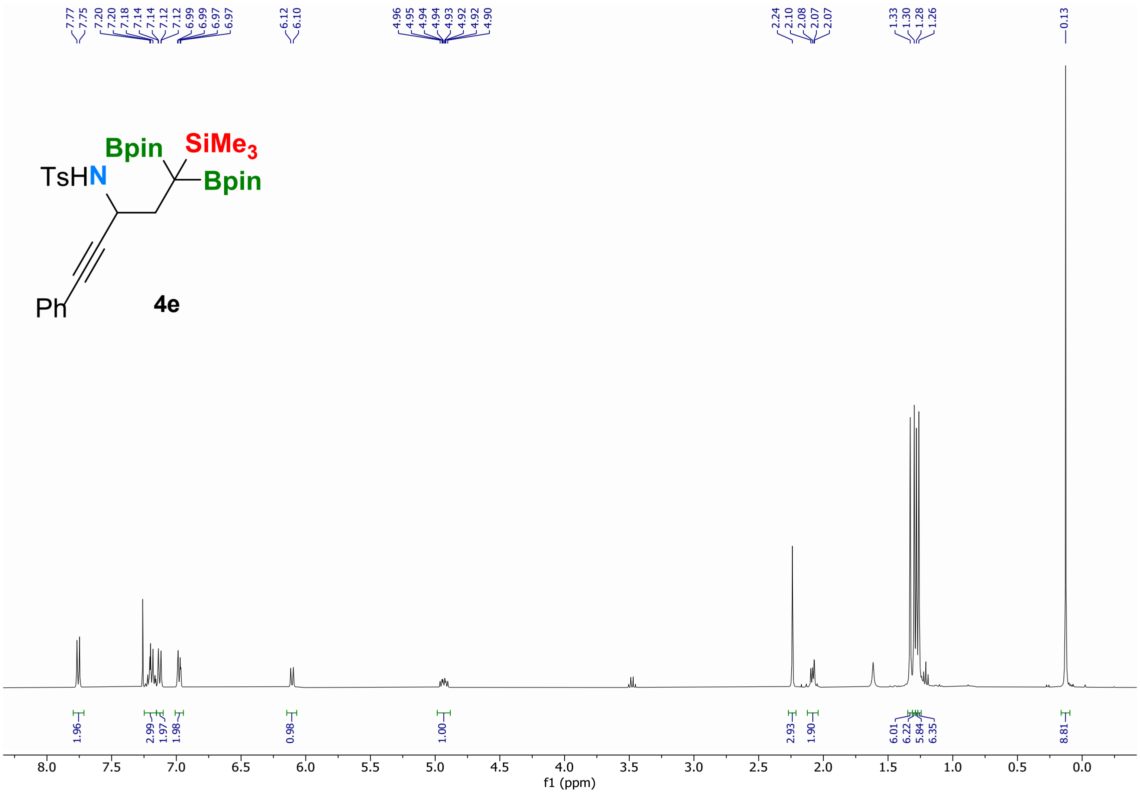

^{13}C NMR {^{1}H} (125 MHz, $CDCl_3$)

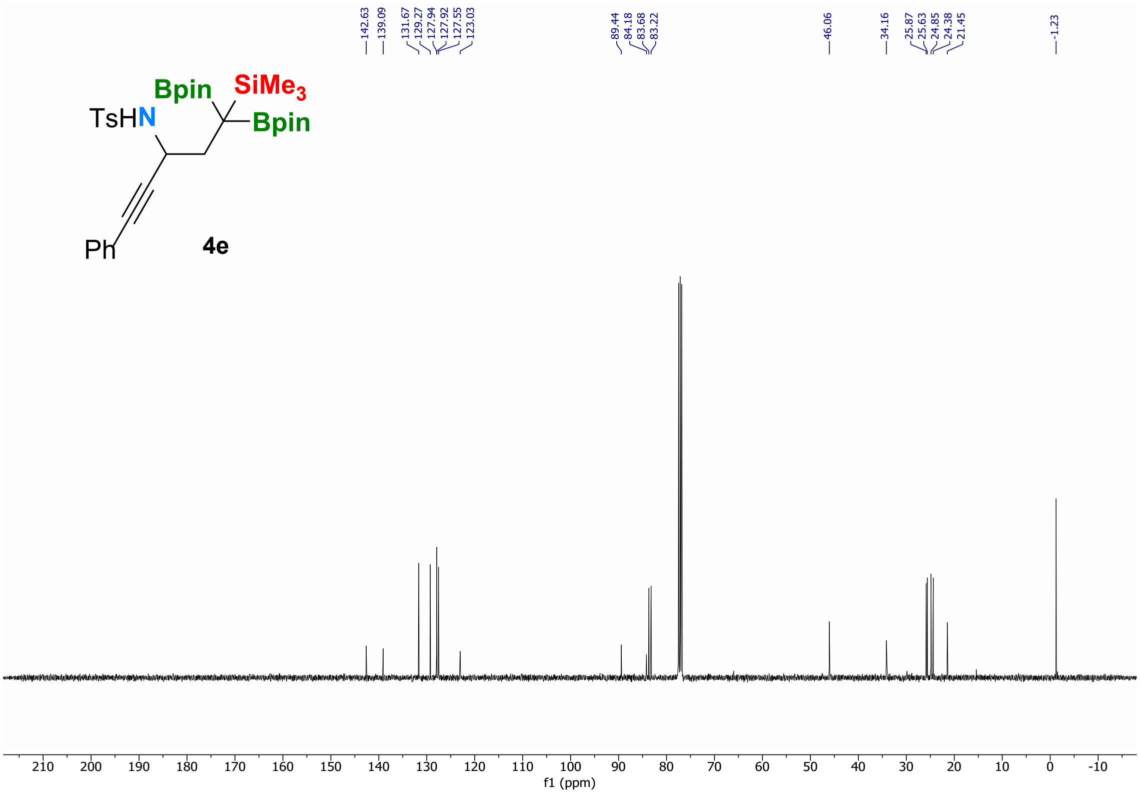

^{11}B NMR (128.3 MHz, $CDCl_3$)

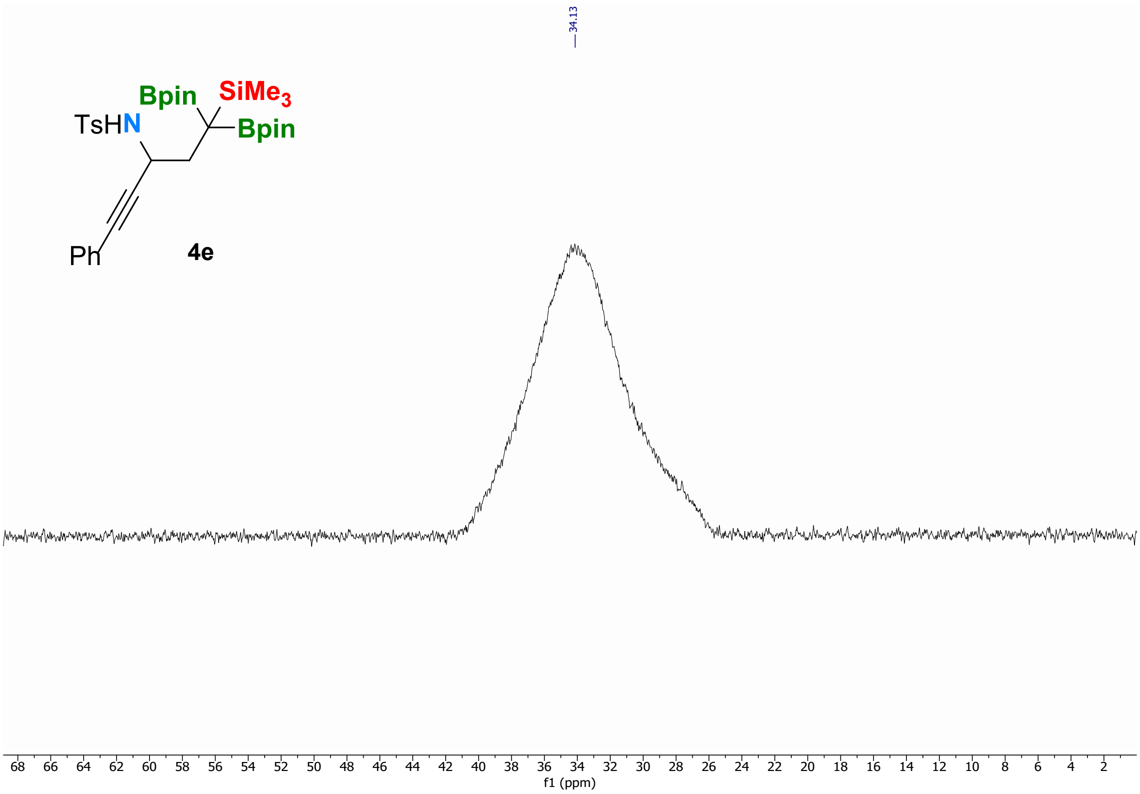

_N_-(2-(bis(4,4,5,5-tetramethyl-1,3,2-dioxaborolan-2-yl)methyl)-4-(4-methoxyphenyl)but-3-yn-1-yl)-4-methylbenzenesulfonamide (6a)

^{1}H NMR (400 MHz, $CDCl_3$)

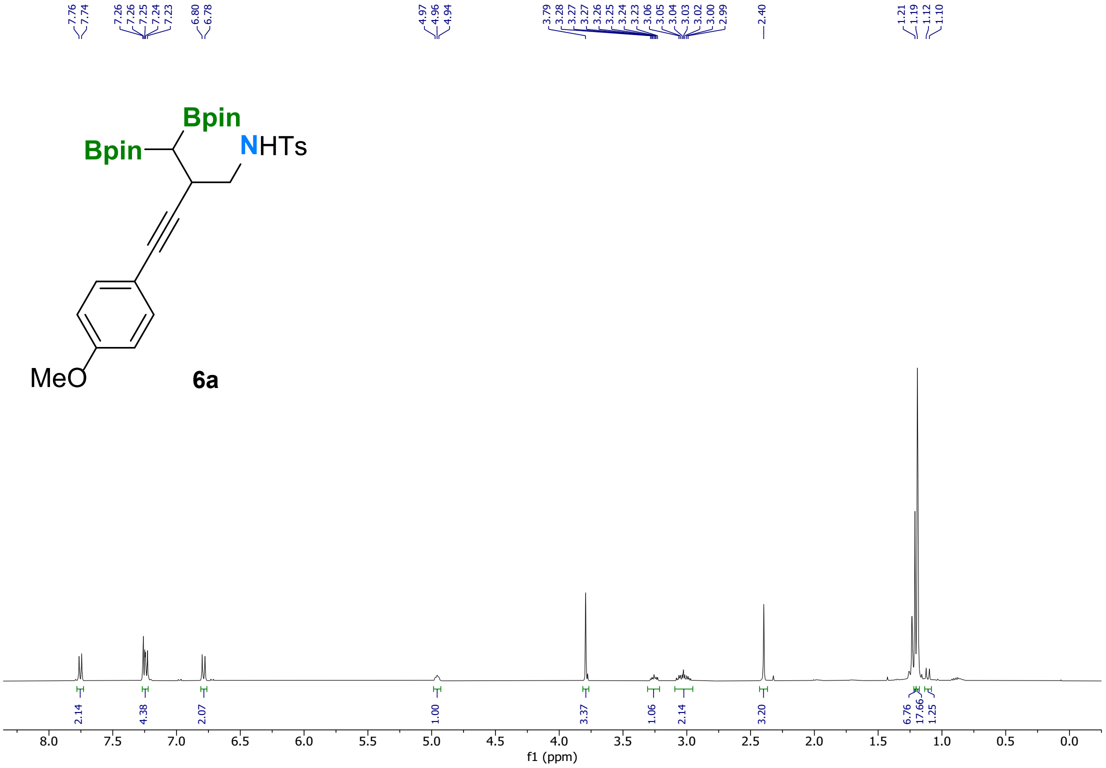

^{13}C NMR {^1H} (125 MHz, $CDCl_3$)

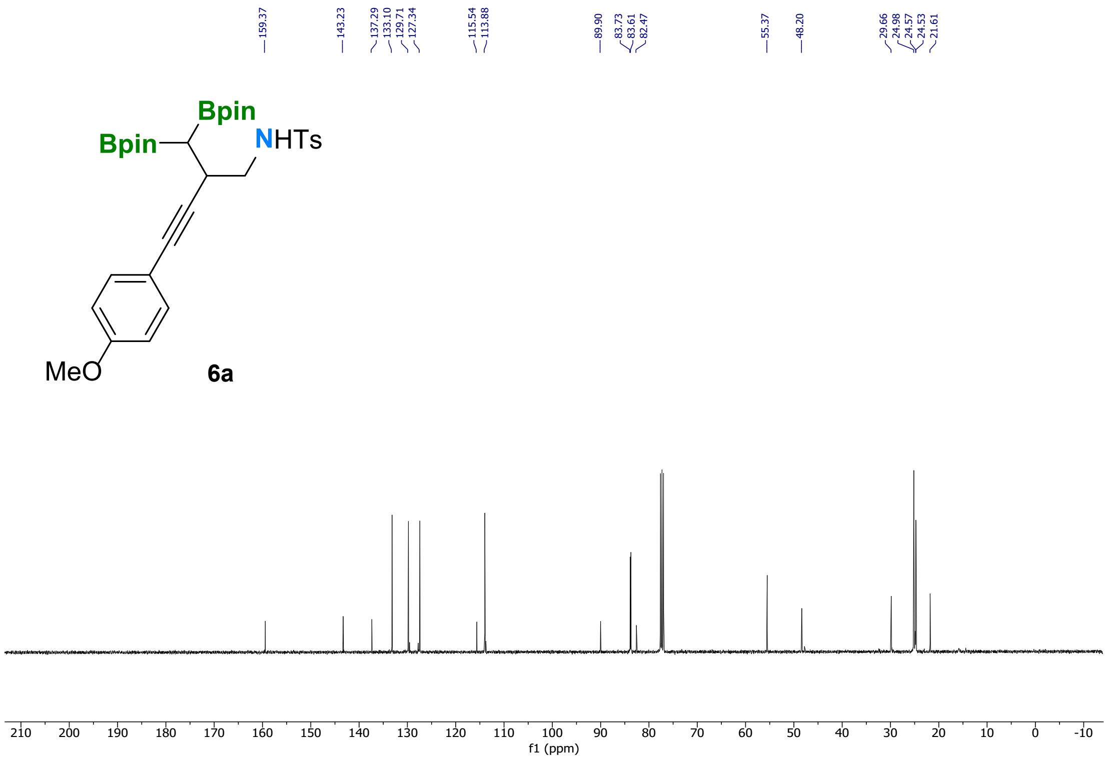

^{11}B NMR (128.3 MHz, $CDCl_3$)

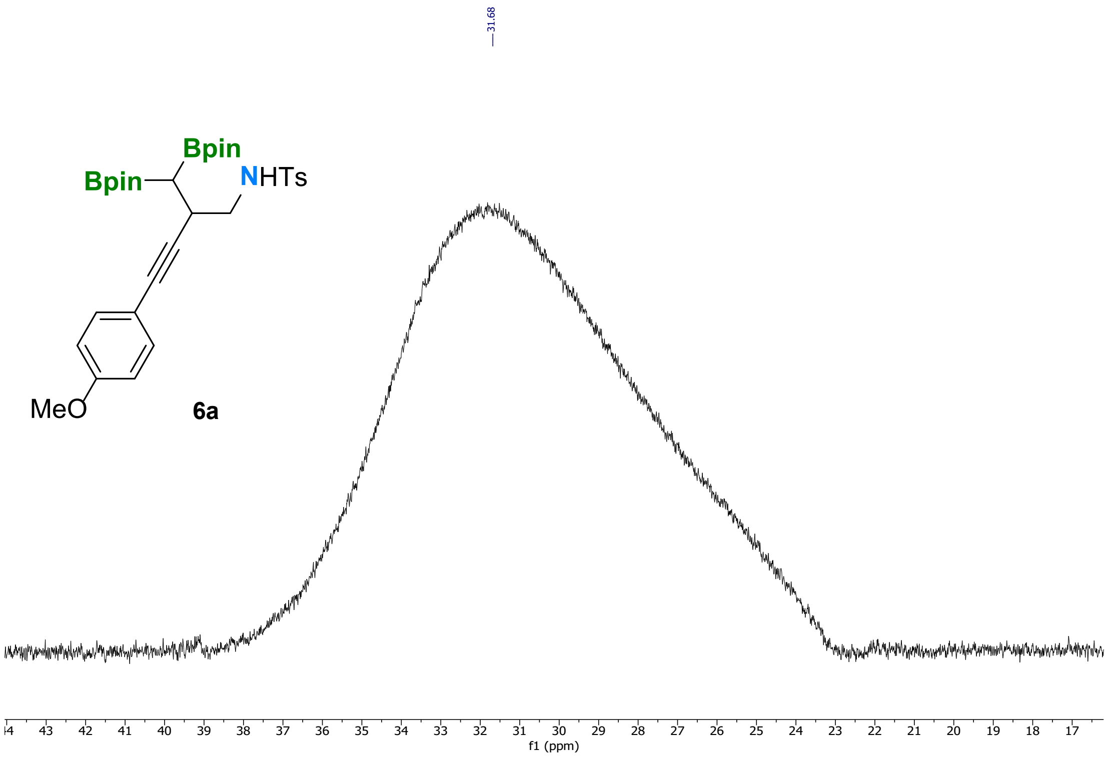

N-(2-(1,1-bis(4,4,5,5-tetramethyl-1,3,2-dioxaborolan-2-yl)ethyl)-4-(4-methoxyphenyl)but-3-yn-1-yl)-4-methylbenzenesulfonamide (6b)

^{1}H NMR (400 MHz, CDCl$_3$)

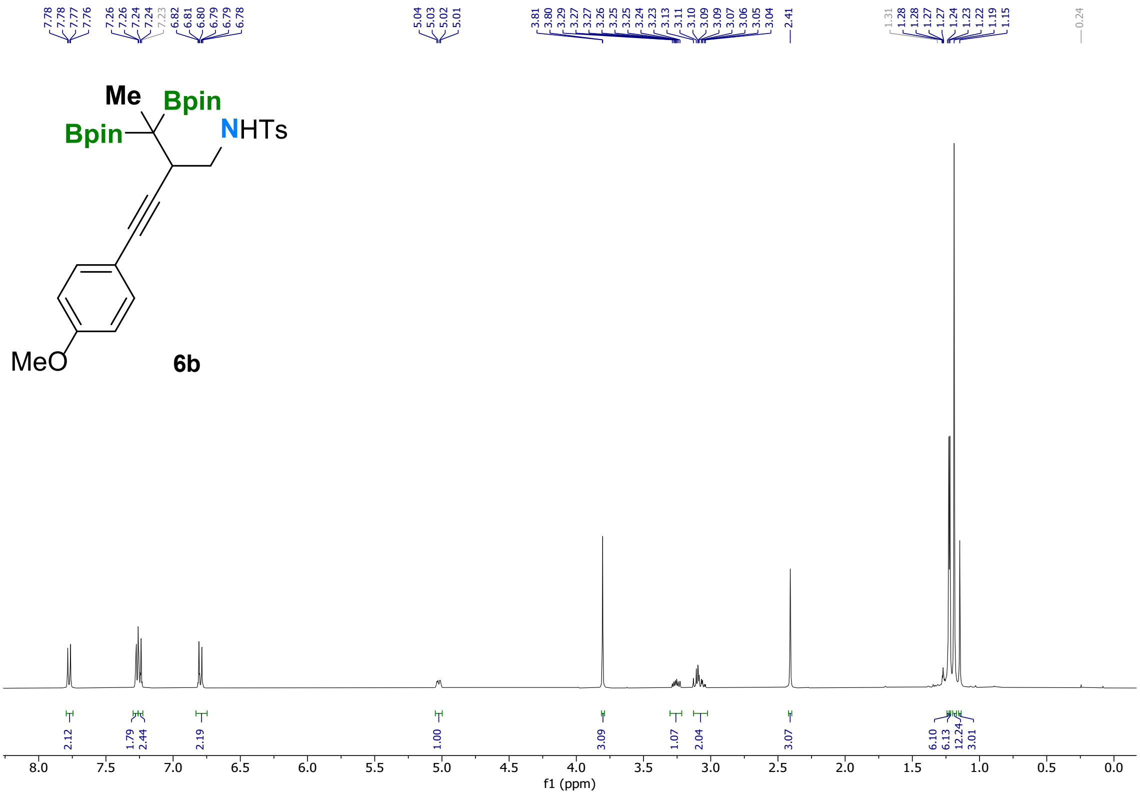

^{13}C NMR {^{1}H} (125 MHz, CDCl$_3$)

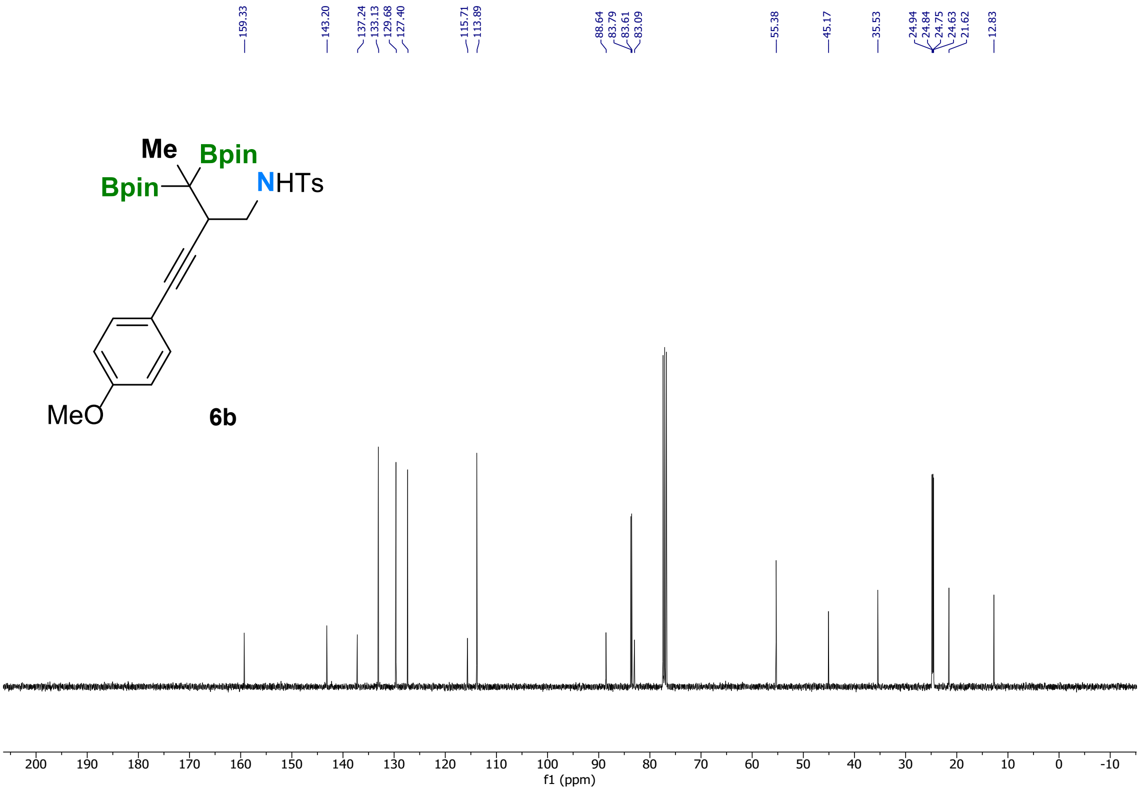

^{11}B NMR (128.3 MHz, $CDCl_3$)

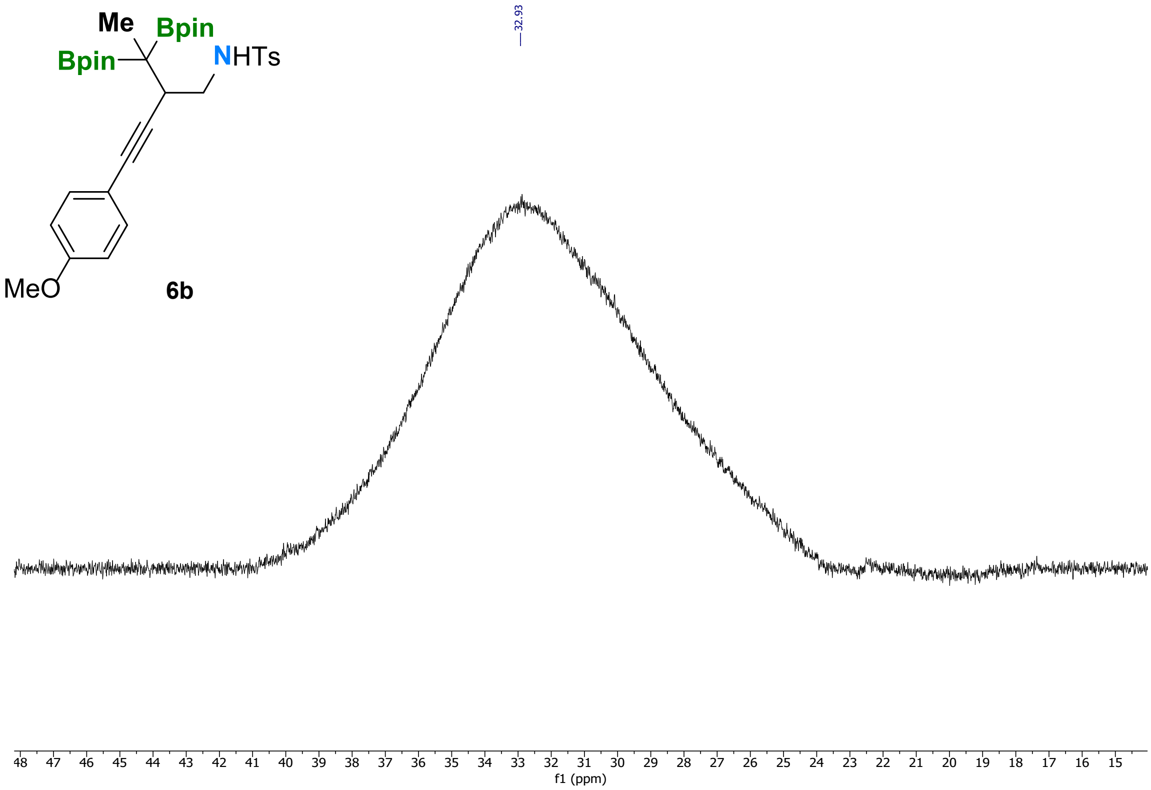

6b

***N*-(2-(bis(4,4,5,5-tetramethyl-1,3,2-dioxaborolan-2-yl)(trimethylsilyl)methyl)-4-(4-methoxyphenyl)but-3-yn-1-yl)-4-methylbenzenesulfonamide (6e)**

^{1}H NMR (400 MHz, $CDCl_3$)

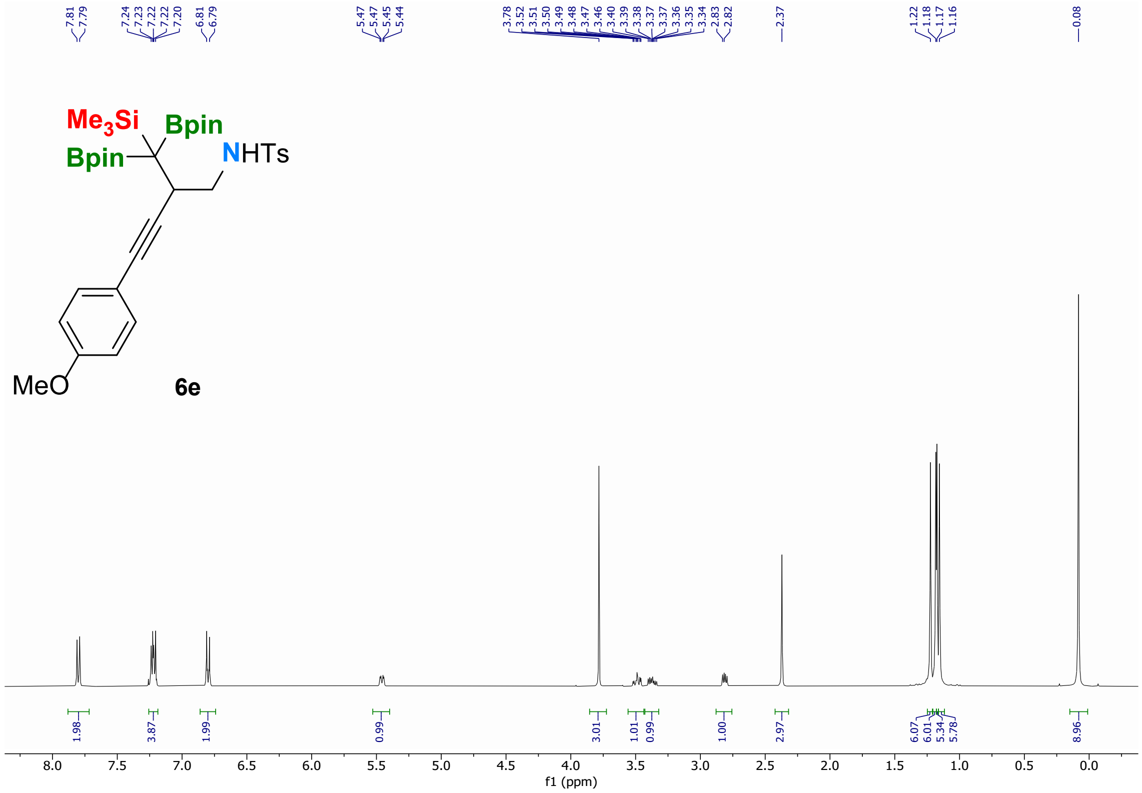

6e

^{13}C NMR {^{1}H} (125 MHz, $CDCl_3$)

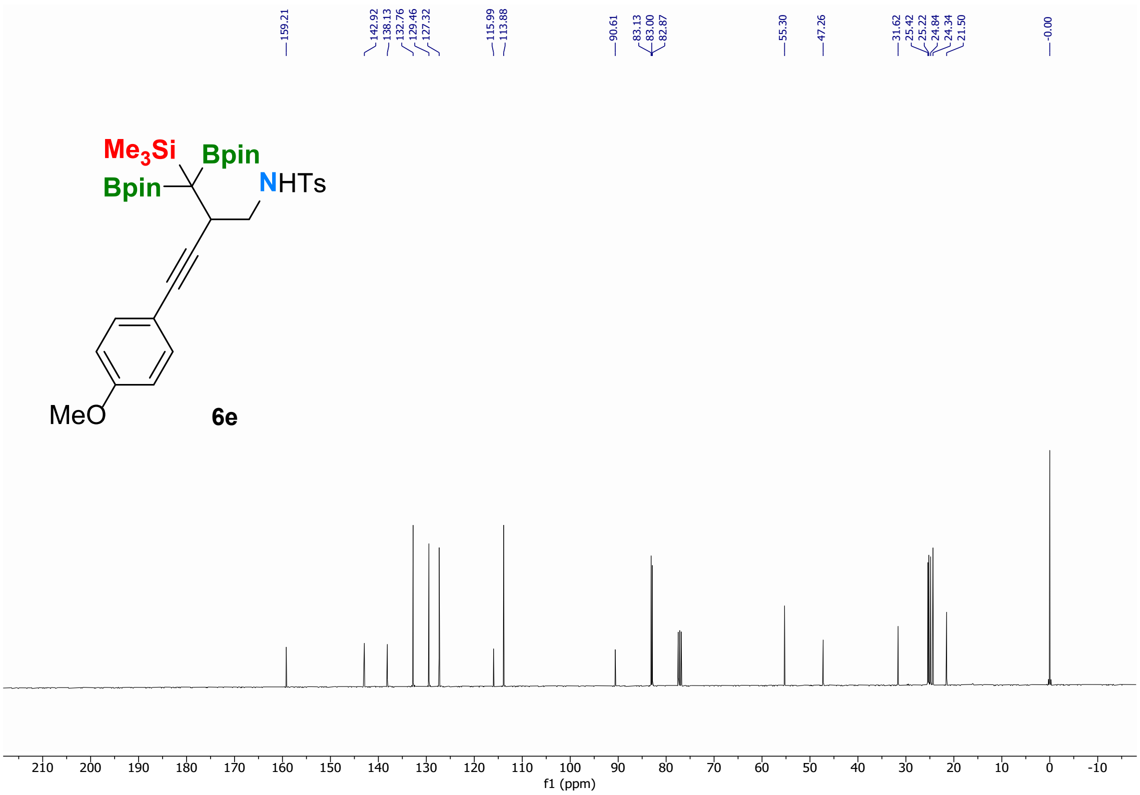

^{11}B NMR (128.3 MHz, $CDCl_3$)

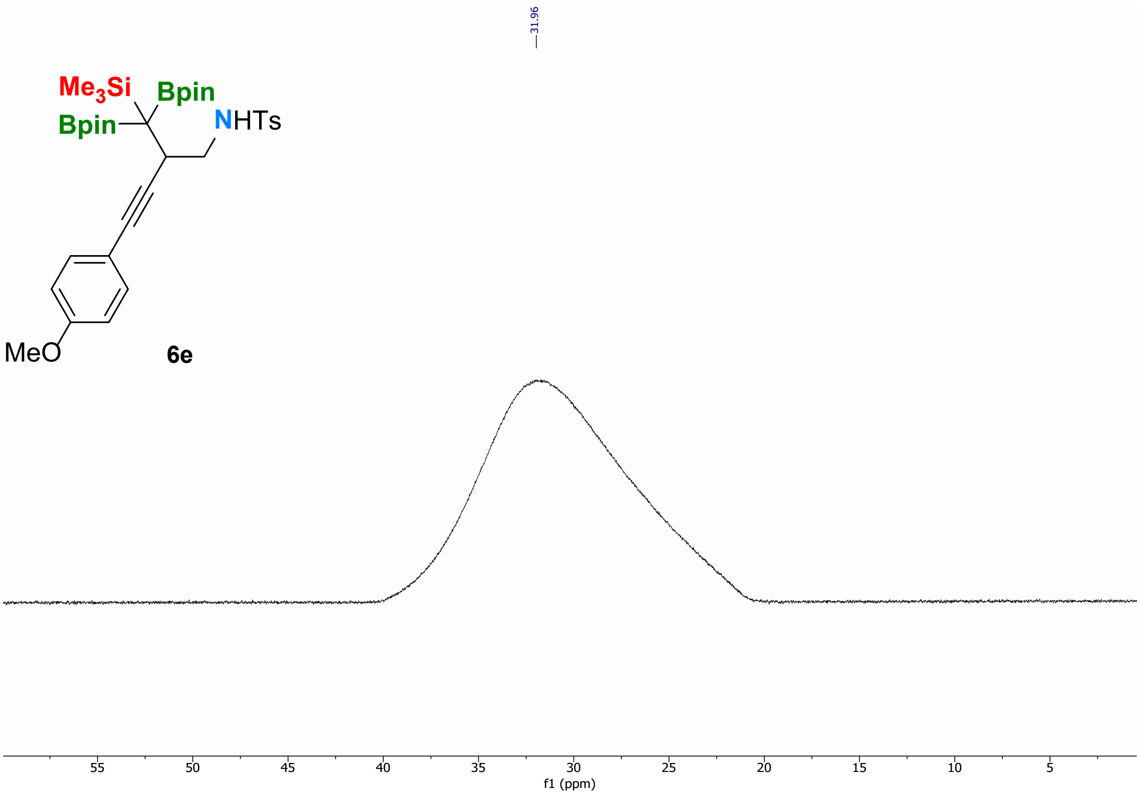

***N*-(2-(bis(4,4,5,5-tetramethyl-1,3,2-dioxaborolan-2-yl)methyl)-4-(4-chlorophenyl)but-3-yn-1-yl)-4-methylbenzenesulfonamide (9a)**

^{1}H NMR (400 MHz, CDCl$_3$)

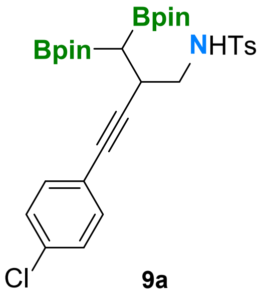

9a

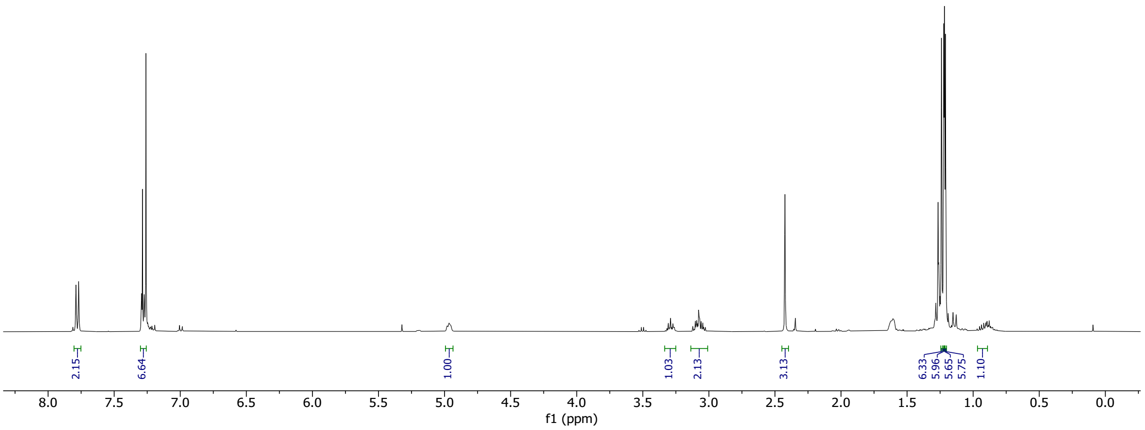

^{13}C NMR {^{1}H} (125 MHz, CDCl$_3$)

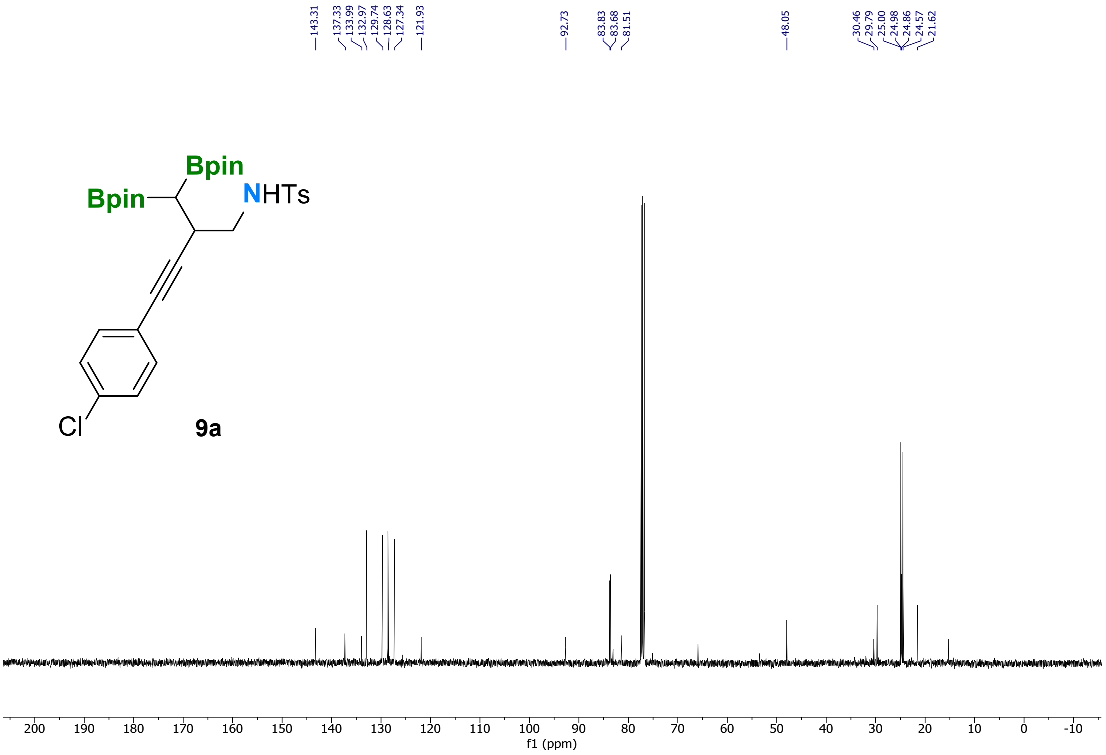

9a

^{11}B NMR (128.3 MHz, $CDCl_3$)

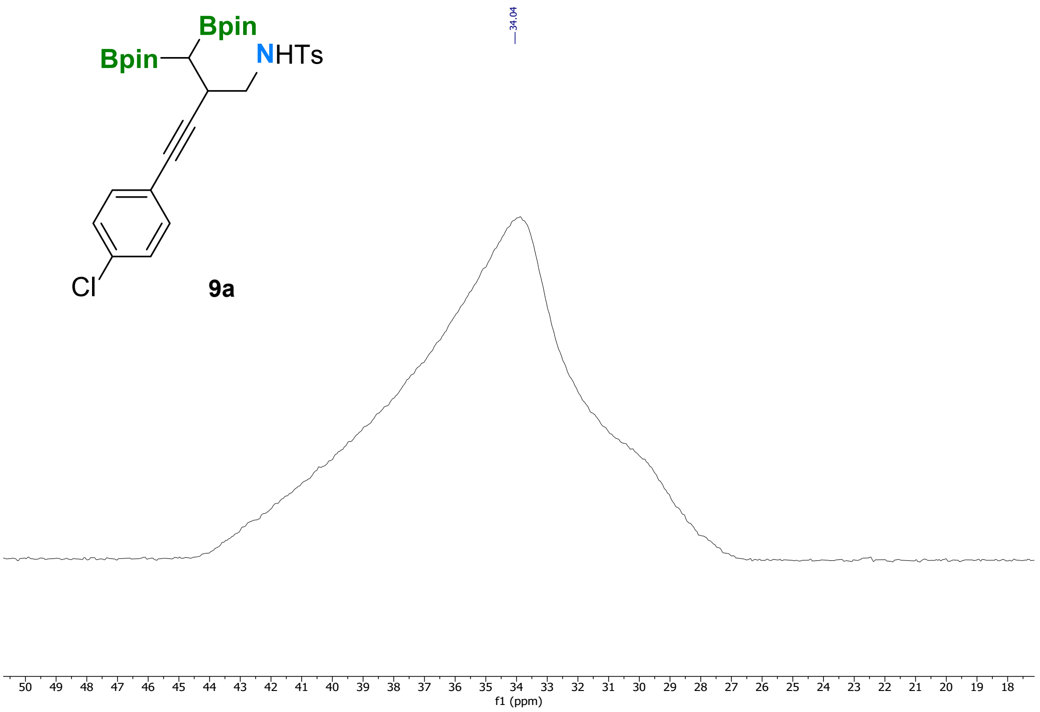

_N_-(2-(1,1-bis(4,4,5,5-tetramethyl-1,3,2-dioxaborolan-2-yl)ethyl)-4-(4-chlorophenyl)but-3-yn-1-yl)-4-methylbenzenesulfonamide (9b)

^{1}H NMR (400 MHz, $CDCl_3$)

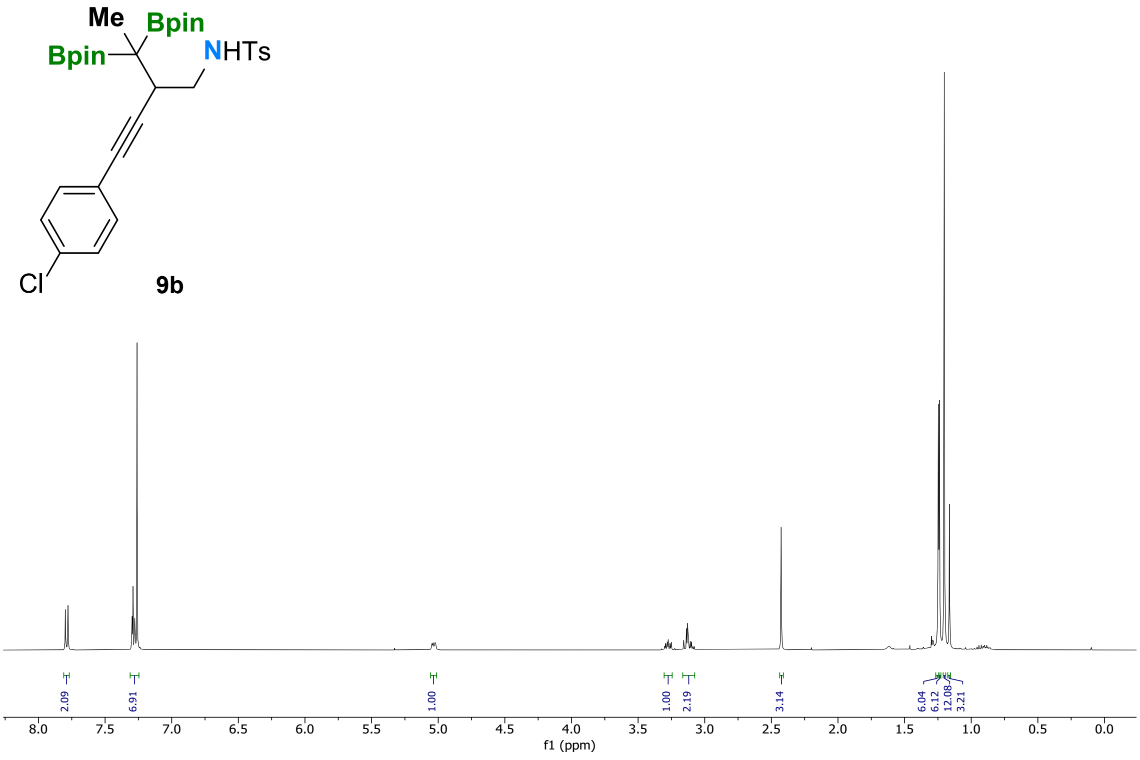

^{13}C NMR {^{1}H} (125 MHz, $CDCl_3$)

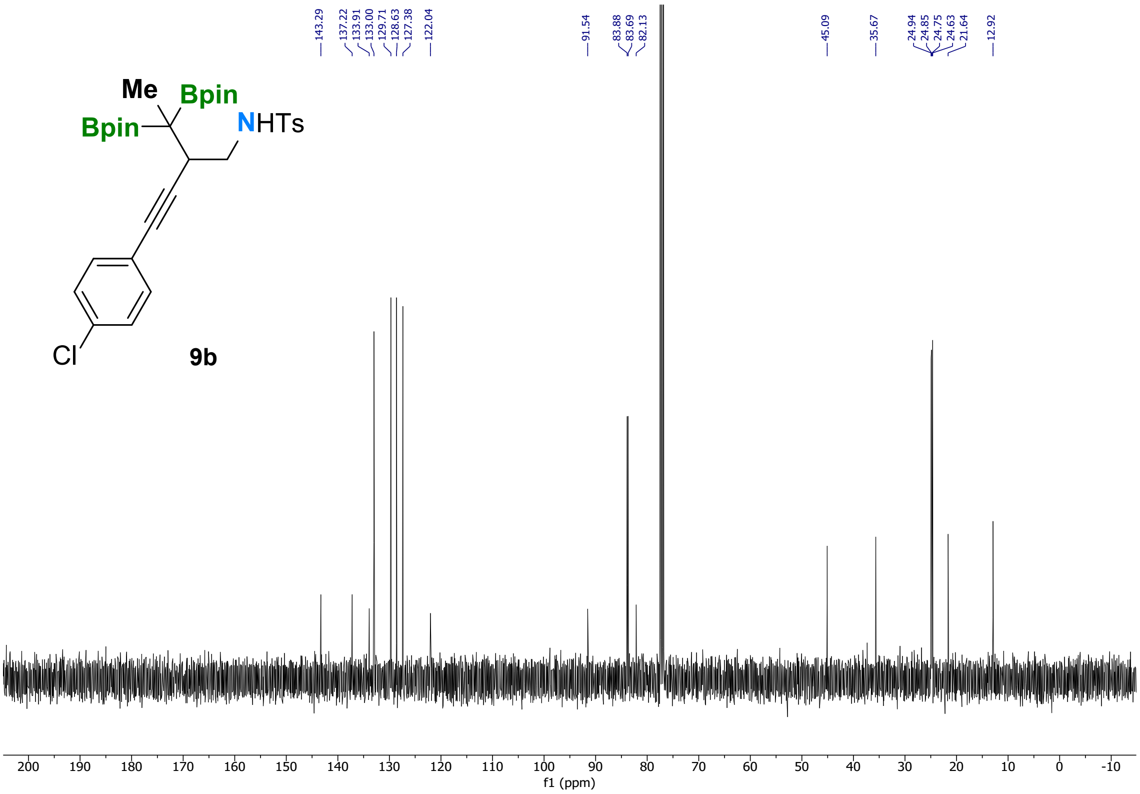

^{11}B NMR (128.3 MHz, $CDCl_3$)

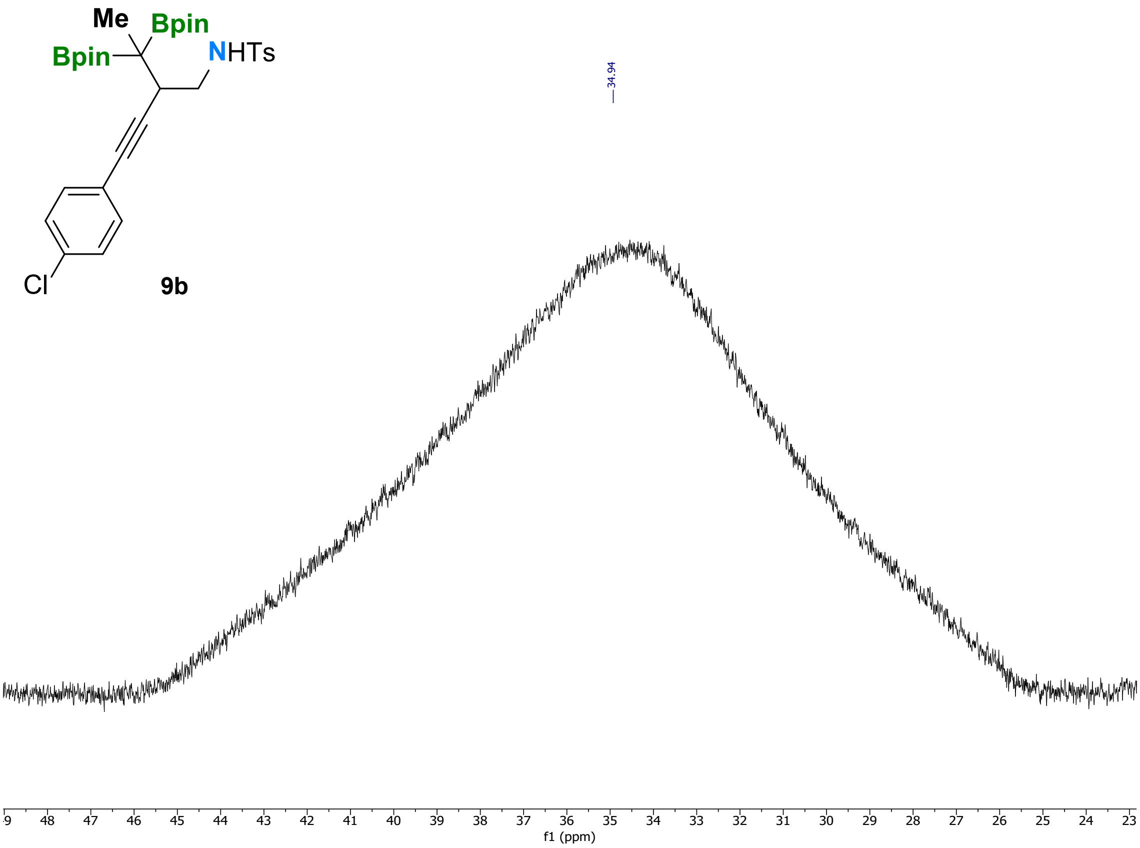

_N_-(2-(bis(4,4,5,5-tetramethyl-1,3,2-dioxaborolan-2-yl)(trimethylsilyl)methyl)-4-(4-chlorophenyl)but-3-yn-1-yl)-4-methylbenzenesulfonamide (9e)

^{1}H NMR (400 MHz, $CDCl_3$)

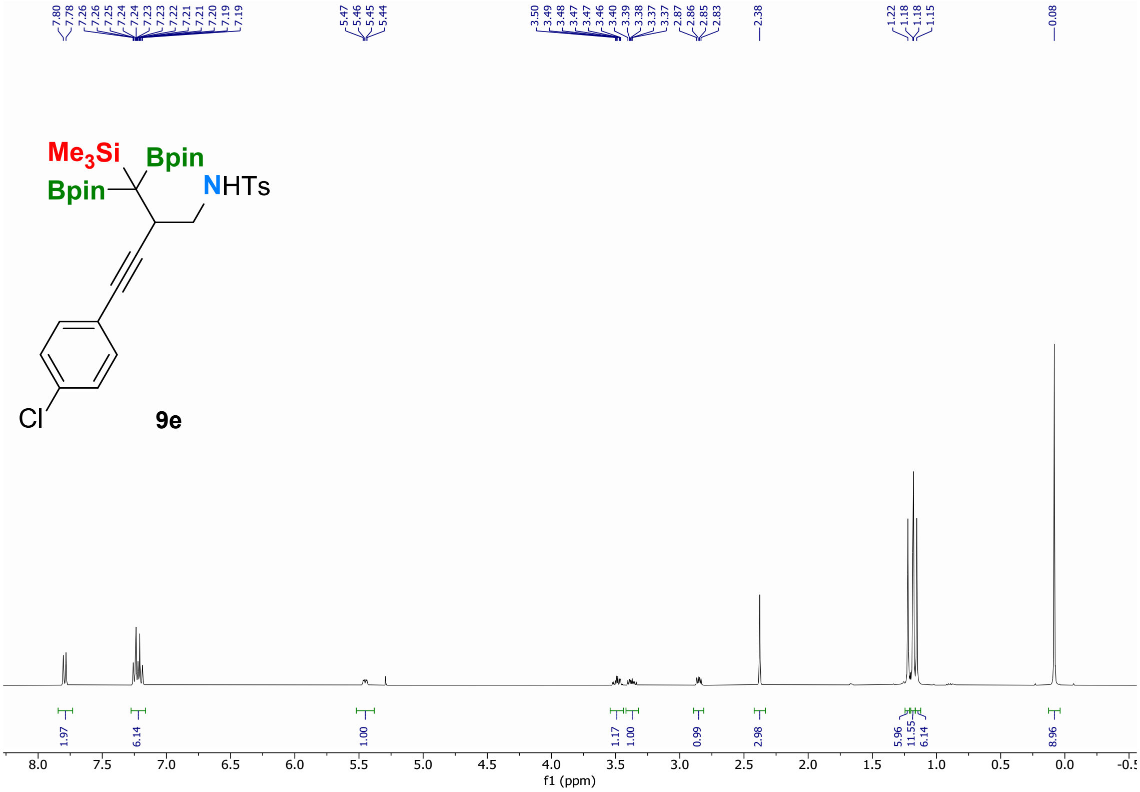

^{13}C NMR {^{1}H} (125 MHz, $CDCl_3$)

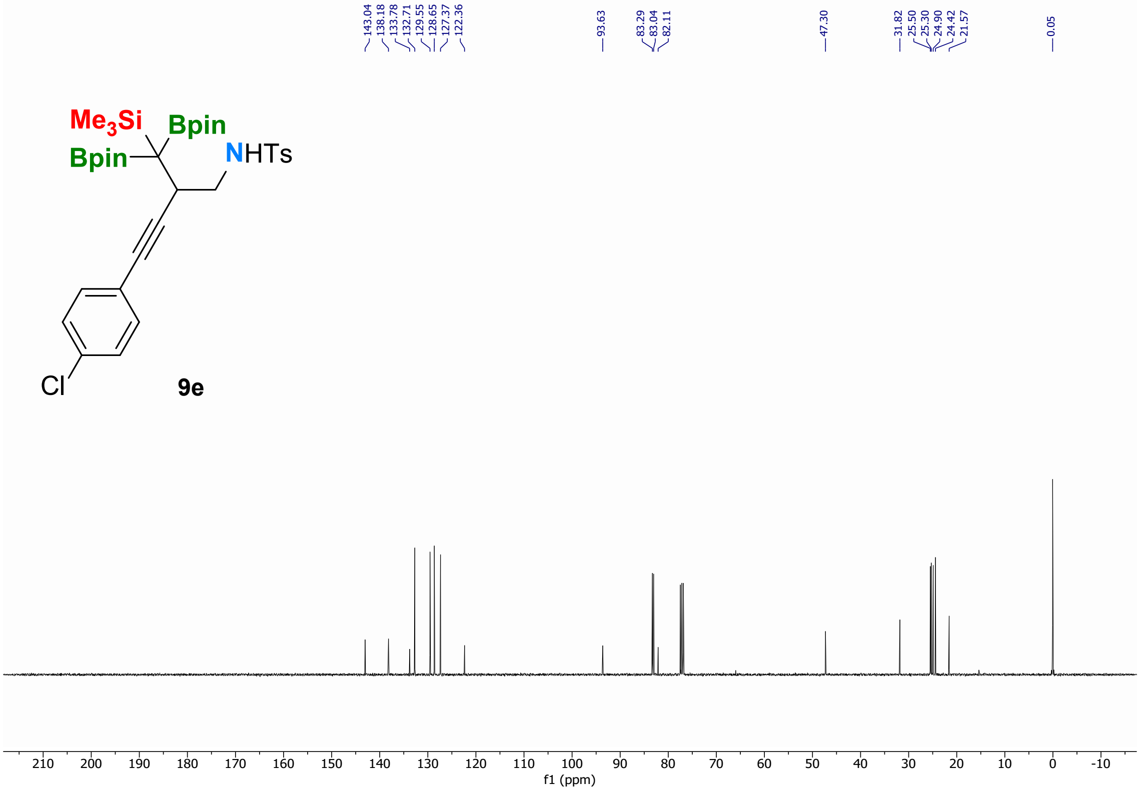

^{11}B NMR (128.3 MHz, $CDCl_3$)

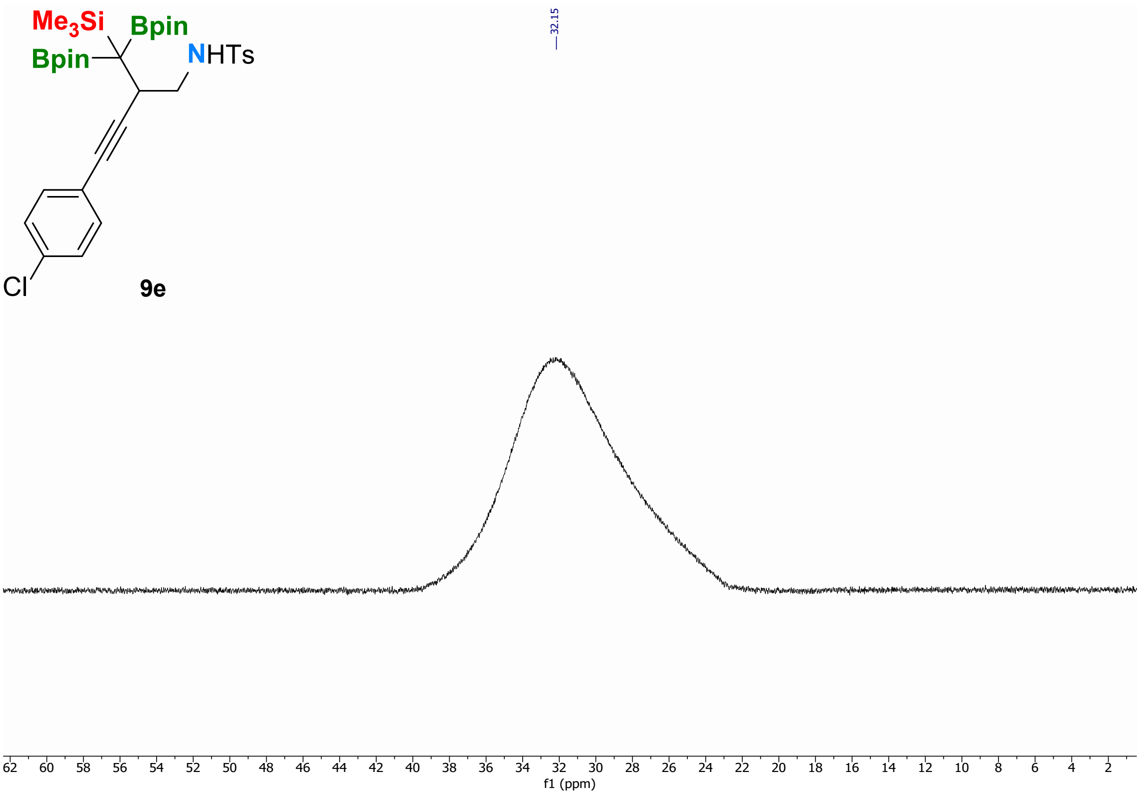

***N*-(2-(bis(4,4,5,5-tetramethyl-1,3,2-dioxaborolan-2-yl)(trimethylsilyl)methyl)-4-(4-chlorophenyl)but-3-yn-1-yl)-4-methylbenzenesulfonamide (10e)**

^{1}H NMR (400 MHz, $CDCl_3$)

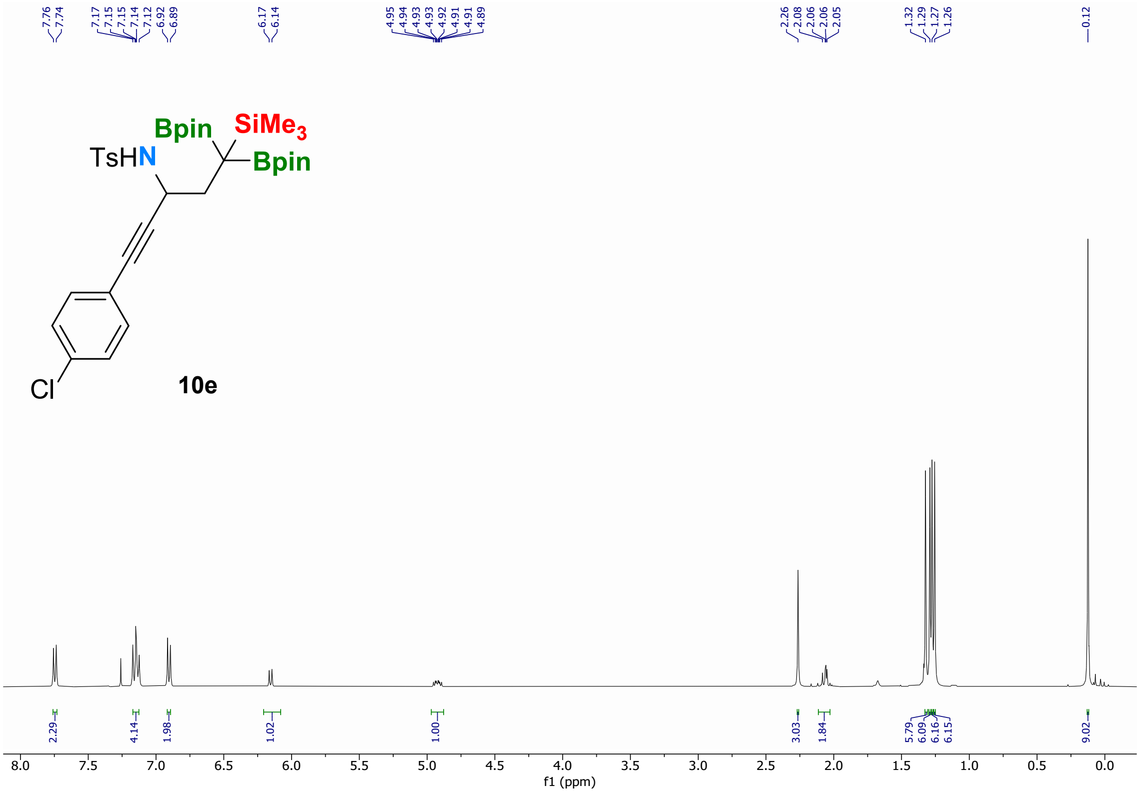

^{13}C NMR {^{1}H} (125 MHz, $CDCl_3$)

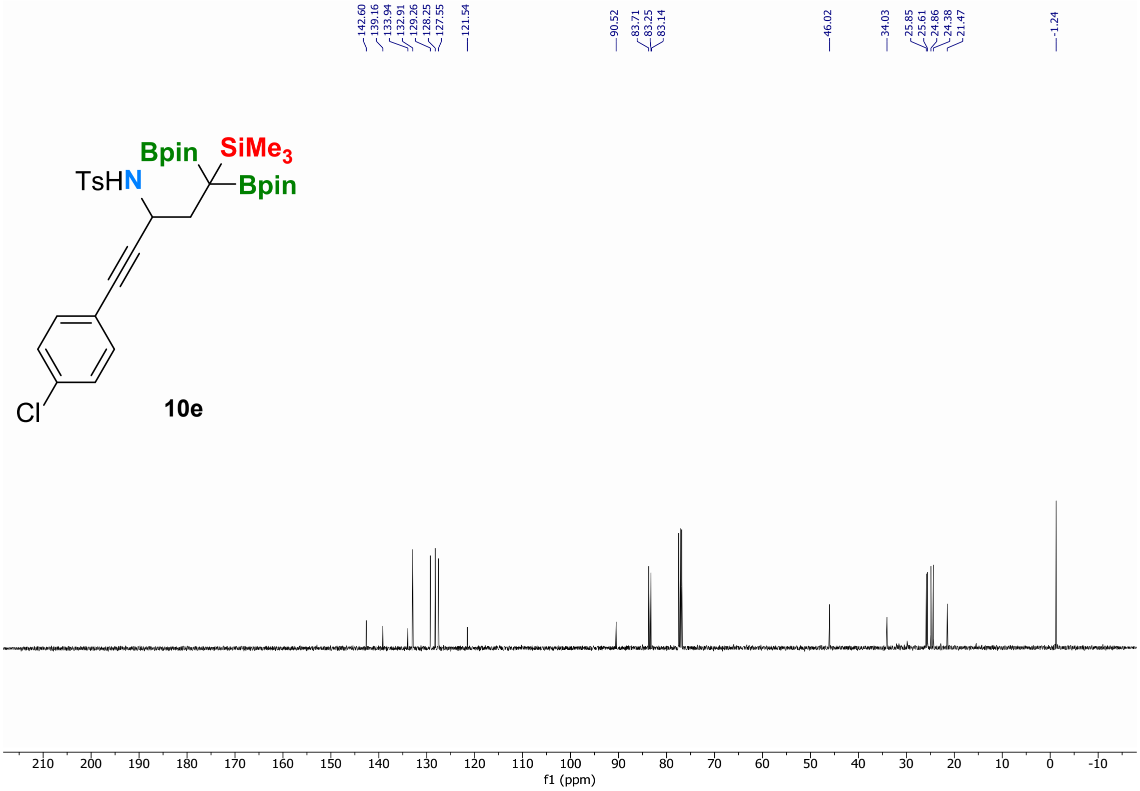

^{11}B NMR (128.3 MHz, $CDCl_3$)

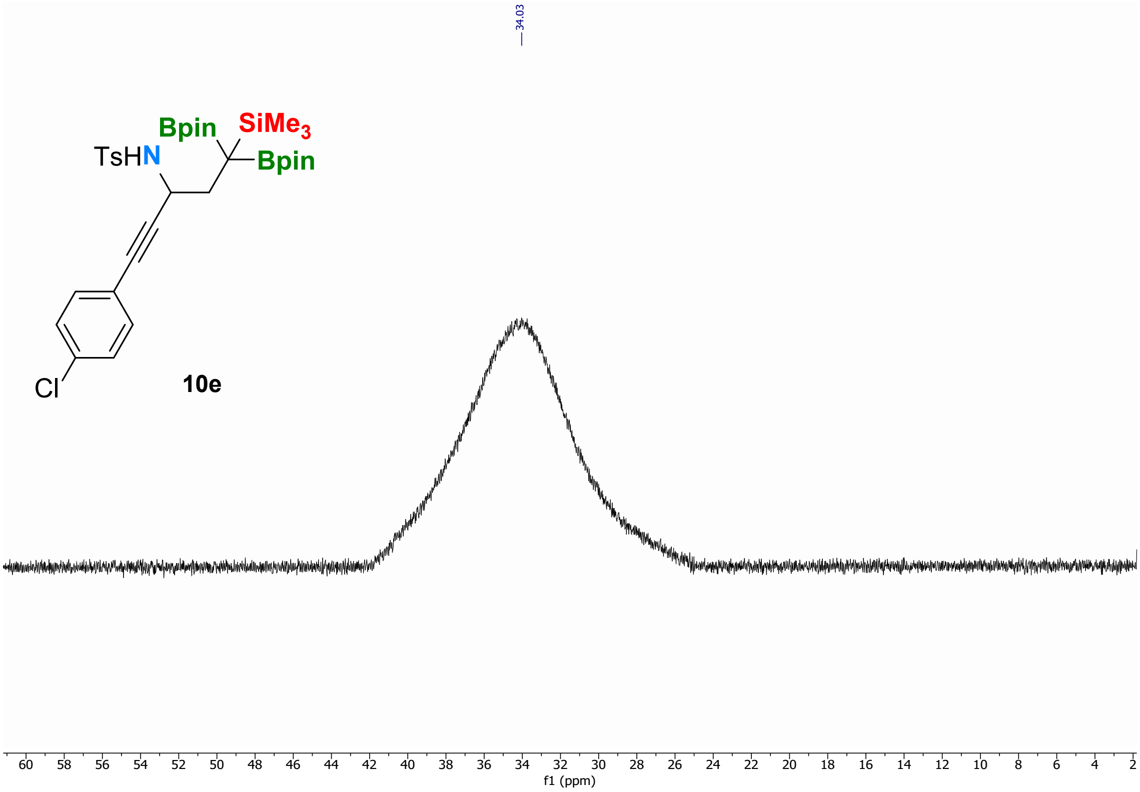

_N_-(2-(bis(4,4,5,5-tetramethyl-1,3,2-dioxaborolan-2-yl)methyl)-4-(naphthalen-2-yl)but-3-yn-1-yl)-4-methylbenzenesulfonamide (12a)

^{1}H NMR (400 MHz, $CDCl_3$)

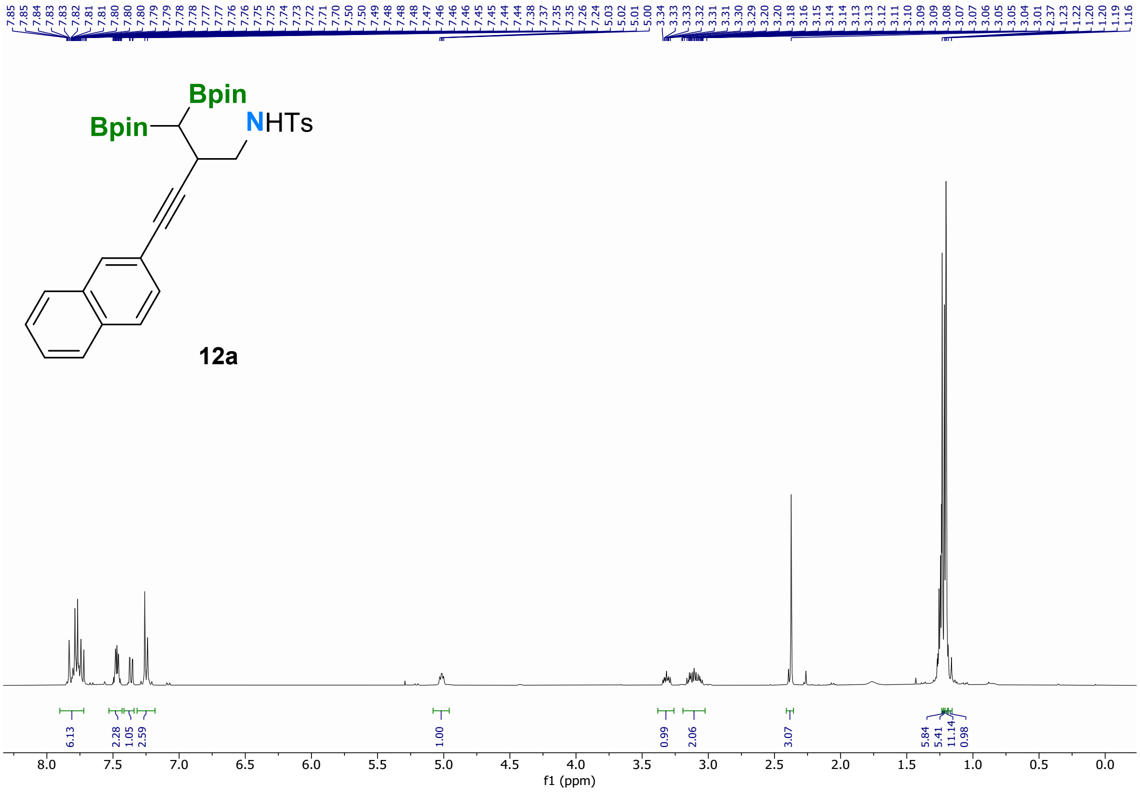

^{13}C NMR {[1]H} (125 MHz, $CDCl_3$)

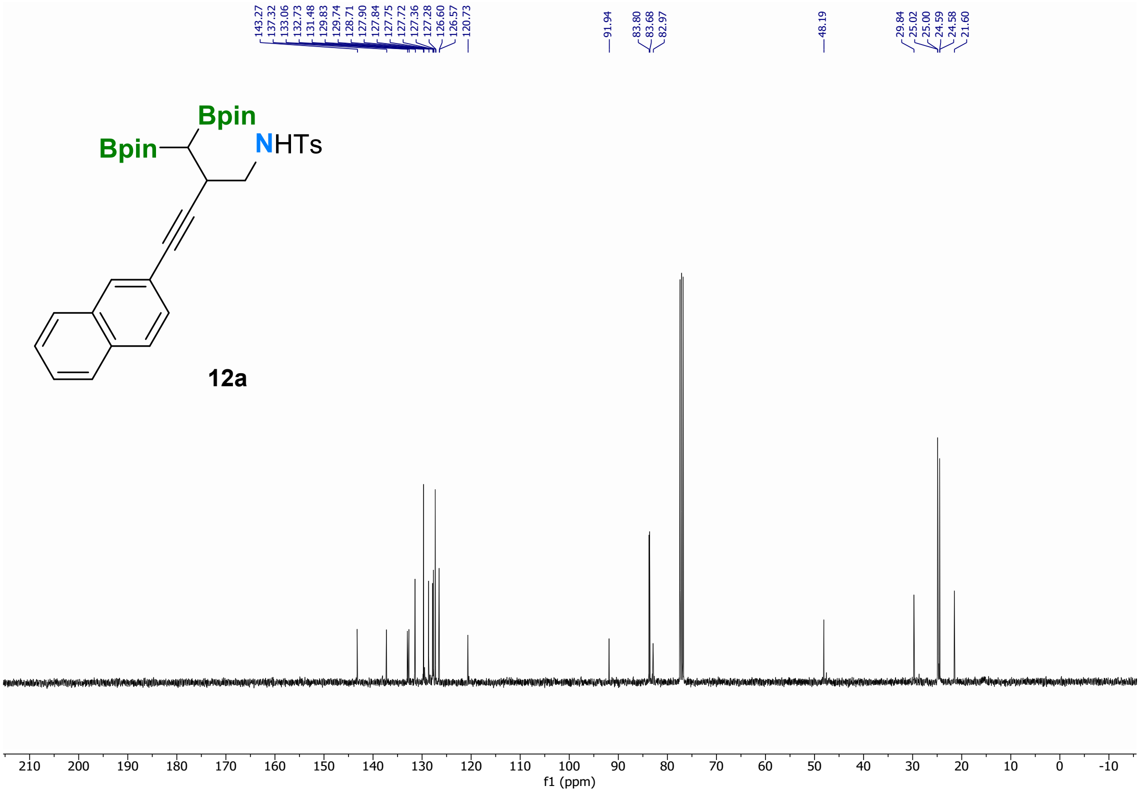

^{11}B NMR (128.3 MHz, CDCl$_3$)

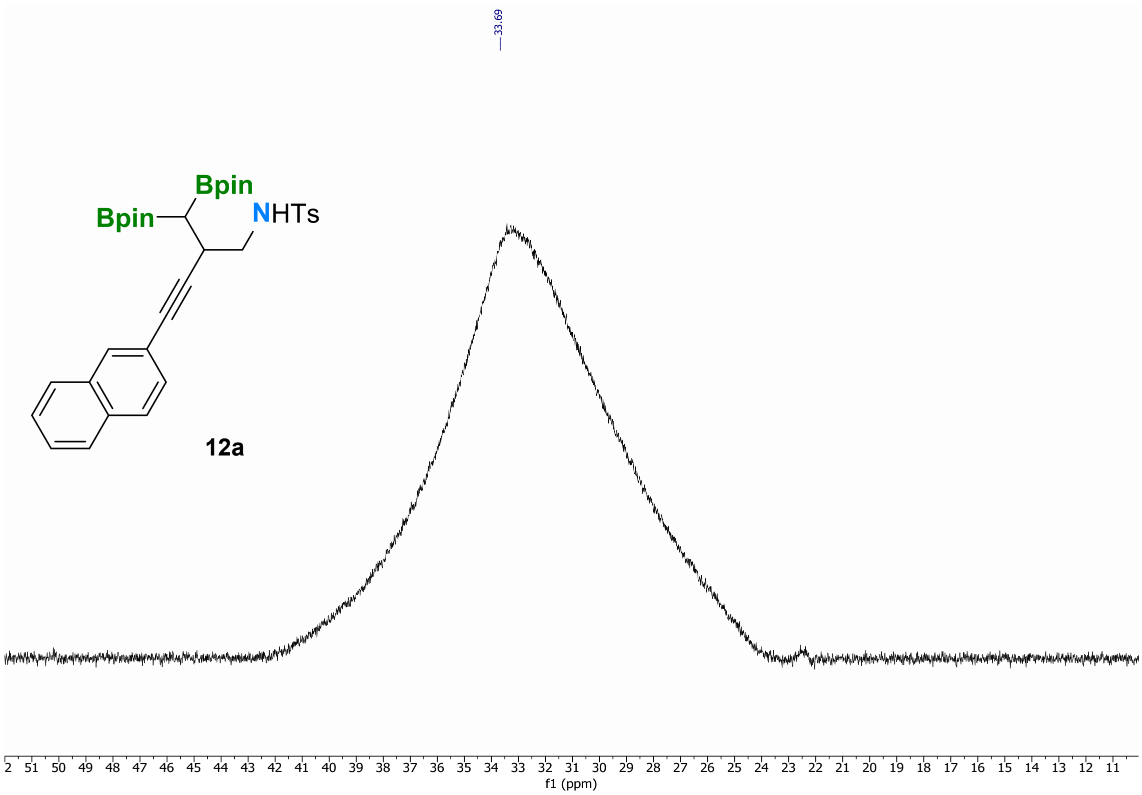

***N*-(2-(1,1-bis(4,4,5,5-tetramethyl-1,3,2-dioxaborolan-2-yl)ethyl)-4-(naphthalen-2-yl)but-3-yn-1-yl)-4-methylbenzenesulfonamide (12b)**

^{1}H NMR (400 MHz, CDCl$_3$)

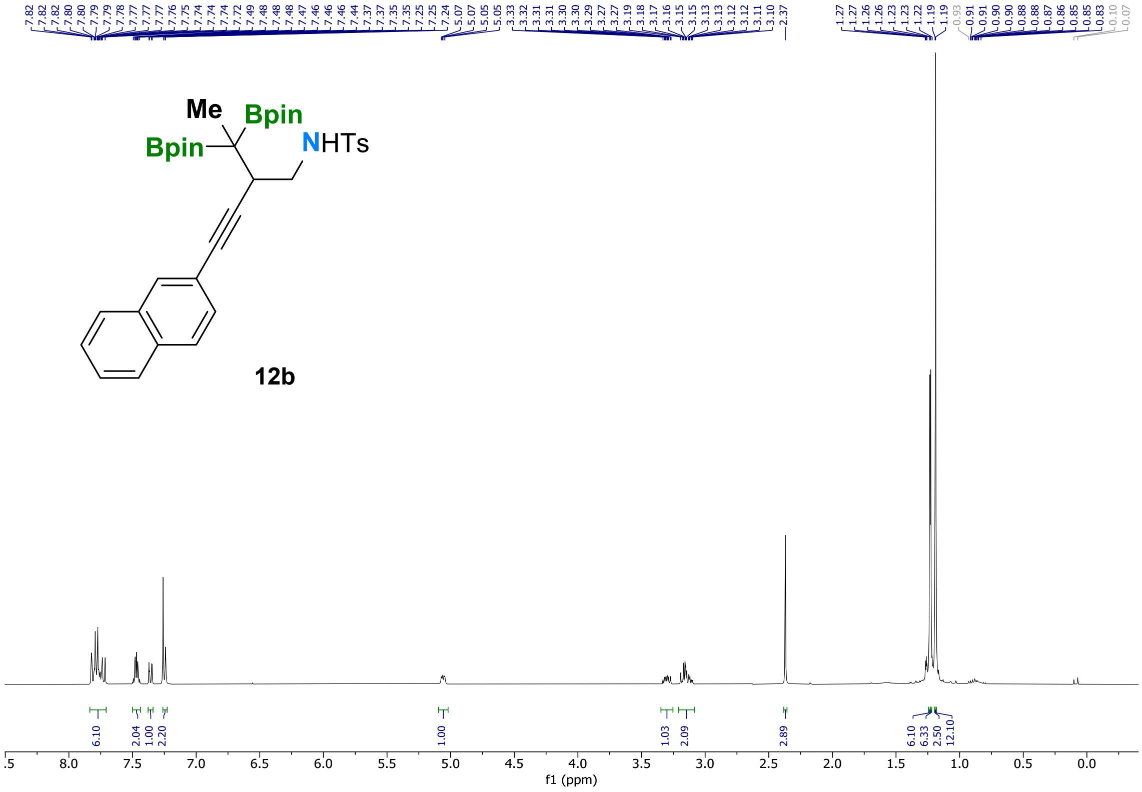

^{13}C NMR {^{1}H} (125 MHz, $CDCl_3$)

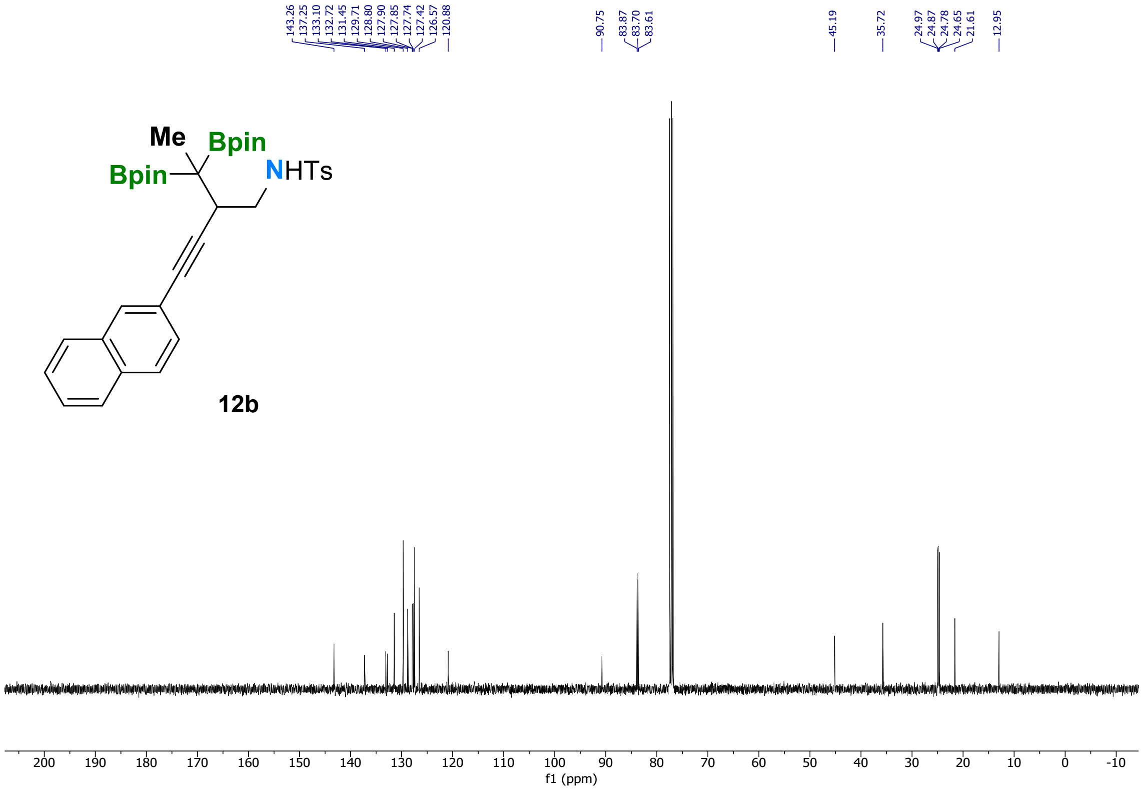

^{11}B NMR (128.3 MHz, $CDCl_3$)

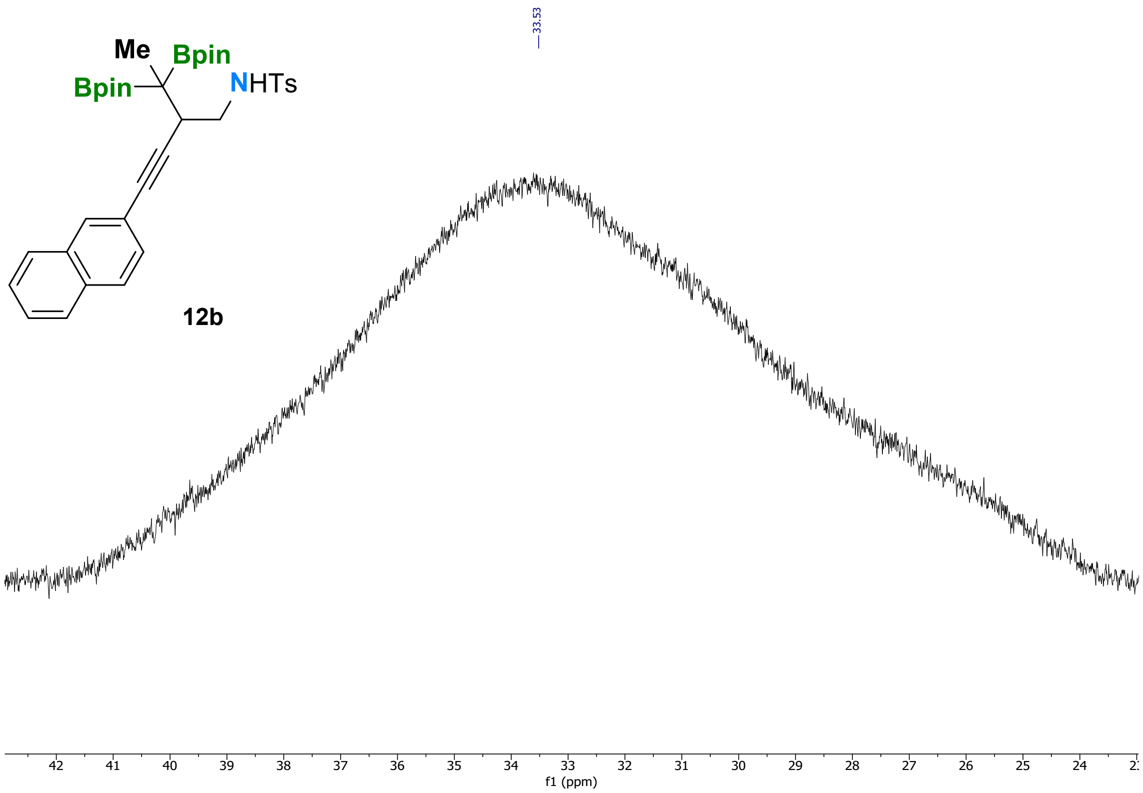

N-(2-(bis(4,4,5,5-tetramethyl-1,3,2-dioxaborolan-2-yl)(trimethylsilyl)methyl)-4-(naphthalen-2-yl)but-3-yn-1-yl)-4-methylbenzenesulfonamide (12e)

^{1}H NMR (400 MHz, $CDCl_3$)

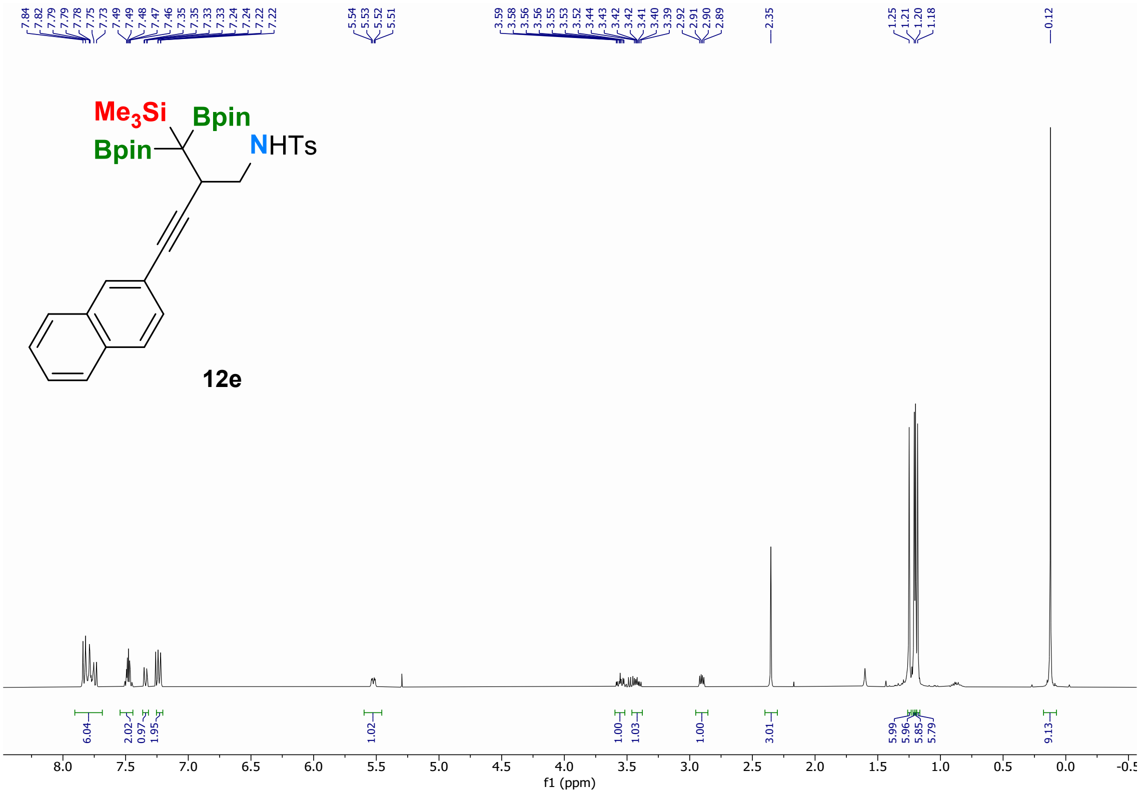

^{13}C NMR {^{1}H} (125 MHz, $CDCl_3$)

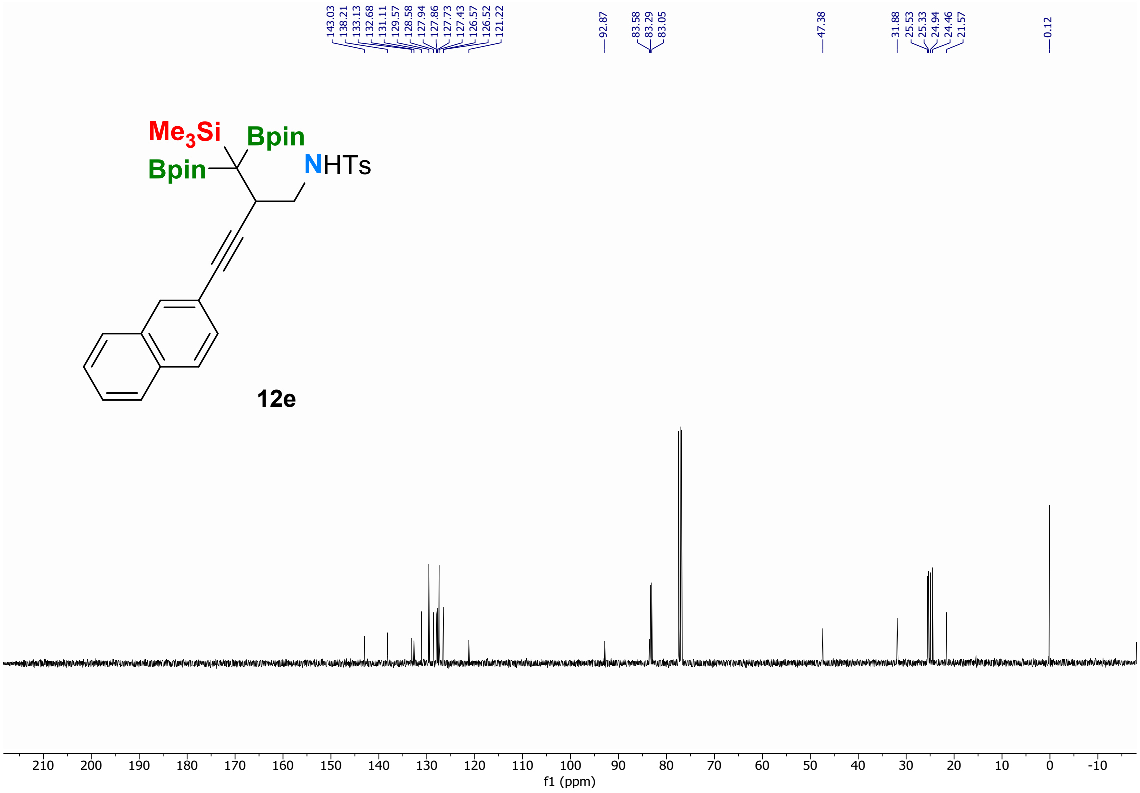

^{11}B NMR (128.3 MHz, CDCl$_3$)

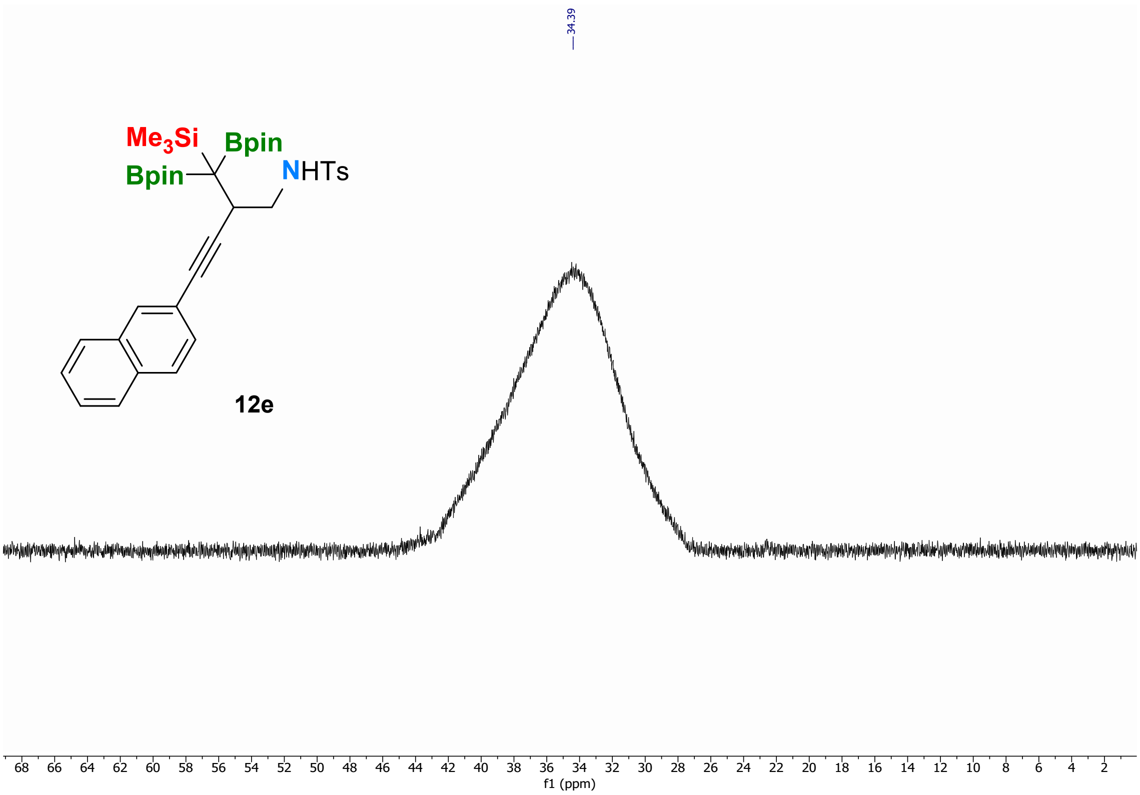

***N*-(2-(bis(4,4,5,5-tetramethyl-1,3,2-dioxaborolan-2-yl)methyl)-4-cyclohexylbut-3-yn-1-yl)-4-methylbenzenesulfonamide (15a)**

^{1}H NMR (400 MHz, CDCl$_3$)

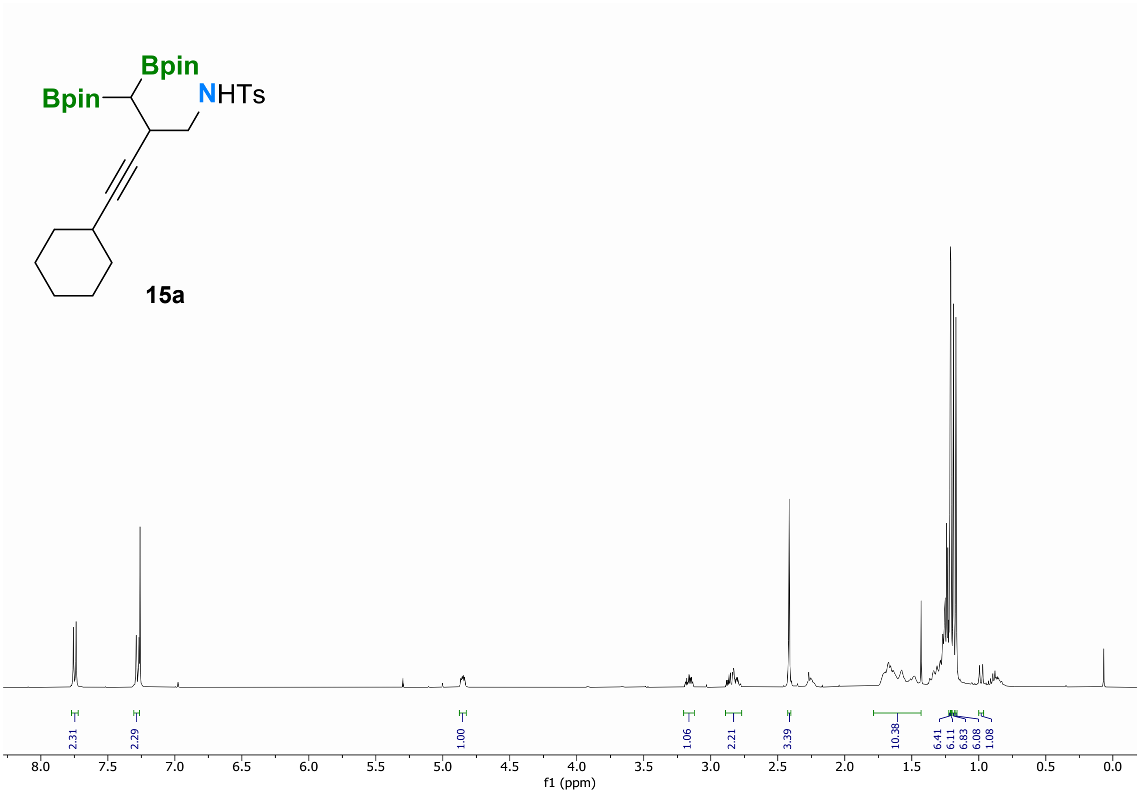

^{13}C NMR {^1H} (125 MHz, $CDCl_3$)

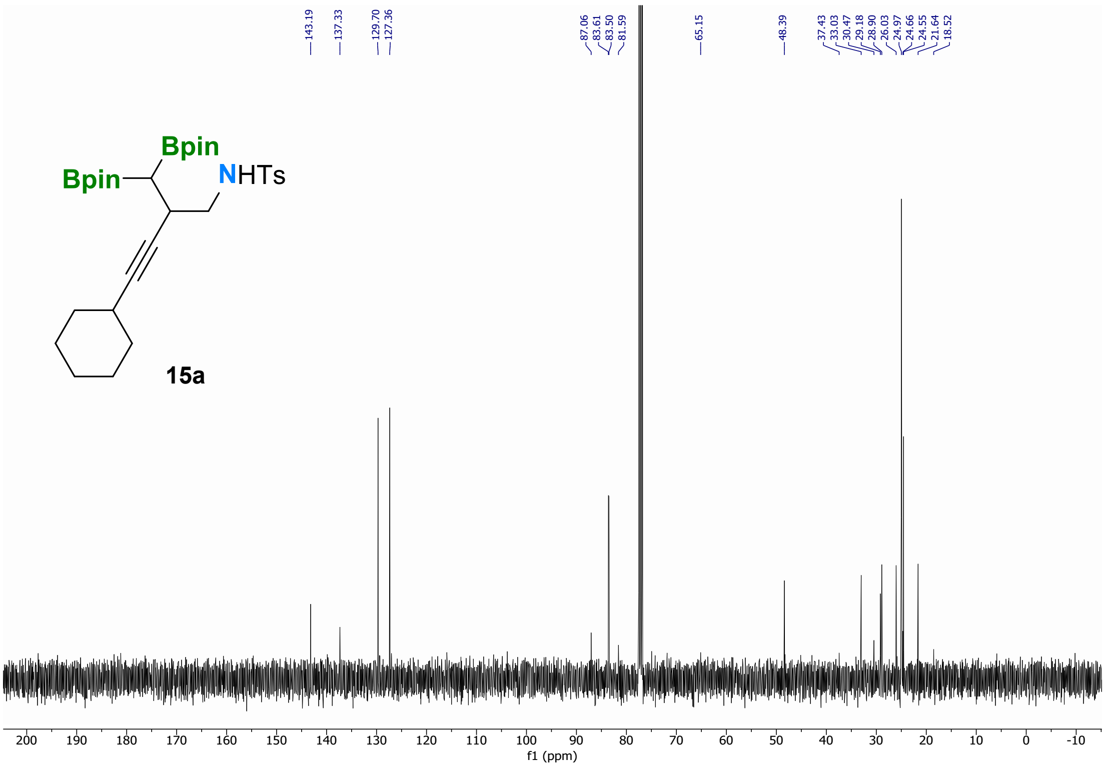

^{11}B NMR (128.3 MHz, $CDCl_3$)

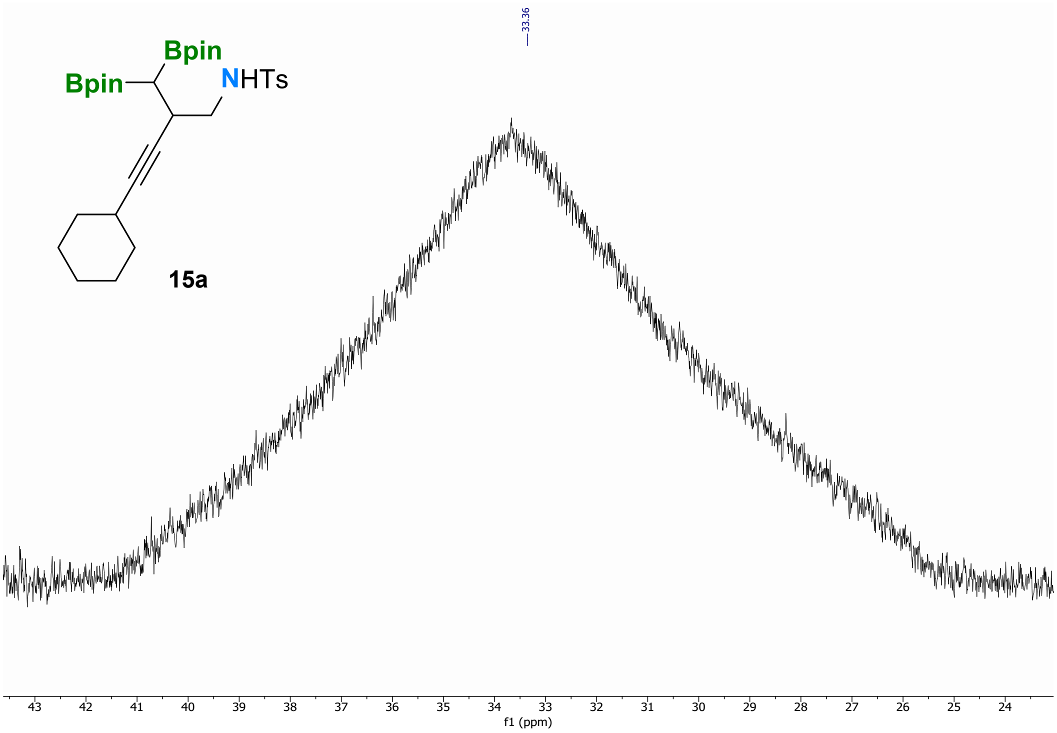

***N*-(2-(1,1-bis(4,4,5,5-tetramethyl-1,3,2-dioxaborolan-2-yl)ethyl)-4-cyclohexylbut-3-yn-1-yl)-4-methylbenzenesulfonamide (15b)**

^{1}H NMR (400 MHz, $CDCl_3$)

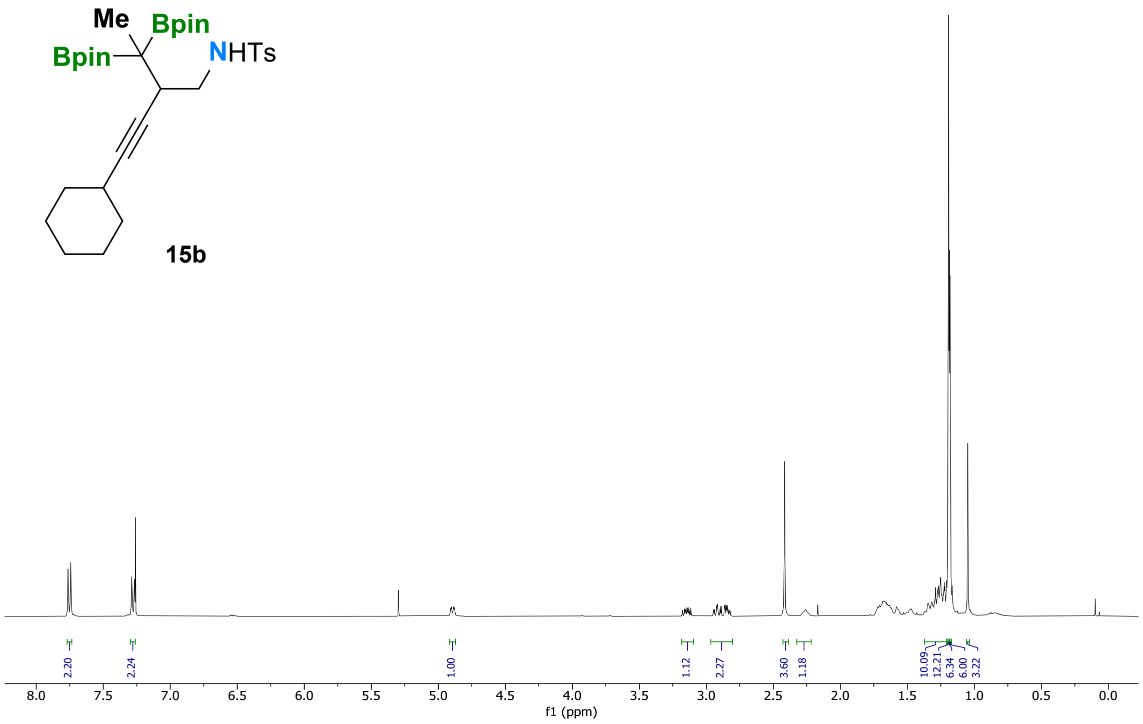

^{13}C NMR {[1]H} (125 MHz, $CDCl_3$)

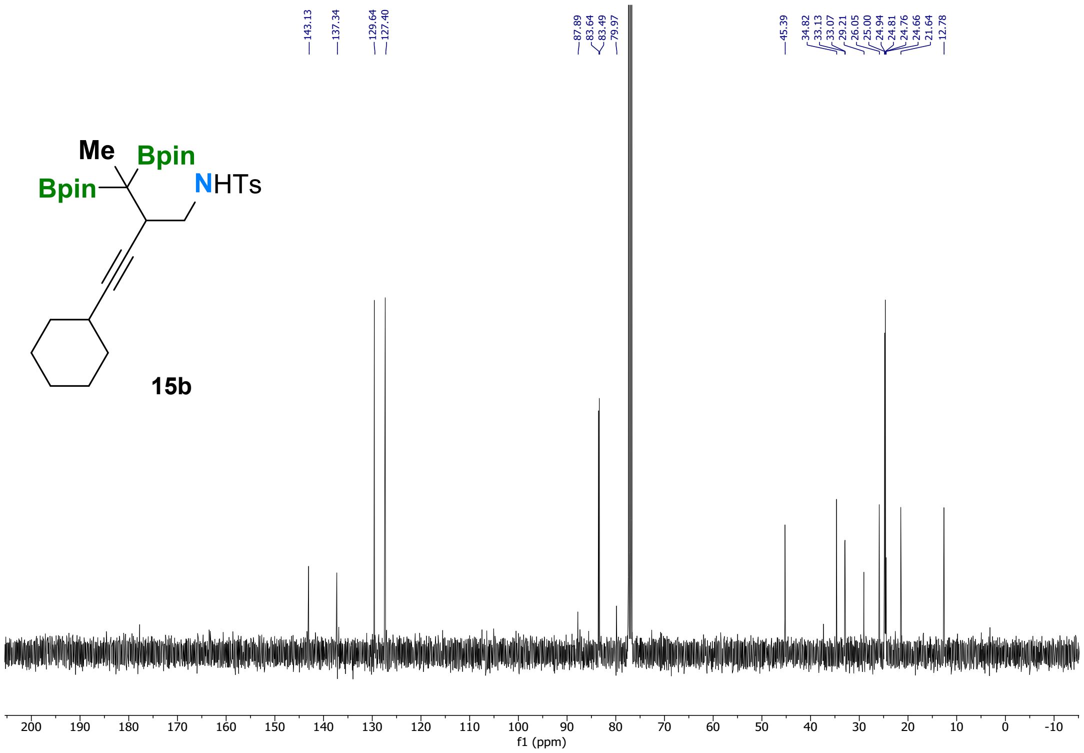

^{11}B NMR (128.3 MHz, $CDCl_3$)

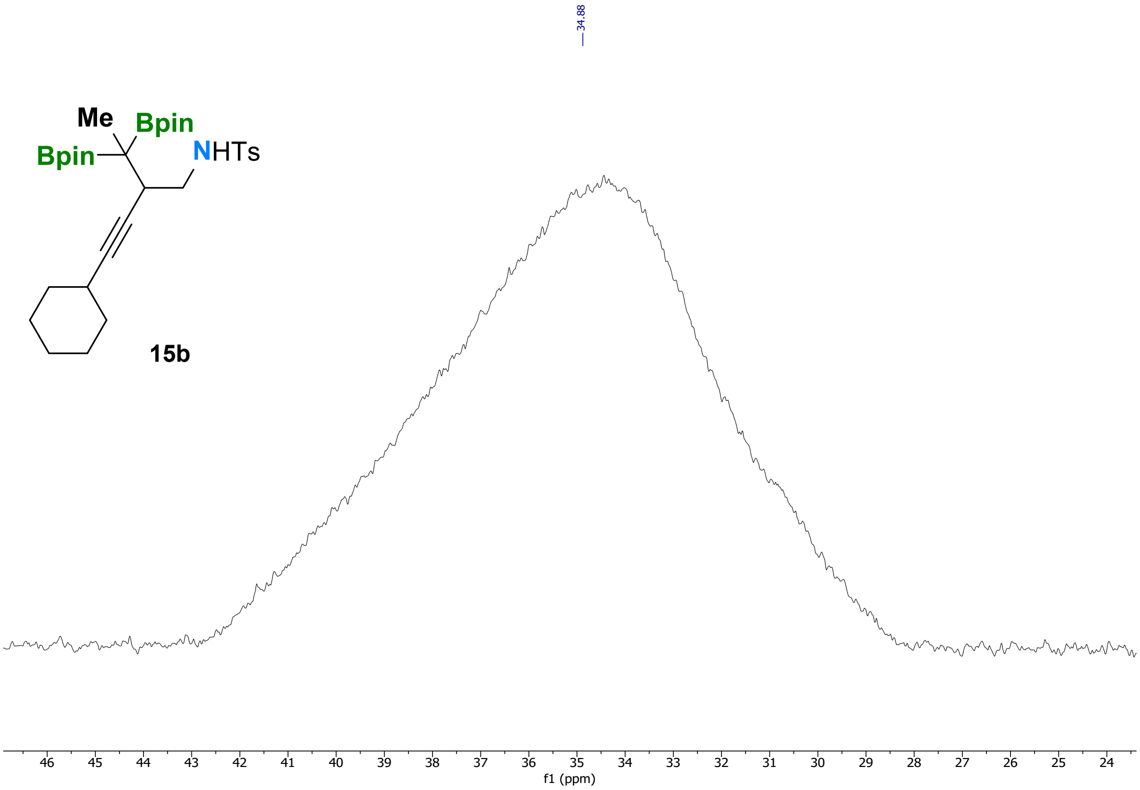

***N*-(2-(bis(4,4,5,5-tetramethyl-1,3,2-dioxaborolan-2-yl)(trimethylsilyl)methyl)-4-cyclohexylbut-3-yn-1-yl)-4-methylbenzenesulfonamide (15e)**

^{1}H NMR (400 MHz, $CDCl_3$)

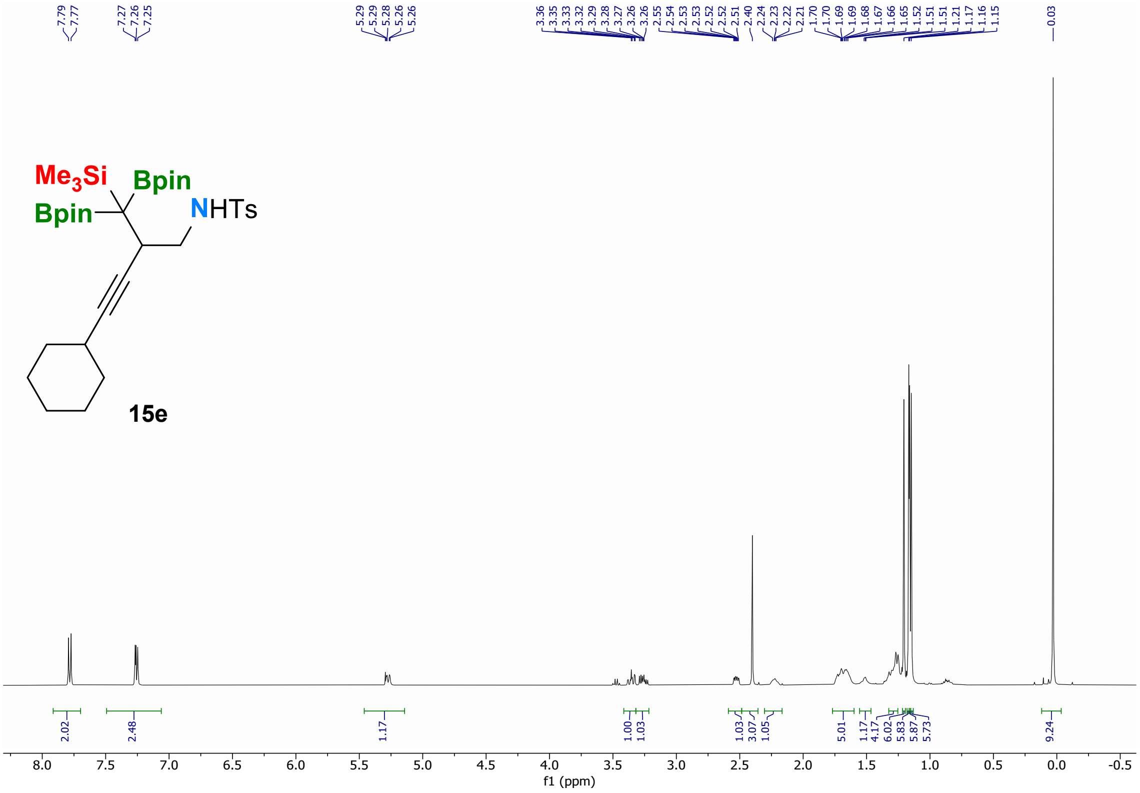

^{13}C NMR {^{1}H} (125 MHz, $CDCl_3$)

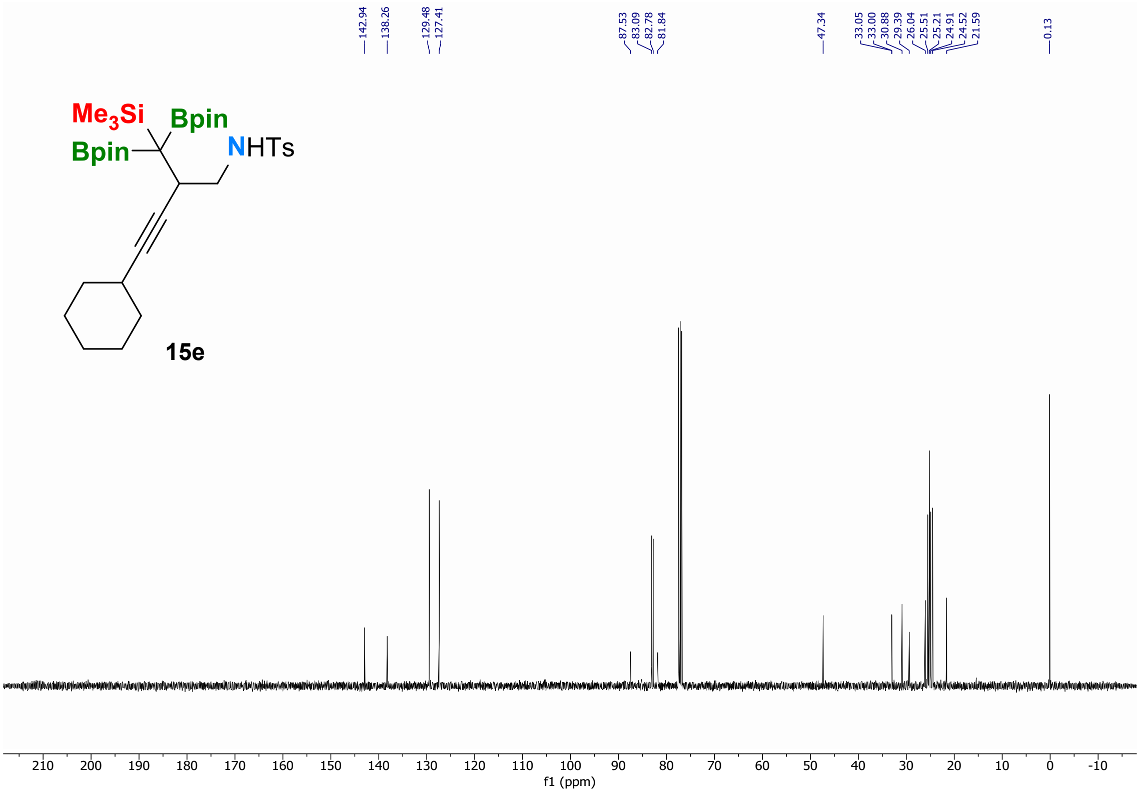

^{11}B NMR (128.3 MHz, $CDCl_3$)

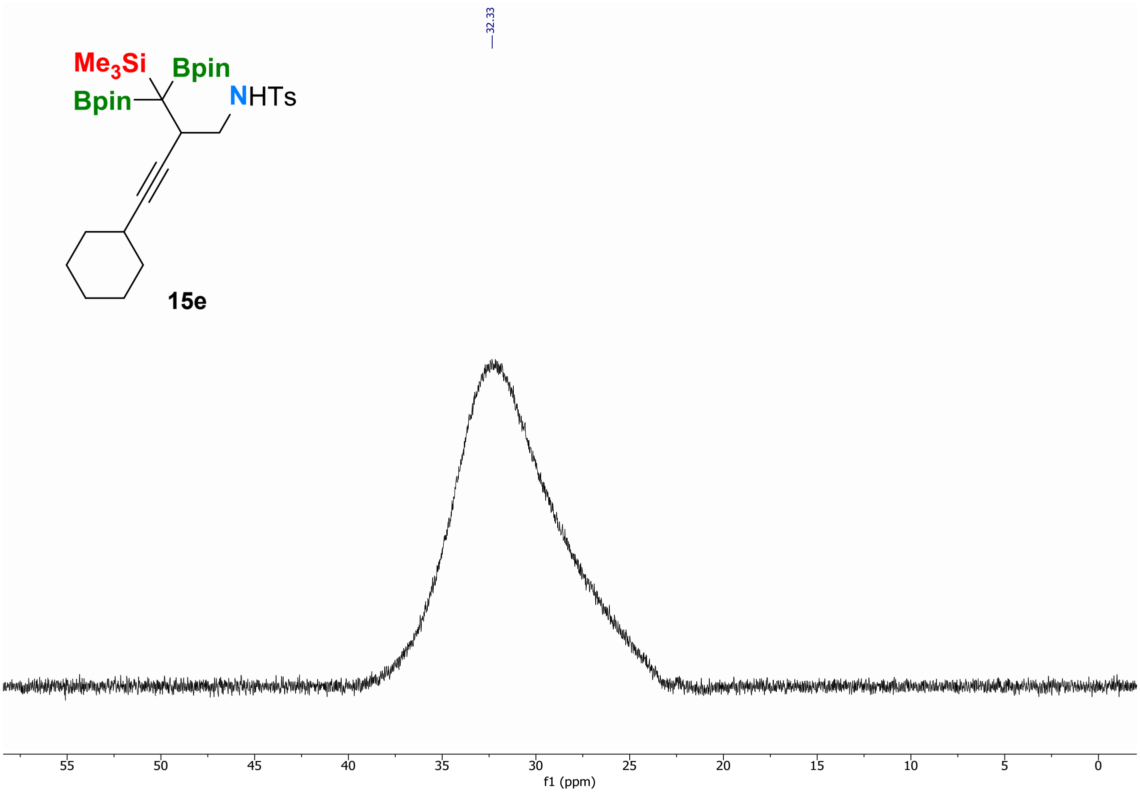

N-(1-cyclohexyl-5,5-bis(4,4,5,5-tetramethyl-1,3,2-dioxaborolan-2-yl)-5-(trimethylsilyl)pent-1-yn-3-yl)-4-methylbenzenesulfonamide (16e)

^{1}H NMR (400 MHz, $CDCl_3$)

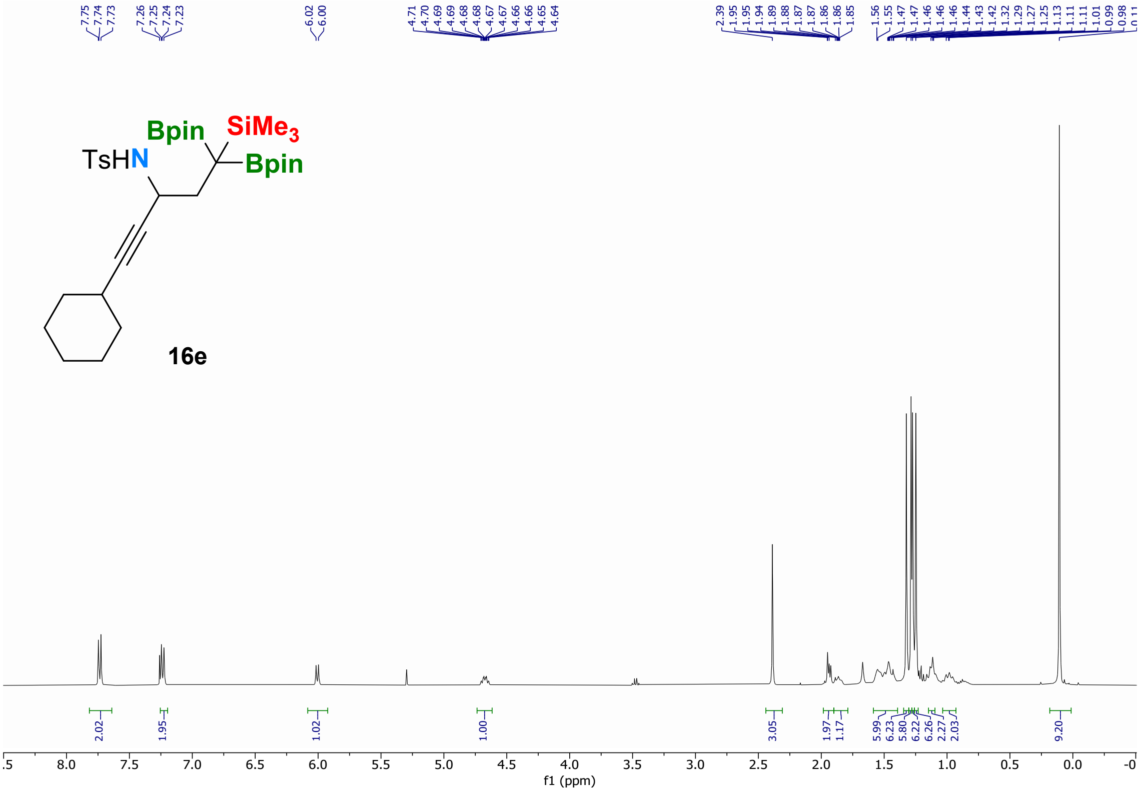

^{13}C NMR {^{1}H} (125 MHz, $CDCl_3$)

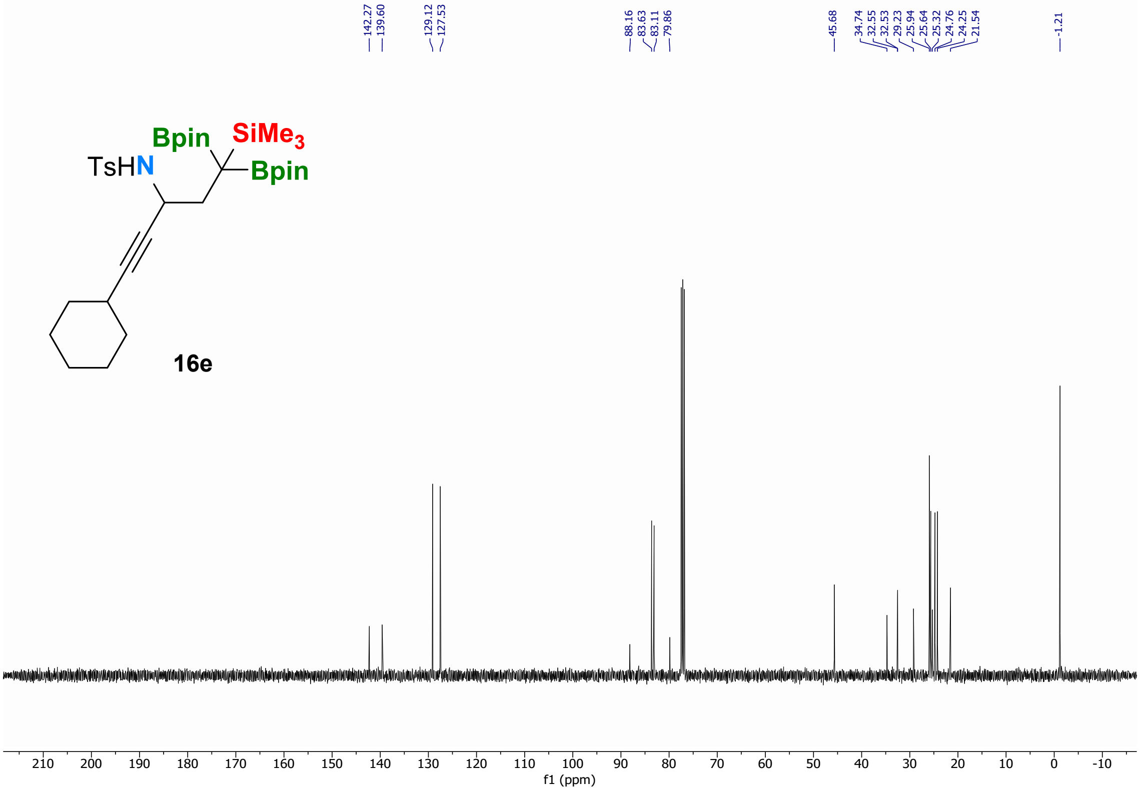

^{11}B NMR (128.3 MHz, $CDCl_3$)

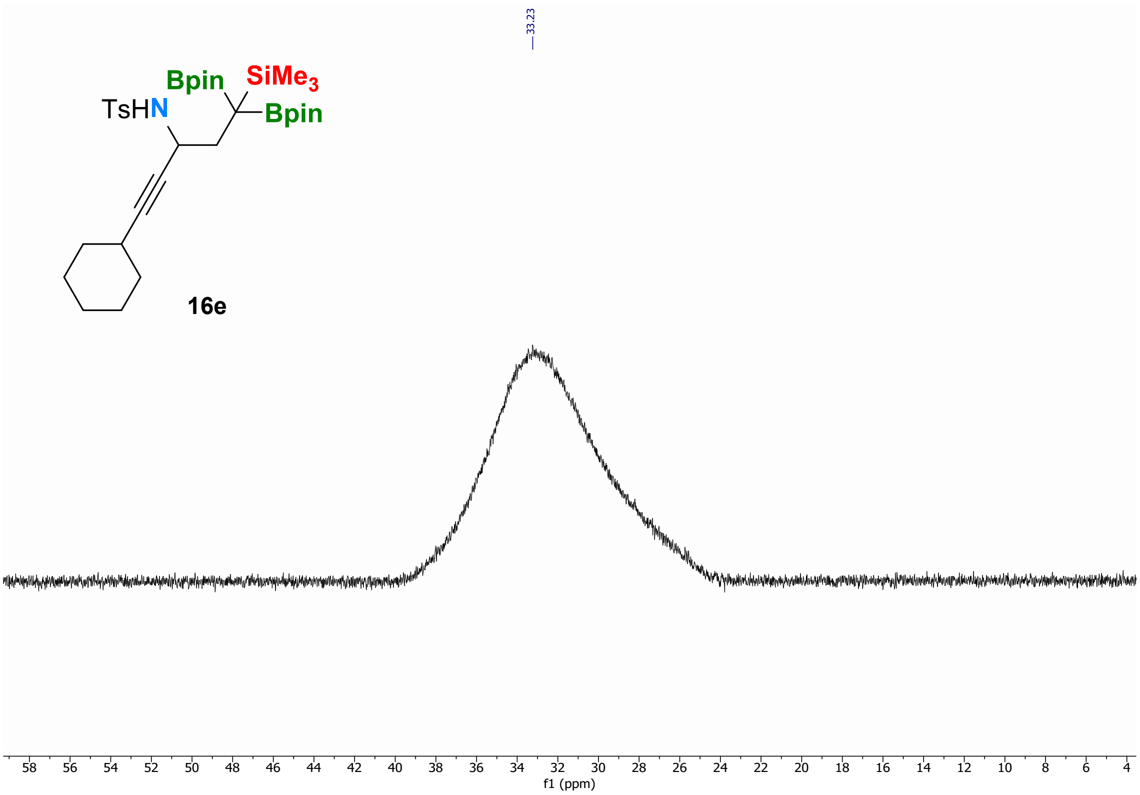

N-(2-(bis(4,4,5,5-tetramethyl-1,3,2-dioxaborolan-2-yl)methyl)dec-3-yn-1-yl)-4-methylbenzenesulfonamide (18a)

^{1}H NMR (400 MHz, $CDCl_3$)

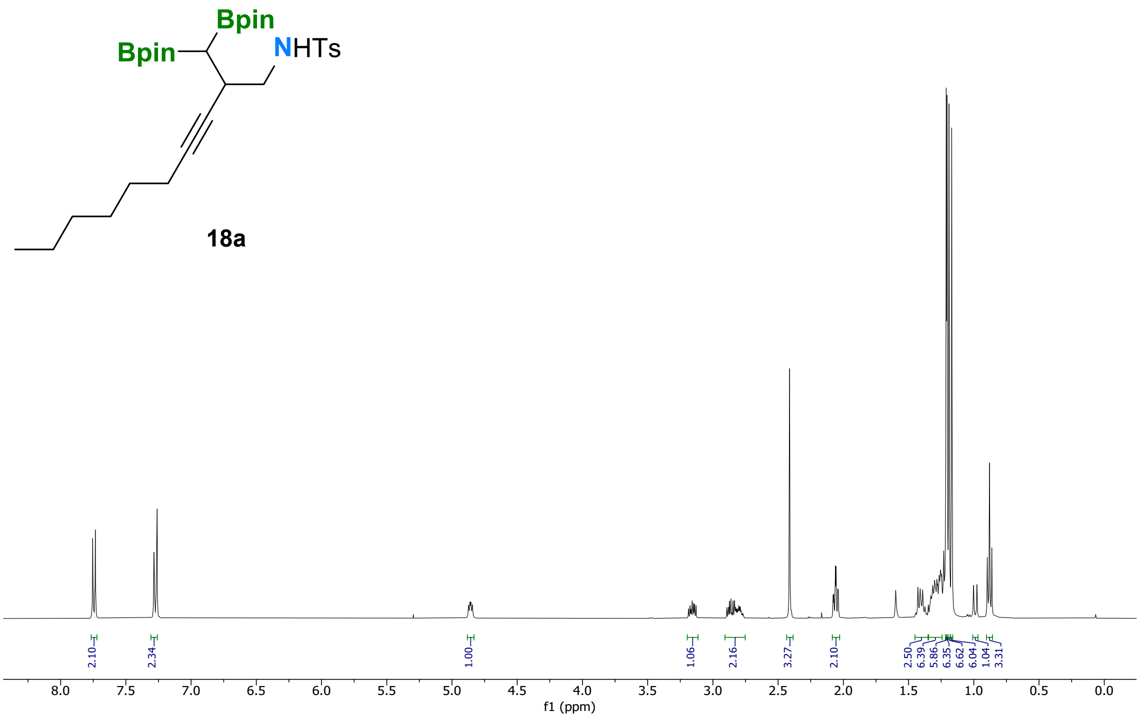

^{13}C NMR {^{1}H} (125 MHz, $CDCl_3$)

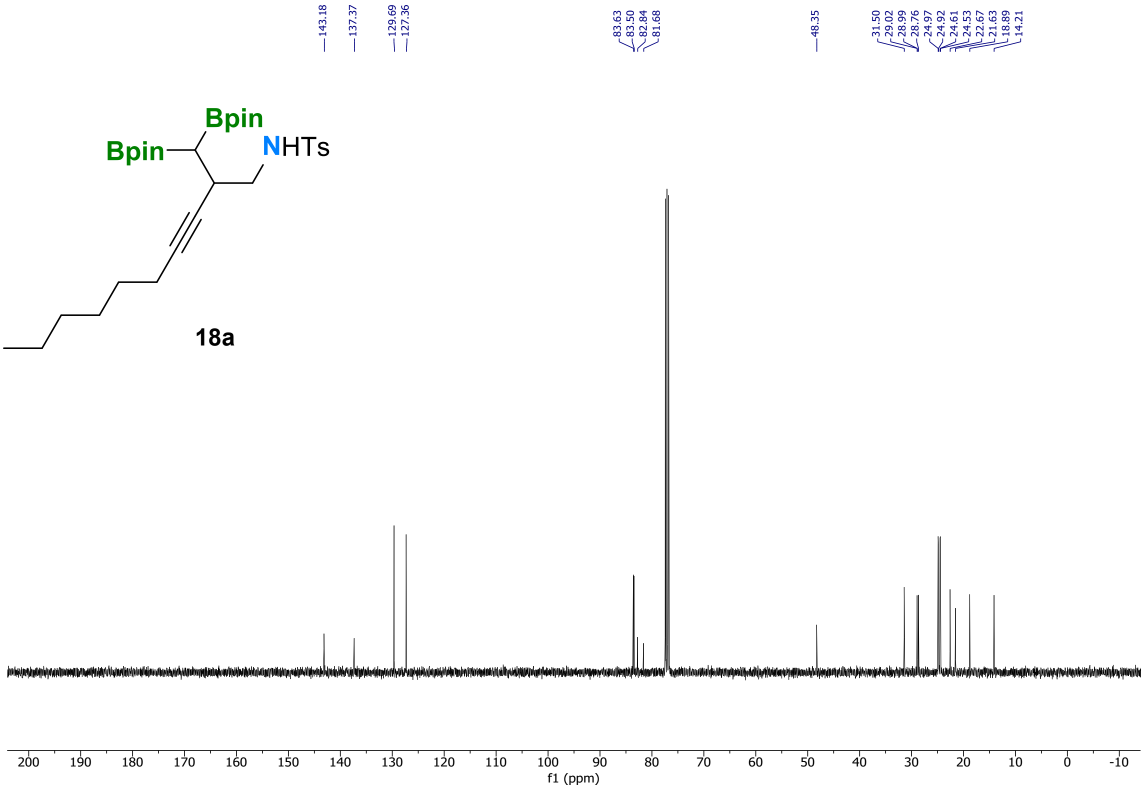

^{11}B NMR (128.3 MHz, $CDCl_3$)

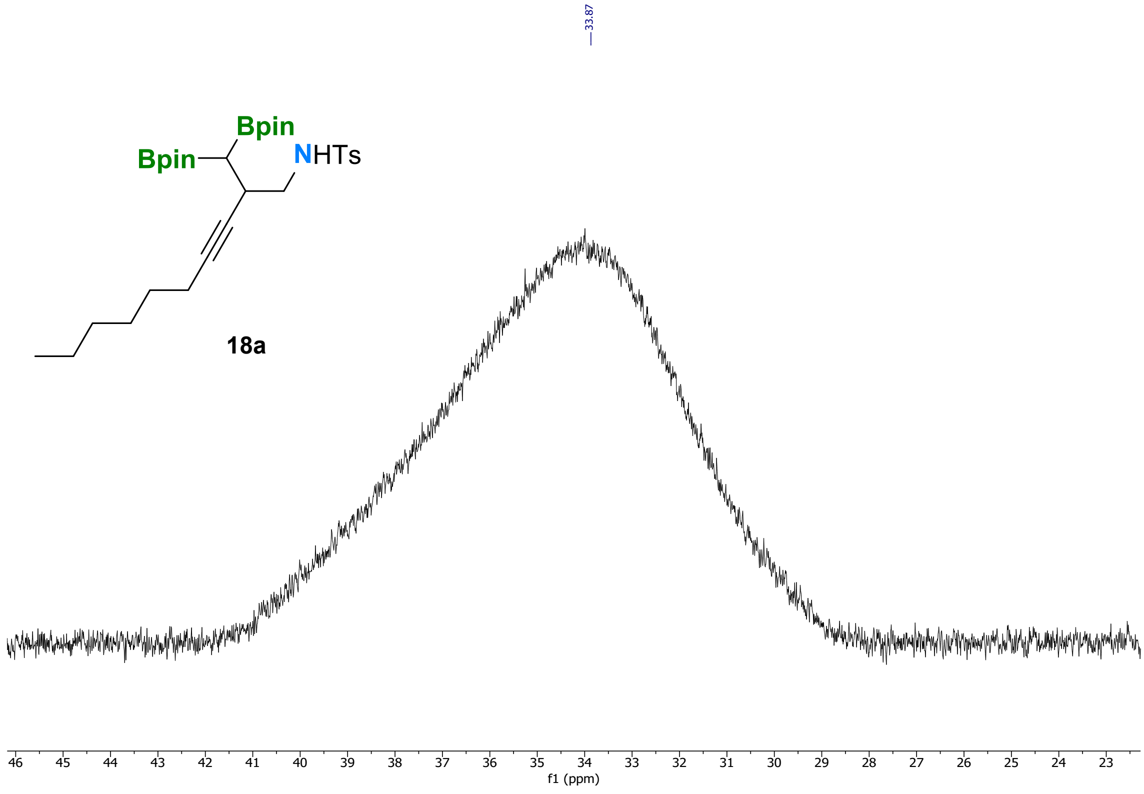

_N_-(2-(1,1-bis(4,4,5,5-tetramethyl-1,3,2-dioxaborolan-2-yl)ethyl)dec-3-yn-1-yl)-4-methylbenzenesulfonamide (18b)

^{1}H NMR (400 MHz, $CDCl_3$)

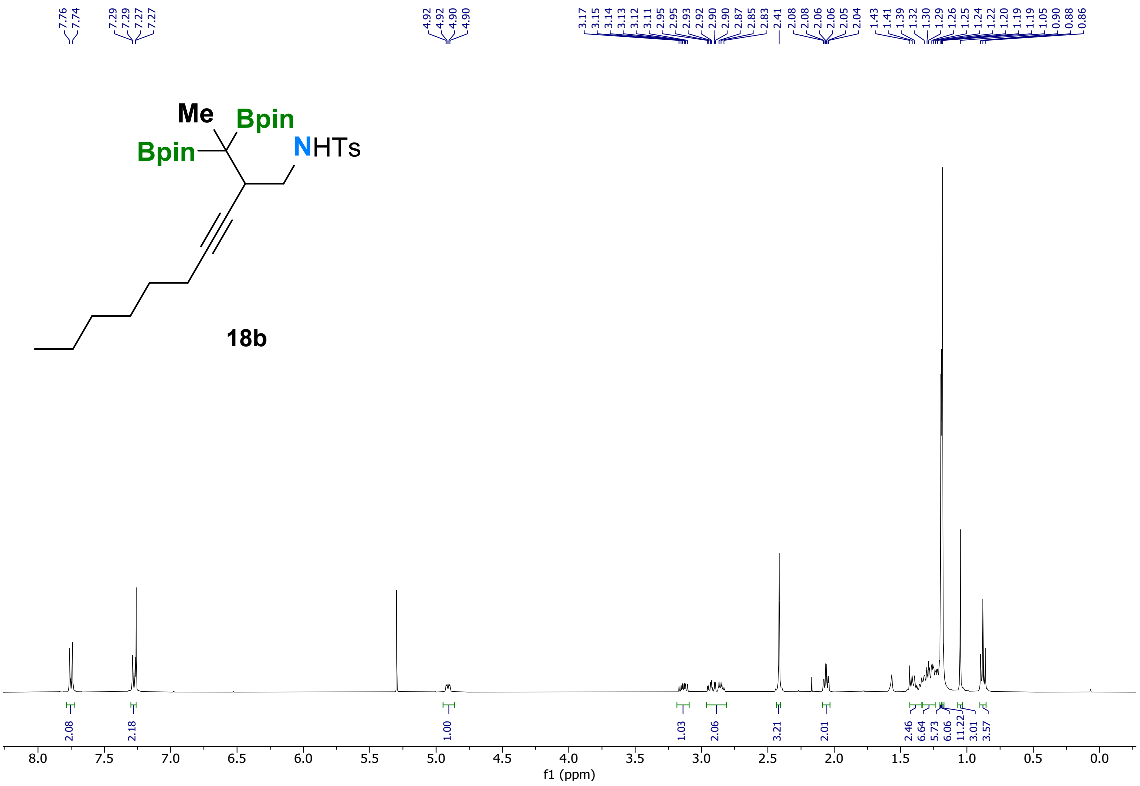

^{13}C NMR {^{1}H} (125 MHz, $CDCl_3$)

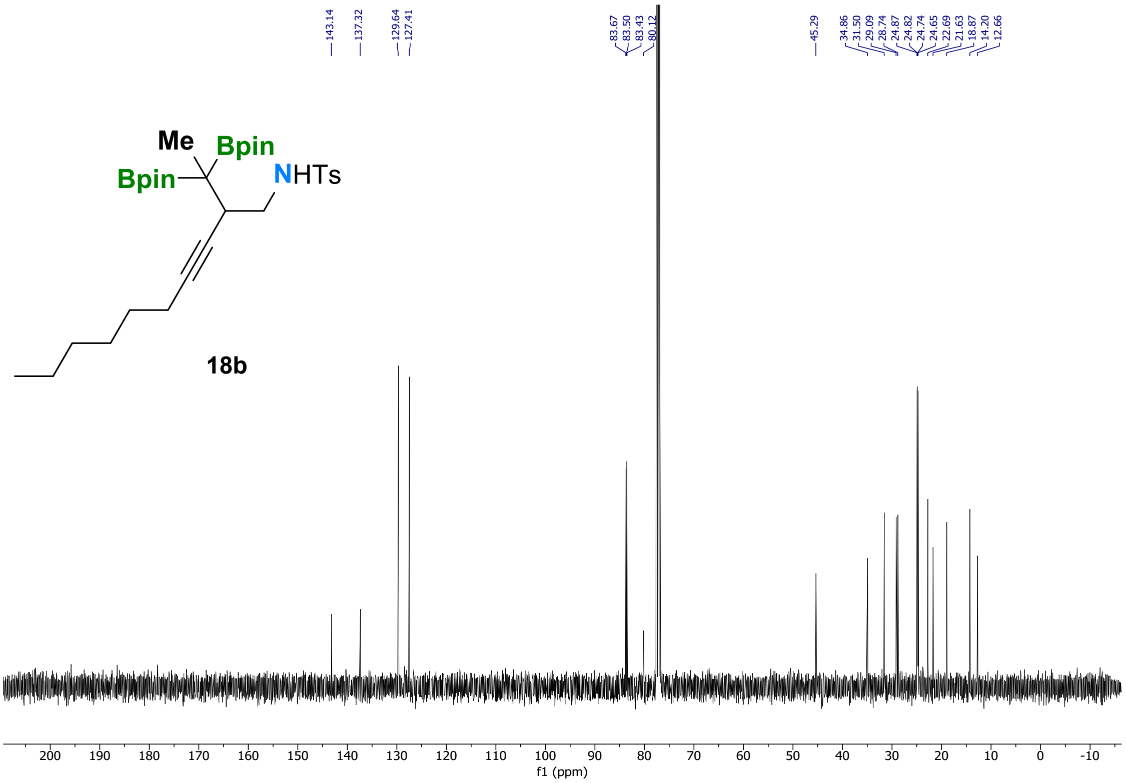

^{11}B NMR (128.3 MHz, $CDCl_3$)

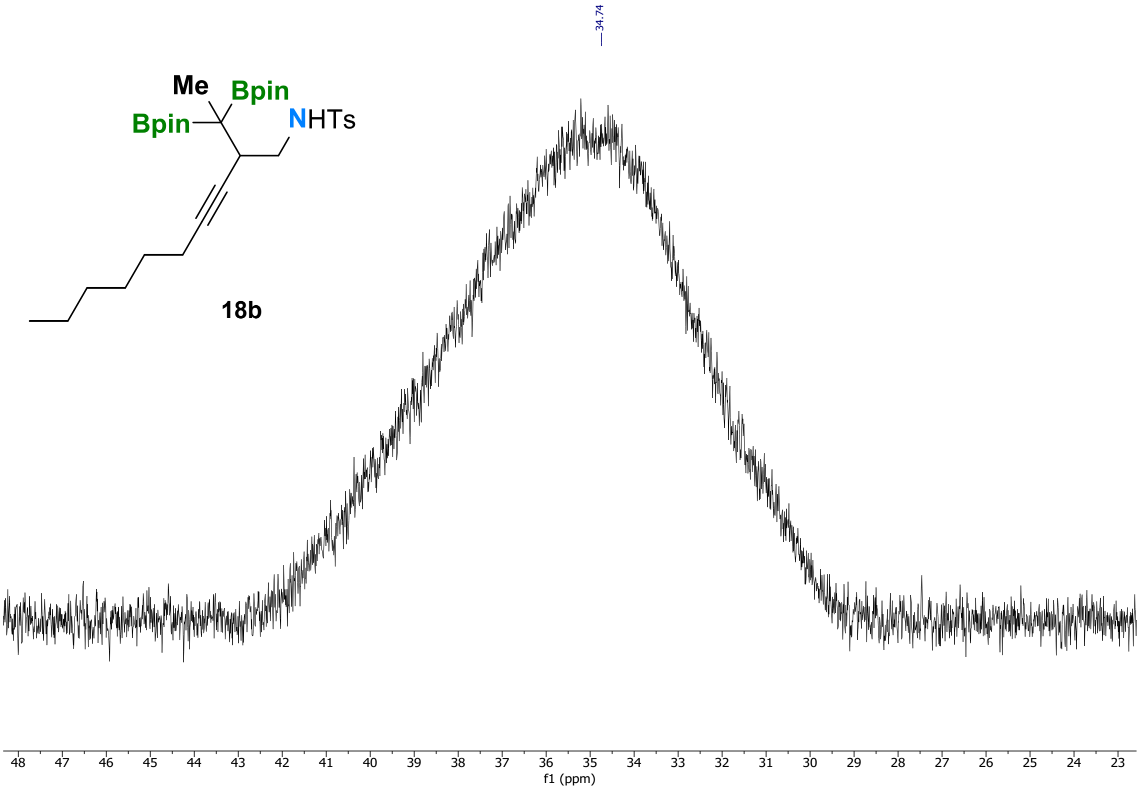

***N*-(2-(bis(4,4,5,5-tetramethyl-1,3,2-dioxaborolan-2-yl)(trimethylsilyl)methyl)dec-3-yn-1-yl)-4-methylbenzenesulfonamide (18e)**

^{1}H NMR (400 MHz, $CDCl_3$)

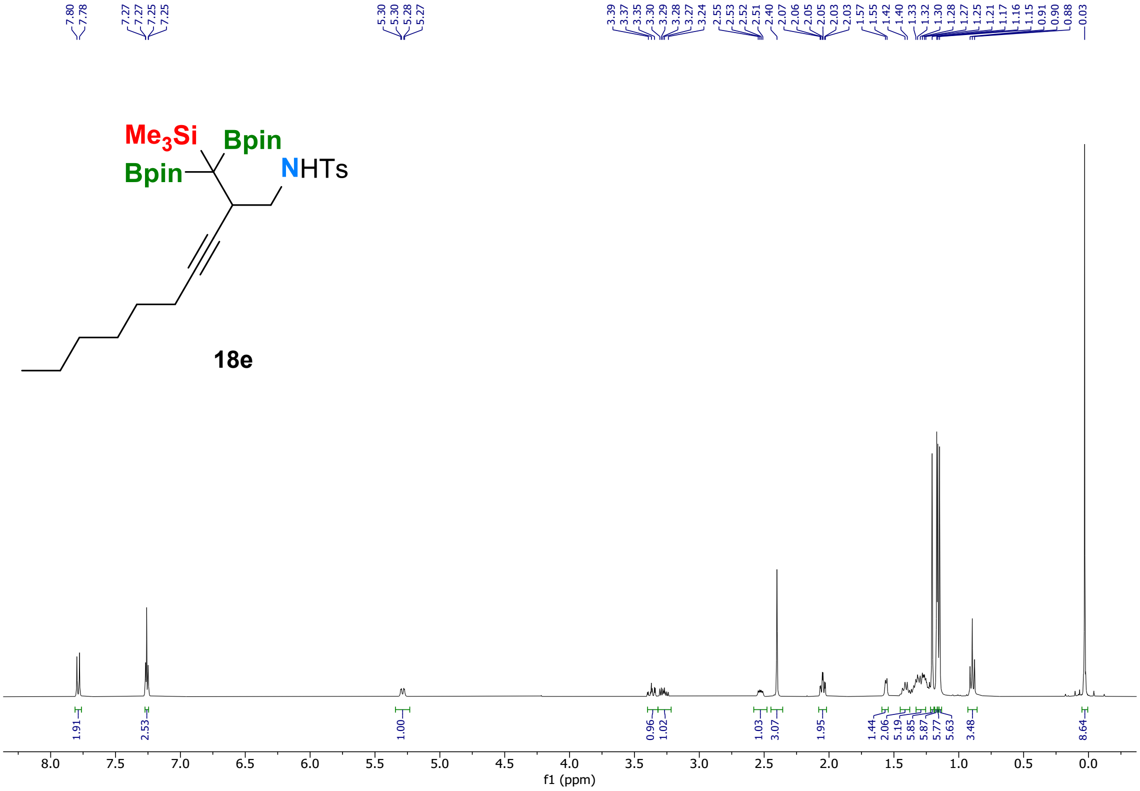

^{13}C NMR {^{1}H} (125 MHz, $CDCl_3$)

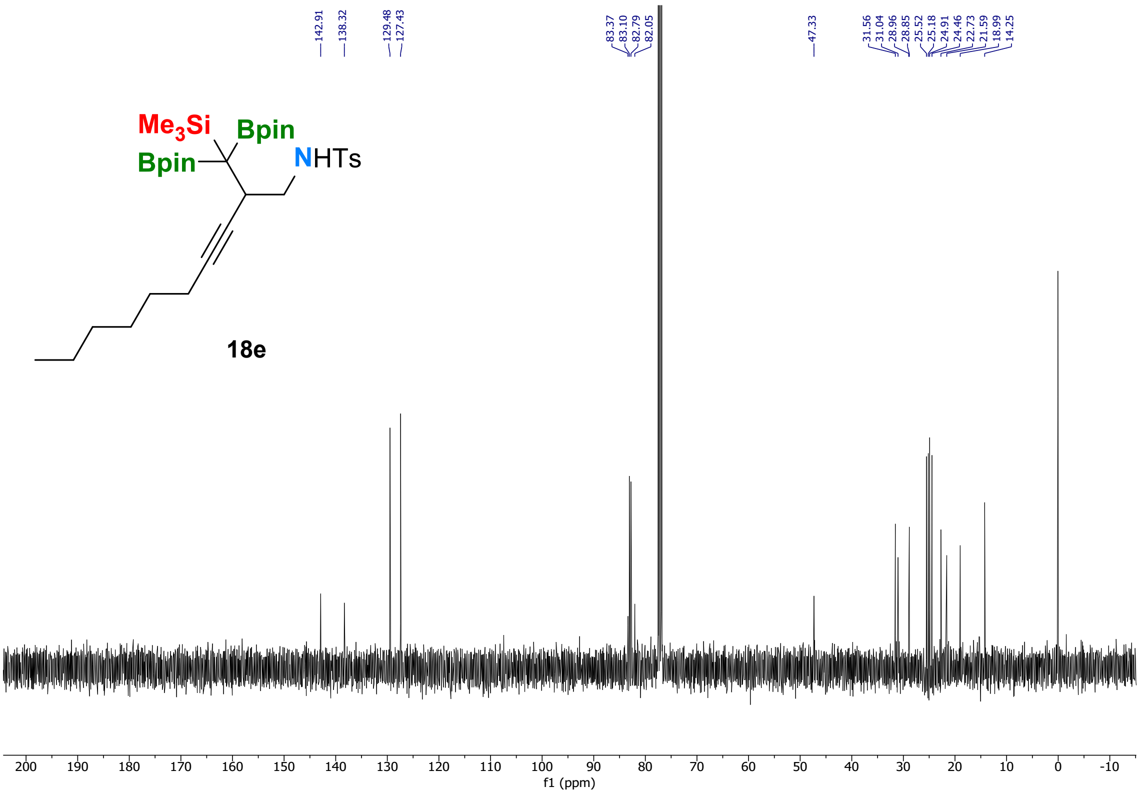

^{11}B NMR (128.3 MHz, $CDCl_3$)

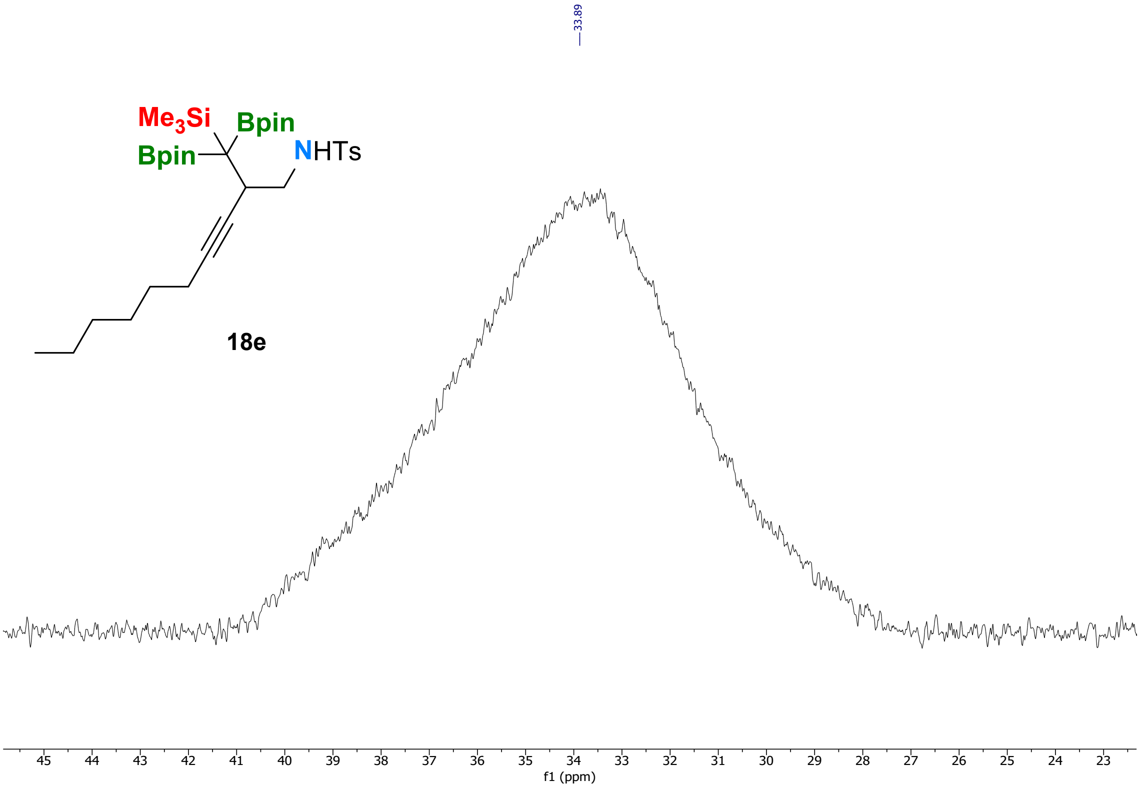

***N*-(1,1-bis(4,4,5,5-tetramethyl-1,3,2-dioxaborolan-2-yl)-1-(trimethylsilyl)undec-4-yn-3-yl)-4-methylbenzenesulfonamide (19e)**

^{1}H NMR (400 MHz, $CDCl_3$)

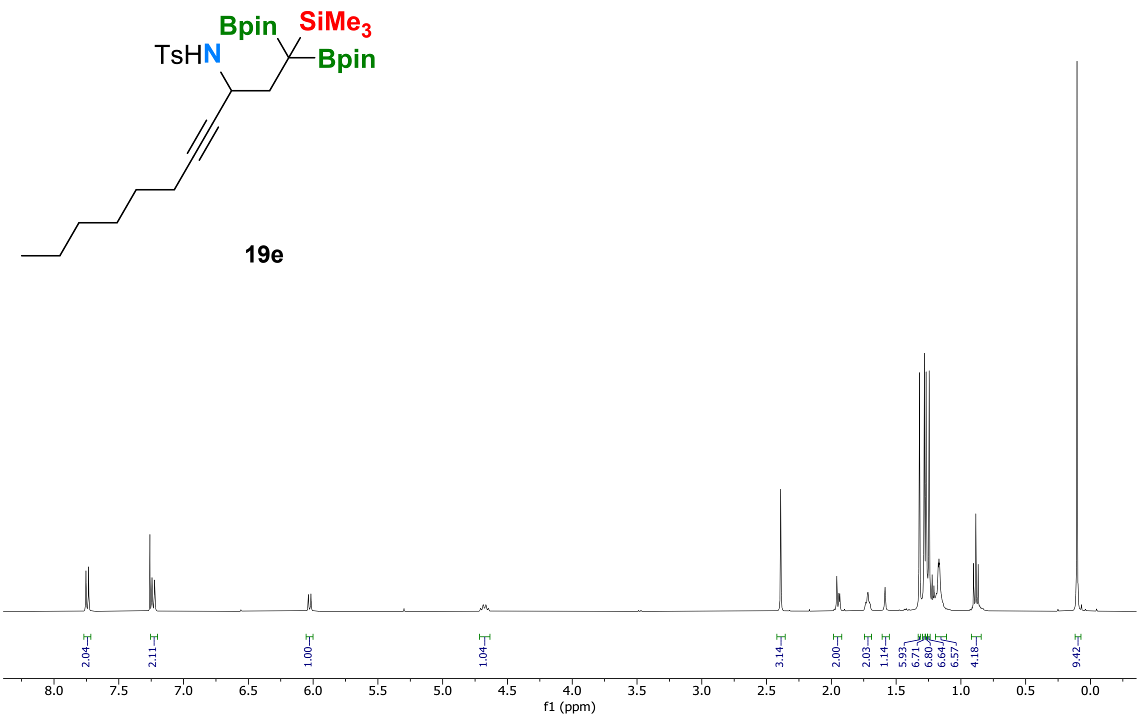

^{13}C NMR {^{1}H} (125 MHz, $CDCl_3$)

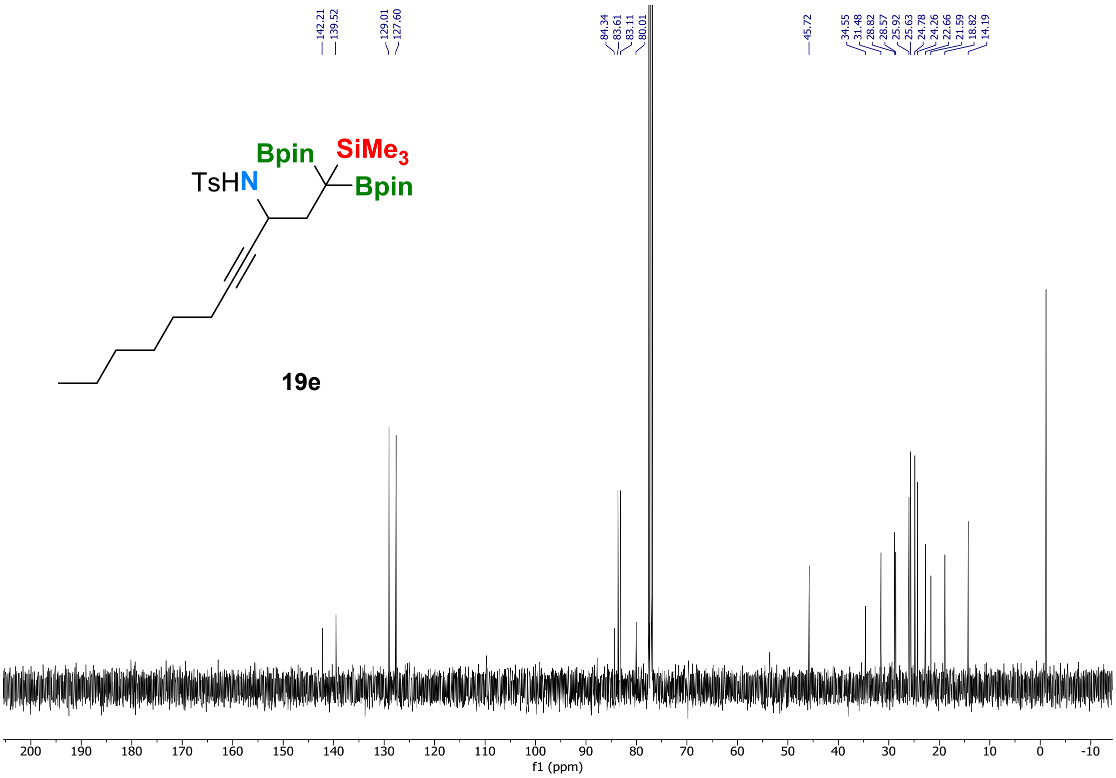

^{11}B NMR (128.3 MHz, $CDCl_3$)

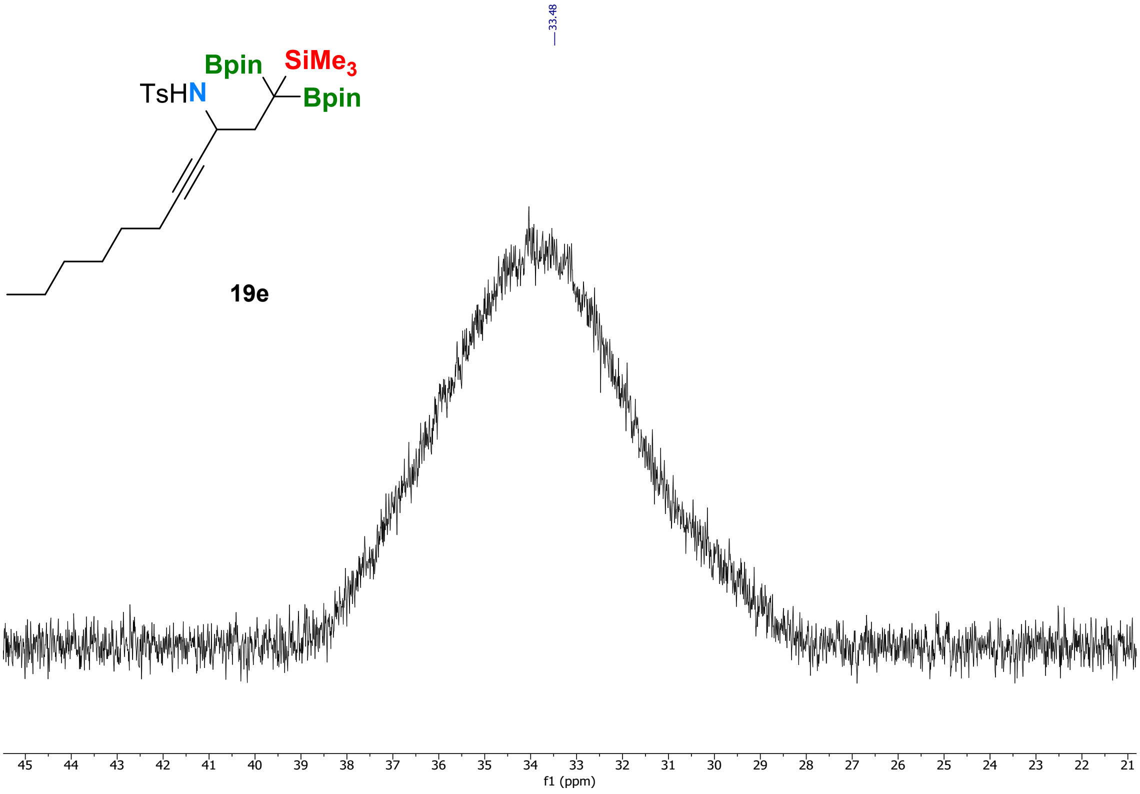

***N*-(2-(bis(4,4,5,5-tetramethyl-1,3,2-dioxaborolan-2-yl)methyl)-6-phenylhex-3-yn-1-yl)-4-methylbenzenesulfonamide (21a)**

^{1}H NMR (400 MHz, $CDCl_3$)

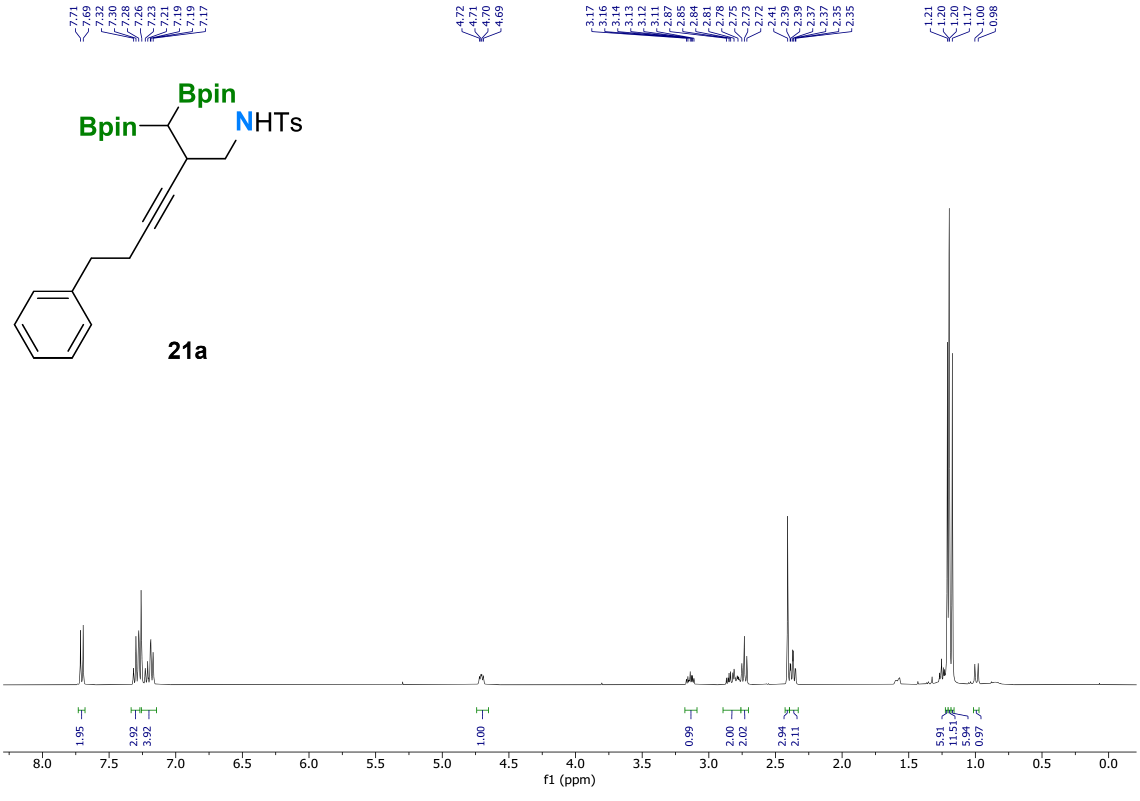

^{13}C NMR {^1H} (125 MHz, $CDCl_3$)

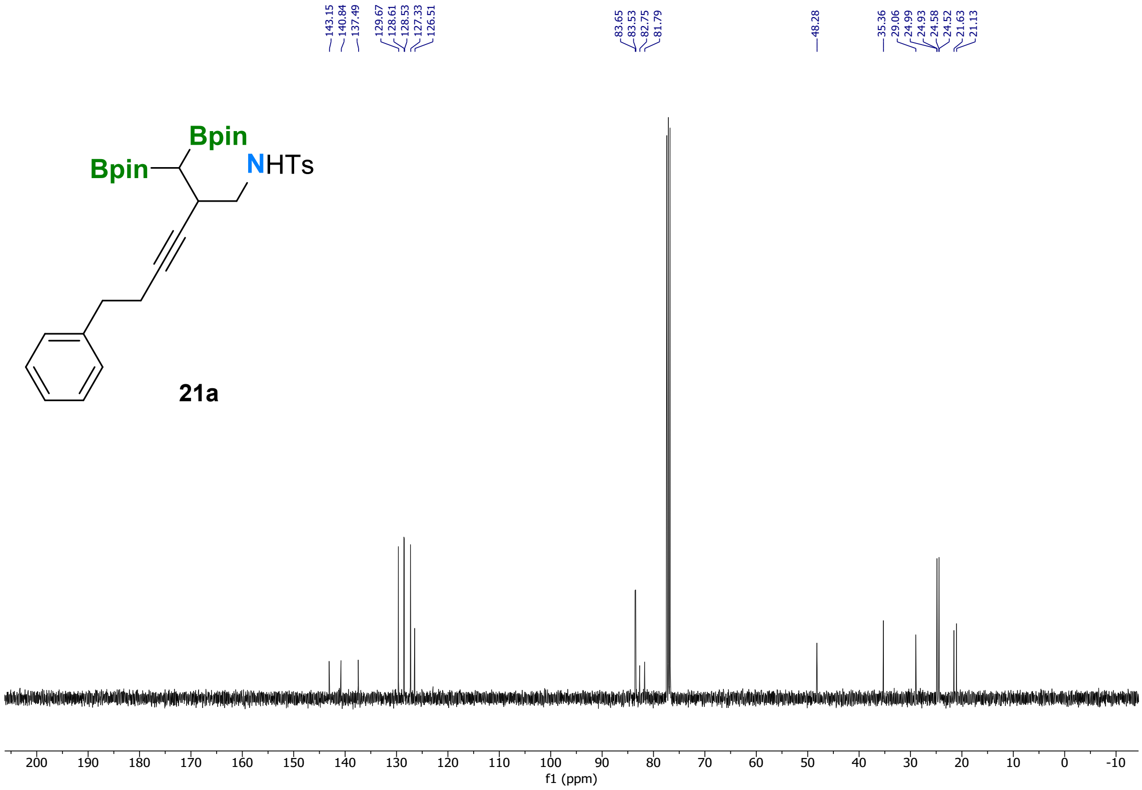

^{11}B NMR (128.3 MHz, $CDCl_3$)

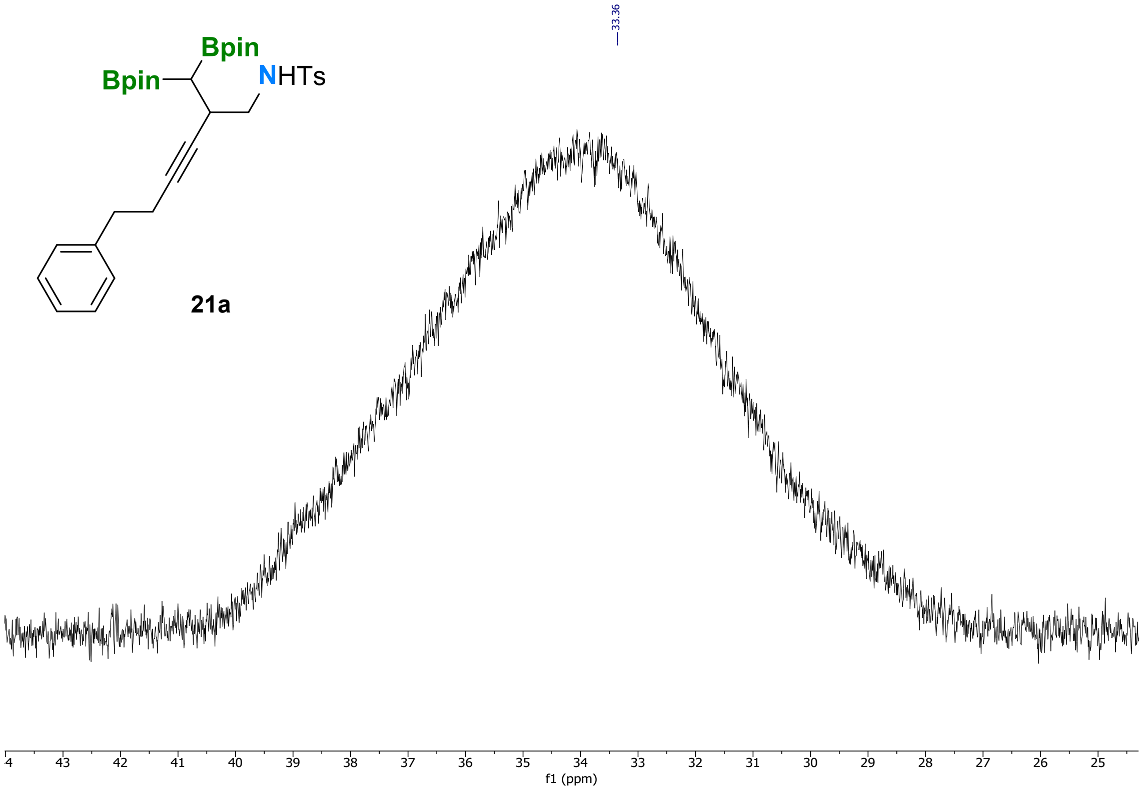

***N*-(2-(1,1-bis(4,4,5,5-tetramethyl-1,3,2-dioxaborolan-2-yl)ethyl)-6-phenylhex-3-yn-1-yl)-4-methylbenzenesulfonamide (21b)**

^{1}H NMR (400 MHz, CDCl$_3$)

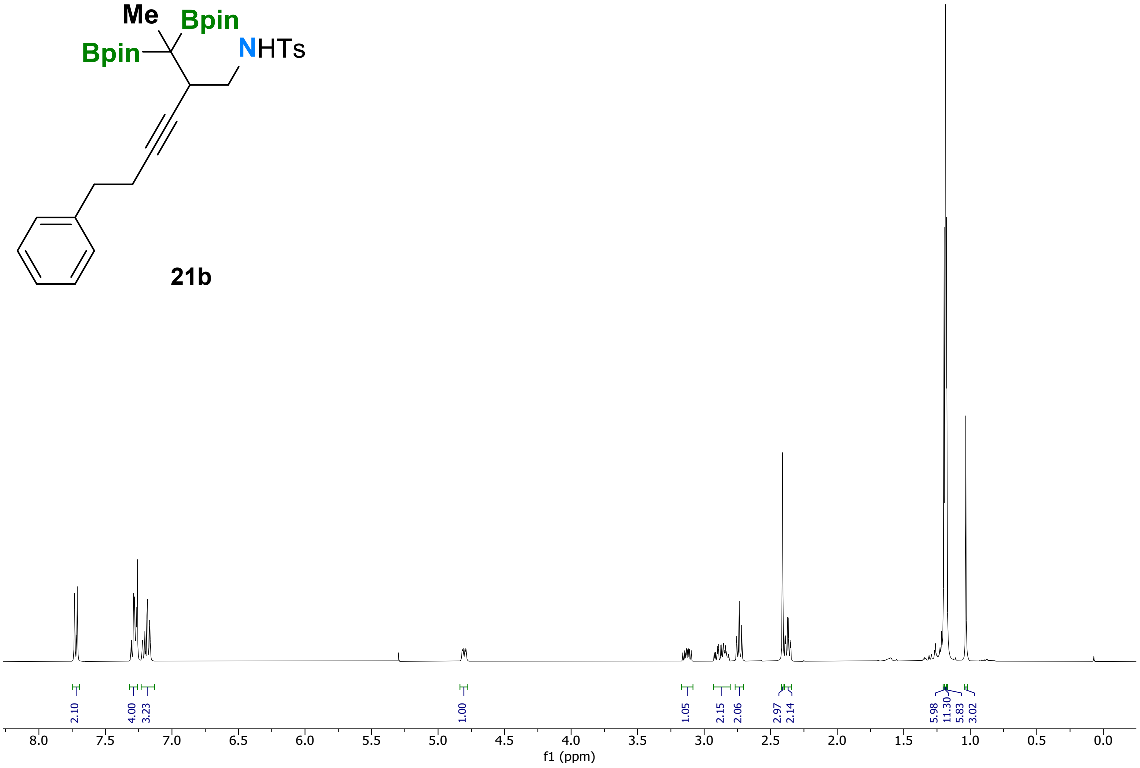

^{13}C NMR {^{1}H} (125 MHz, CDCl$_3$)

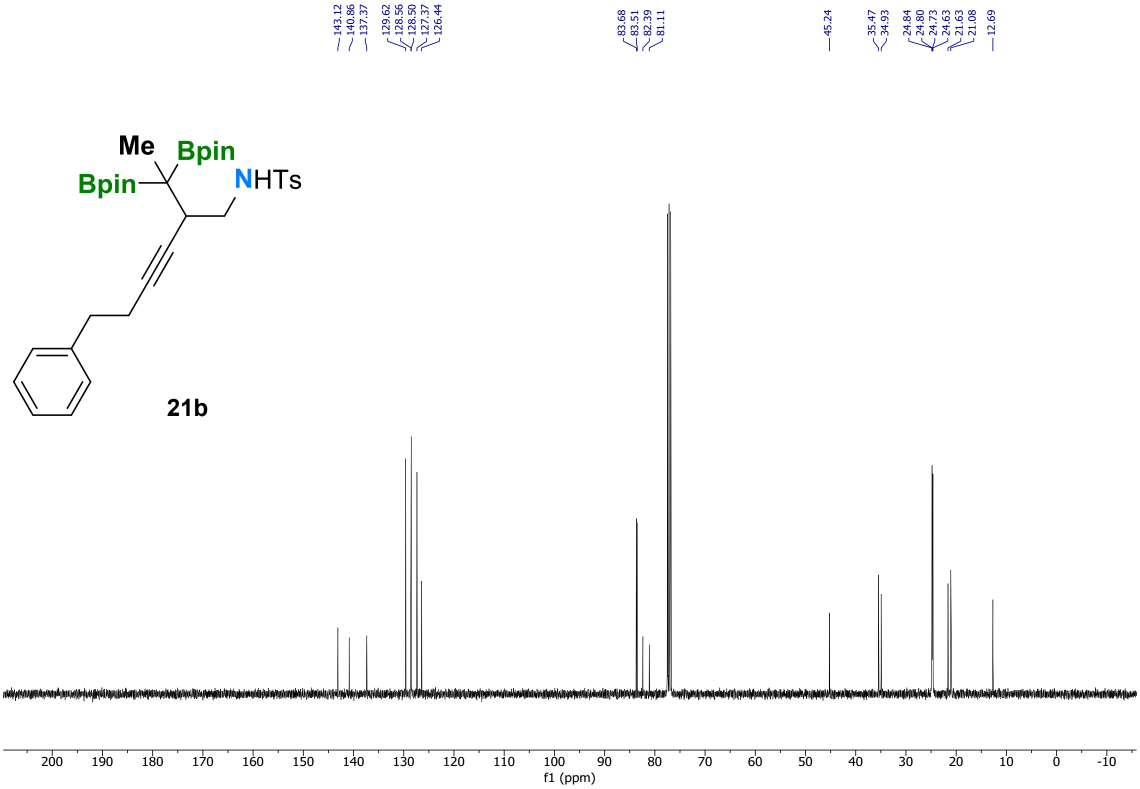

^{11}B NMR (128.3 MHz, CDCl$_3$)

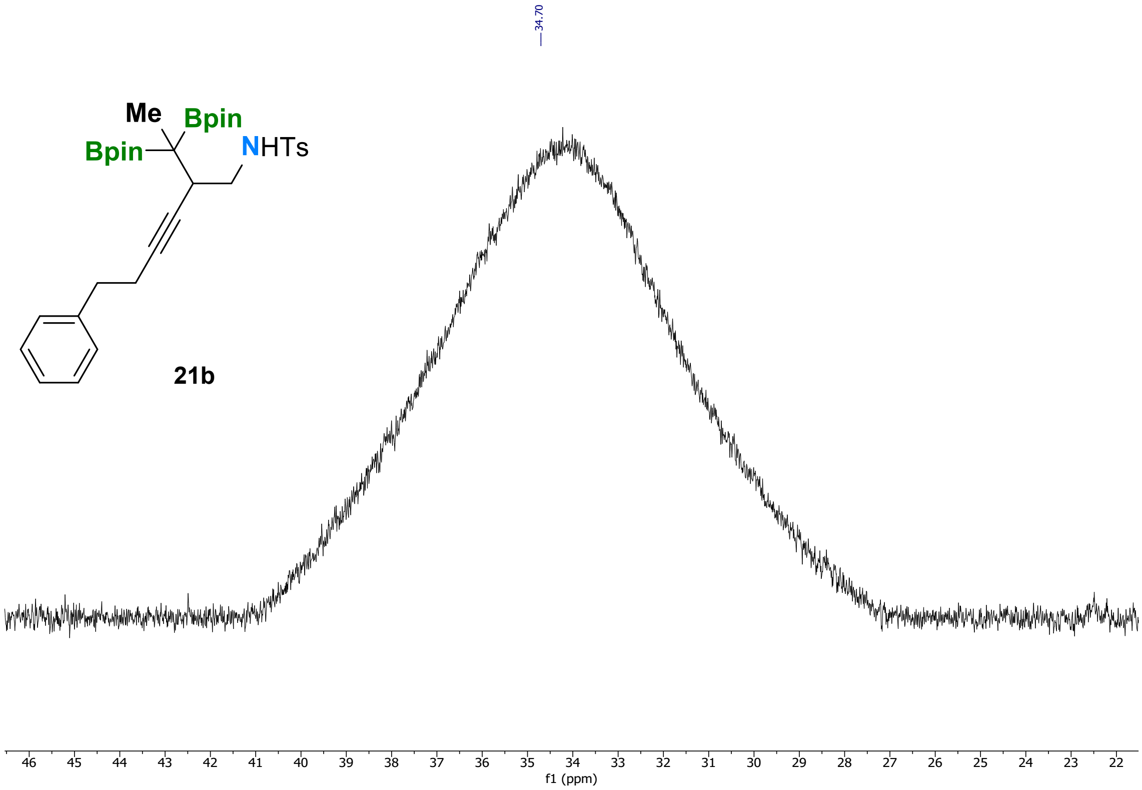

***N*-(2-(bis(4,4,5,5-tetramethyl-1,3,2-dioxaborolan-2-yl)(trimethylsilyl)methyl)-6-phenylhex-3-yn-1-yl)-4-methylbenzenesulfonamide (21e)**

^{1}H NMR (400 MHz, CDCl$_3$)

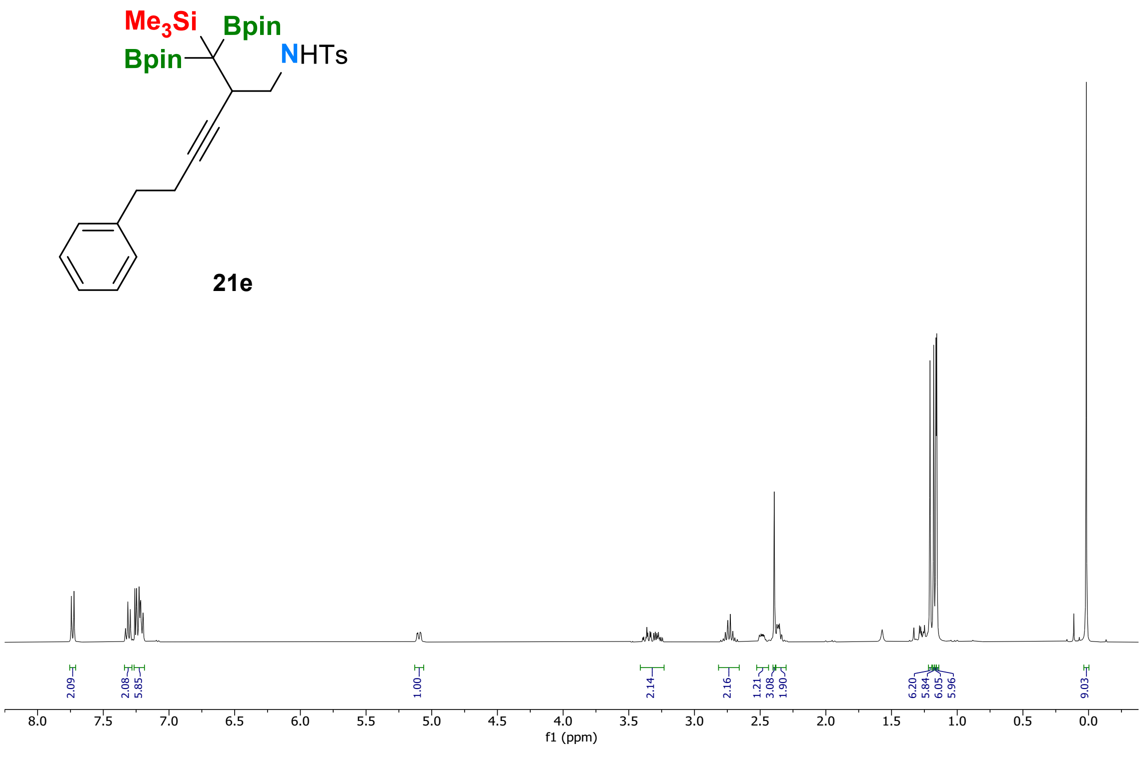

^{13}C NMR {^{1}H} (125 MHz, $CDCl_3$)

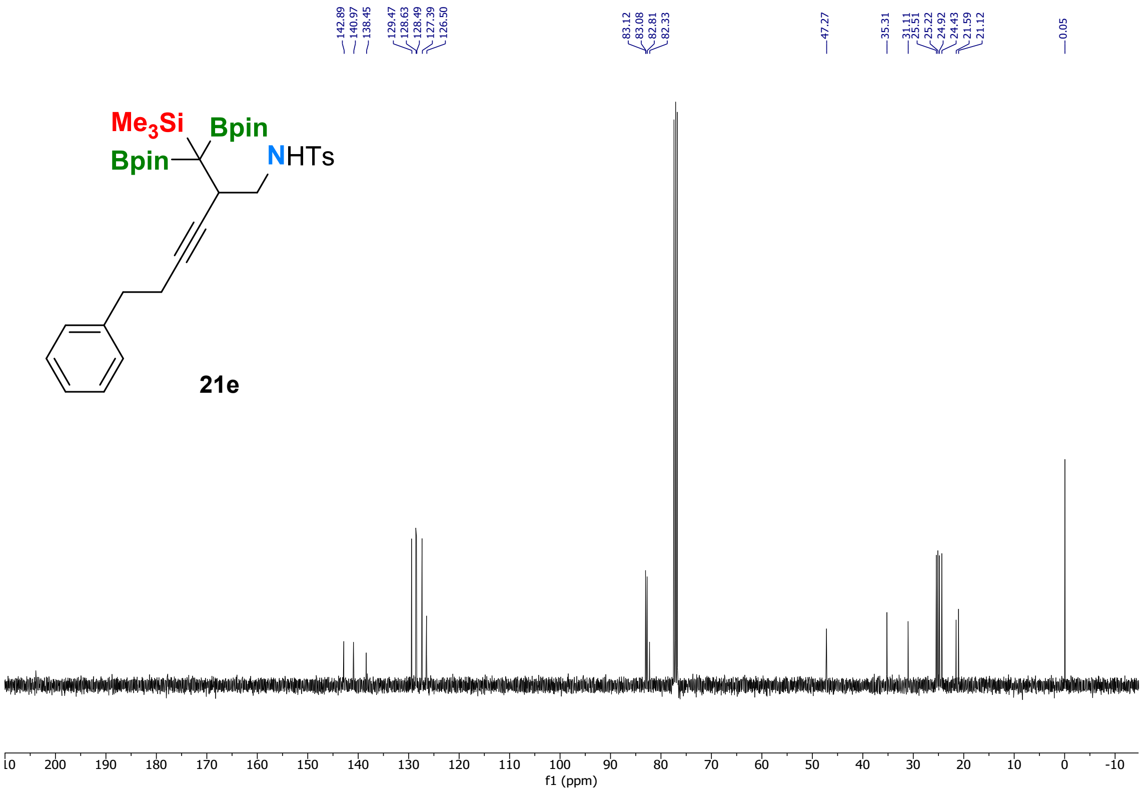

^{11}B NMR (128.3 MHz, $CDCl_3$)

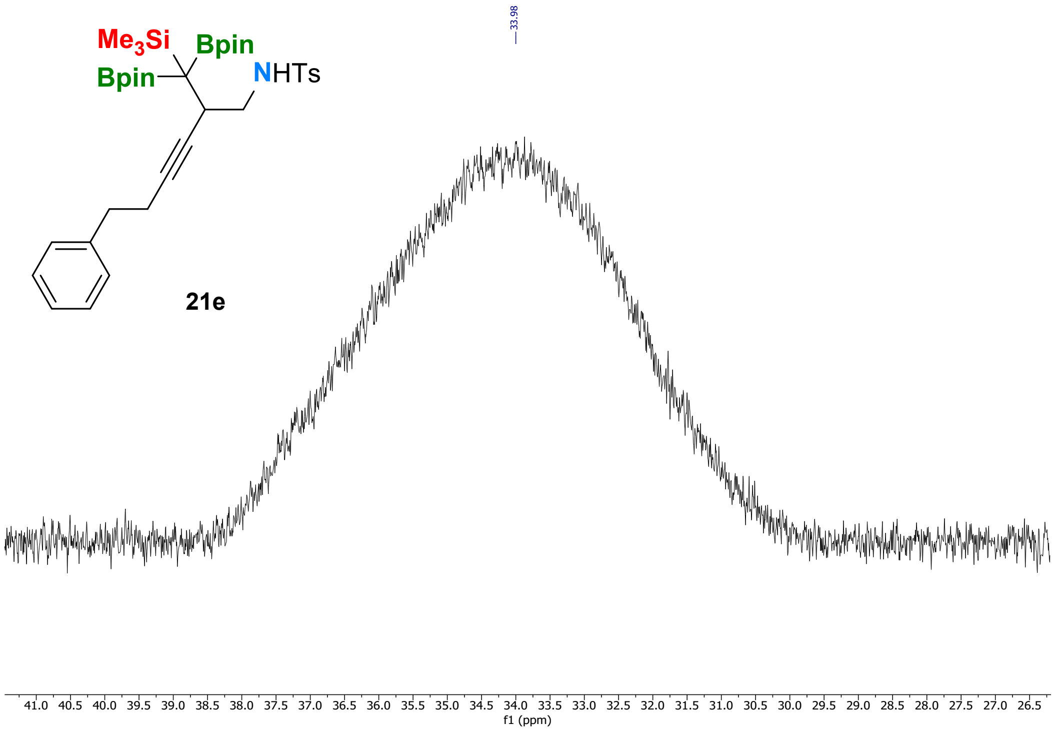

4-methyl-*N*-(8-phenyl-2,2-bis(4,4,5,5-tetramethyl-1,3,2-dioxaborolan-2-yl)oct-5-yn-4-yl)benzenesulfonamide (22b)

^{1}H NMR (400 MHz, CDCl$_3$)

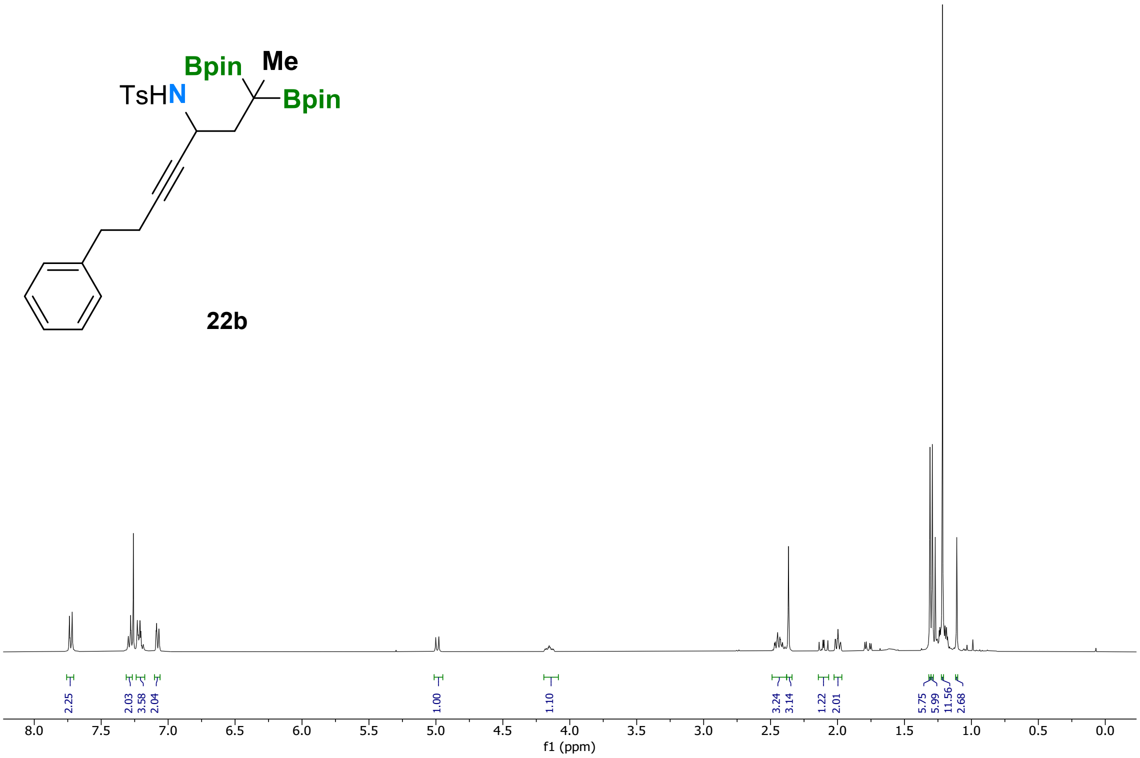

^{13}C NMR {^{1}H} (125 MHz, CDCl$_3$)

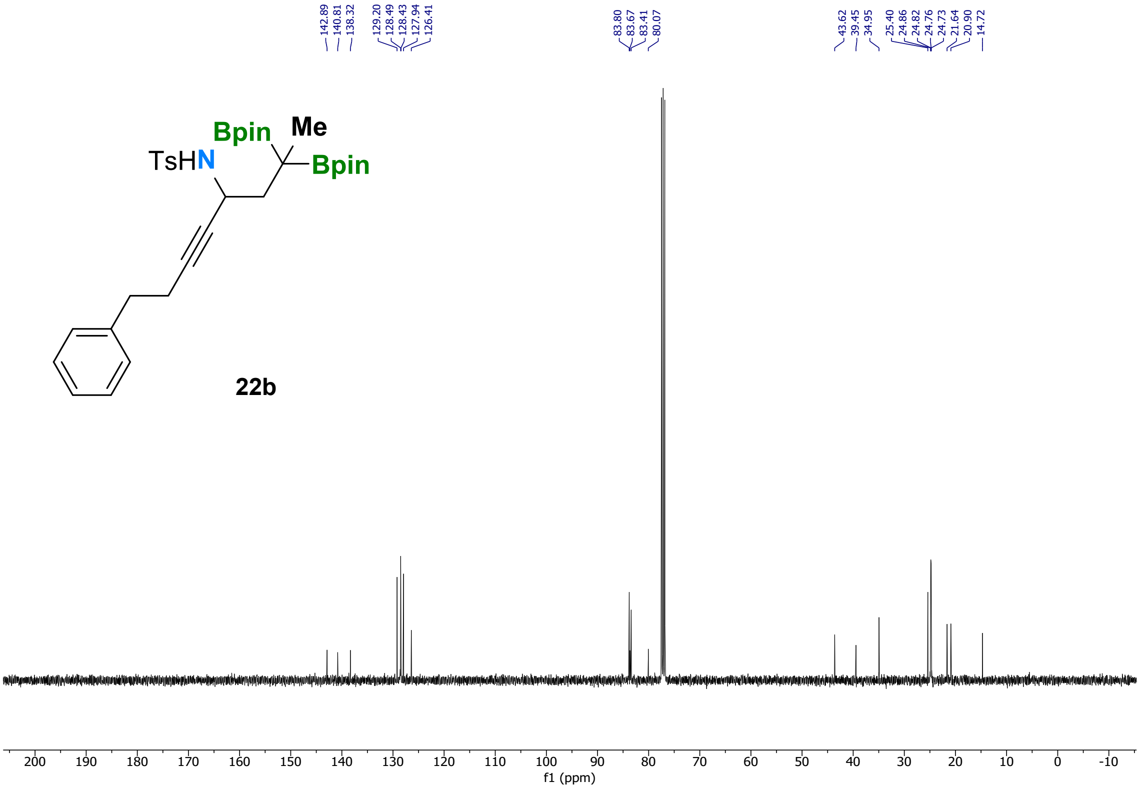

^{11}B NMR (128.3 MHz, $CDCl_3$)

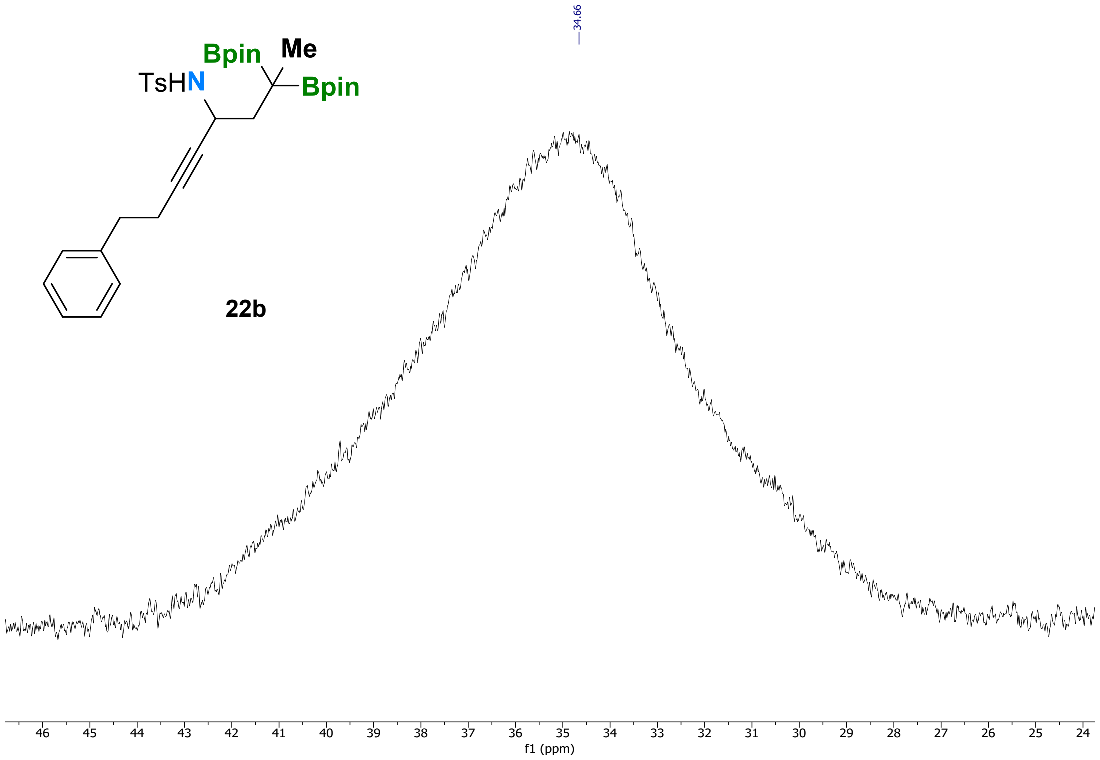

N-(2-(bis(4,4,5,5-tetramethyl-1,3,2-dioxaborolan-2-yl)methyl)-2-methyl-4-phenylbut-3-yn-1-yl)-4-methylbenzenesulfonamide (24a)

^{1}H NMR (400 MHz, $CDCl_3$)

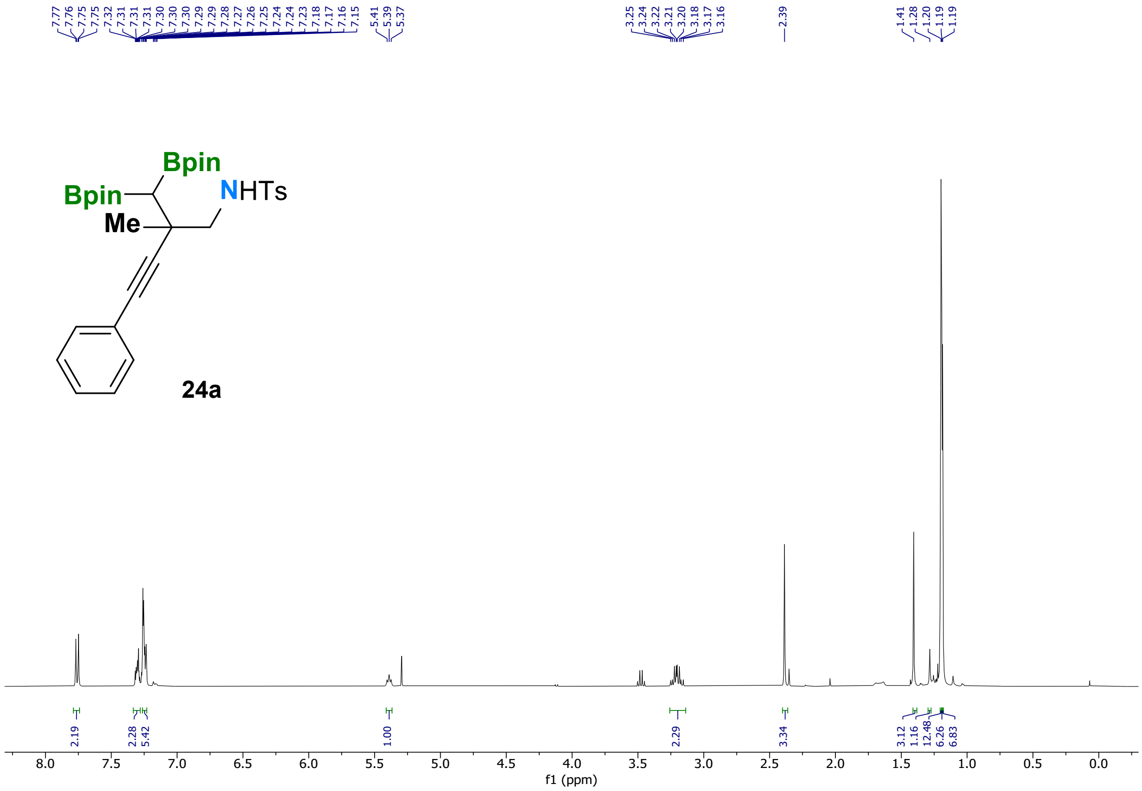

^{13}C NMR {^{1}H} (125 MHz, $CDCl_3$)

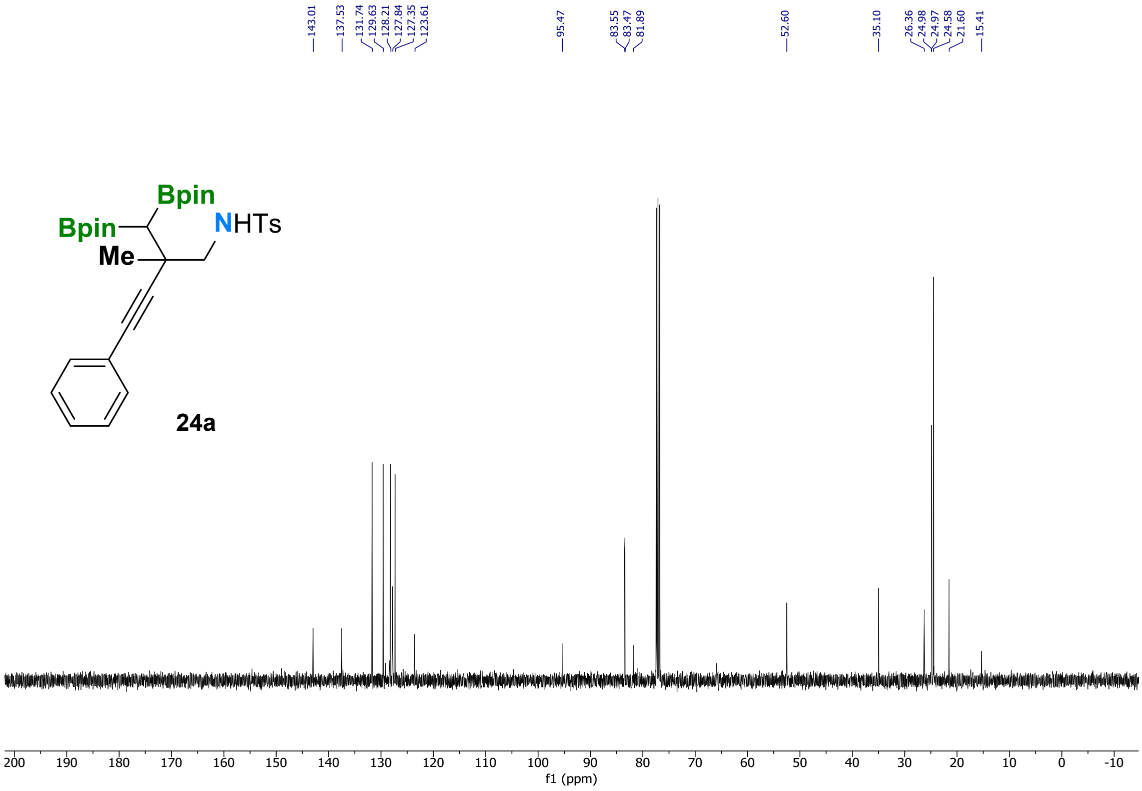

^{11}B NMR (128.3 MHz, $CDCl_3$)

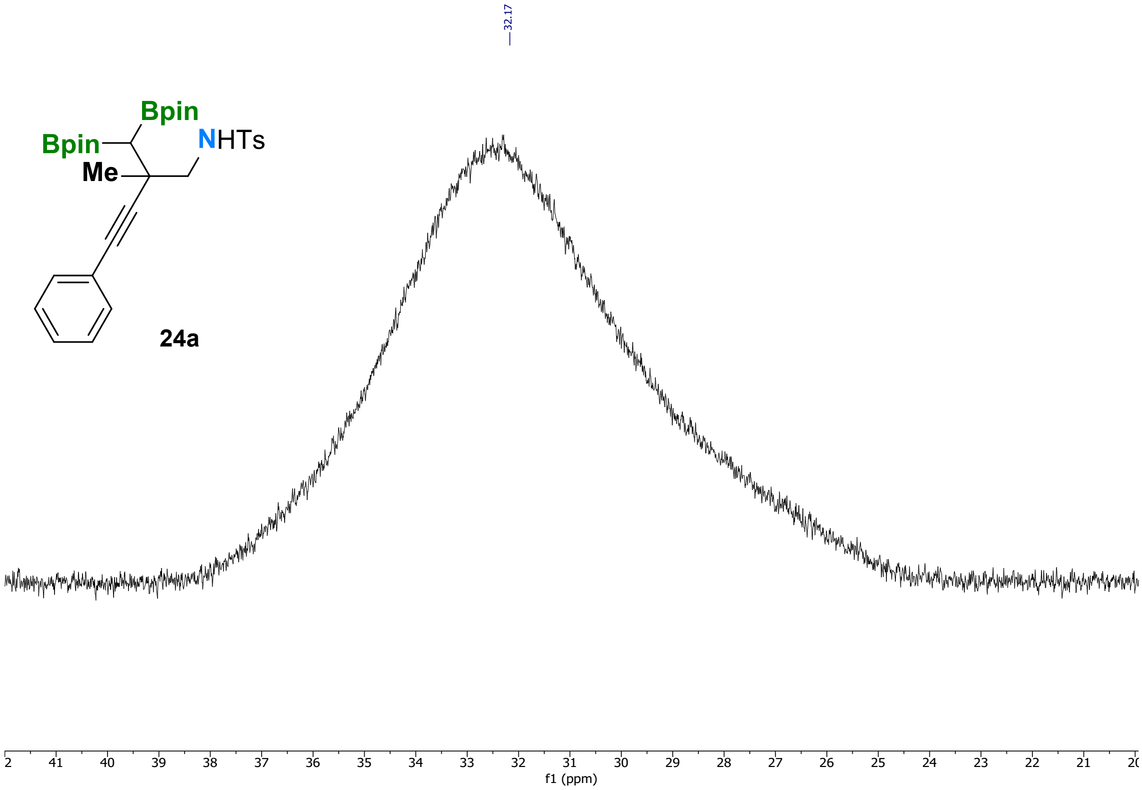

N-(2-(1,1-bis(4,4,5,5-tetramethyl-1,3,2-dioxaborolan-2-yl)ethyl)-2-methyl-4-phenylbut-3-yn-1-yl)-4-methylbenzenesulfonamide (24b)

^{1}H NMR (400 MHz, $CDCl_3$)

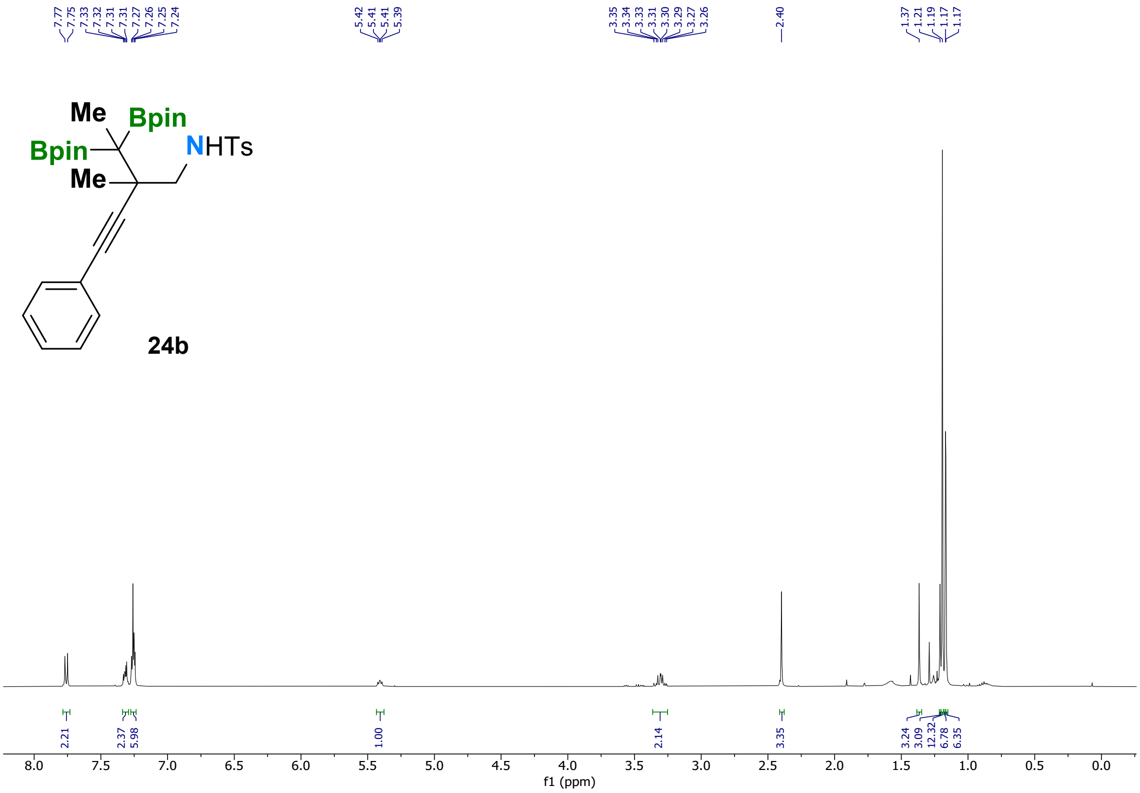

^{13}C NMR {^{1}H} (125 MHz, $CDCl_3$)

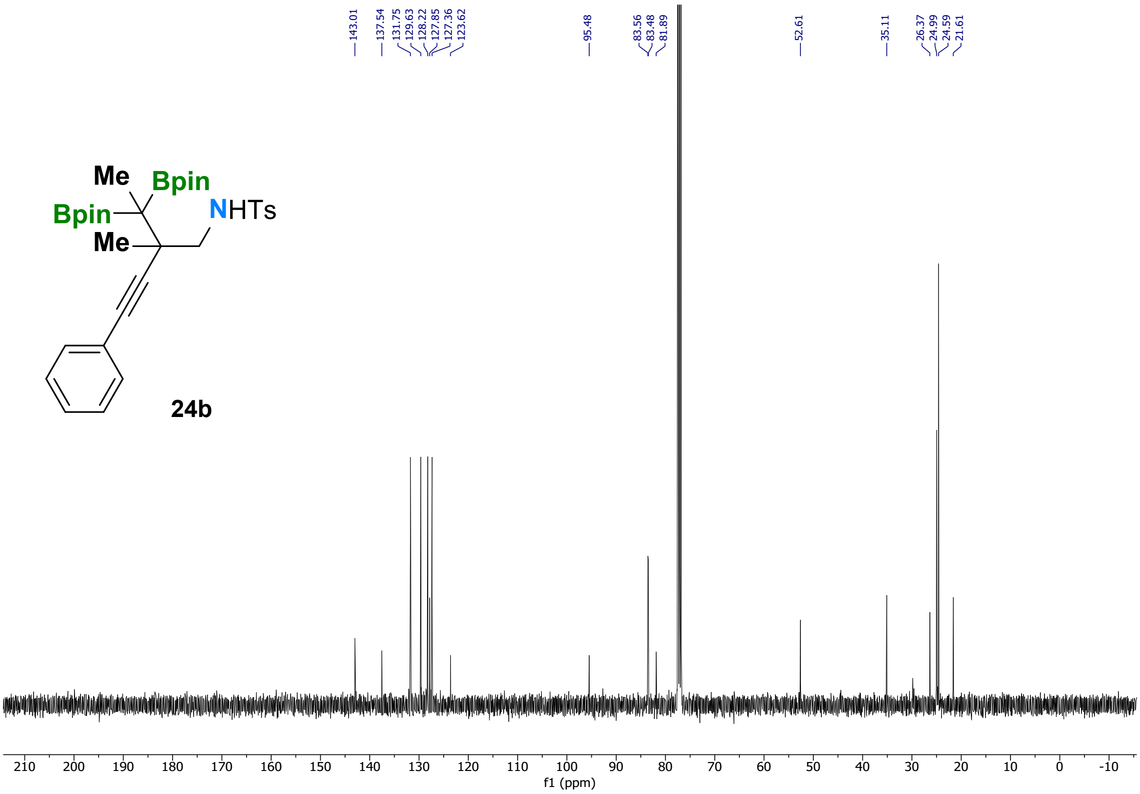

^{11}B NMR (128.3 MHz, CDCl$_3$)

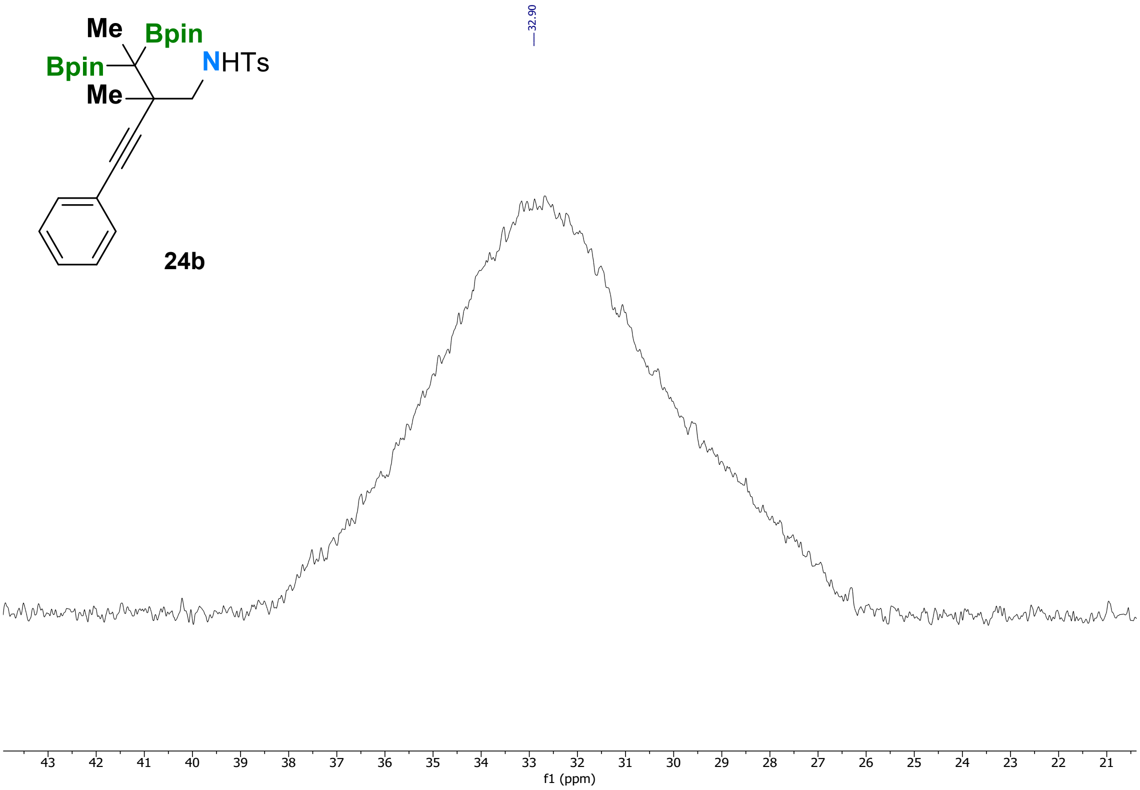

N-(2-(bis(4,4,5,5-tetramethyl-1,3,2-dioxaborolan-2-yl)(trimethylsilyl)methyl)-2-methyl-4-phenylbut-3-yn-1-yl)-4-methylbenzenesulfonamide (24e)

^{1}H NMR (400 MHz, CDCl$_3$)

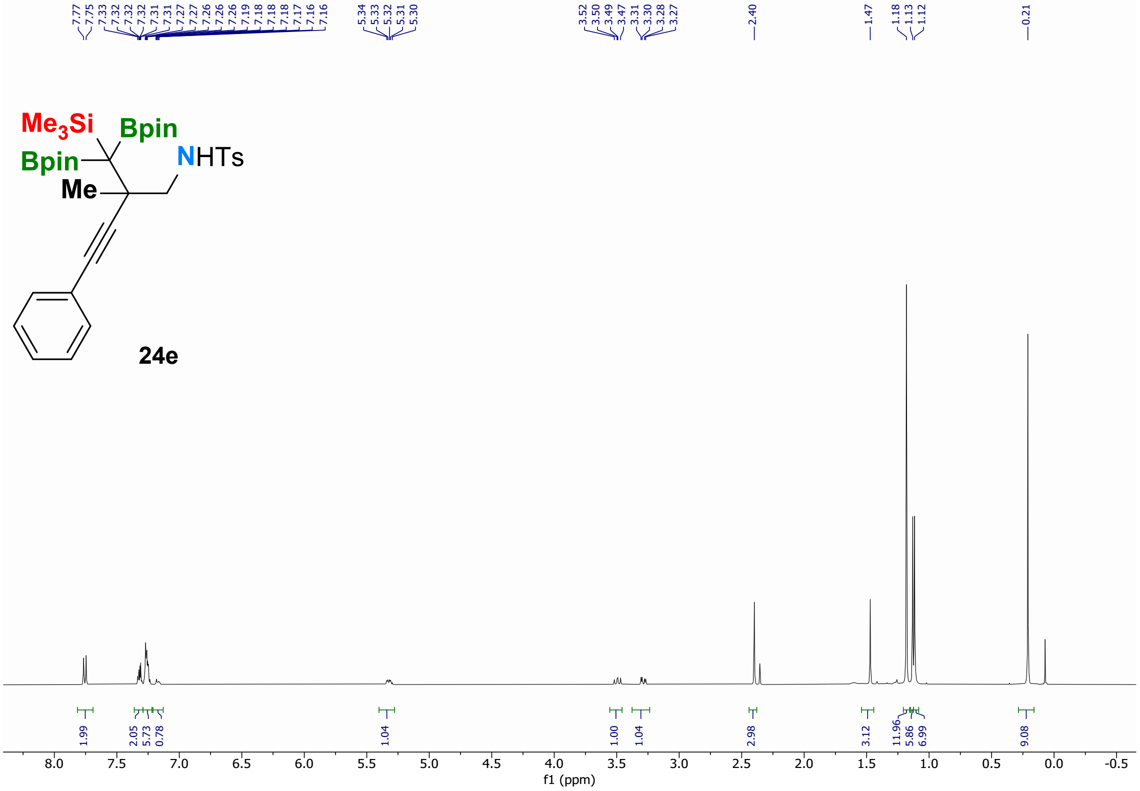

^{13}C NMR {^{1}H} (125 MHz, $CDCl_3$)

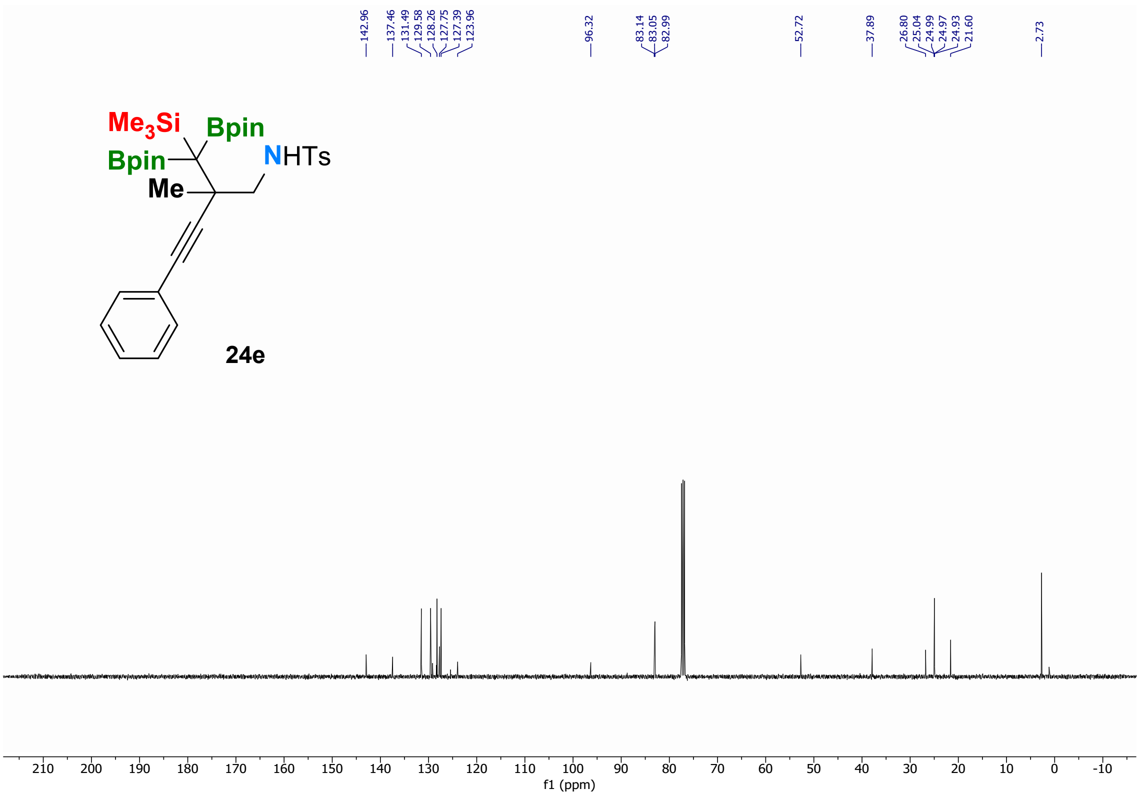

^{11}B NMR (128.3 MHz, $CDCl_3$)

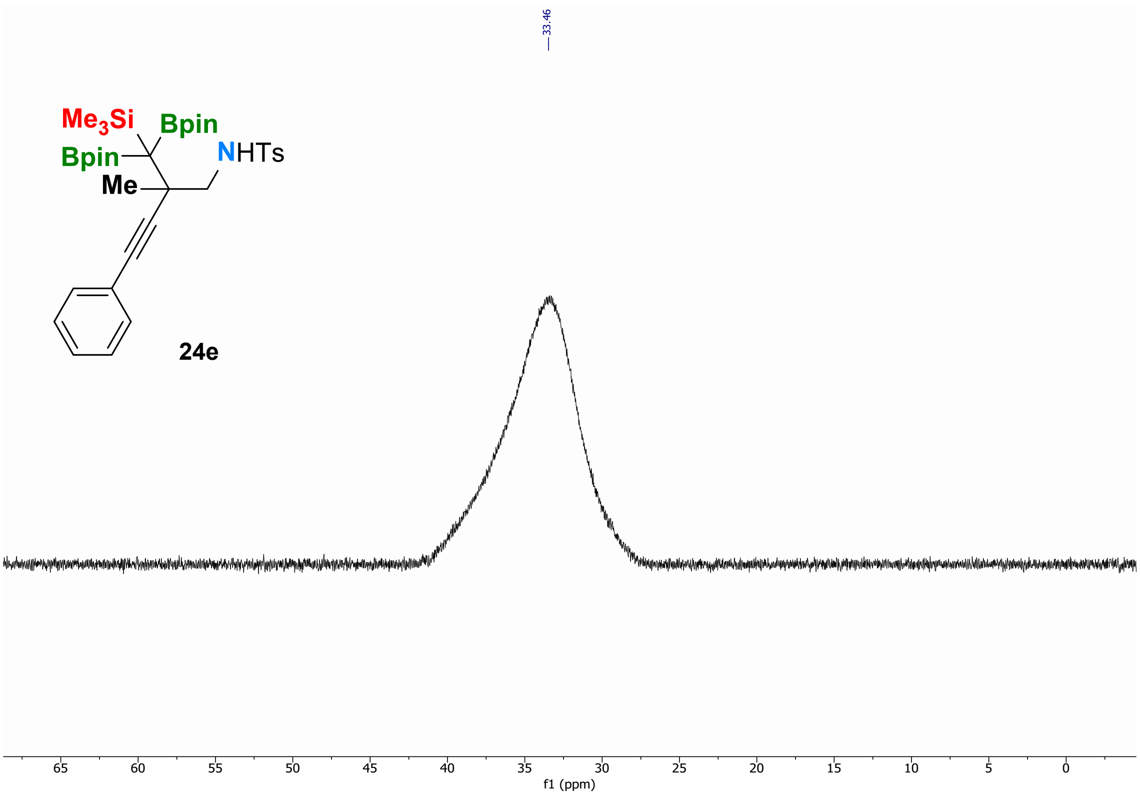

_N_-(1-cyclohexyl-3-methyl-5,5-bis(4,4,5,5-tetramethyl-1,3,2-dioxaborolan-2-yl)-5-(trimethylsilyl)pent-1-yn-3-yl)-4-methylbenzenesulfonamide (25e)

^{1}H NMR (400 MHz, $CDCl_3$)

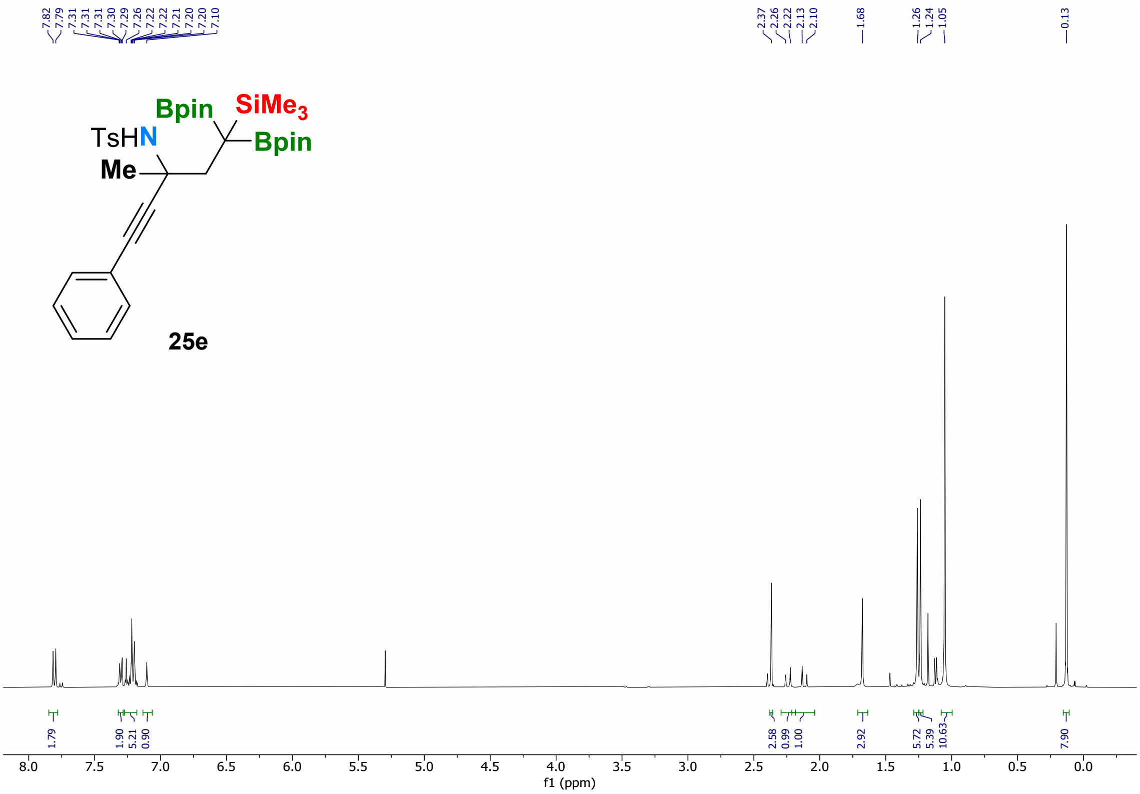

^{13}C NMR {^{1}H} (125 MHz, $CDCl_3$)

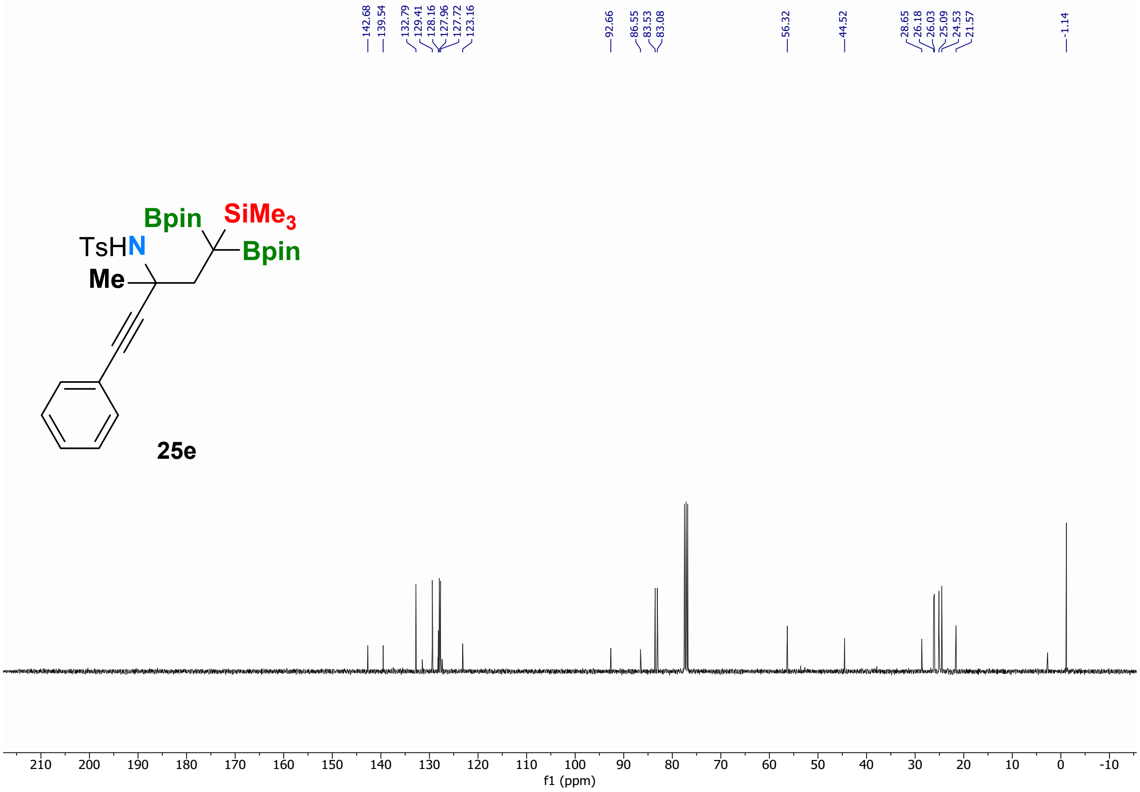

^{11}B NMR (128.3 MHz, $CDCl_3$)

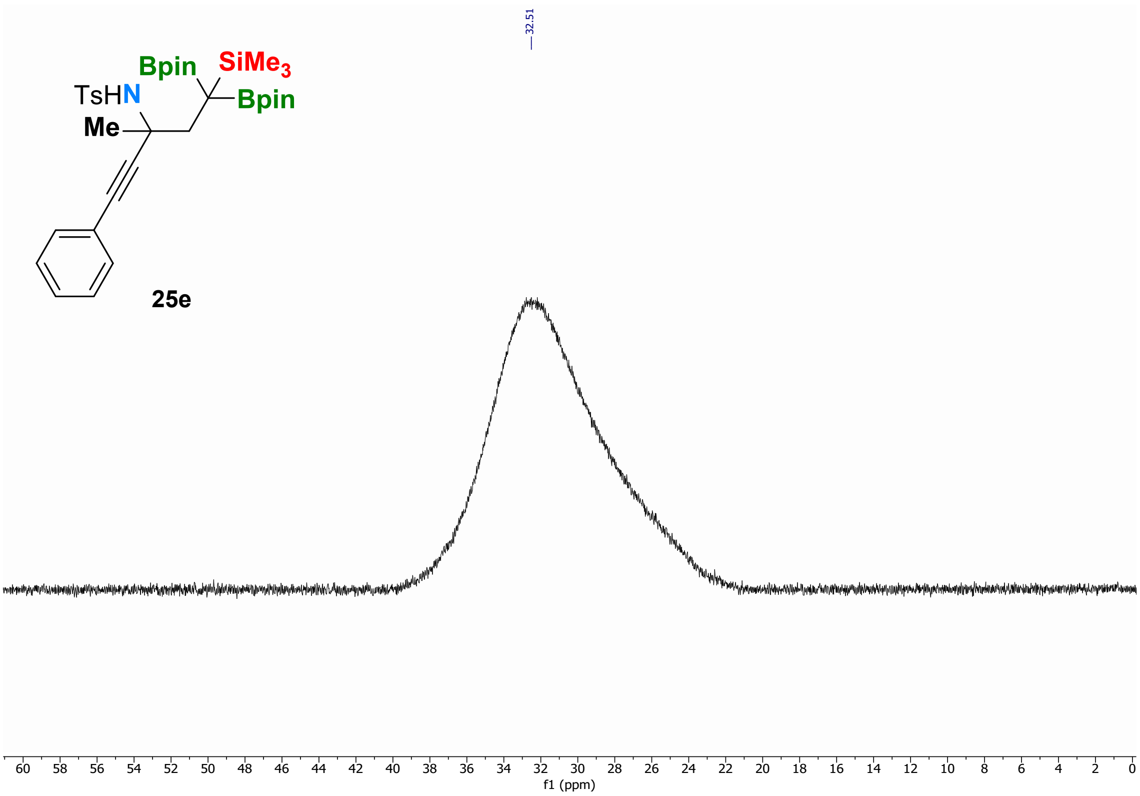

N-(2-(bis(4,4,5,5-tetramethyl-1,3,2-dioxaborolan-2-yl)(trimethylsilyl)methyl)-2-methyl-4-(naphthalen-2-yl)but-3-yn-1-yl)-4-methylbenzenesulfonamide (27e)

^{1}H NMR (400 MHz, $CDCl_3$)

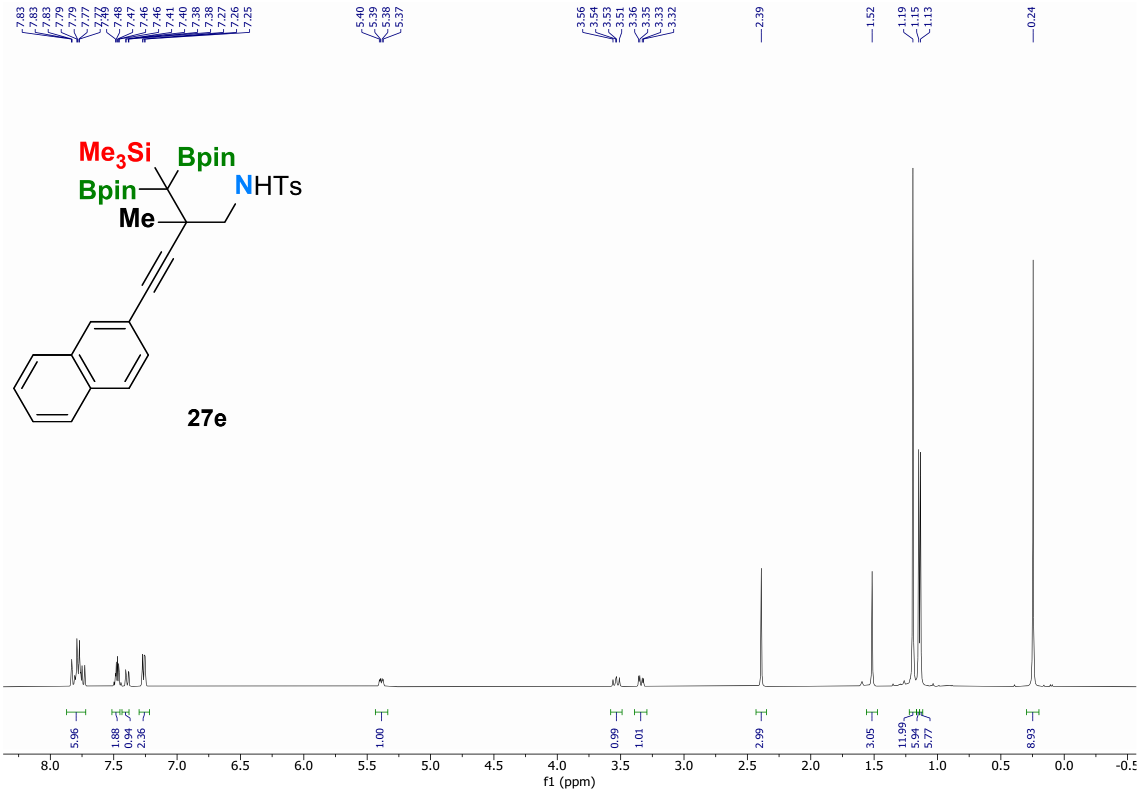

^{13}C NMR {^{1}H} (125 MHz, $CDCl_3$)

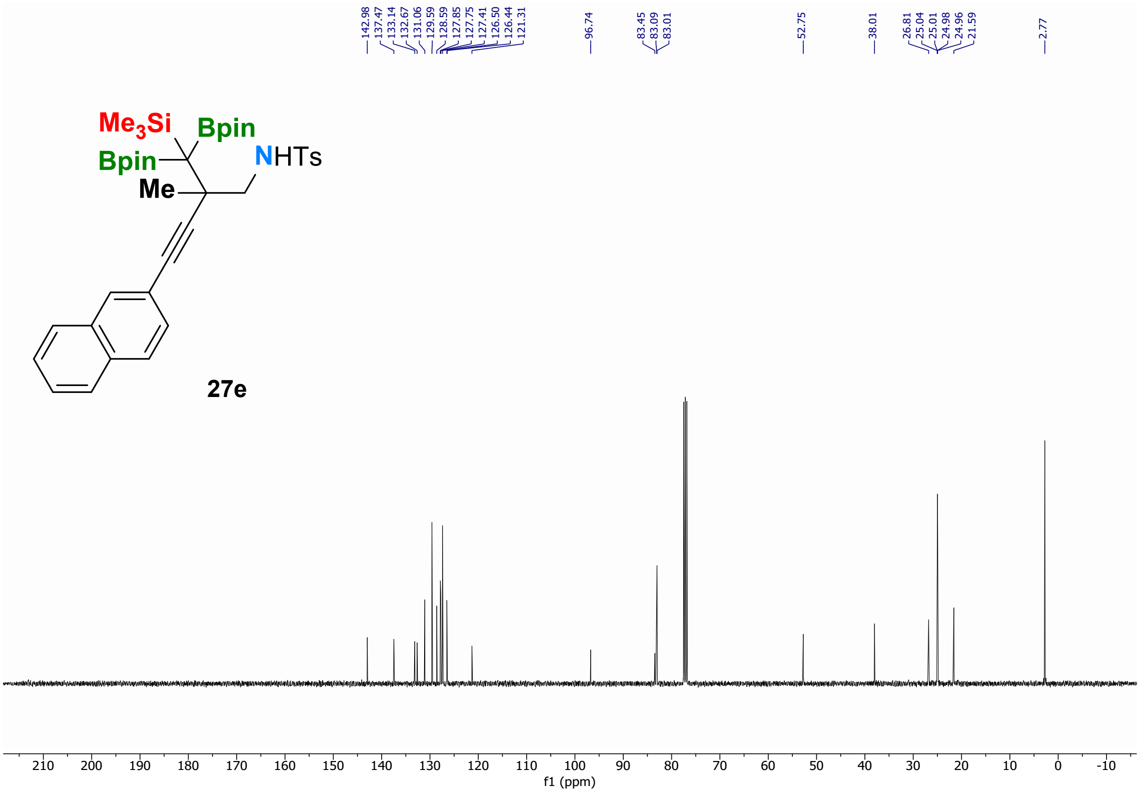

^{11}B NMR (128.3 MHz, $CDCl_3$)

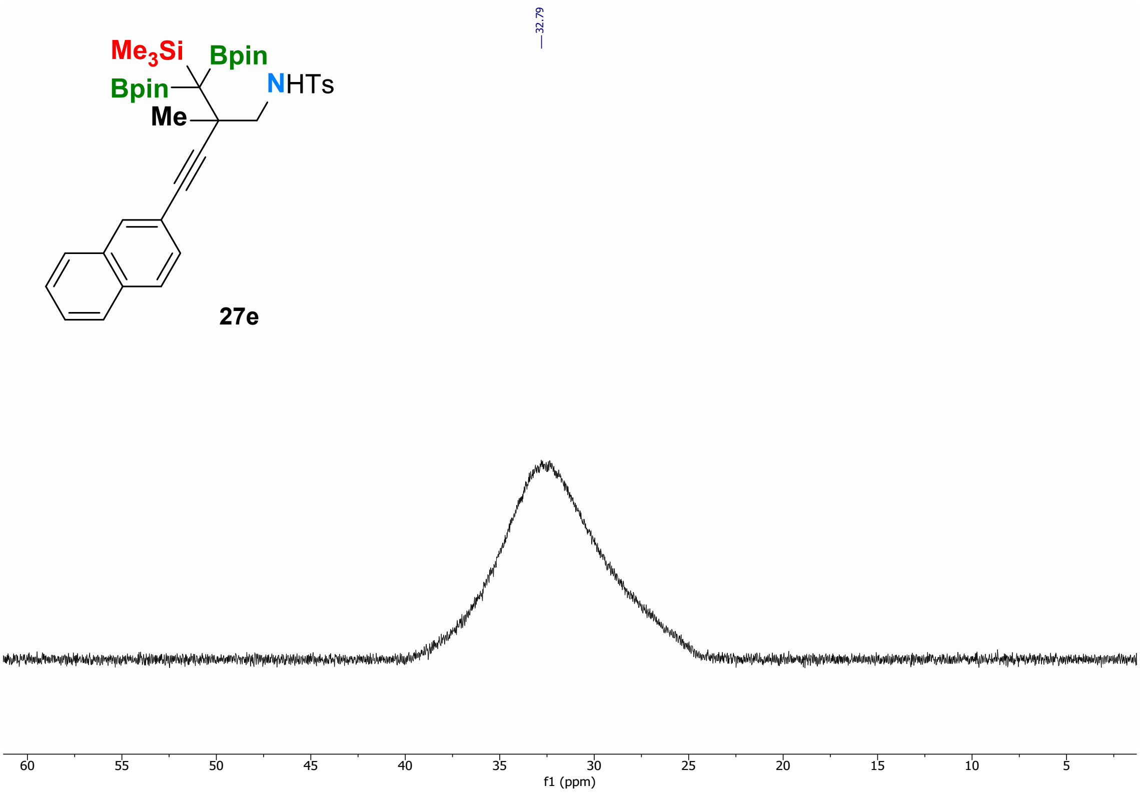

4-methyl-*N*-(3-methyl-1-(naphthalen-2-yl)-5,5-bis(4,4,5,5-tetramethyl-1,3,2-dioxaborolan-2-yl)-5-(trimethylsilyl)pent-1-yn-3-yl)benzenesulfonamide (28e)

^{1}H NMR (400 MHz, $CDCl_3$)

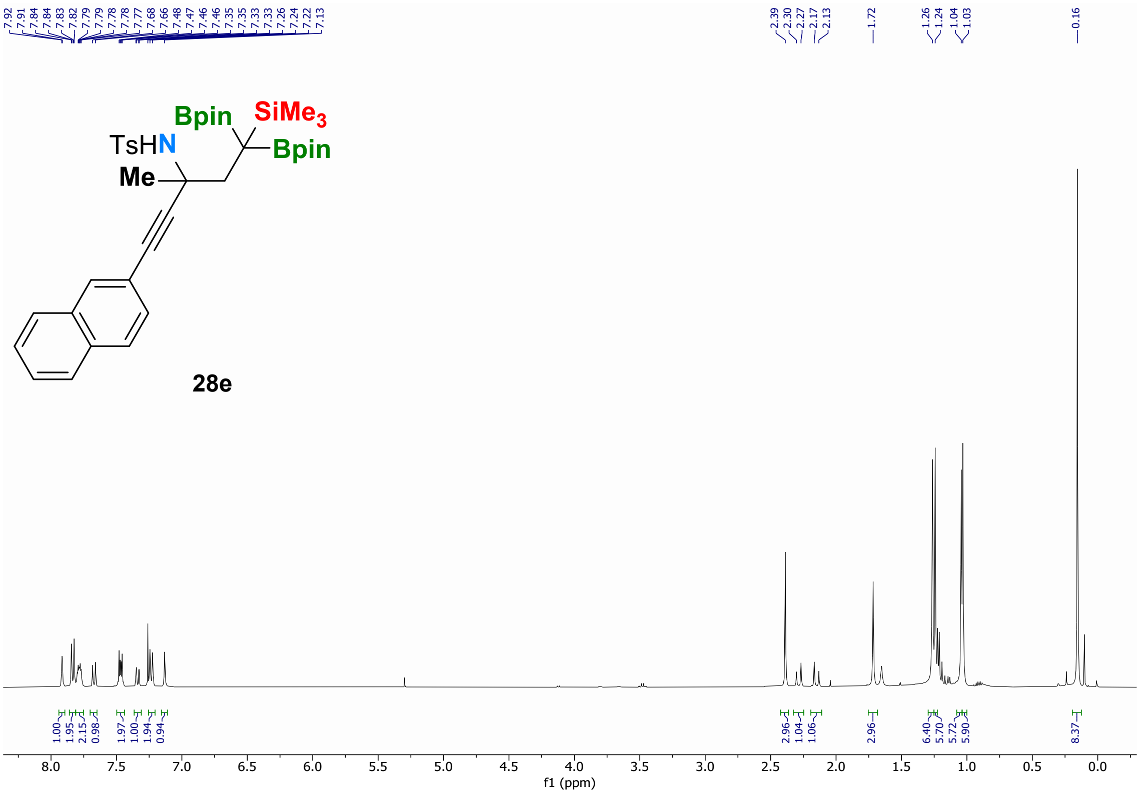

^{13}C NMR {[1]H} (125 MHz, $CDCl_3$)

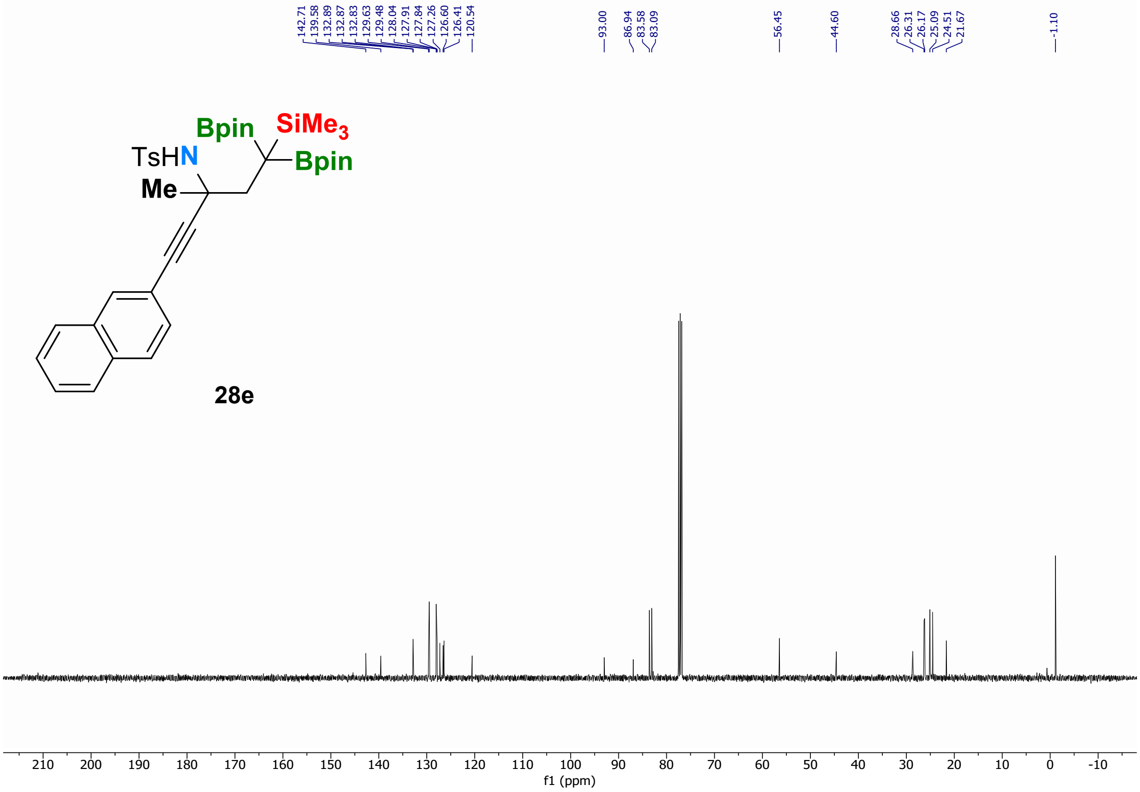

^{11}B NMR (128.3 MHz, $CDCl_3$)

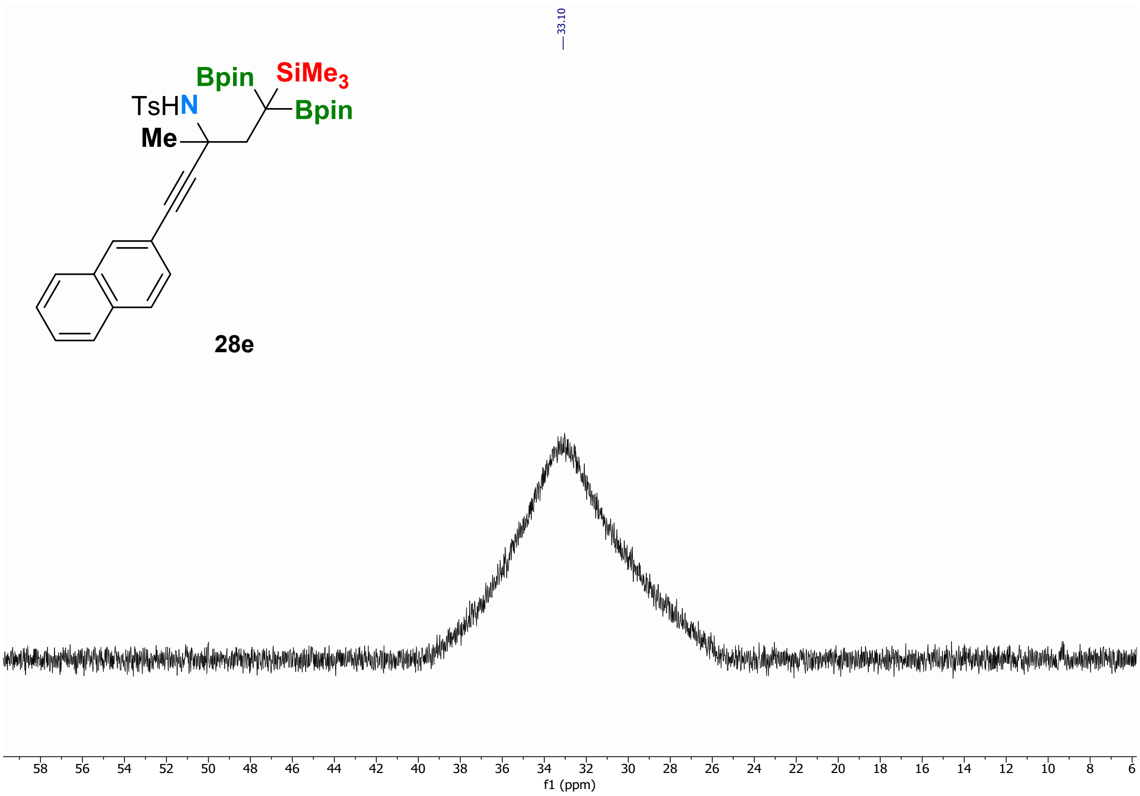

***N*-(2-(bis(4,4,5,5-tetramethyl-1,3,2-dioxaborolan-2-yl)methyl)-4-(4-(trifluoromethyl)phenyl)but-3-yn-1-yl)-4-methylbenzenesulfonamide (36a)**

^{1}H NMR (400 MHz, $CDCl_3$)

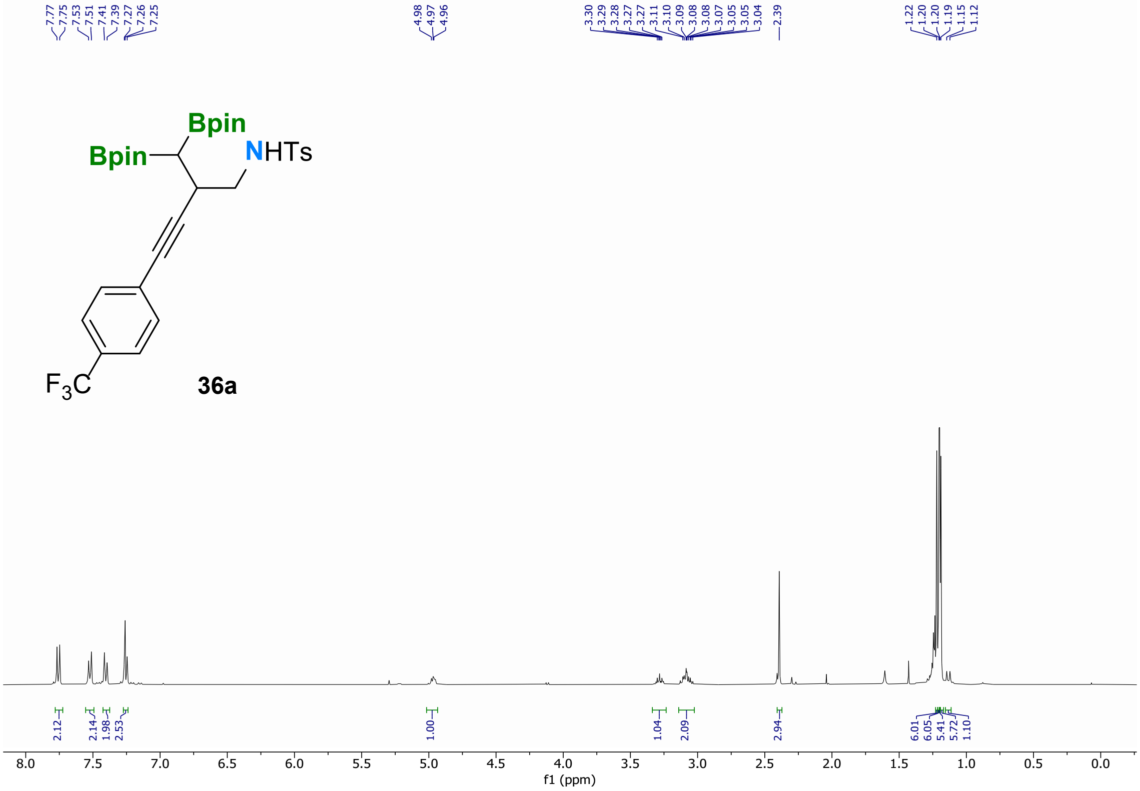

^{13}C NMR {^{1}H} (125 MHz, $CDCl_3$)

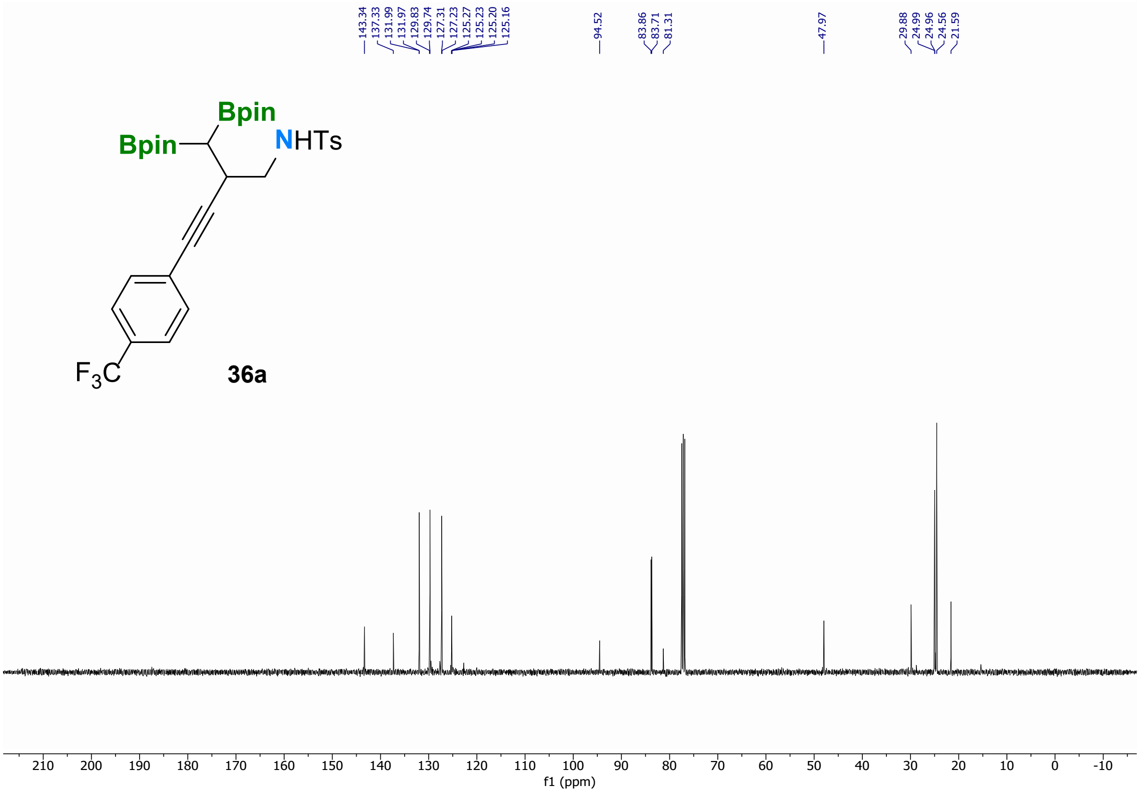

^{11}B NMR (128.3 MHz, $CDCl_3$)

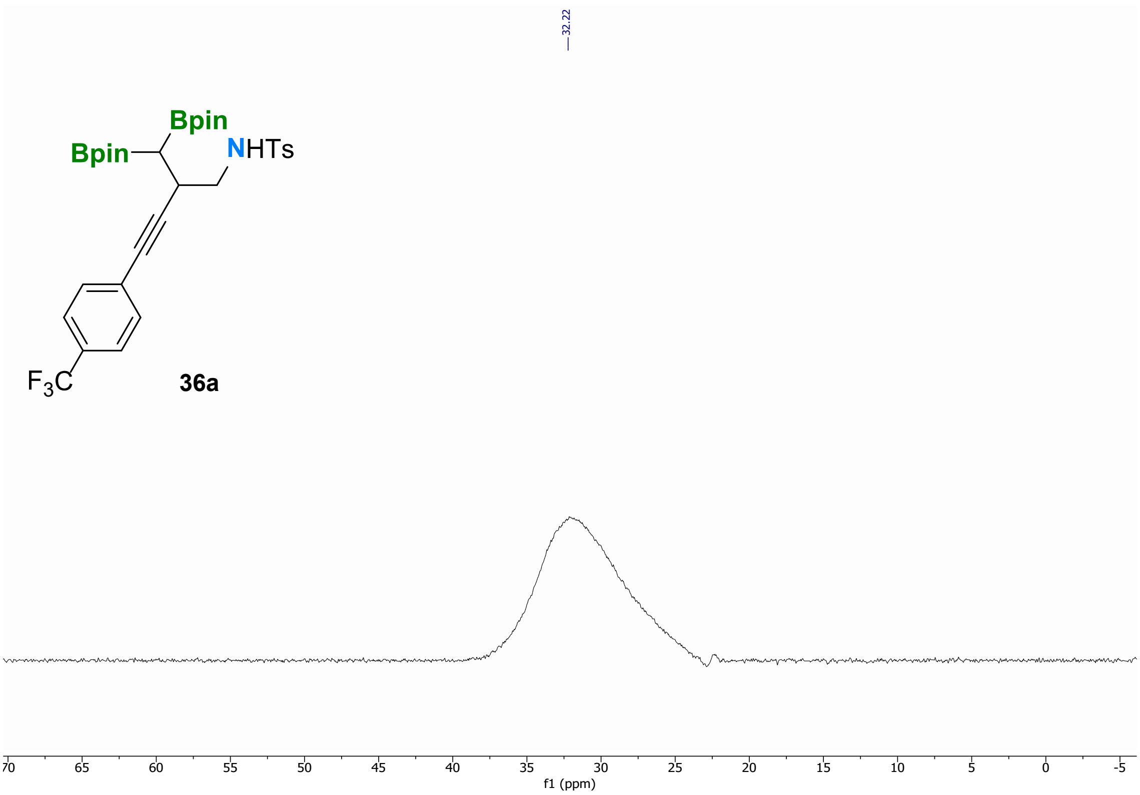

^{19}F NMR {^{1}H} (376.5 MHz, $CDCl_3$)

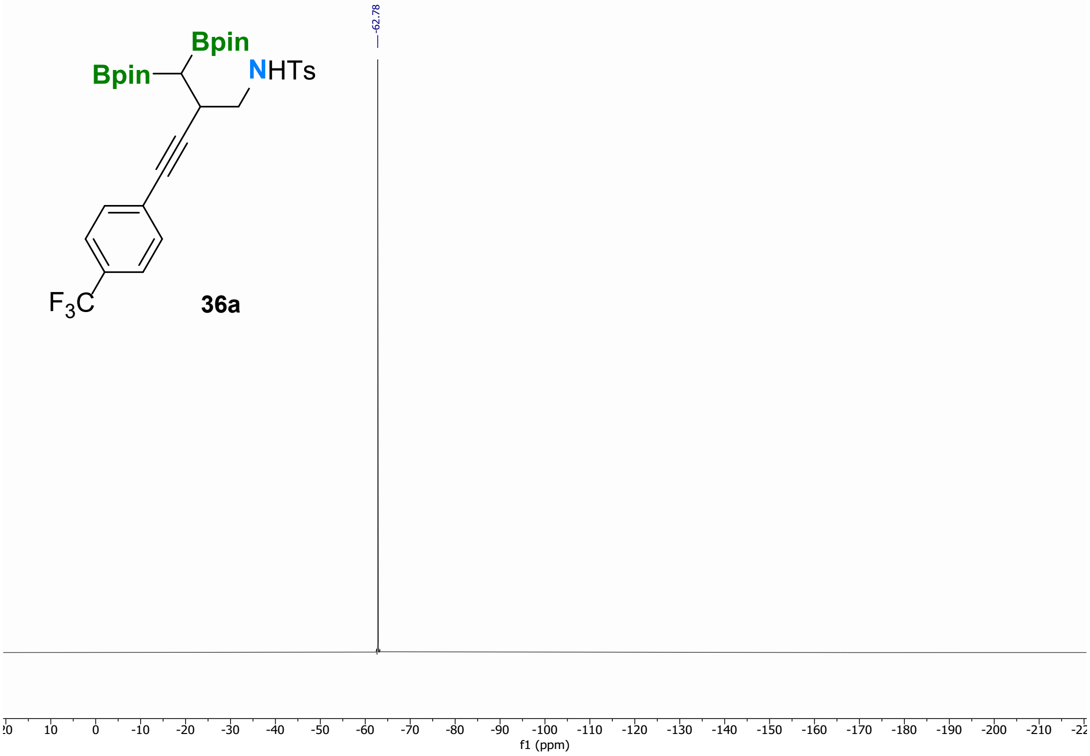

N-(2-(bis(4,4,5,5-tetramethyl-1,3,2-dioxaborolan-2-yl)methyl)-4-(4-(trifluoromethyl)phenyl)but-3-yn-1-yl)-4-methylbenzenesulfonamide (36b)

^{1}H NMR (400 MHz, $CDCl_3$)

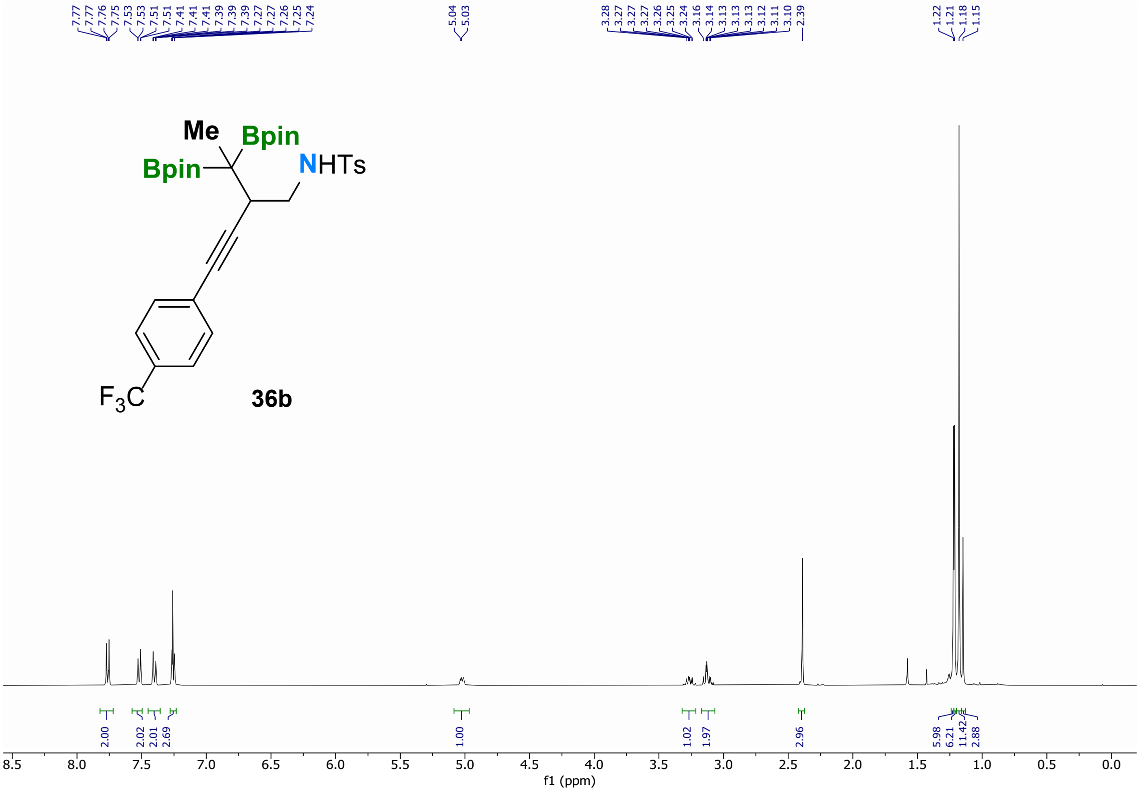

^{13}C NMR {^{1}H} (125 MHz, $CDCl_3$)

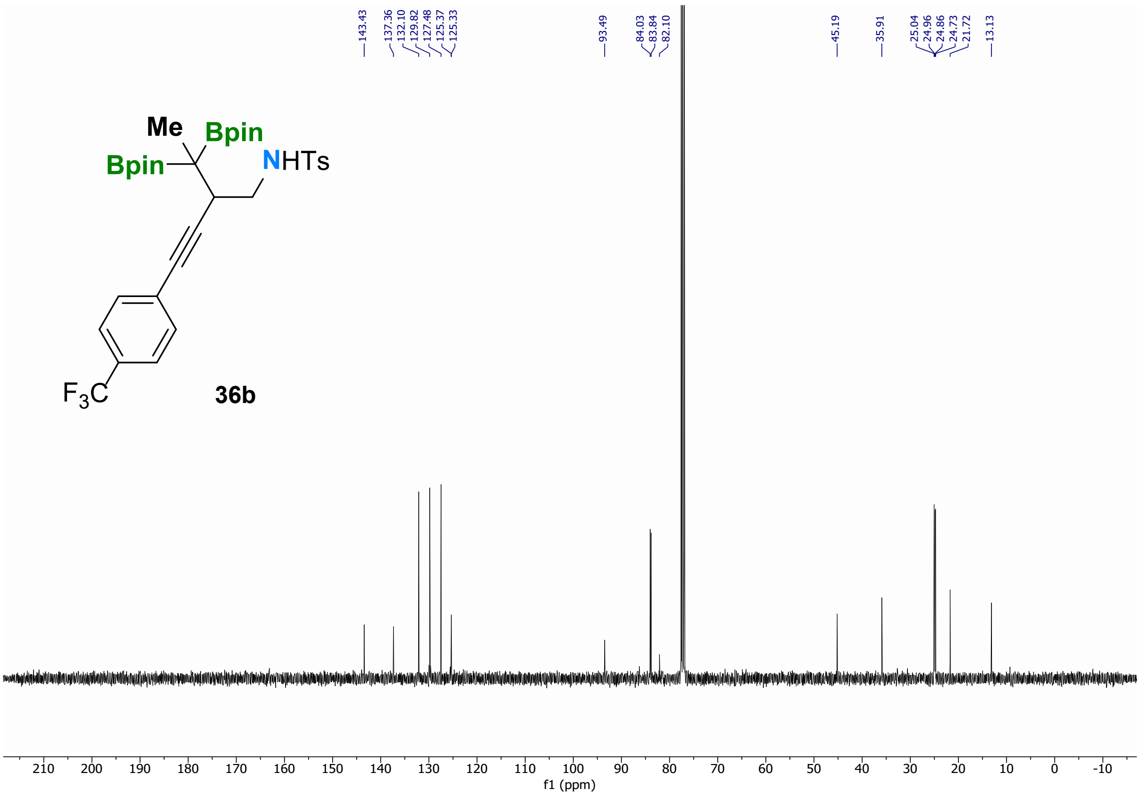

^{11}B NMR (128.3 MHz, $CDCl_3$)

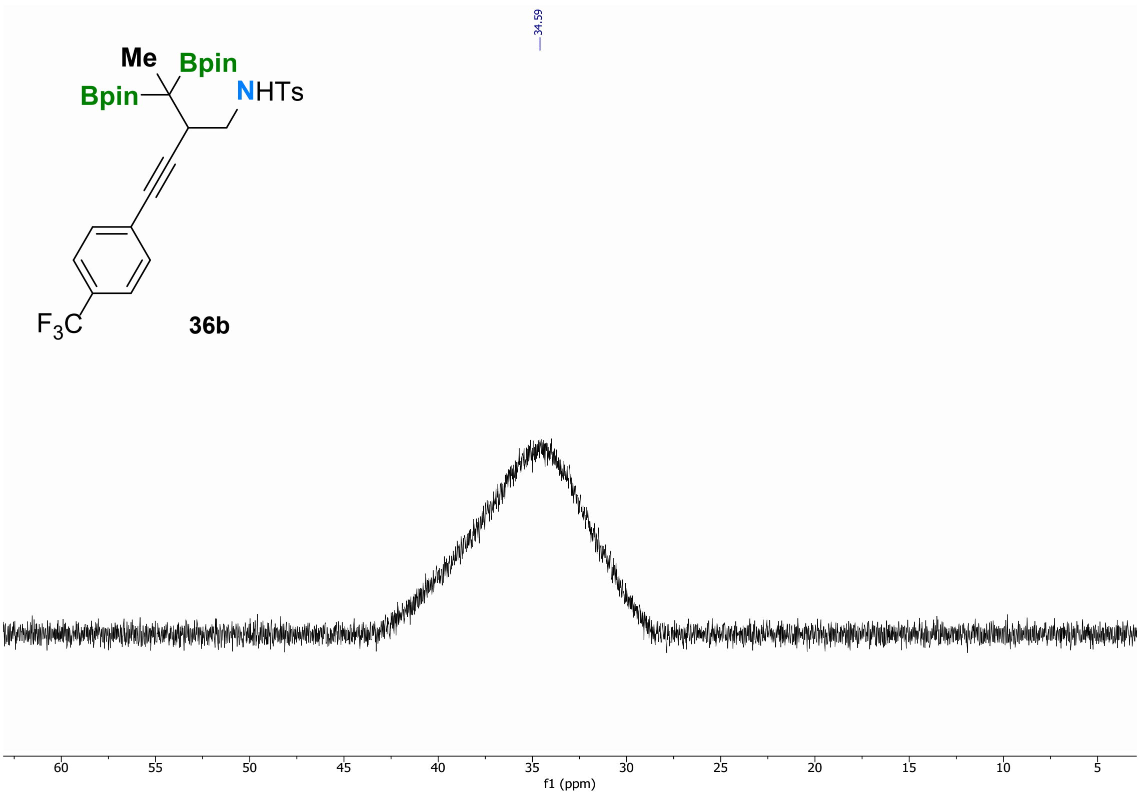

^{19}F NMR {^{1}H} (376.5 MHz, $CDCl_3$)

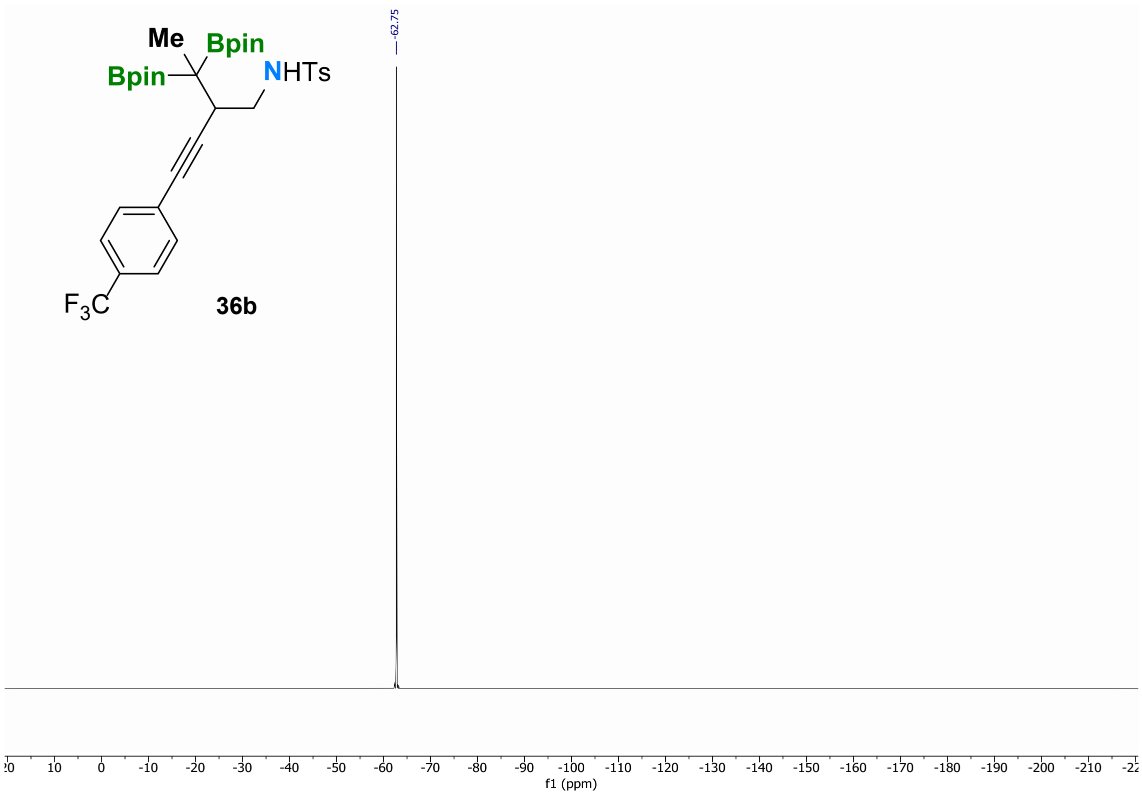

***N*-(2-(bis(4,4,5,5-tetramethyl-1,3,2-dioxaborolan-2-yl)(trimethylsilyl)methyl)-4-(4-(trifluoromethyl)phenyl)but-3-yn-1-yl)-4-methylbenzenesulfonamide (36e)**

^{1}H NMR (400 MHz, $CDCl_3$)

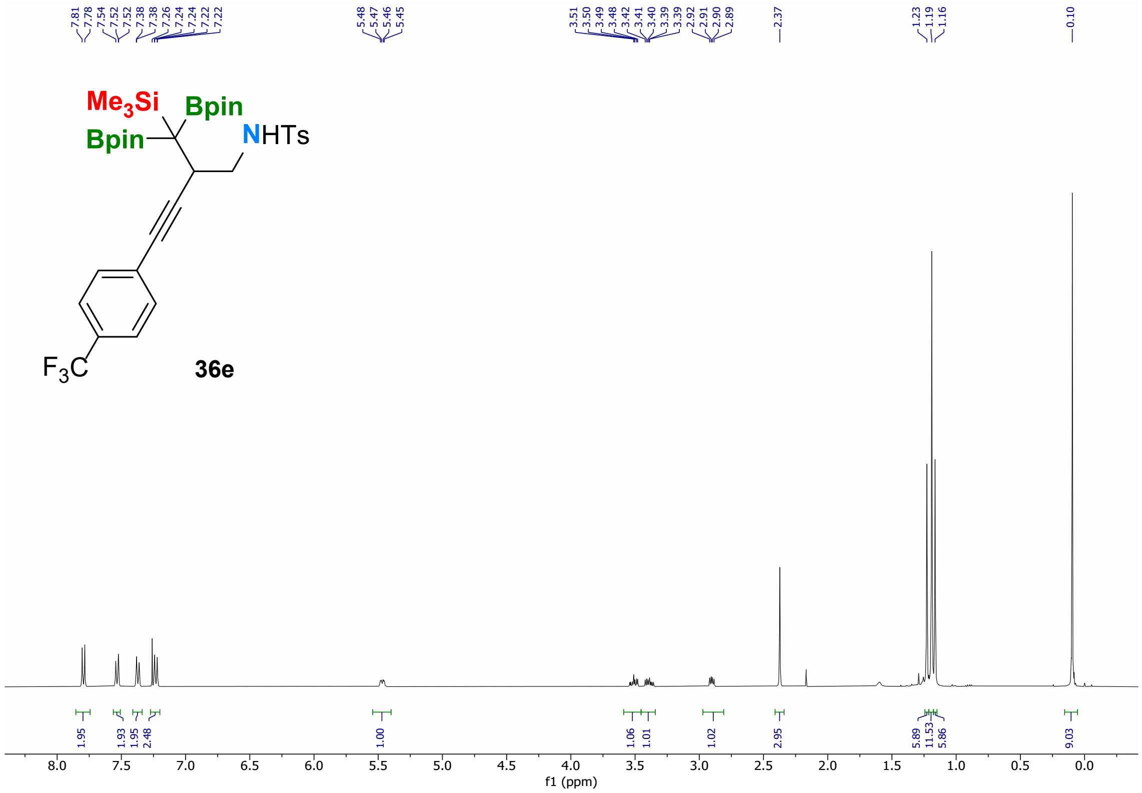

^{13}C NMR {^{1}H} (125 MHz, $CDCl_3$)

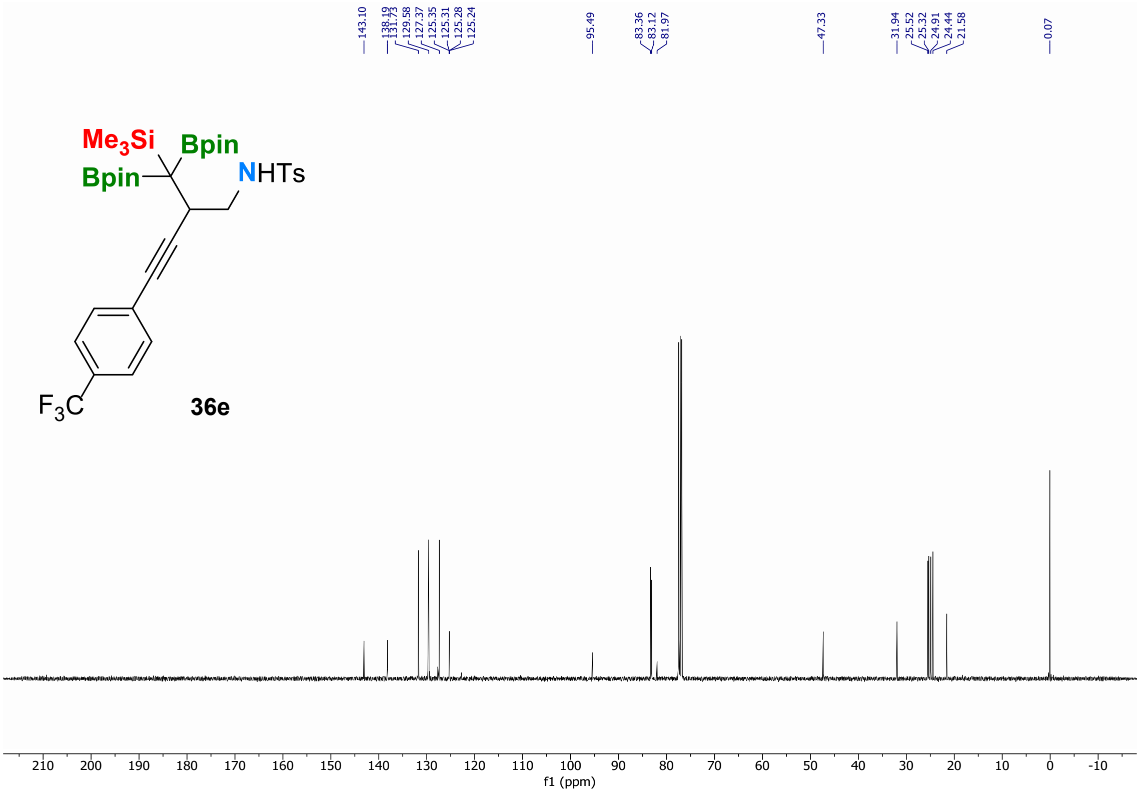

^{11}B NMR (128.3 MHz, $CDCl_3$)

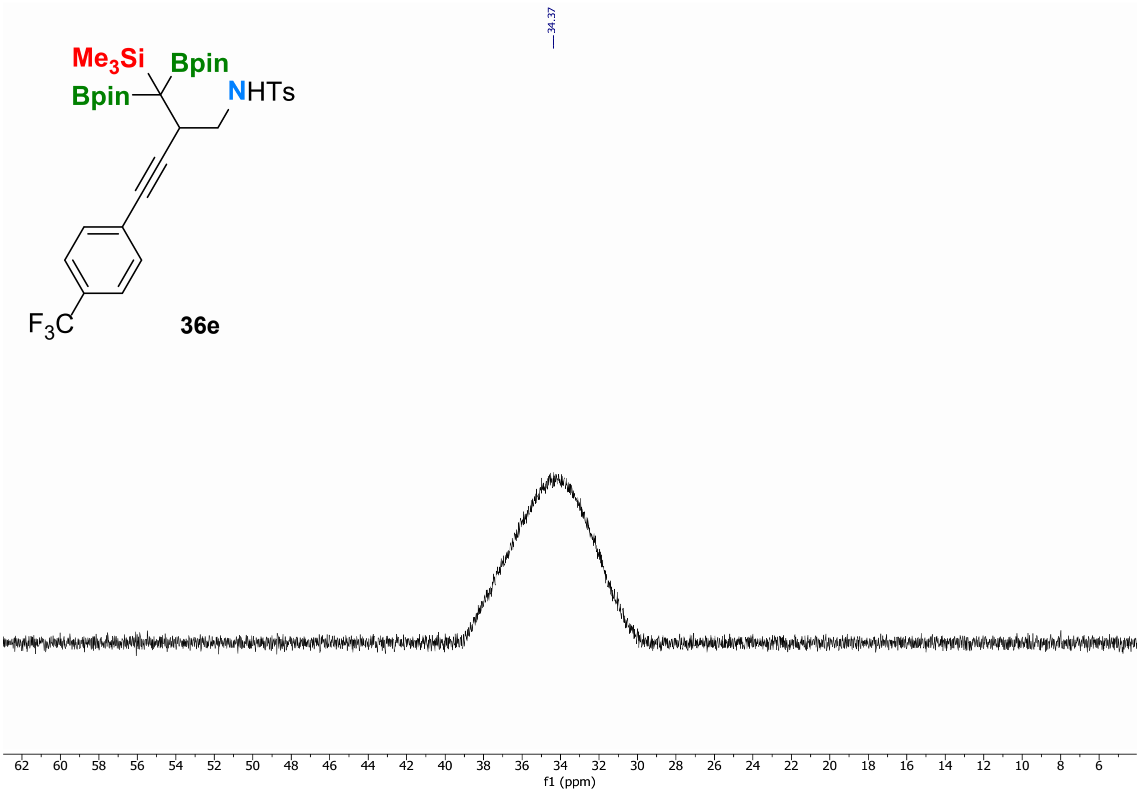

^{19}F NMR {^{1}H} (376.5 MHz, $CDCl_3$)

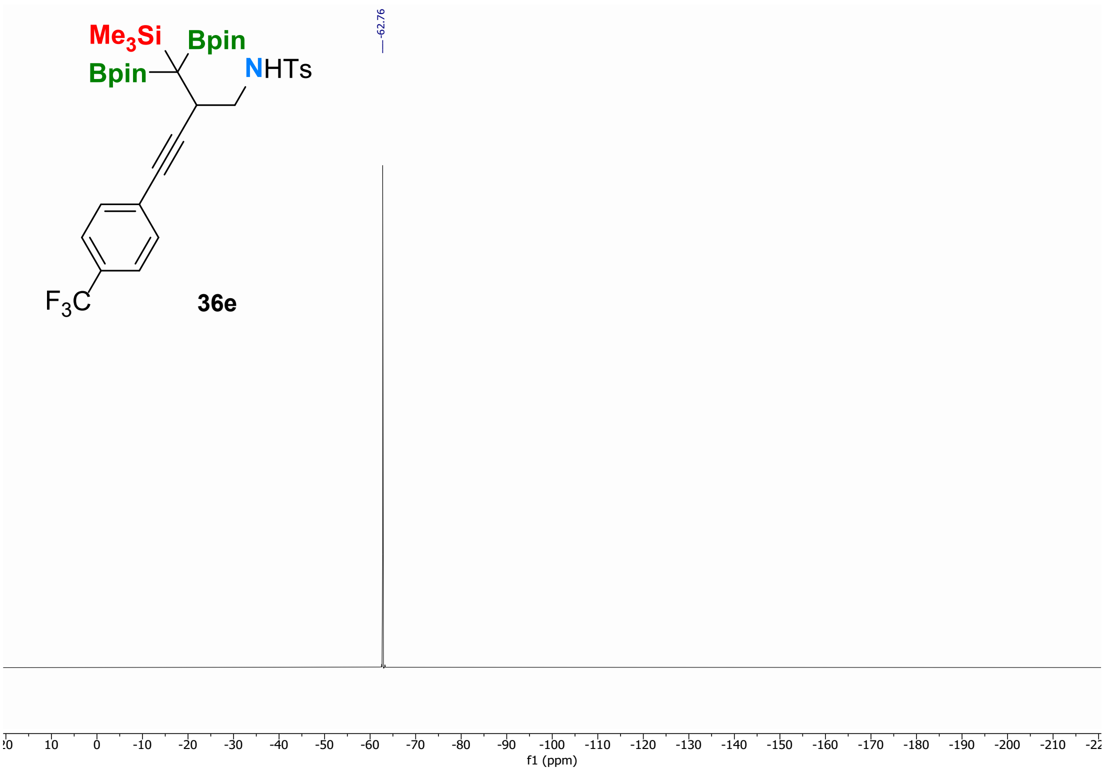

***N*-(5,5-bis(4,4,5,5-tetramethyl-1,3,2-dioxaborolan-2-yl)-1-(4-(trifluoromethyl)phenyl)-5-(trimethylsilyl)pent-1-yn-3-yl)-4-methylbenzenesulfonamide (37e)**

^{1}H NMR (400 MHz, $CDCl_3$)

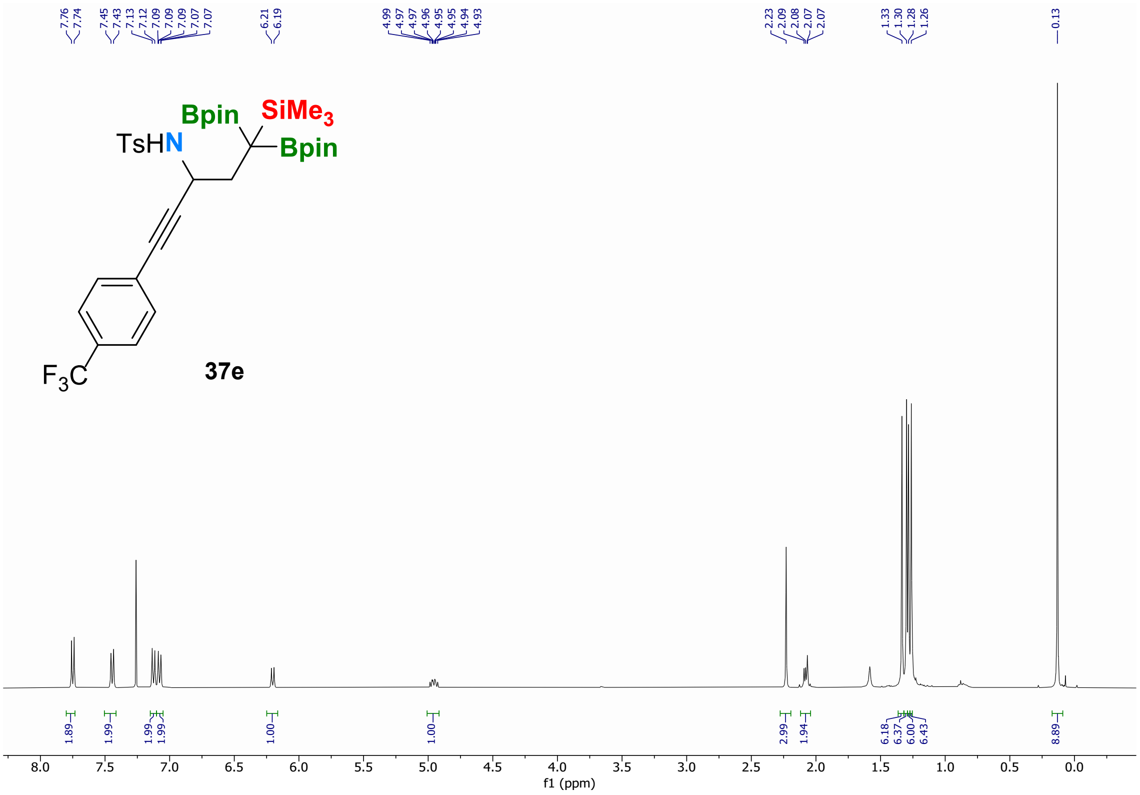

^{13}C NMR {^{1}H} (125 MHz, $CDCl_3$)

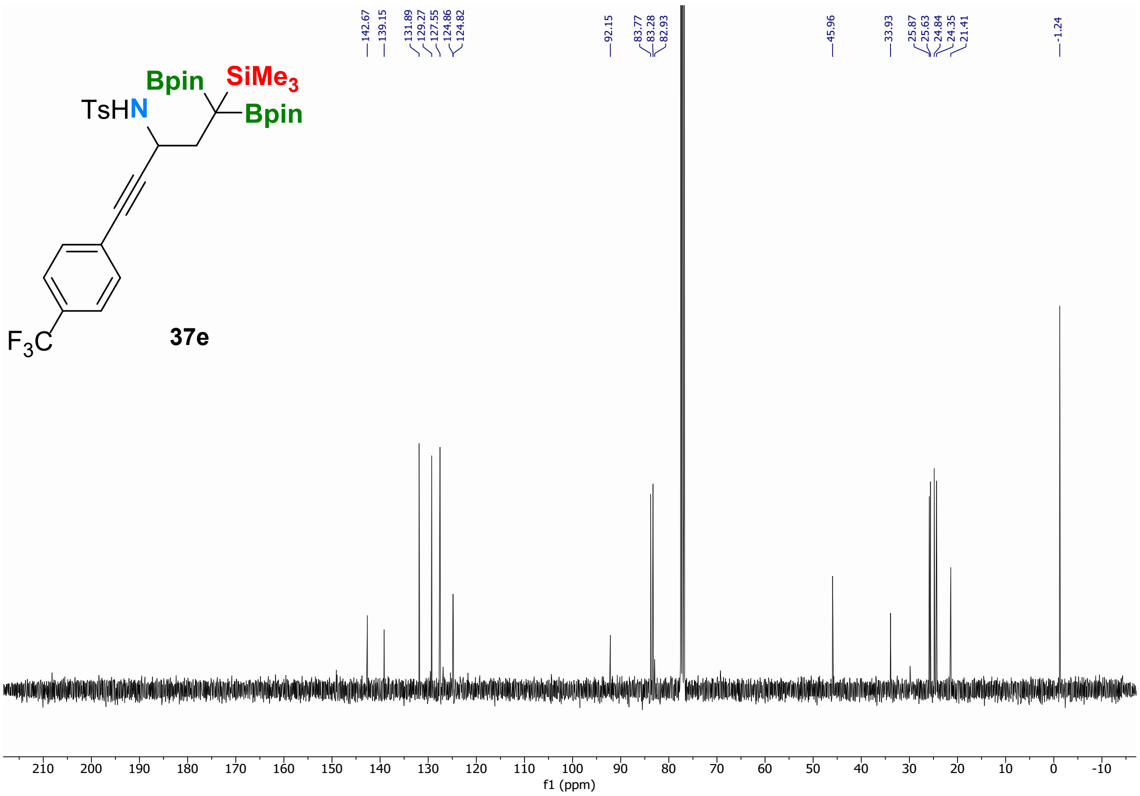

^{11}B NMR (128.3 MHz, $CDCl_3$)

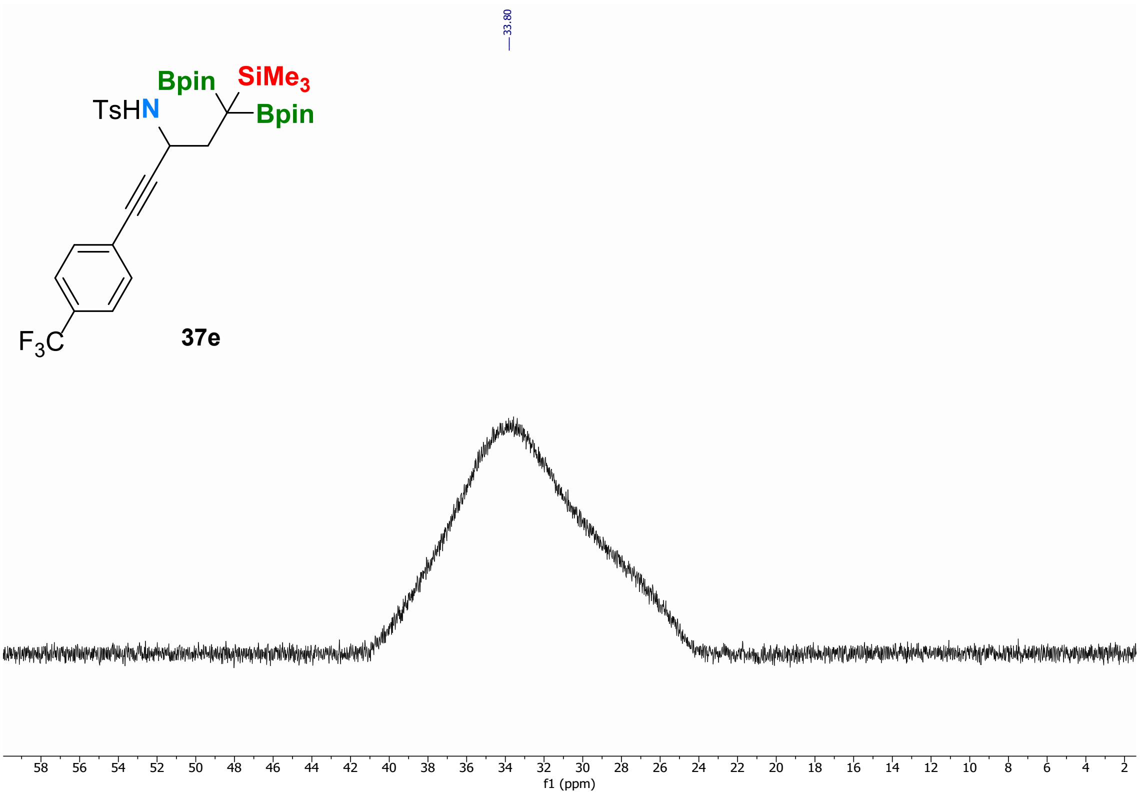

^{19}F NMR {^{1}H} (376.5 MHz, $CDCl_3$)

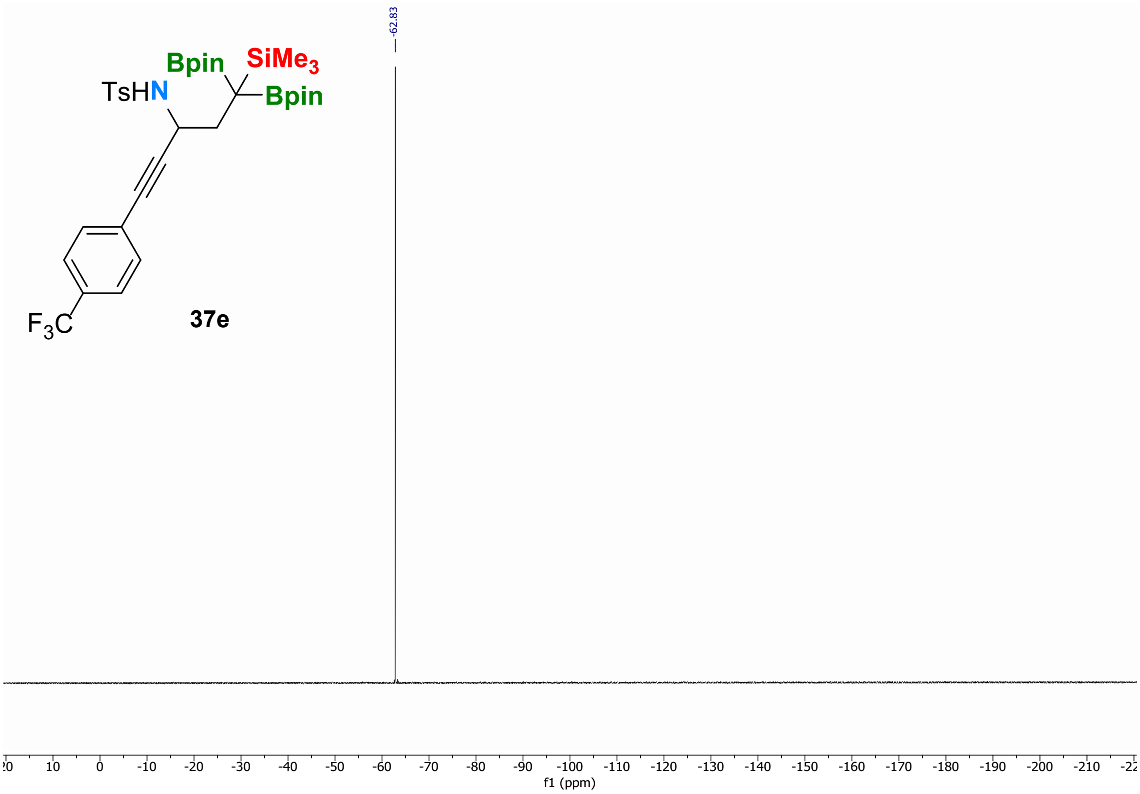

S15. NMR spectra of substituted 2,3-dihydropyrroles

3-(1,1-bis(4,4,5,5-tetramethyl-1,3,2-dioxaborolan-2-yl)ethyl)-3-methyl-5-phenyl-1-tosyl-2,3-dihydro-1H-pyrrole (29b)

^{1}H NMR (400 MHz, $CDCl_3$)

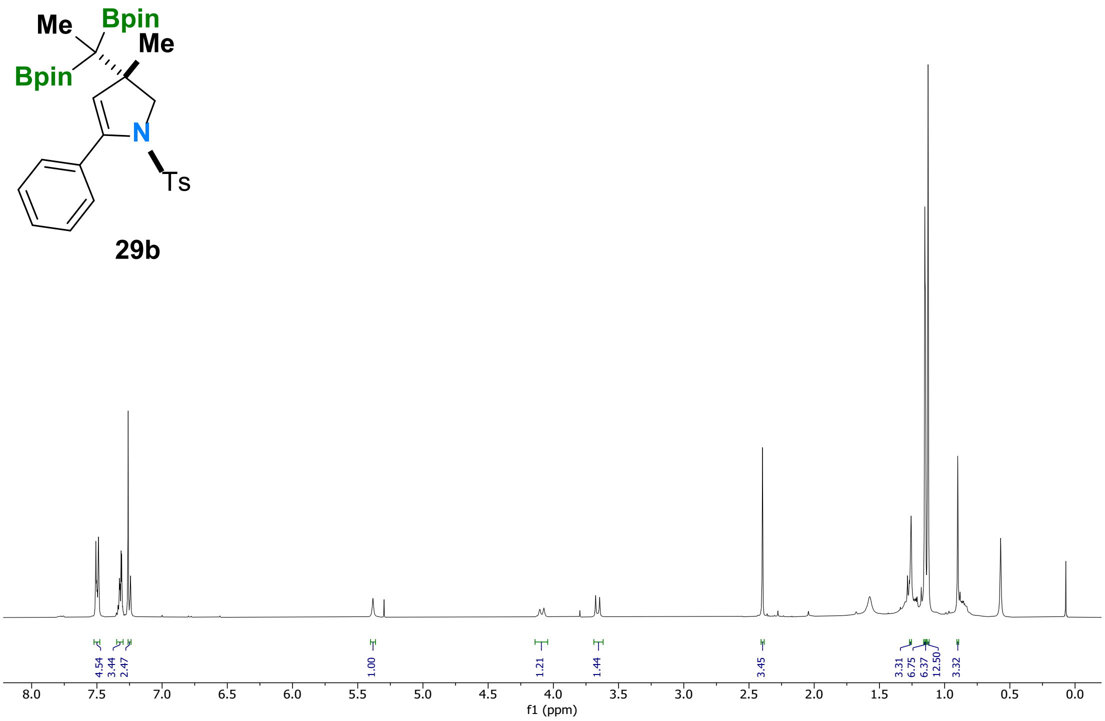

^{13}C NMR {^{1}H} (125 MHz, $CDCl_3$)

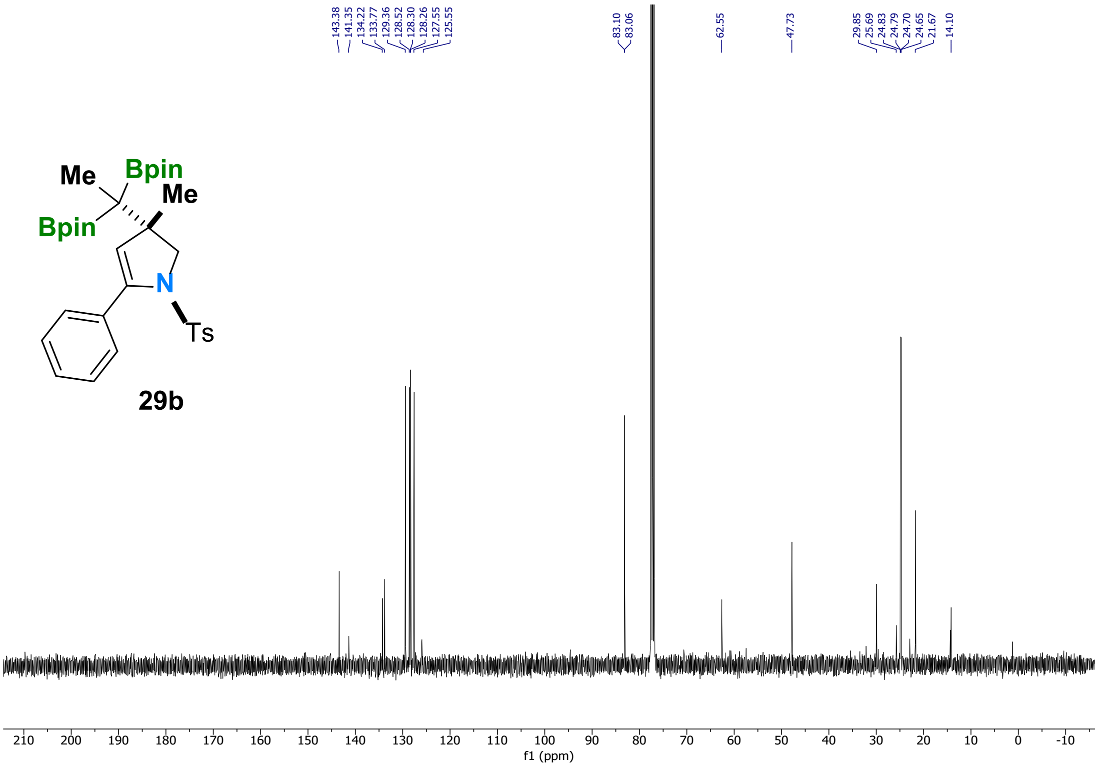

^{11}B NMR (128.3 MHz, $CDCl_3$)

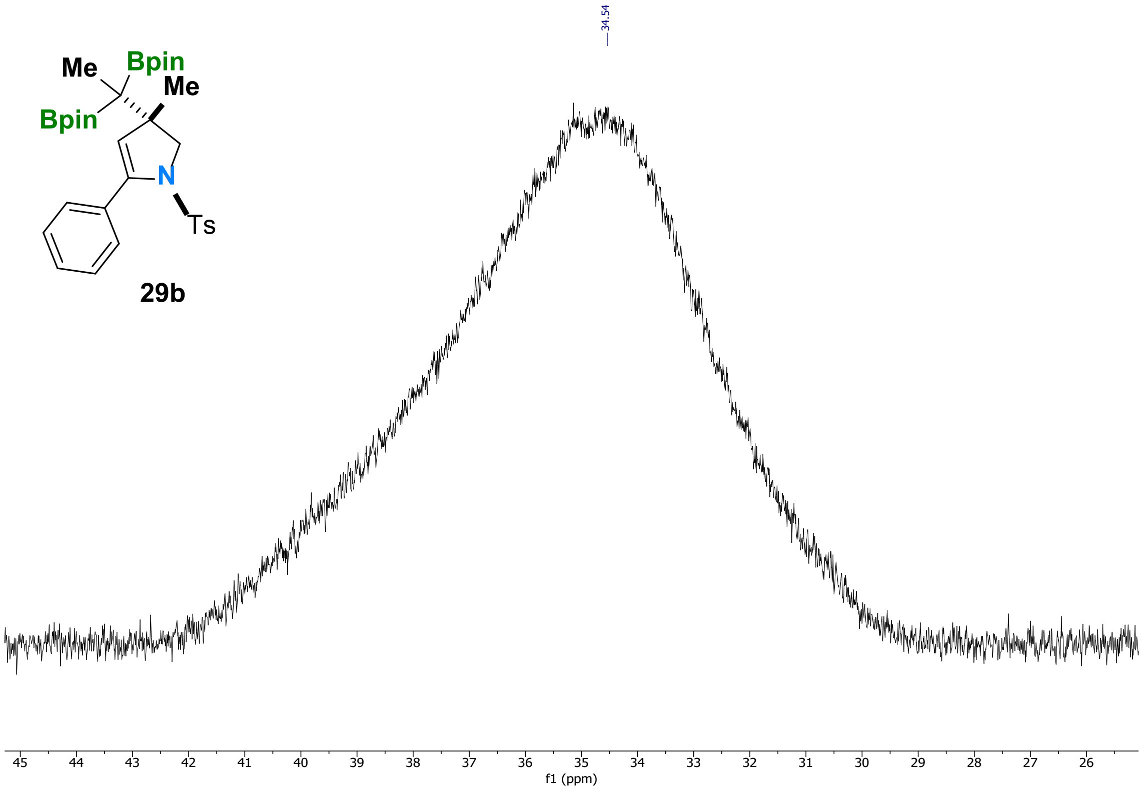

3-(bis(4,4,5,5-tetramethyl-1,3,2-dioxaborolan-2-yl)(trimethylsilyl)methyl)-3-methyl-5-phenyl-1-tosyl-2,3-dihydro-1*H*-pyrrole (29e)

^{1}H NMR (400 MHz, $CDCl_3$)

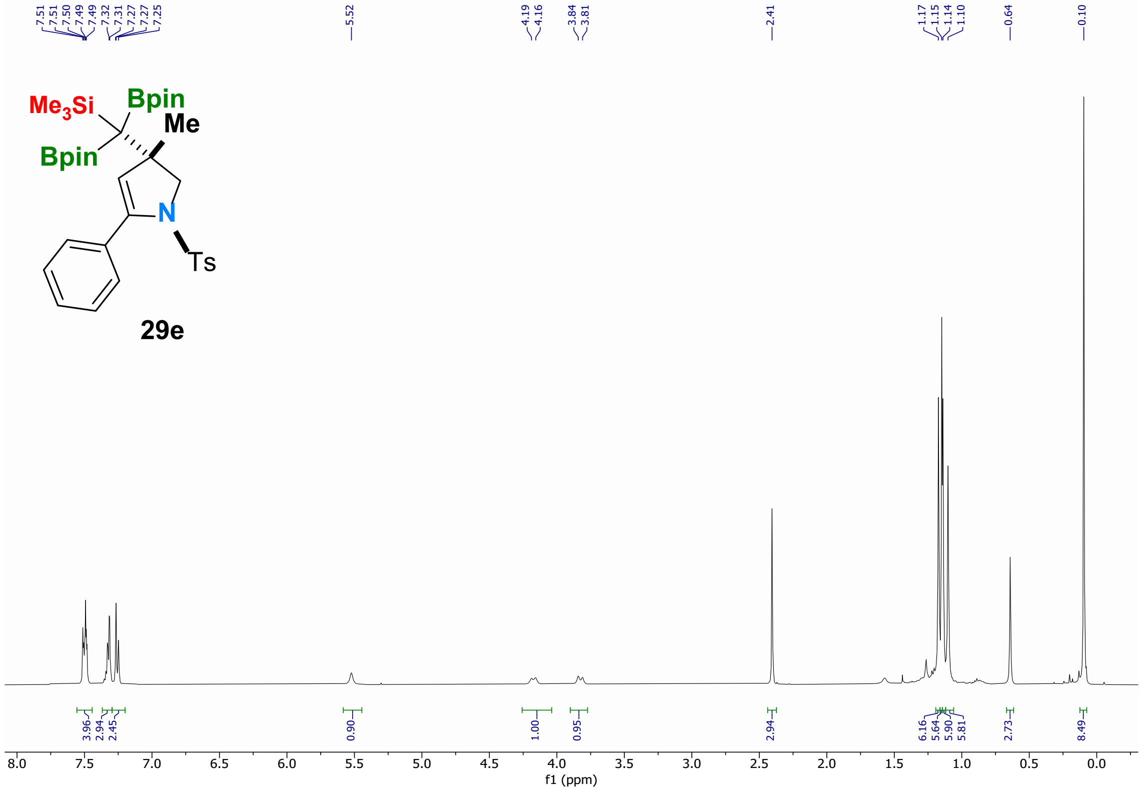

^{13}C NMR {^{1}H} (125 MHz, $CDCl_3$)

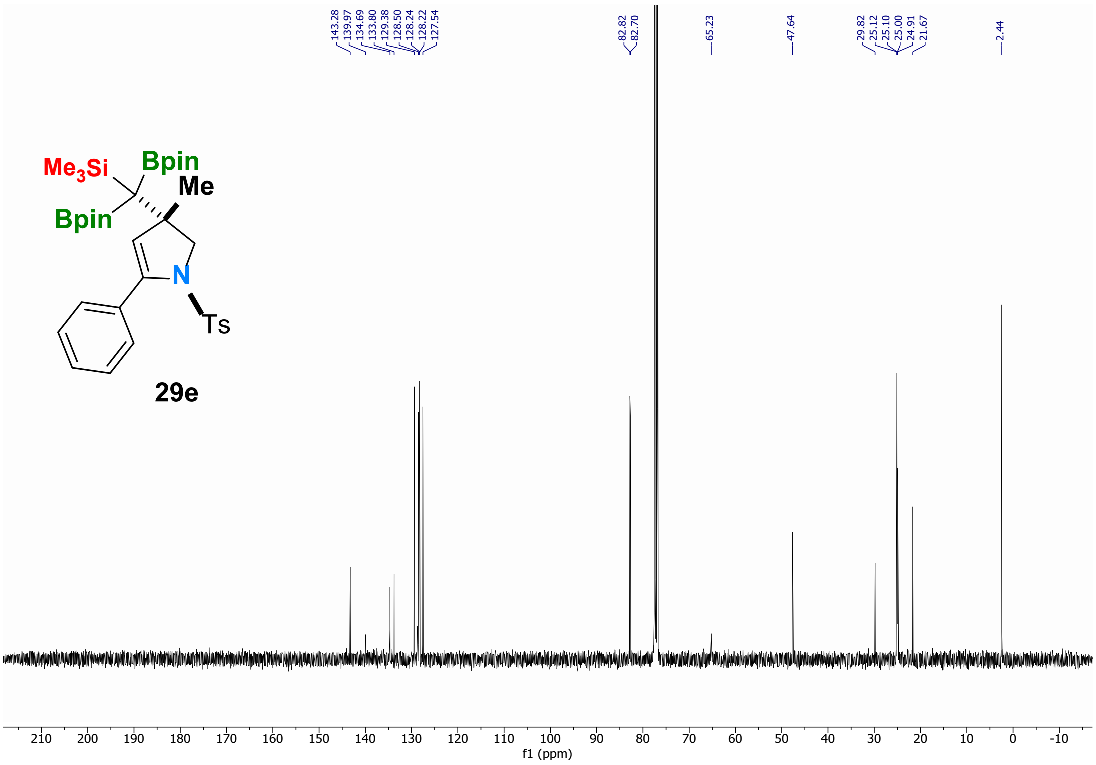

^{11}B NMR (128.3 MHz, $CDCl_3$)

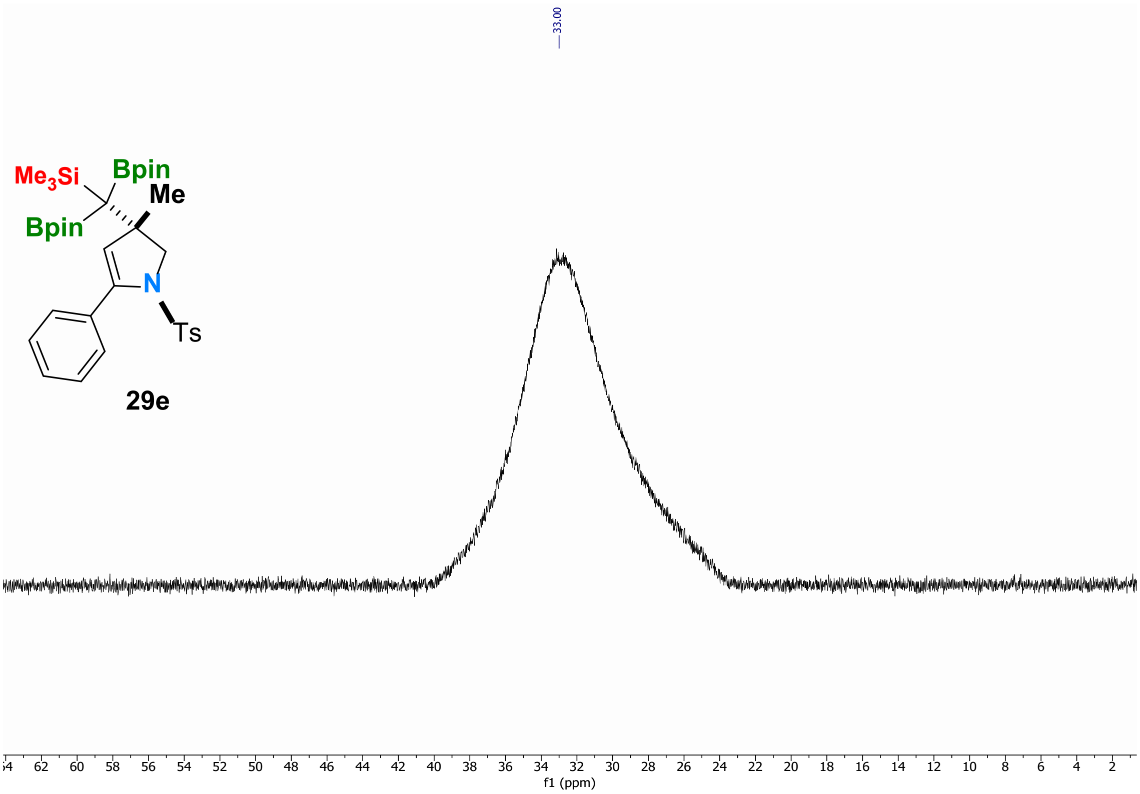

3-(bis(4,4,5,5-tetramethyl-1,3,2-dioxaborolan-2-yl)(trimethylsilyl)methyl)-3-methyl-5-(naphthalen-2-yl)-1-tosyl-2,3-dihydro-1*H*-pyrrole (31e)

^{1}H NMR (400 MHz, $CDCl_3$)

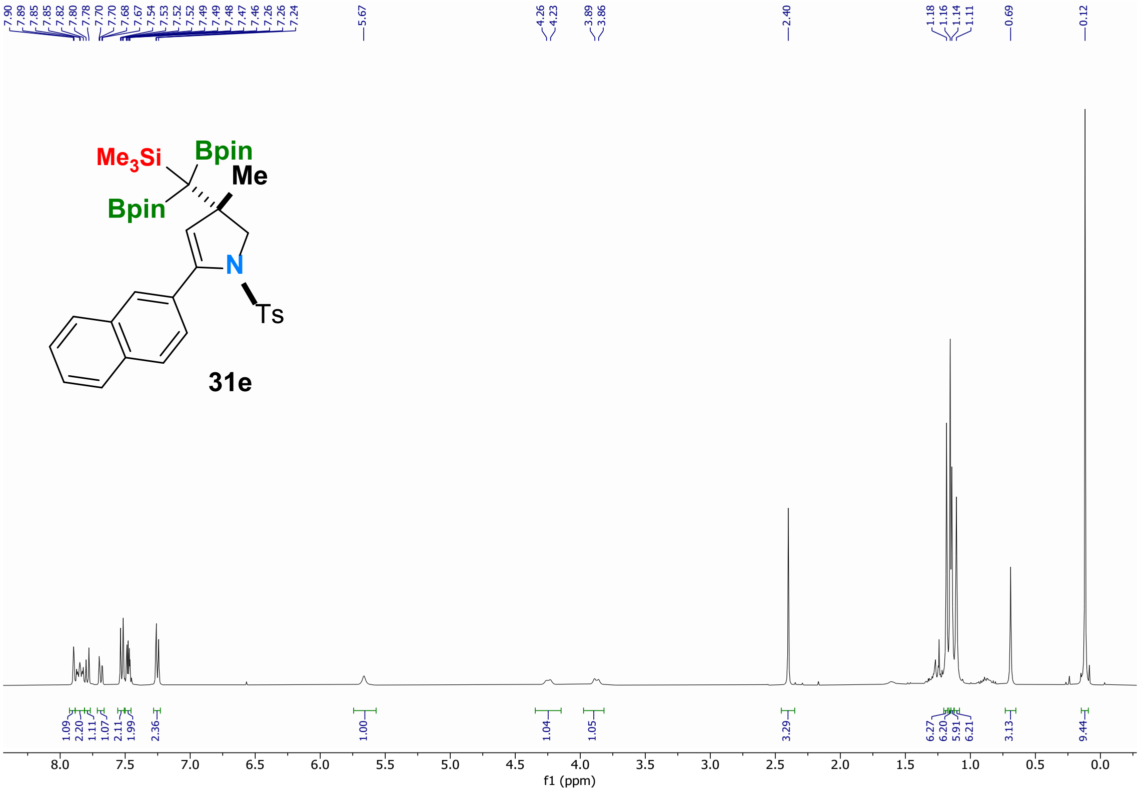

^{13}C NMR {^{1}H} (125 MHz, $CDCl_3$)

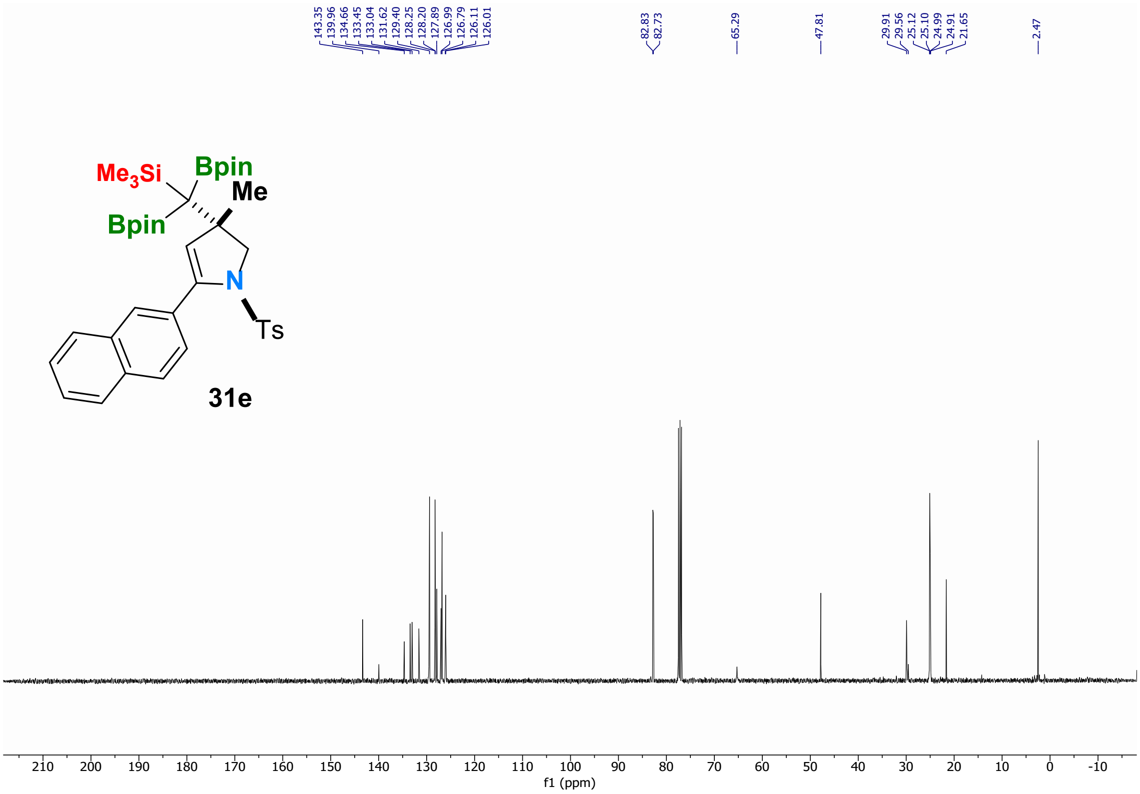

^{11}B NMR (128.3 MHz, $CDCl_3$)

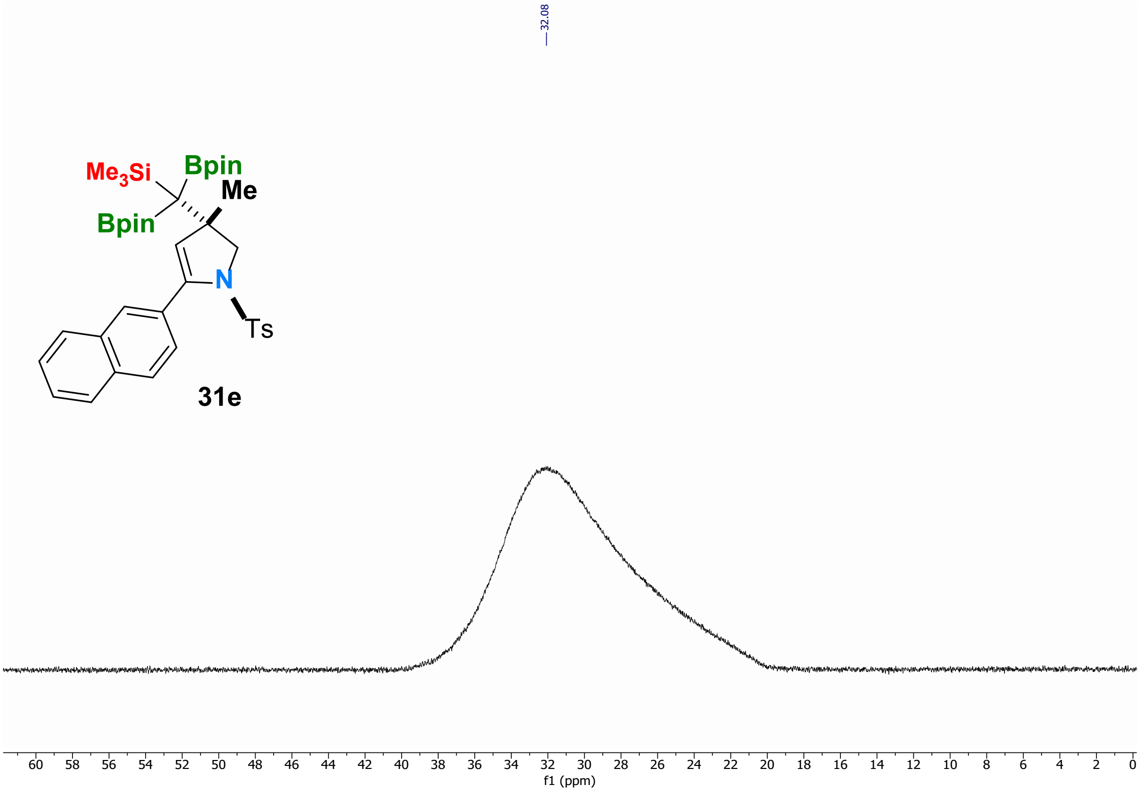

S16. NMR spectra of (*Z*)-2-alkylidene-1-tosylazetidines

(Z)-2-benzylidene-3-(1,1-bis(4,4,5,5-tetramethyl-1,3,2-dioxaborolan-2-yl)ethyl)-1-tosylazetidine (30b)

^{1}H NMR (400 MHz, CDCl$_3$)

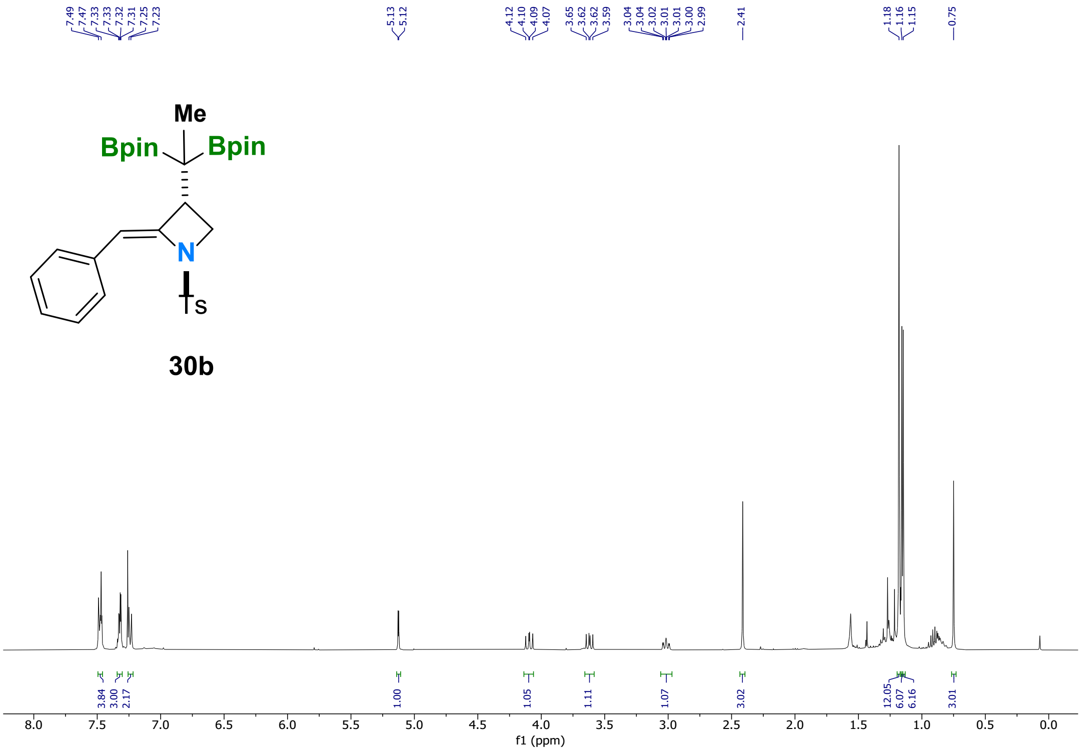

^{13}C NMR {^{1}H} (125 MHz, CDCl$_3$)

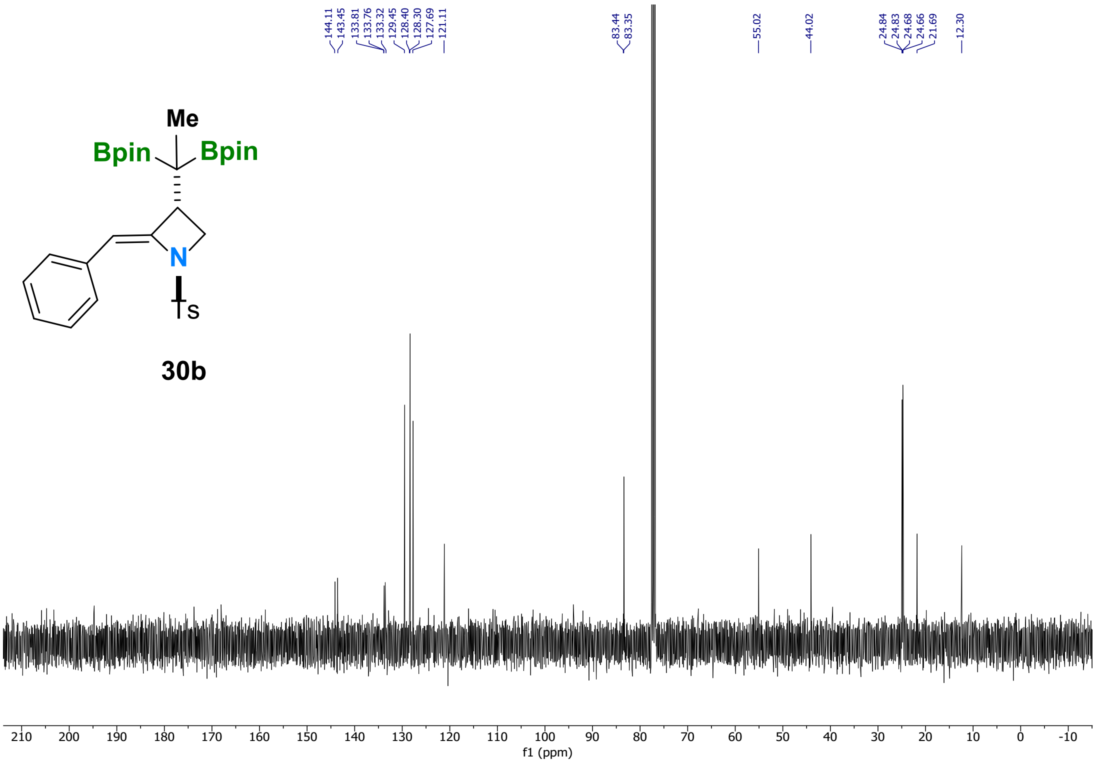

^{11}B NMR (128.3 MHz, $CDCl_3$)

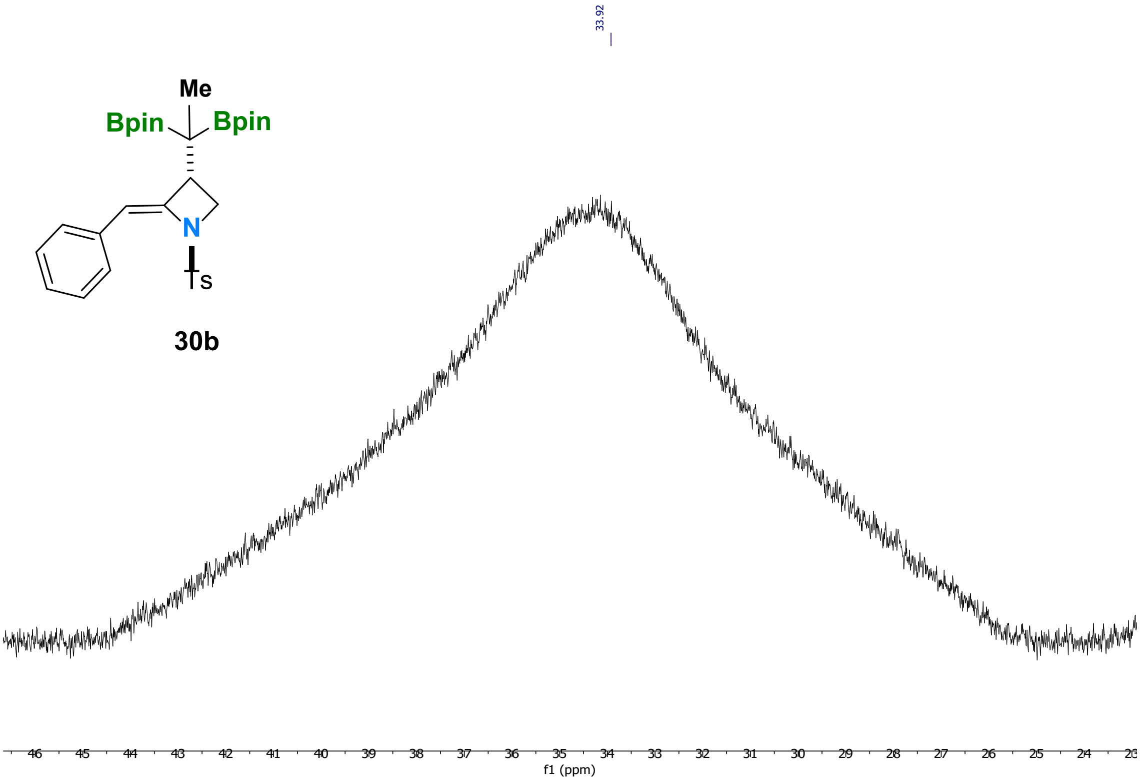

(*Z*)-2-benzylidene-3-(bis(4,4,5,5-tetramethyl-1,3,2-dioxaborolan-2-yl)(trimethylsilyl)methyl)-1-tosylazetidine (30e)

^{1}H NMR (400 MHz, $CDCl_3$)

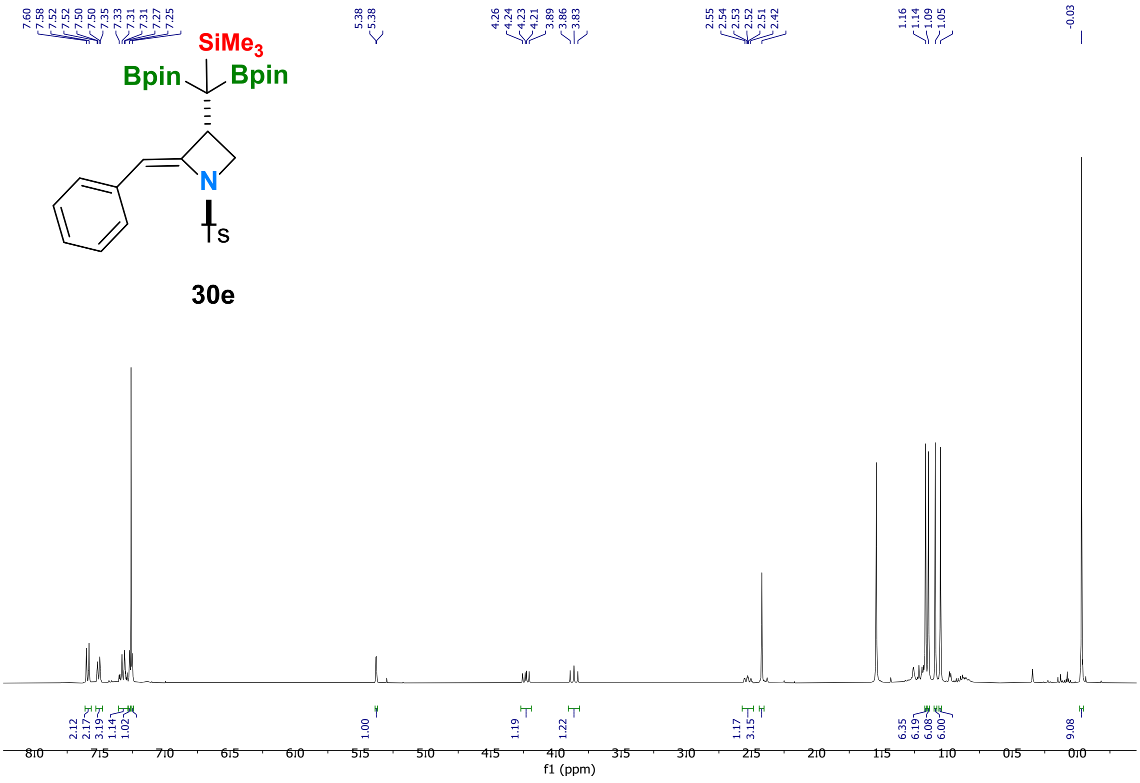

^{13}C NMR {^{1}H} (125 MHz, $CDCl_3$)

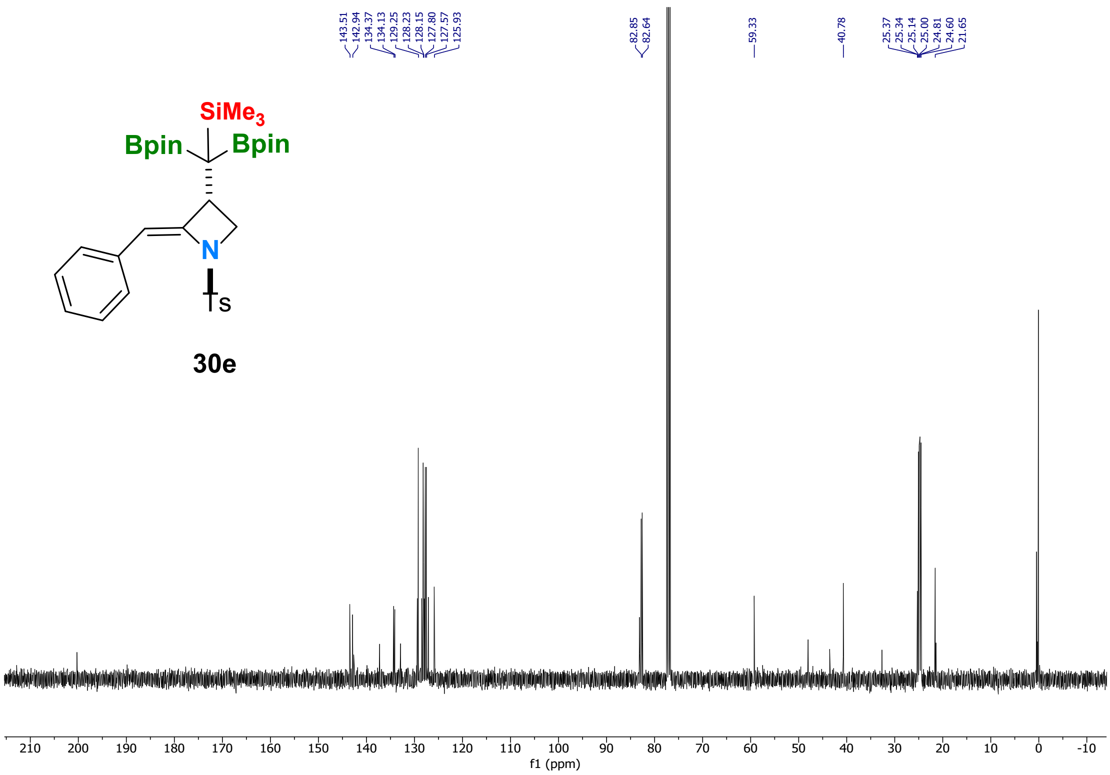

30e

^{11}B NMR (128.3 MHz, $CDCl_3$)

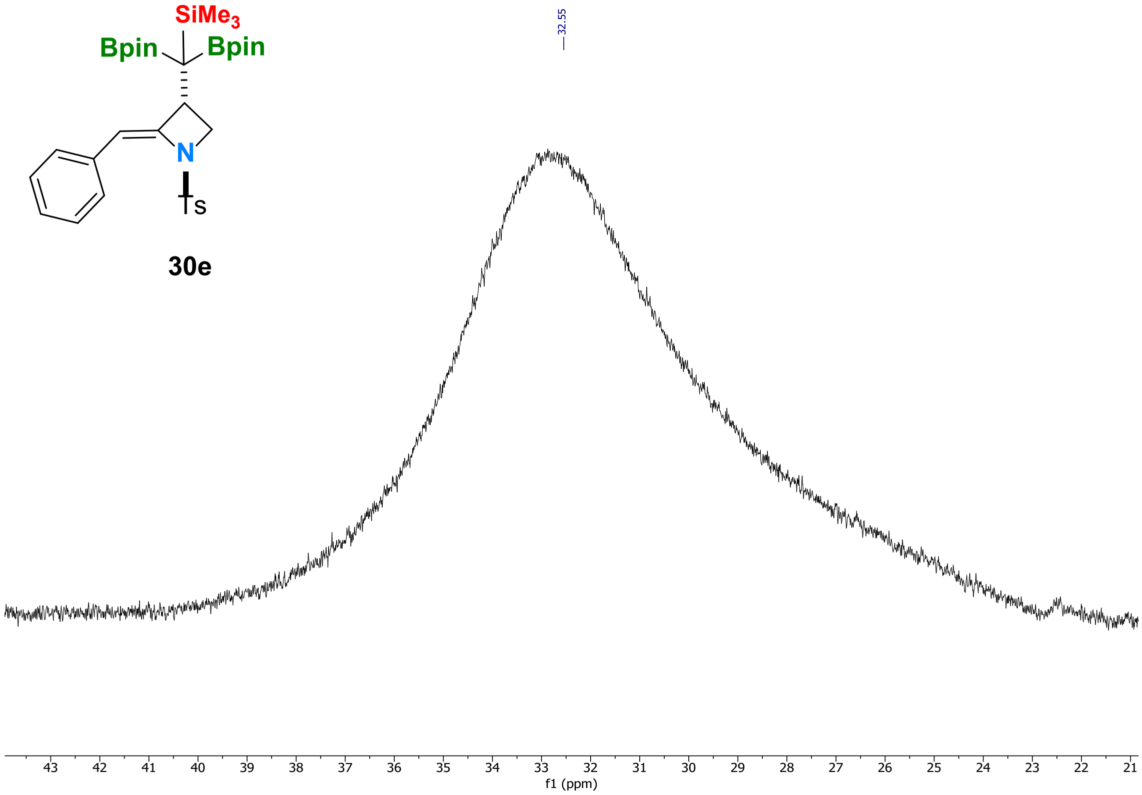

30e

(*Z*)-3-(1,1-bis(4,4,5,5-tetramethyl-1,3,2-dioxaborolan-2-yl)ethyl)-2-(4-chlorobenzylidene)-1-tosylazetidine (32b)

^{1}H NMR (400 MHz, $CDCl_3$)

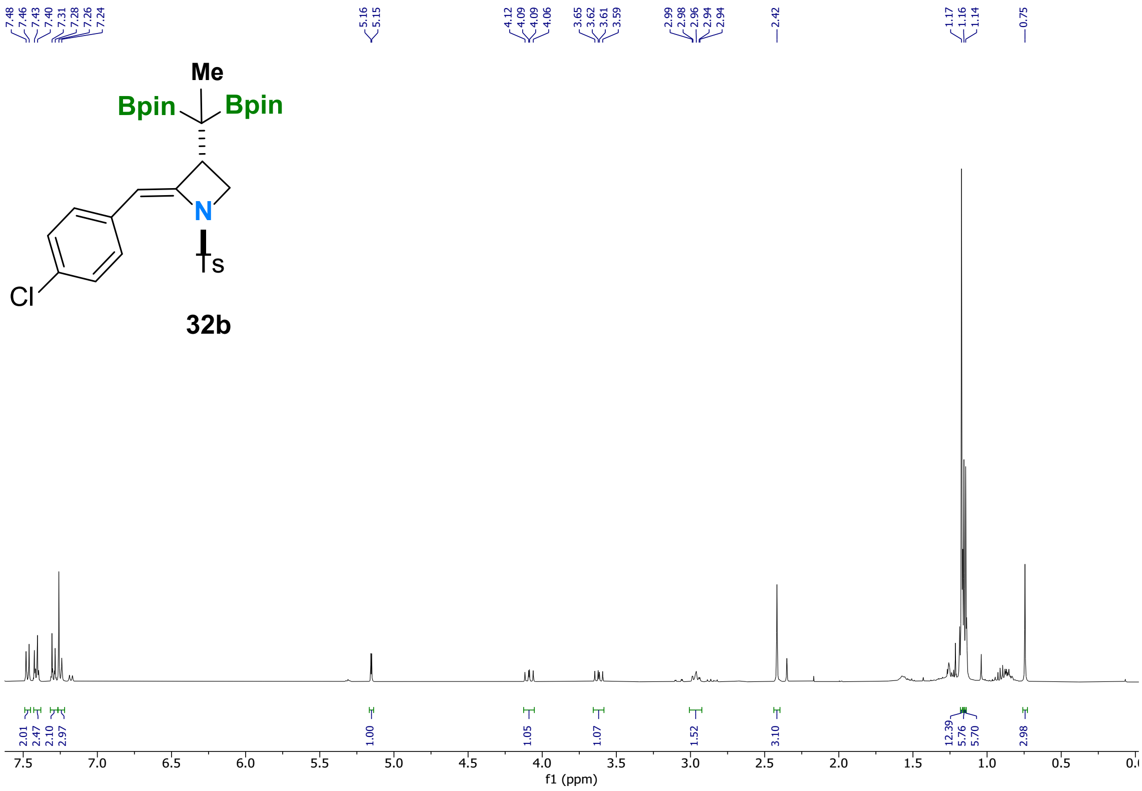

^{13}C NMR {^{1}H} (125 MHz, $CDCl_3$)

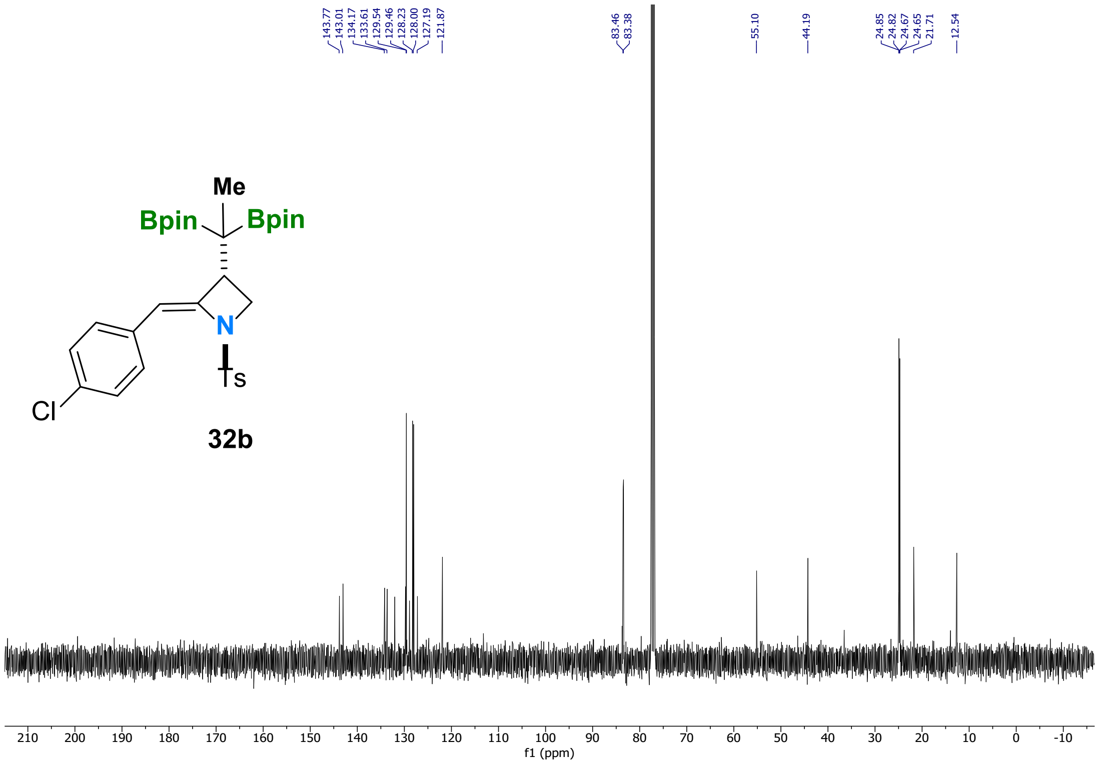

^{11}B NMR (128.3 MHz, CDCl$_3$)

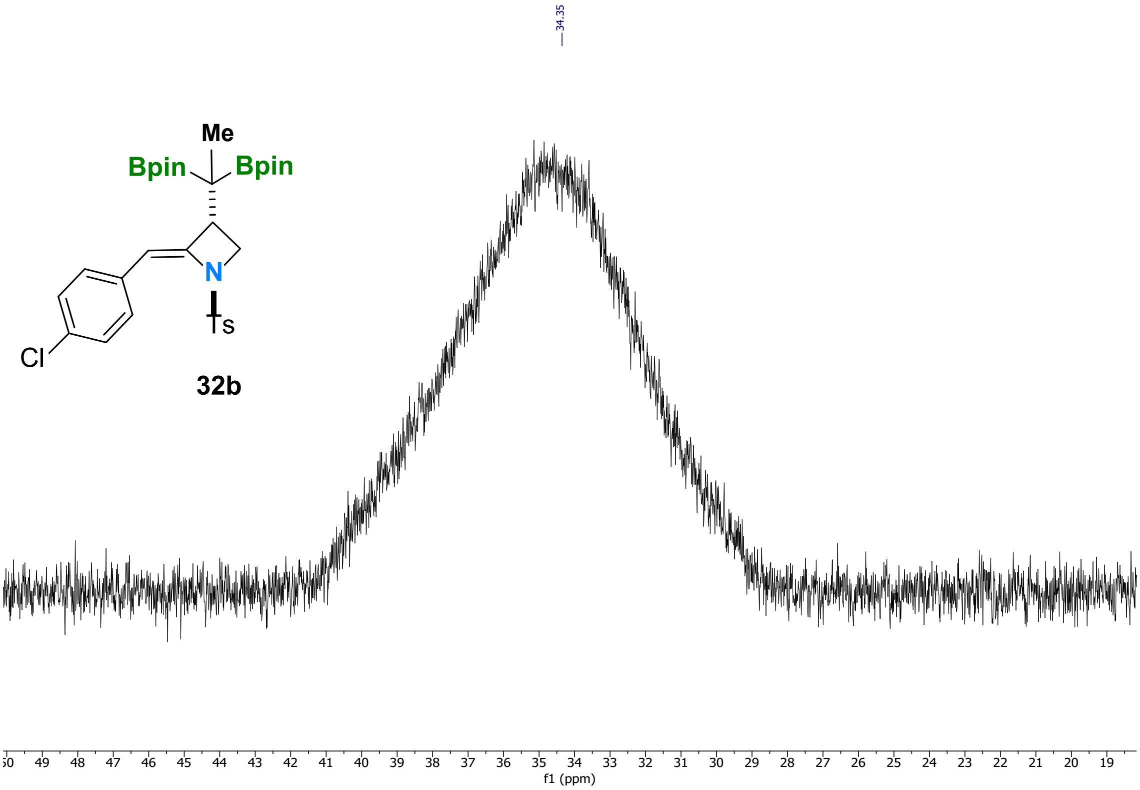

(*Z*)-3-(bis(4,4,5,5-tetramethyl-1,3,2-dioxaborolan-2-yl)(trimethylsilyl)methyl)-2-(4-chlorobenzylidene)-1-tosylazetidine (32e)

^{1}H NMR (400 MHz, CDCl$_3$)

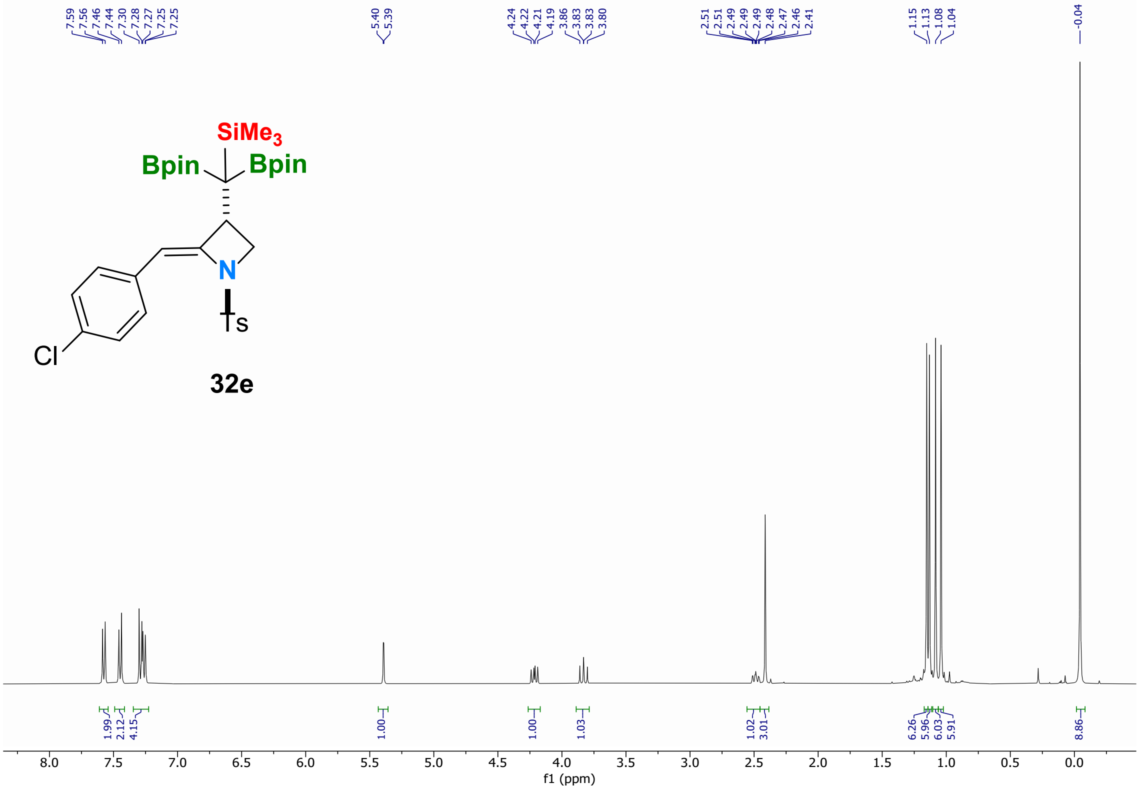

^{13}C NMR {^{1}H} (125 MHz, $CDCl_3$)

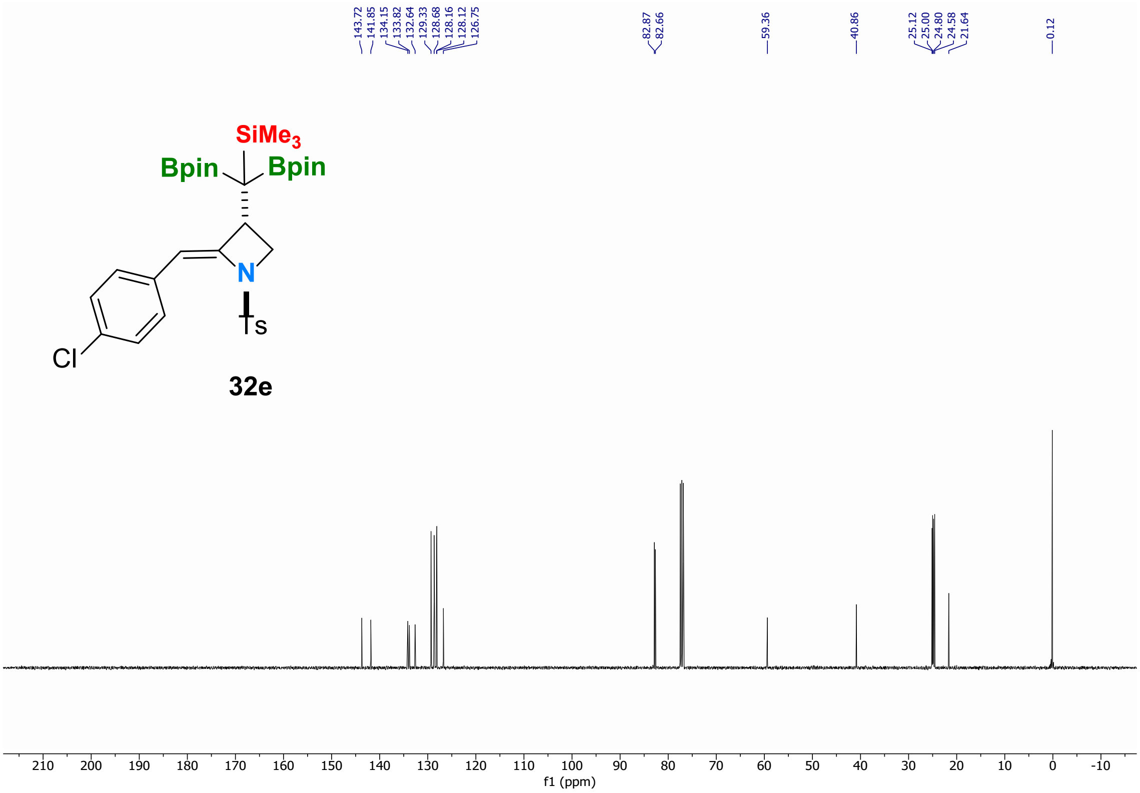

^{11}B NMR (128.3 MHz, $CDCl_3$)

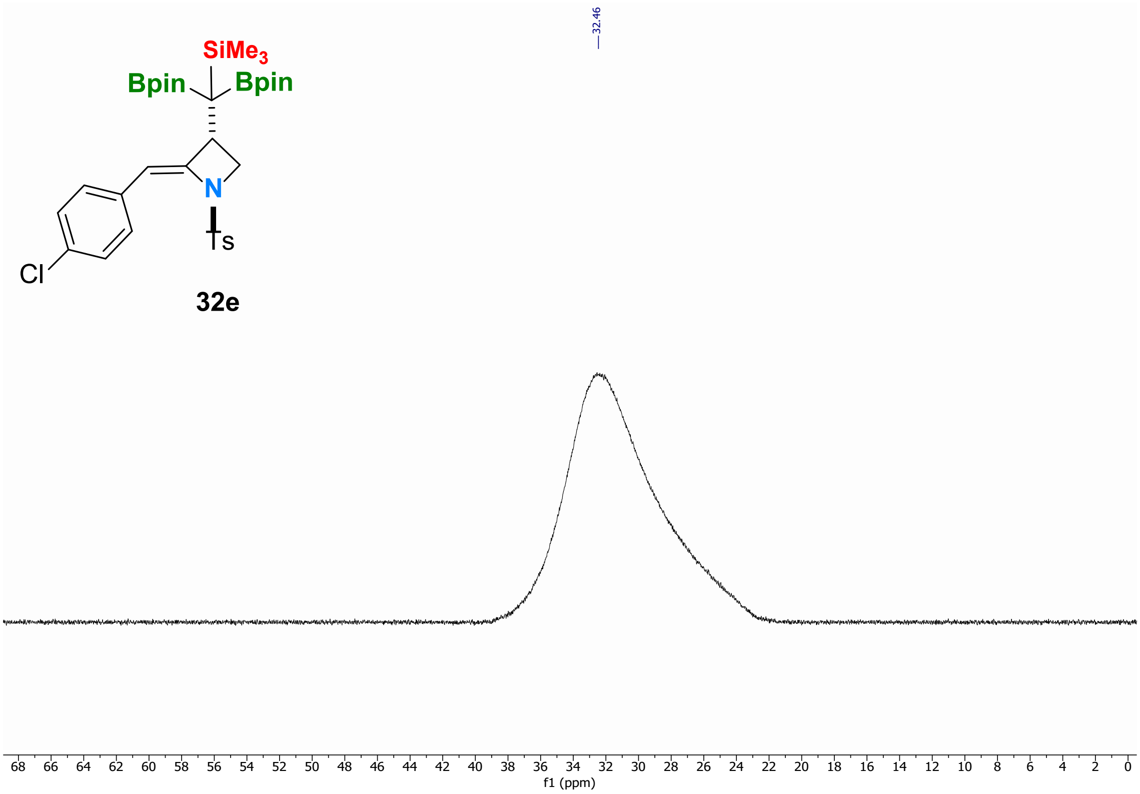

(*Z*)-3-(1,1-bis(4,4,5,5-tetramethyl-1,3,2-dioxaborolan-2-yl)ethyl)-2-(naphthalen-2-ylmethylene)-1-tosylazetidine (33b)

^{1}H NMR (400 MHz, $CDCl_3$)

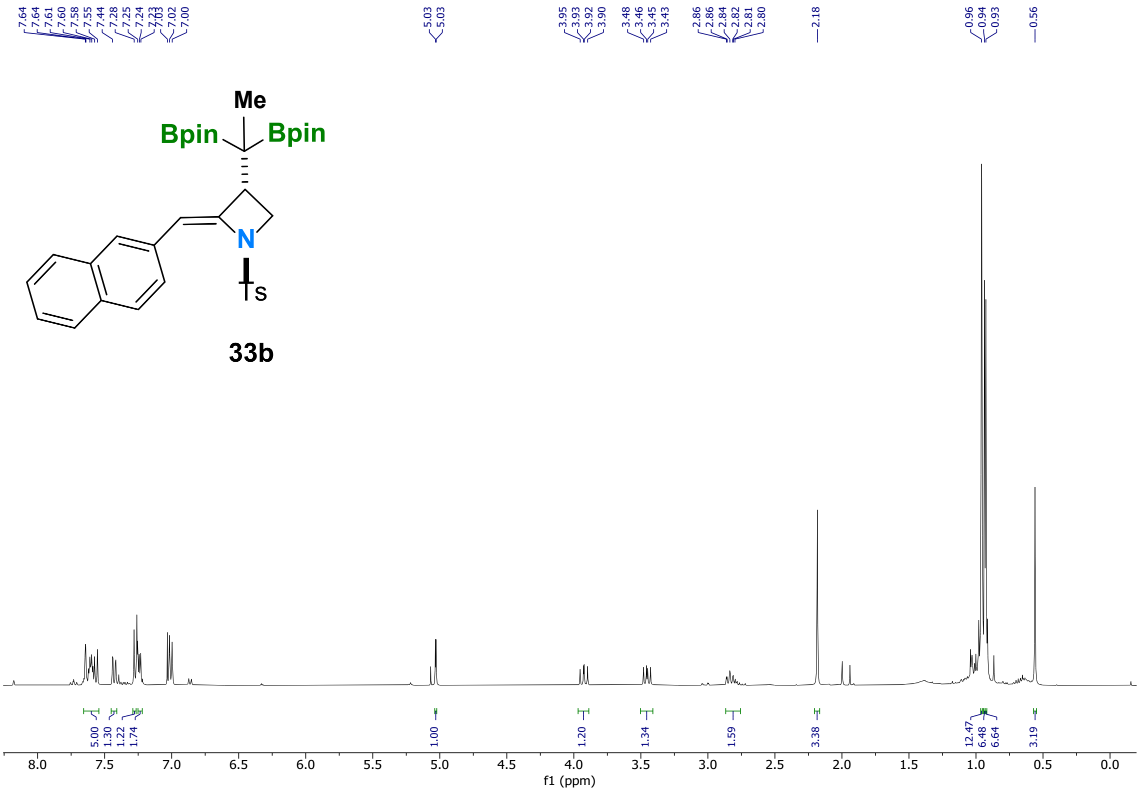

^{13}C NMR {^{1}H} (125 MHz, $CDCl_3$)

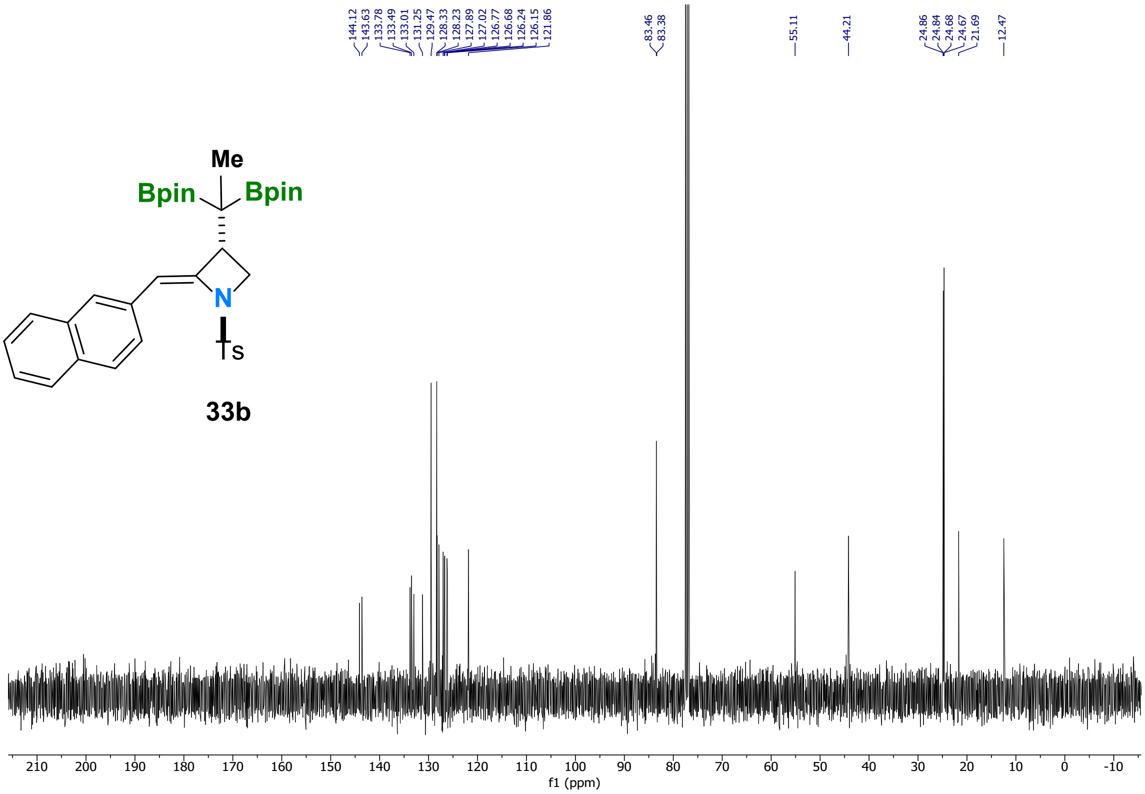

^{11}B NMR (128.3 MHz, CDCl$_3$)

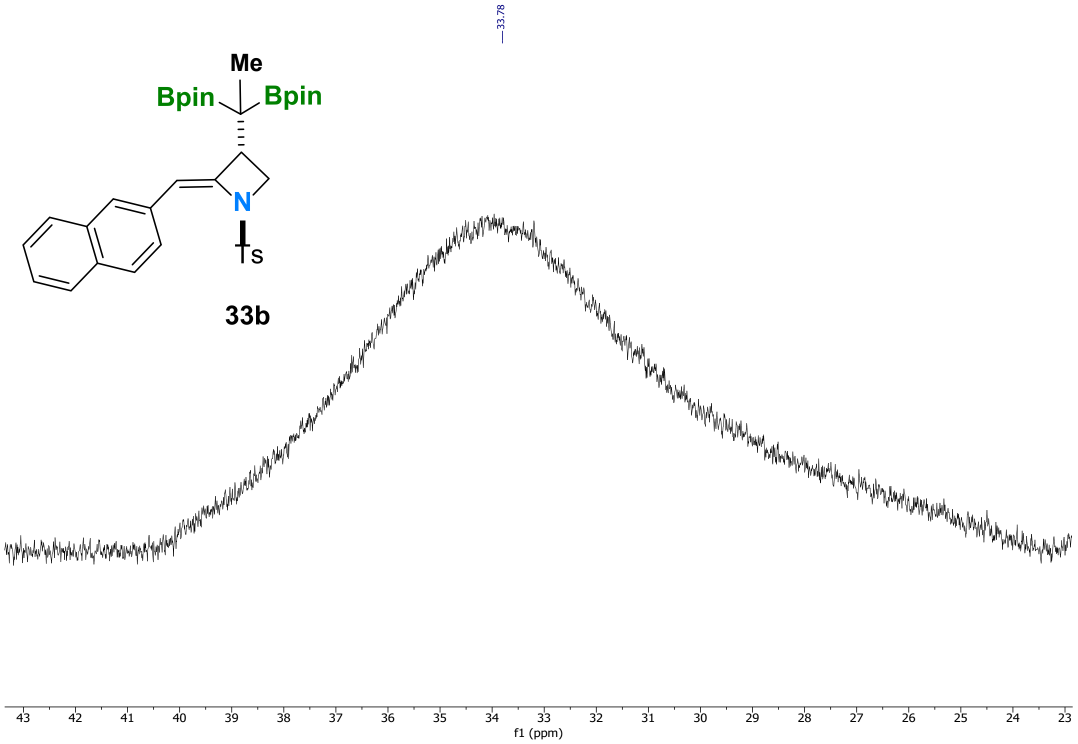

(*Z*)-3-(bis(4,4,5,5-tetramethyl-1,3,2-dioxaborolan-2-yl)(trimethylsilyl)methyl)-2-(naphthalen-2-ylmethylene)-1-tosylazetidine (33e)

^{1}H NMR (400 MHz, CDCl$_3$)

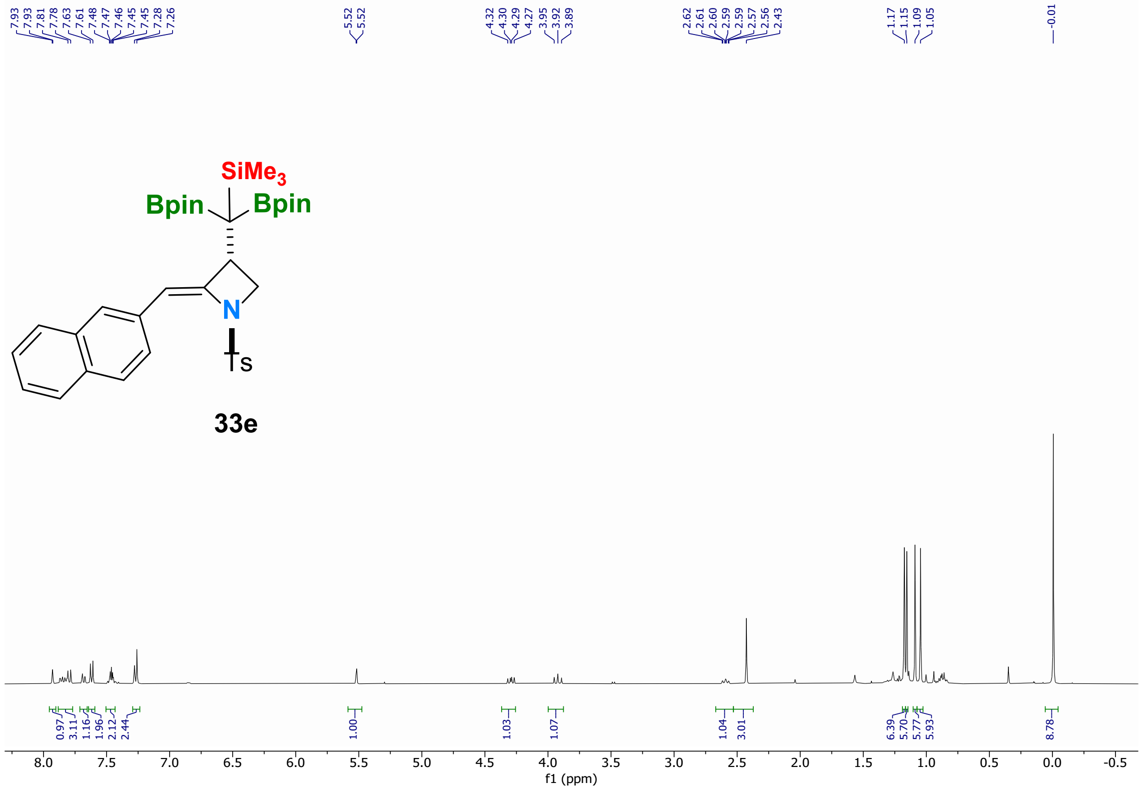

^{13}C NMR {^{1}H} (125 MHz, $CDCl_3$)

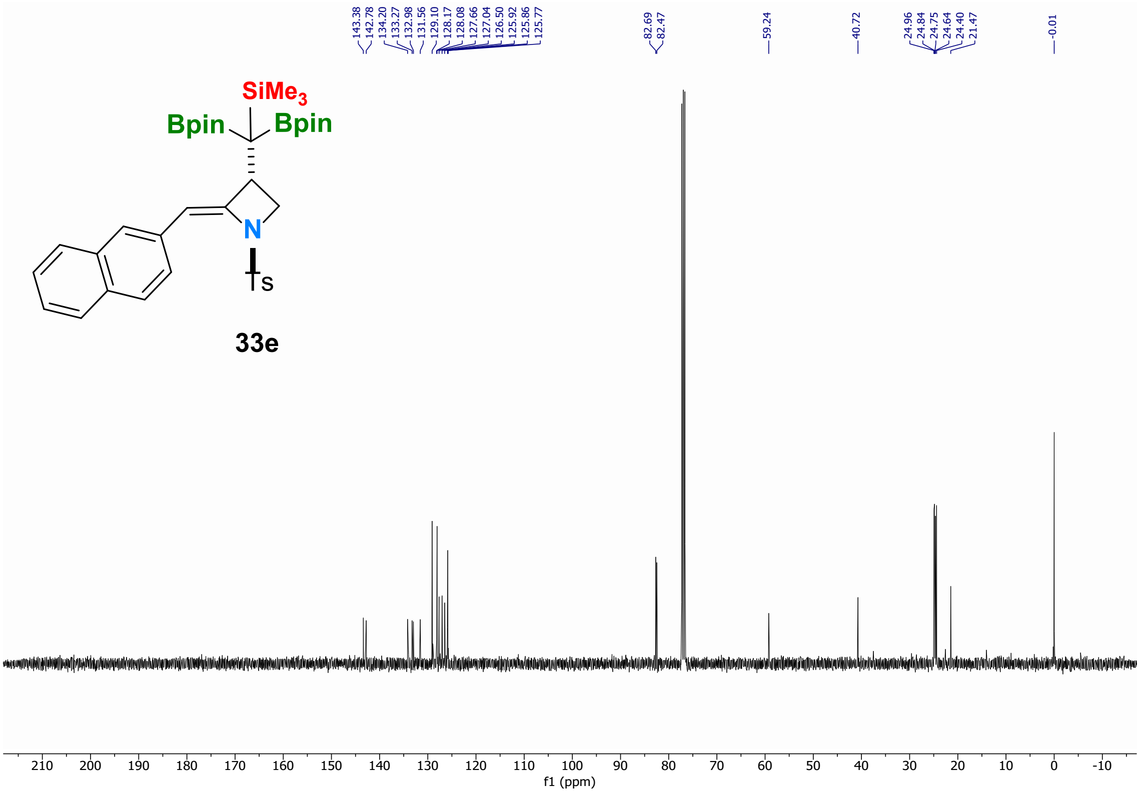

^{11}B NMR (128.3 MHz, $CDCl_3$)

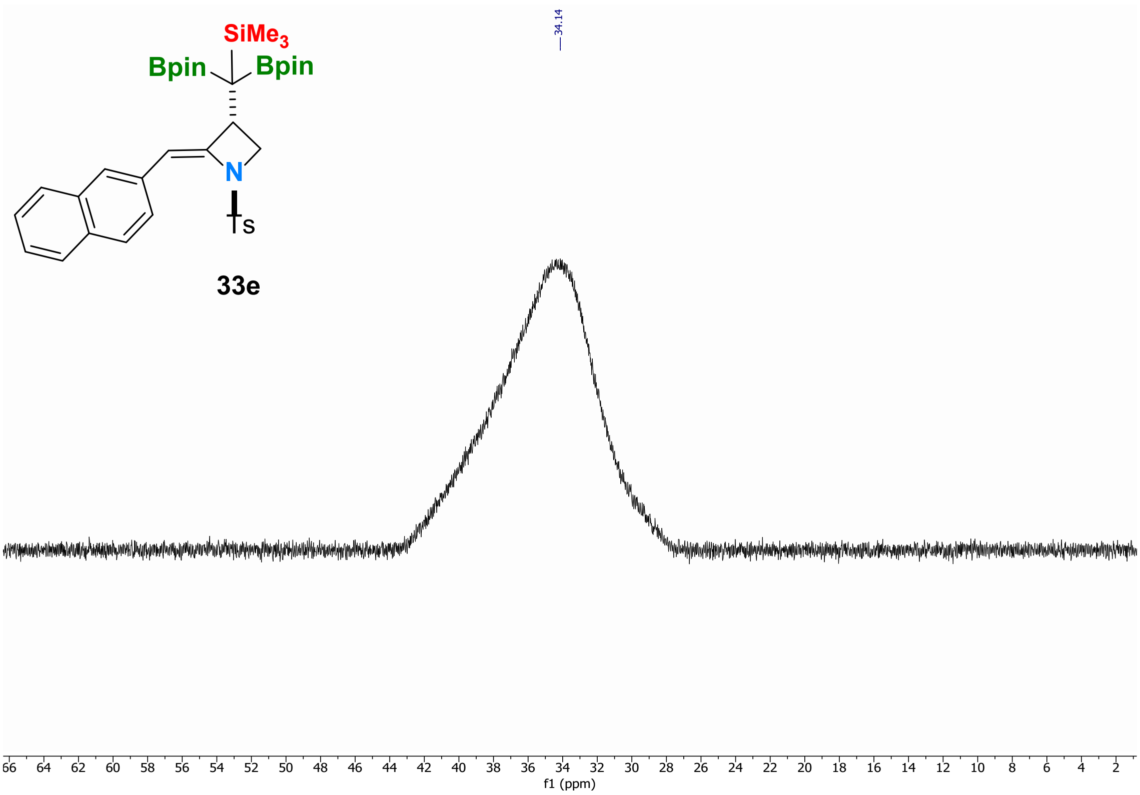

(*Z*)-3-(bis(4,4,5,5-tetramethyl-1,3,2-dioxaborolan-2-yl)(trimethylsilyl)methyl)-2-(cyclohexylmethylene)-1-tosylazetidine (34e)

^{1}H NMR (400 MHz, $CDCl_3$)

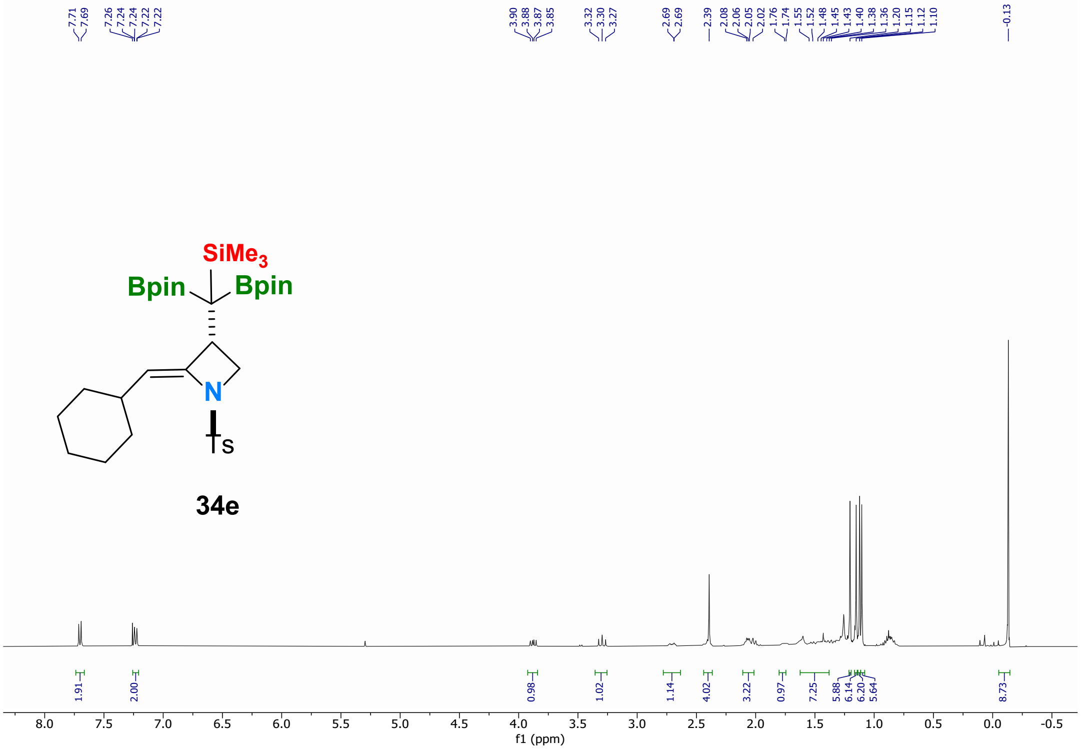

^{13}C NMR {^{1}H} (125 MHz, $CDCl_3$)

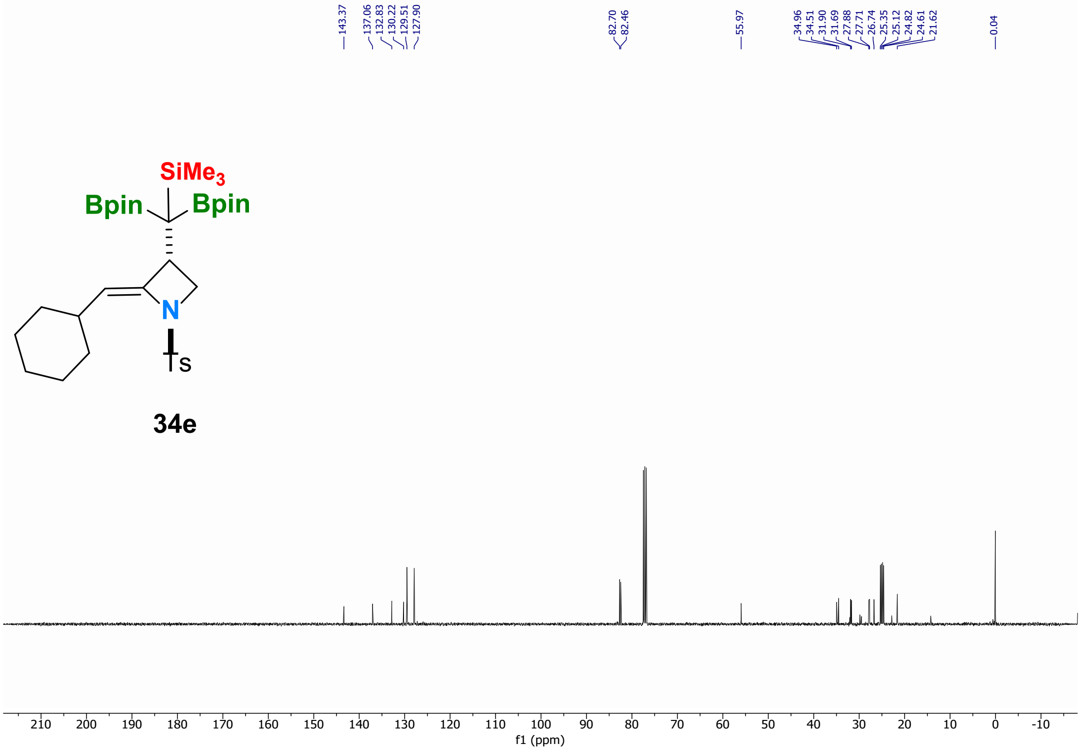

^{11}B NMR (128.3 MHz, CDCl$_3$)

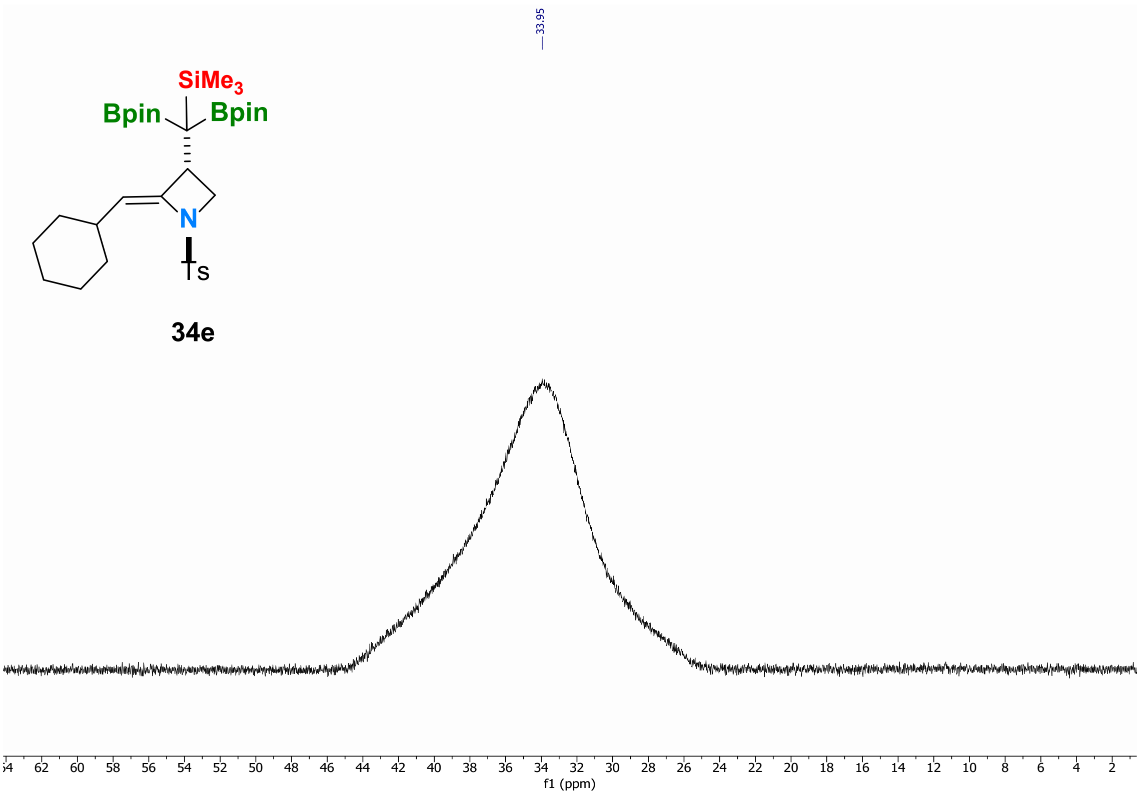

(*Z*)-3-(bis(4,4,5,5-tetramethyl-1,3,2-dioxaborolan-2-yl)(trimethylsilyl)methyl)-2-heptylidene-1-tosylazetidine (35e)

^{1}H NMR (400 MHz, CDCl$_3$)

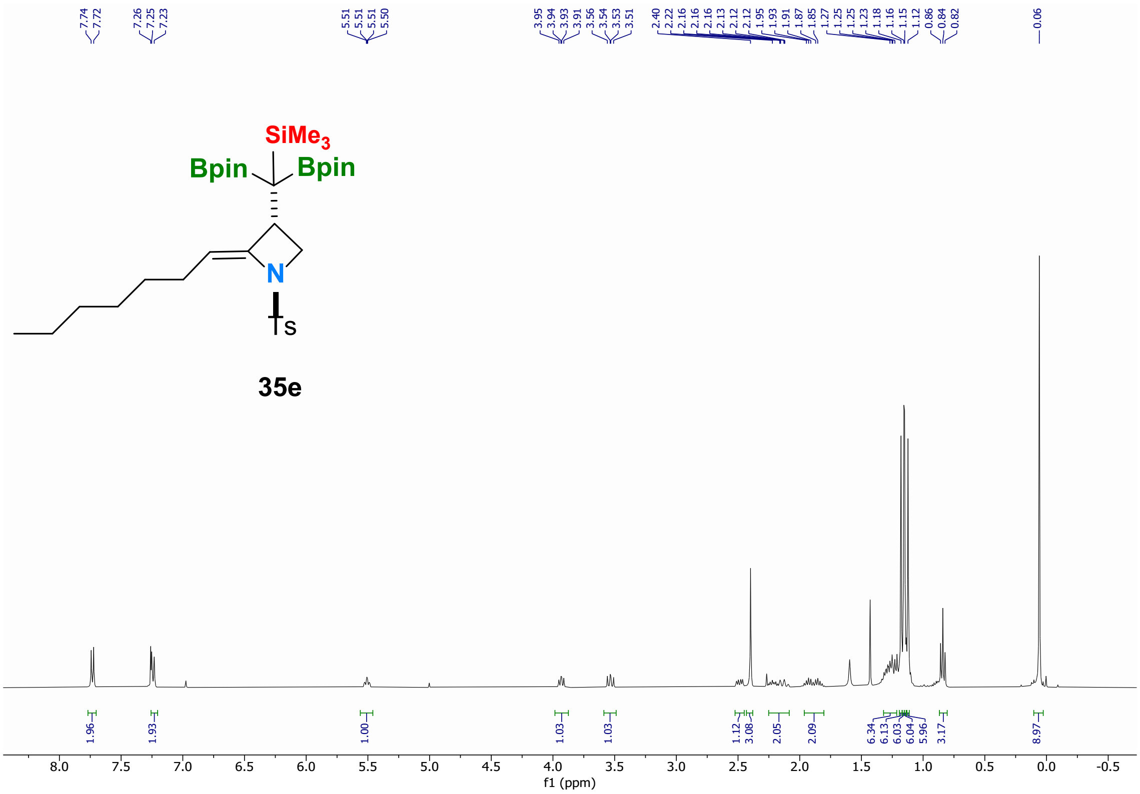

^{13}C NMR {^{1}H} (125 MHz, CDCl$_3$)

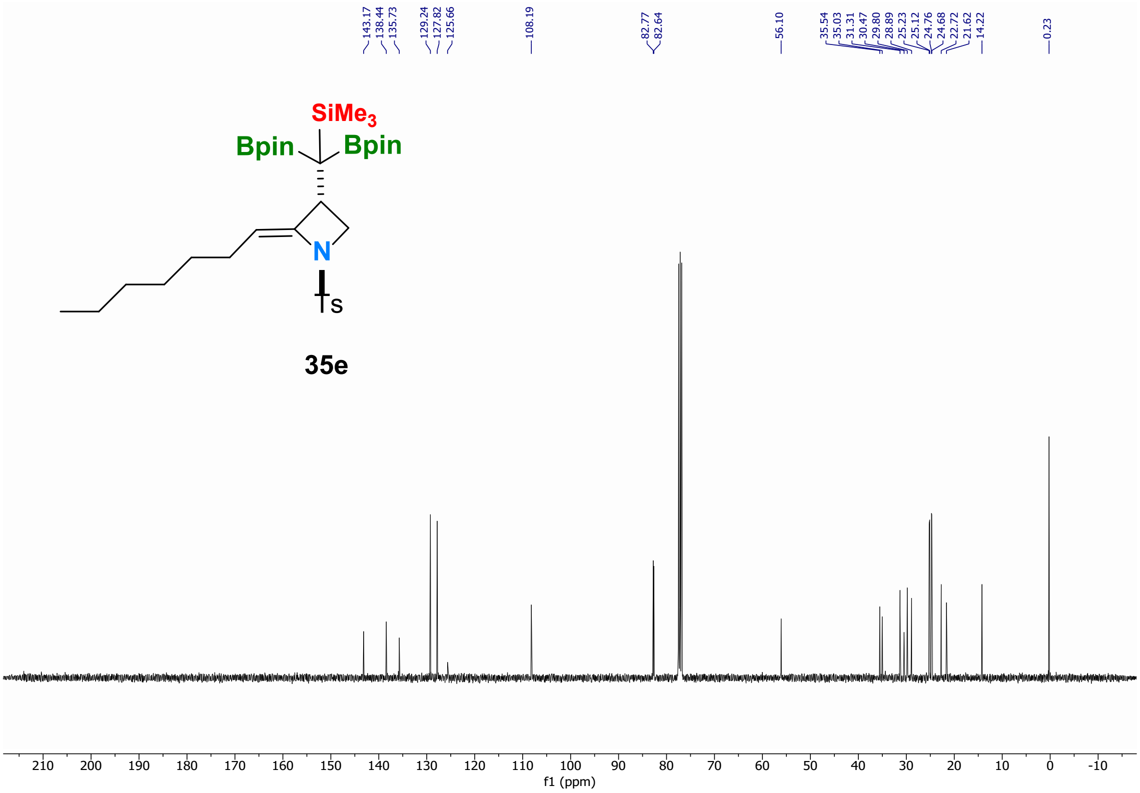

^{11}B NMR (128.3 MHz, CDCl$_3$)

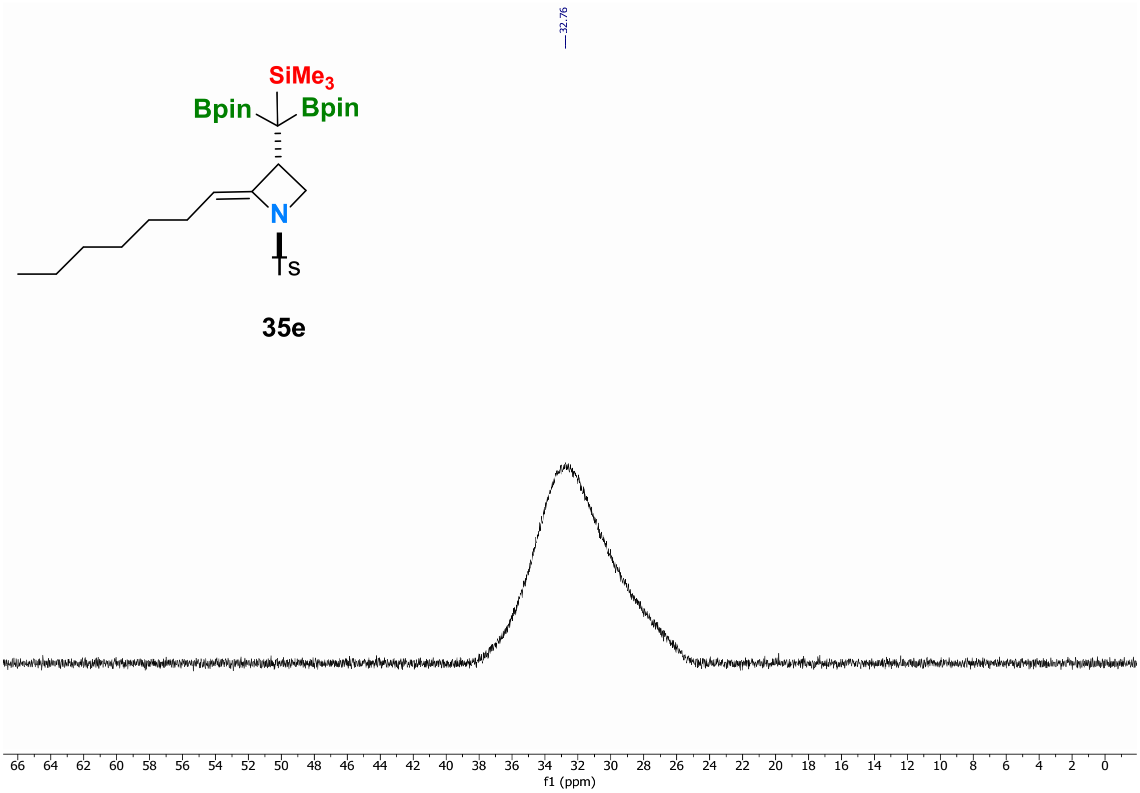

(*Z*)-3-(bis(4,4,5,5-tetramethyl-1,3,2-dioxaborolan-2-yl)methyl)-1-tosyl-2-(4-(trifluoromethyl)benzylidene)azetidine (38a)

^{1}H NMR (400 MHz, toluene d8)

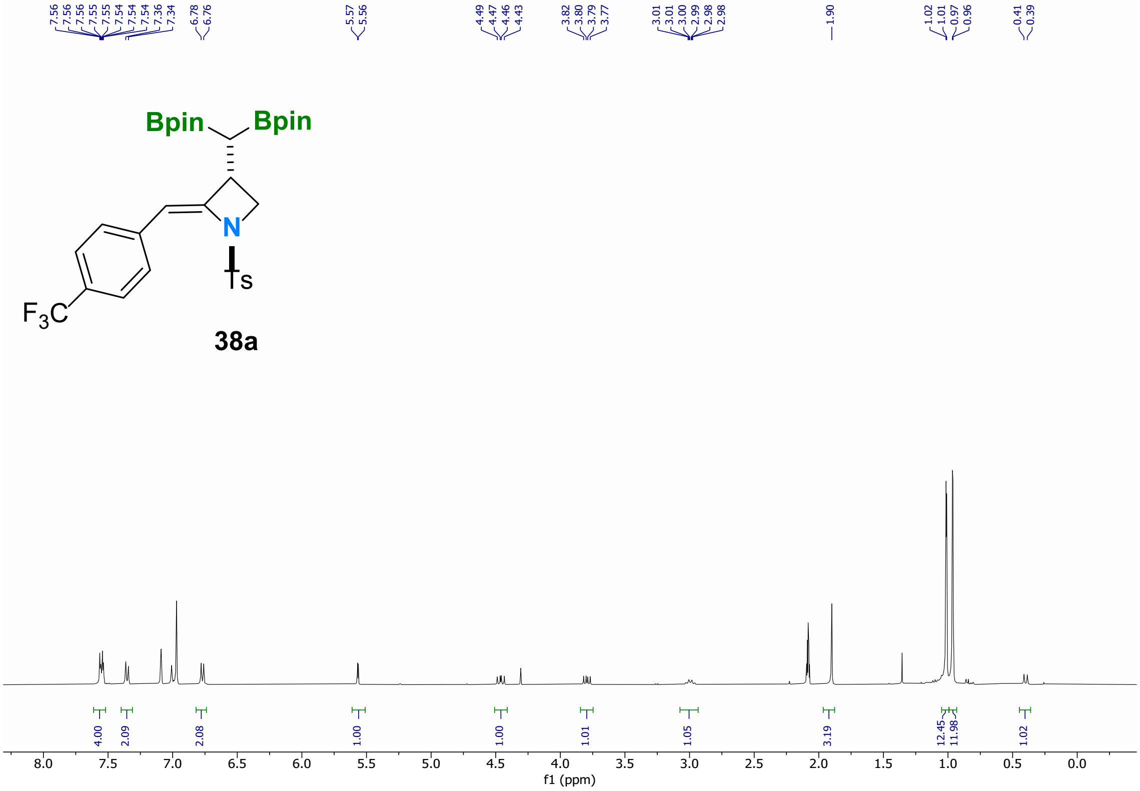

^{13}C NMR {^{1}H} (125 MHz, toluene d8)

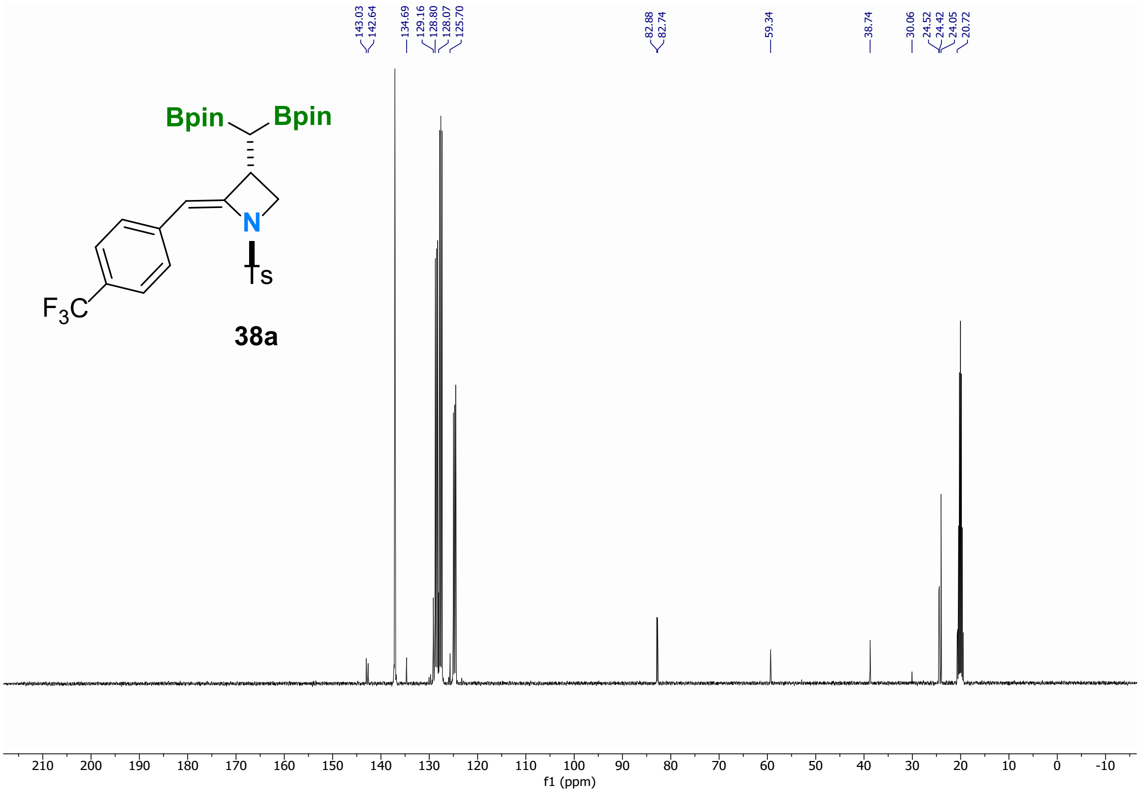

^{11}B NMR (128.3 MHz, toluene d8)

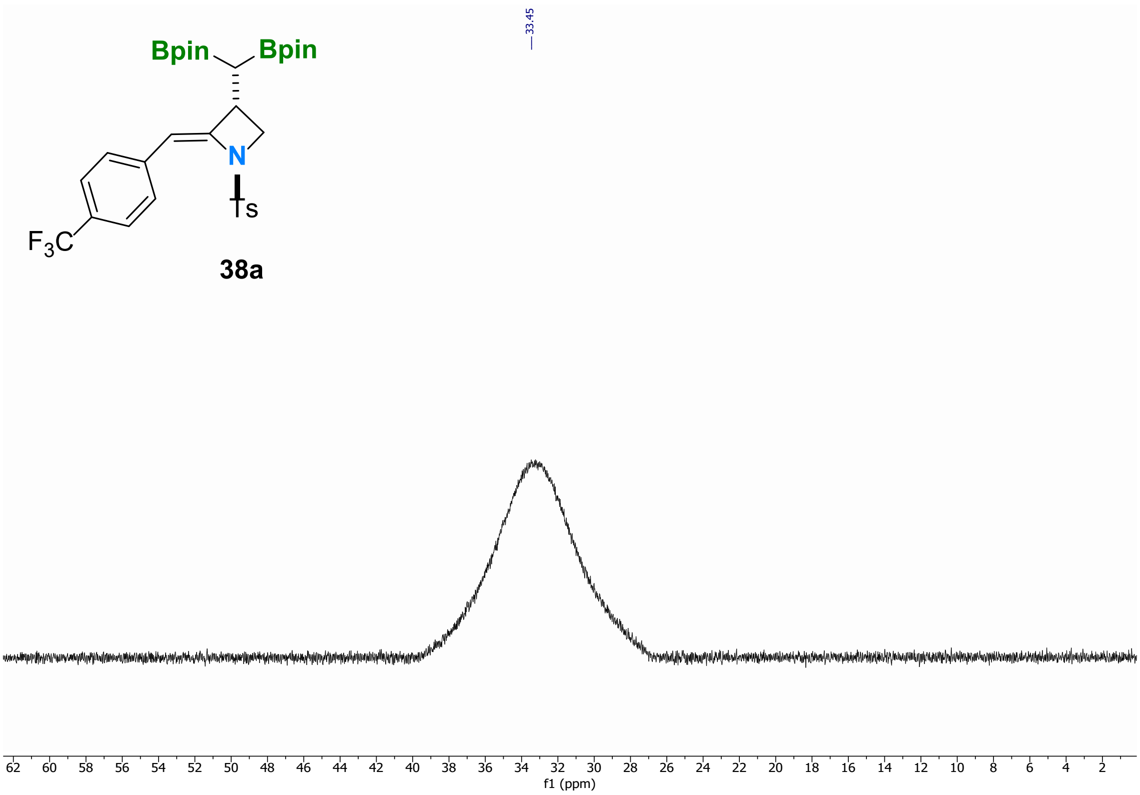

^{19}F NMR {^{1}H} (376.5 MHz, toluene d^8)

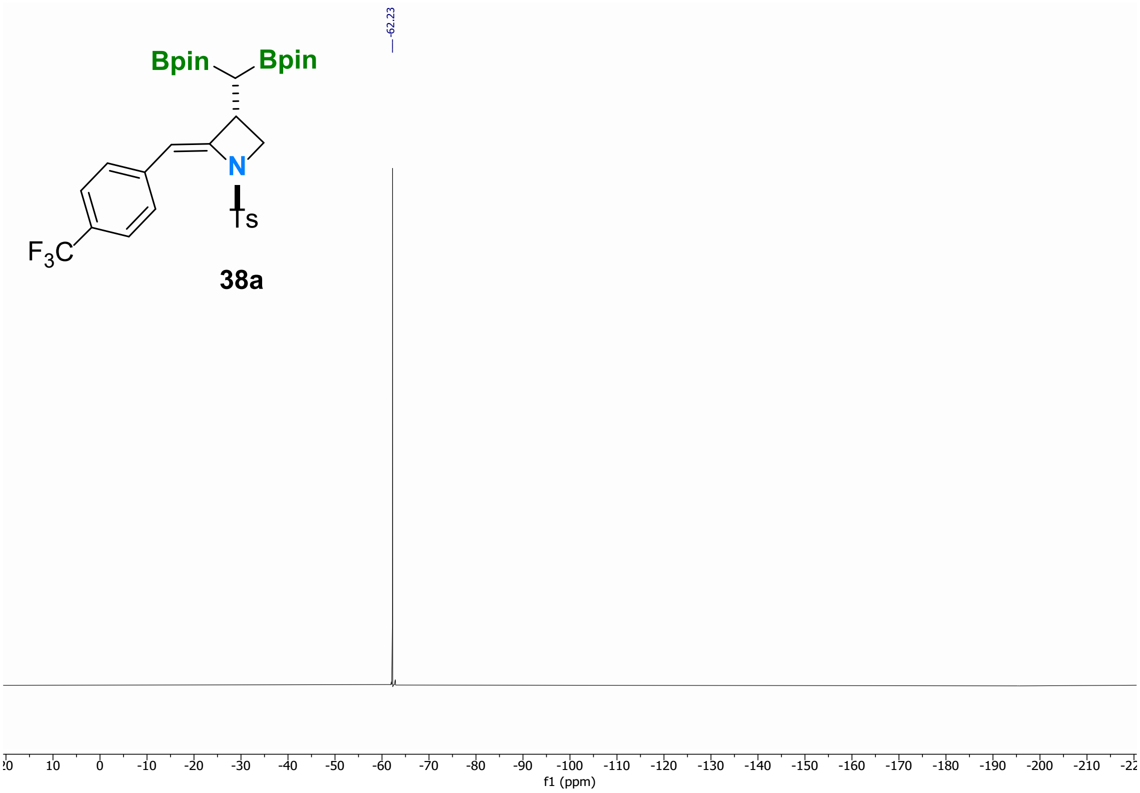

(*Z*)-3-(1,1-bis(4,4,5,5-tetramethyl-1,3,2-dioxaborolan-2-yl)ethyl)-1-tosyl-2-(4-(trifluoromethyl)benzylidene)azetidine (38b)

^{1}H NMR (400 MHz, toluene d8)

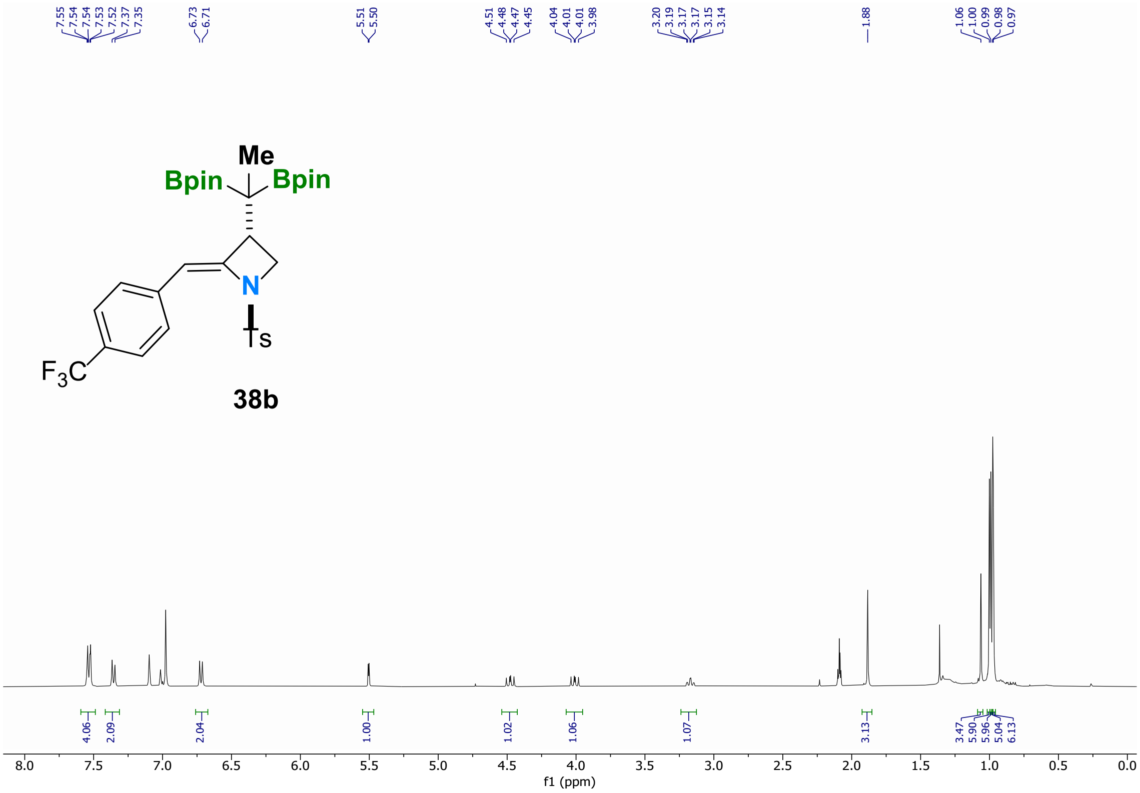

1D-NMR NOE experiment for 38b

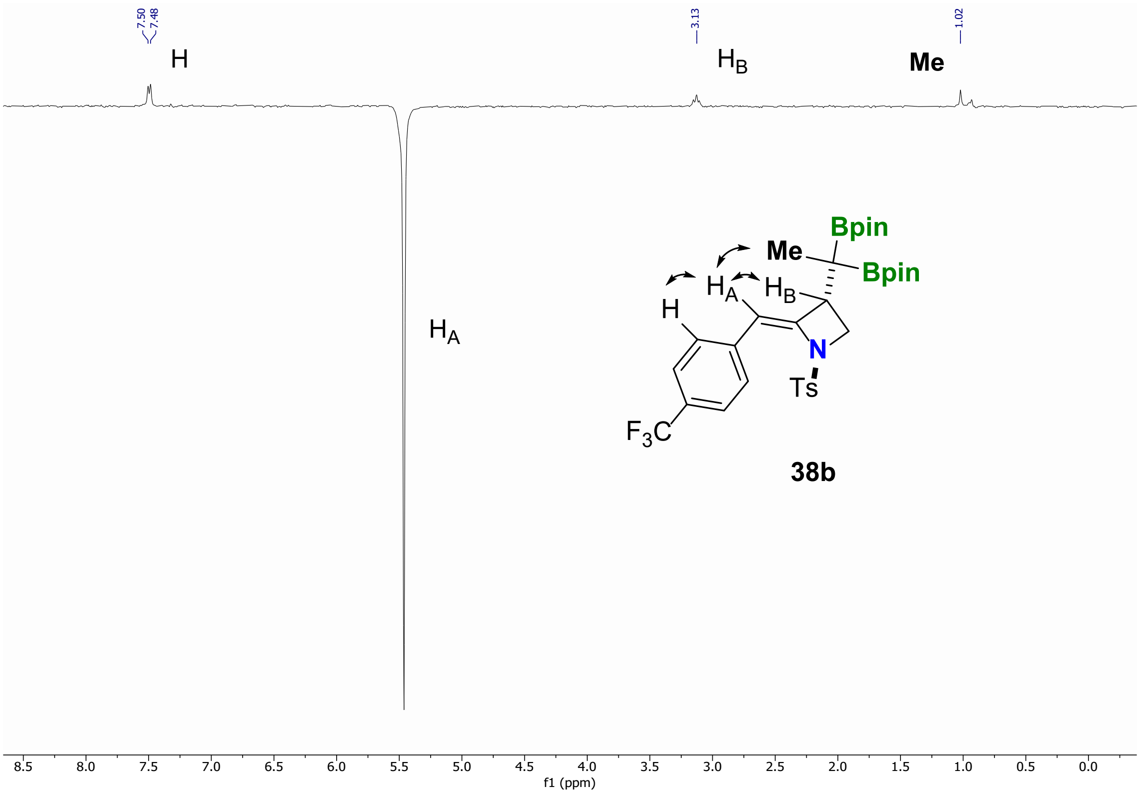

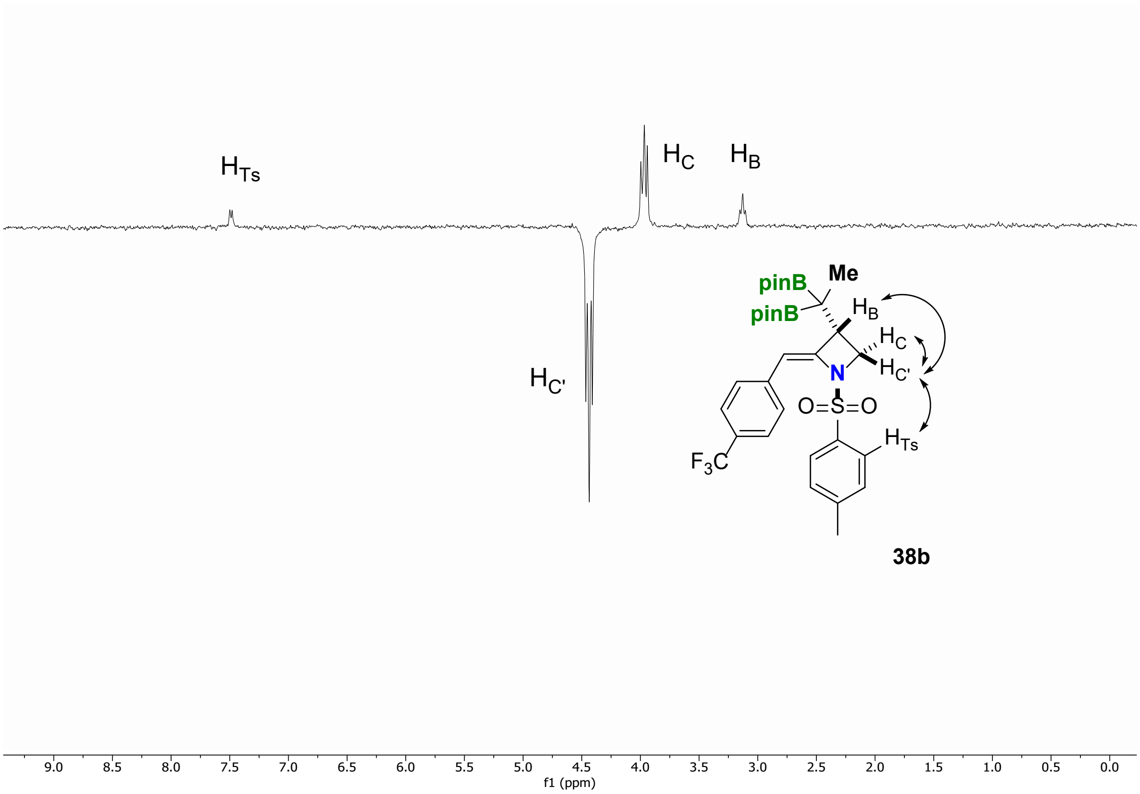

^{13}C NMR {^{1}H} (125 MHz, toluene d8)

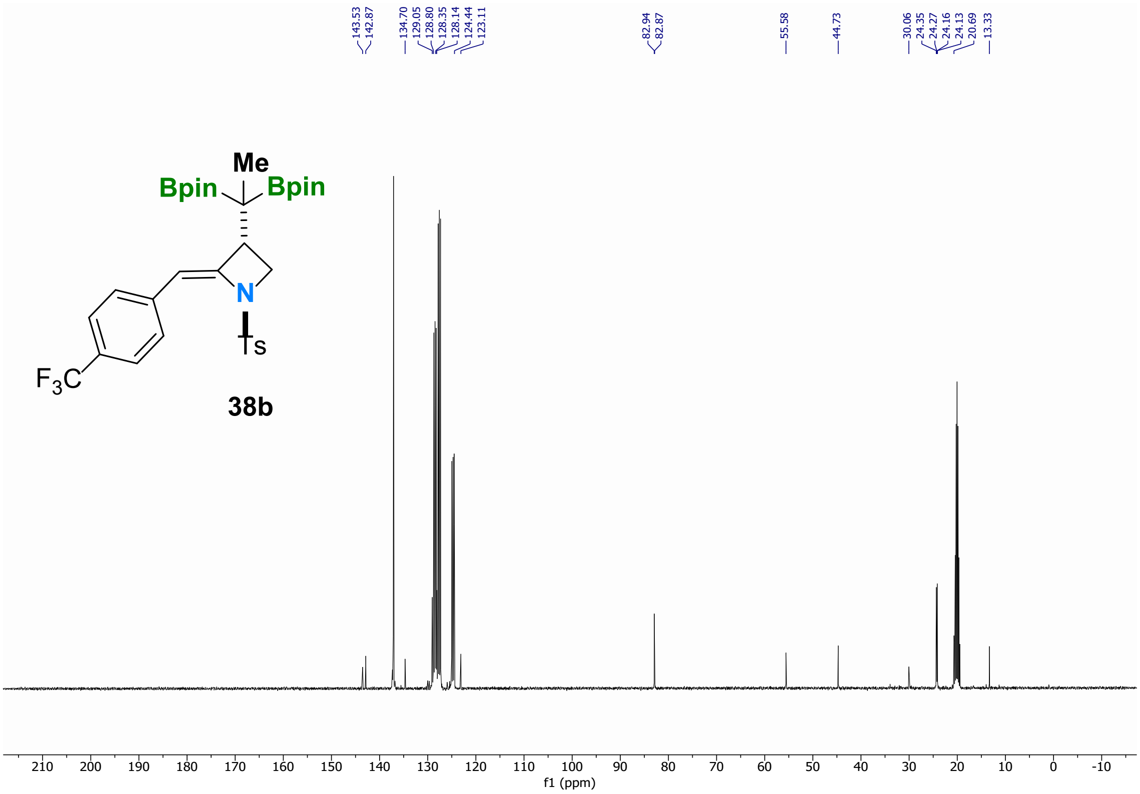

^{11}B NMR (128.3 MHz, toluene d8)

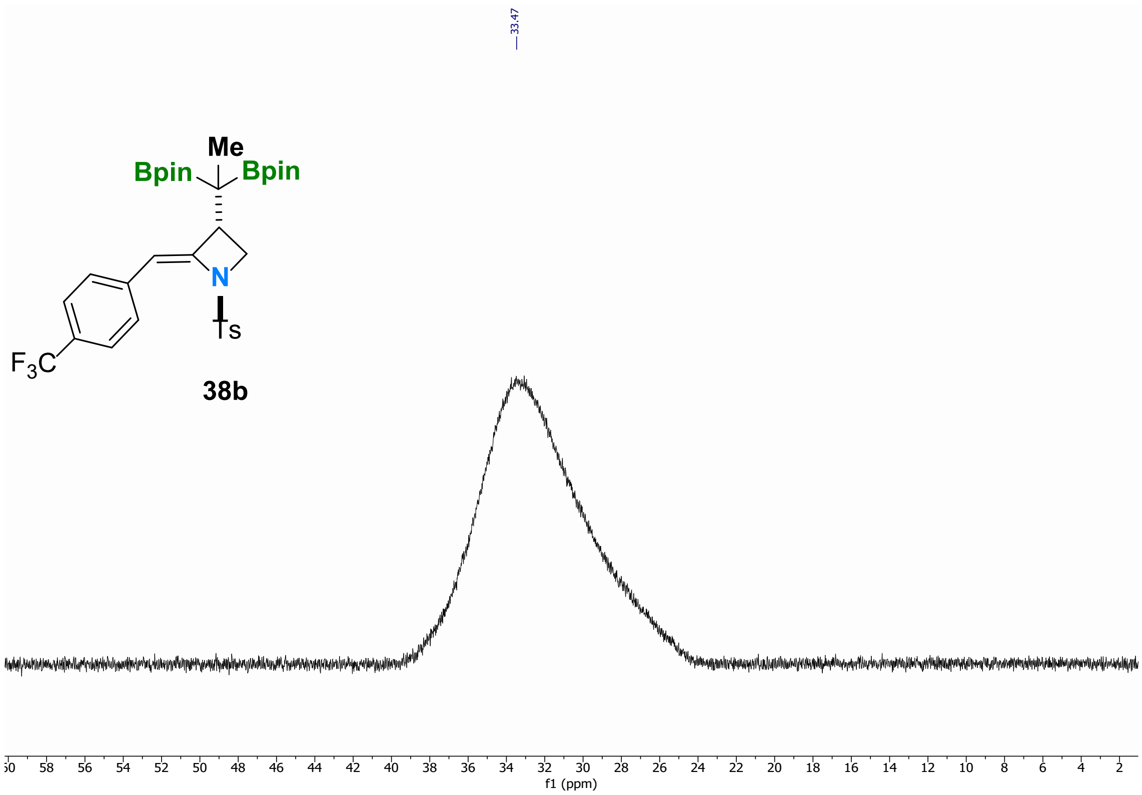

^{19}F NMR {^{1}H} (376.5 MHz, toluene d^{8})

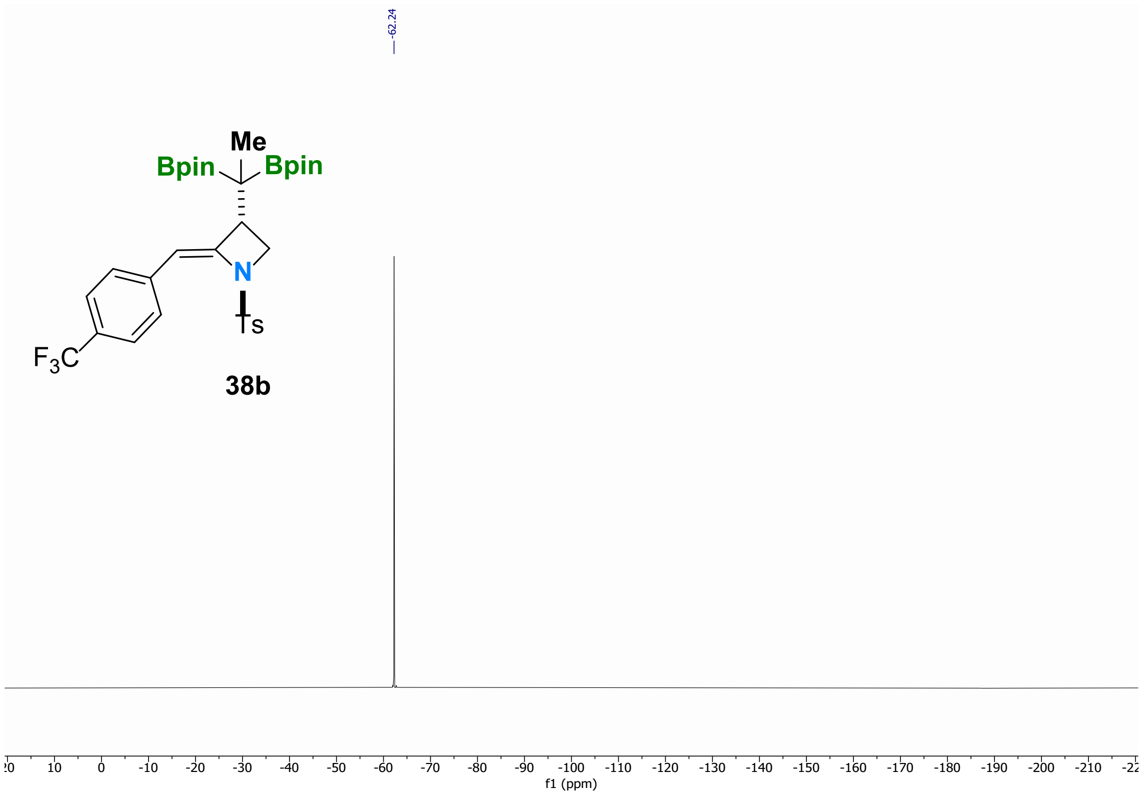

S17. NMR spectra of 39b and 40b

2-Methyl-2-(naphthalen-2-ylethynyl)-1-tosylaziridine

^{1}H NMR (500 MHz, $CDCl_3$)

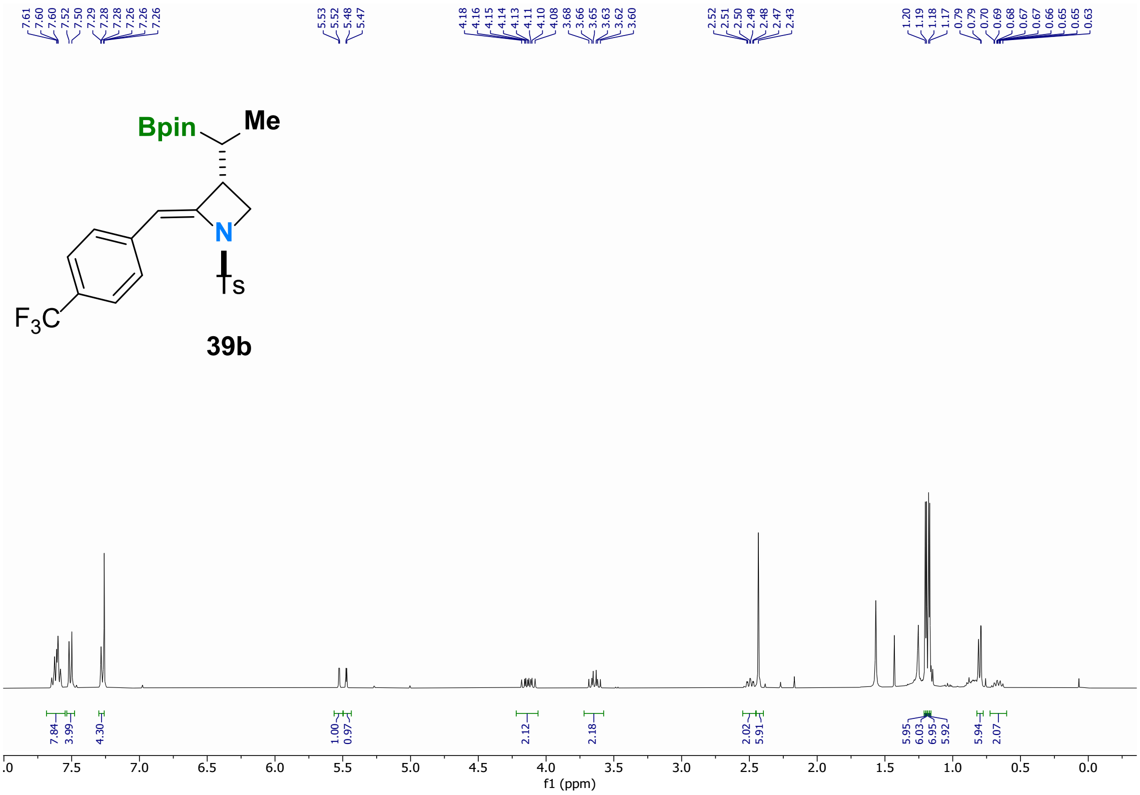

^{13}C NMR {^{1}H} (125 MHz, $CDCl_3$)

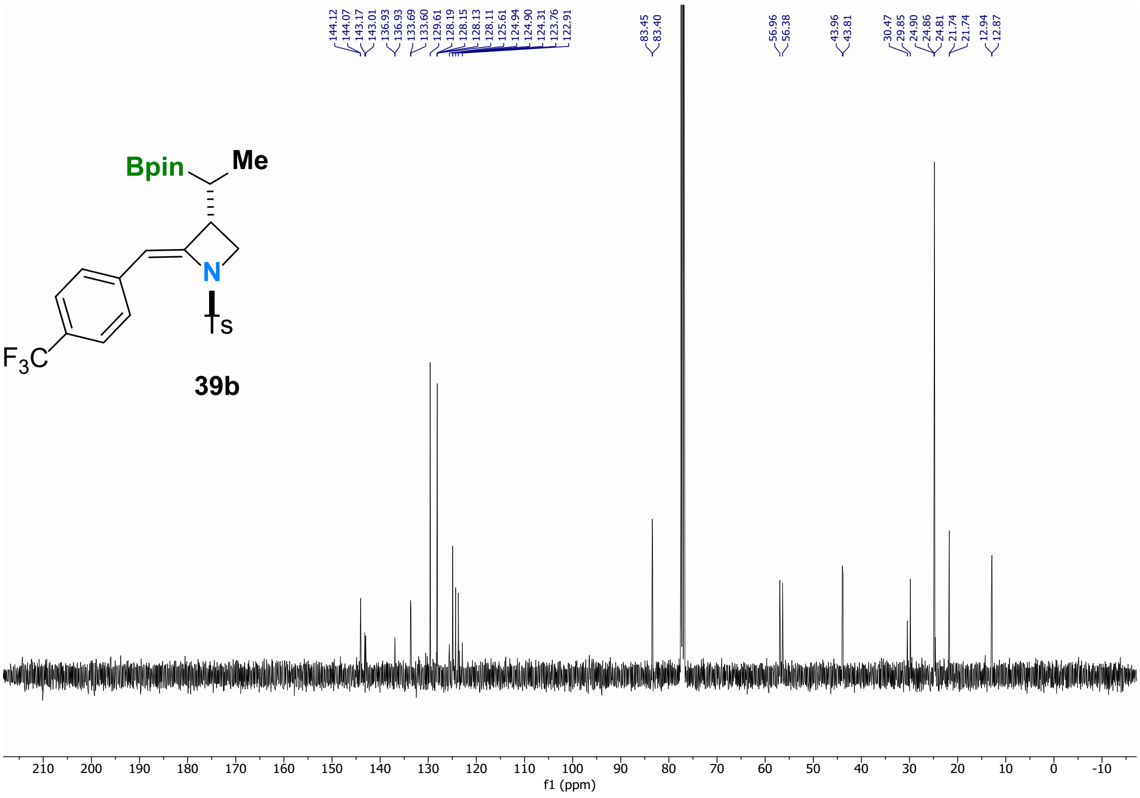

^{11}B NMR (128.3 MHz, $CDCl_3$)

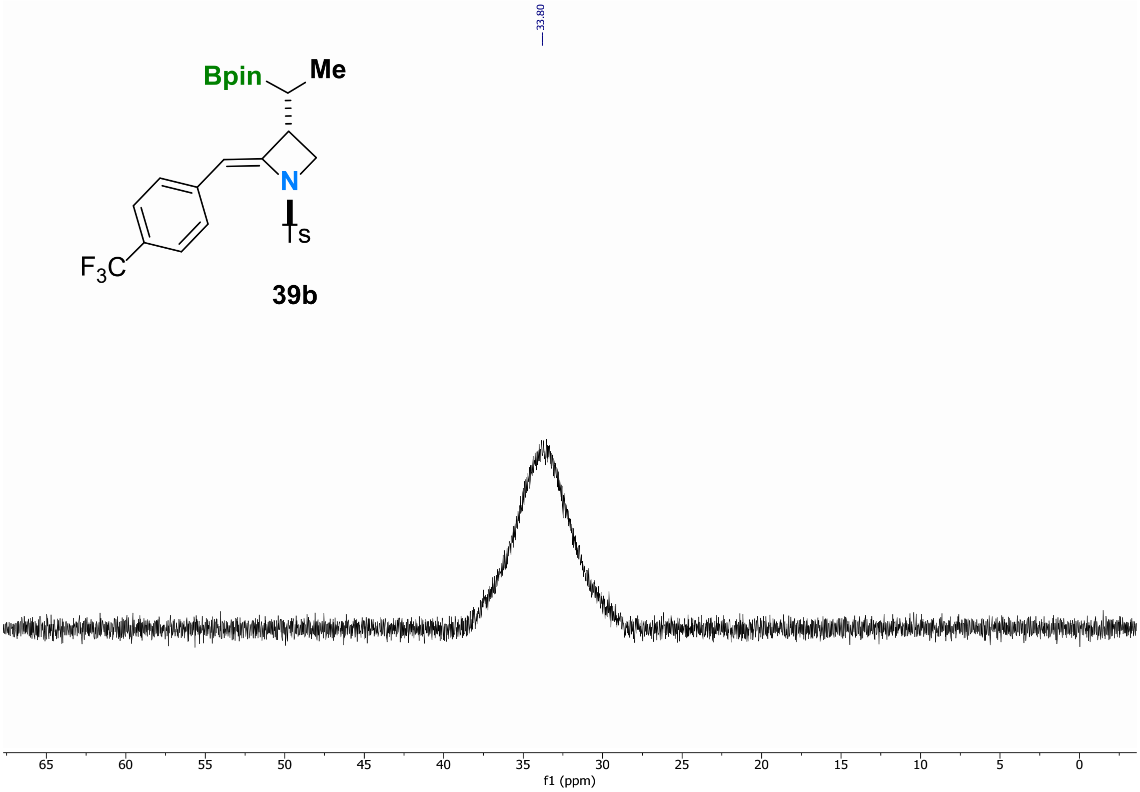

^{19}F NMR {^{1}H} (376.5 MHz, $CDCl_3$)

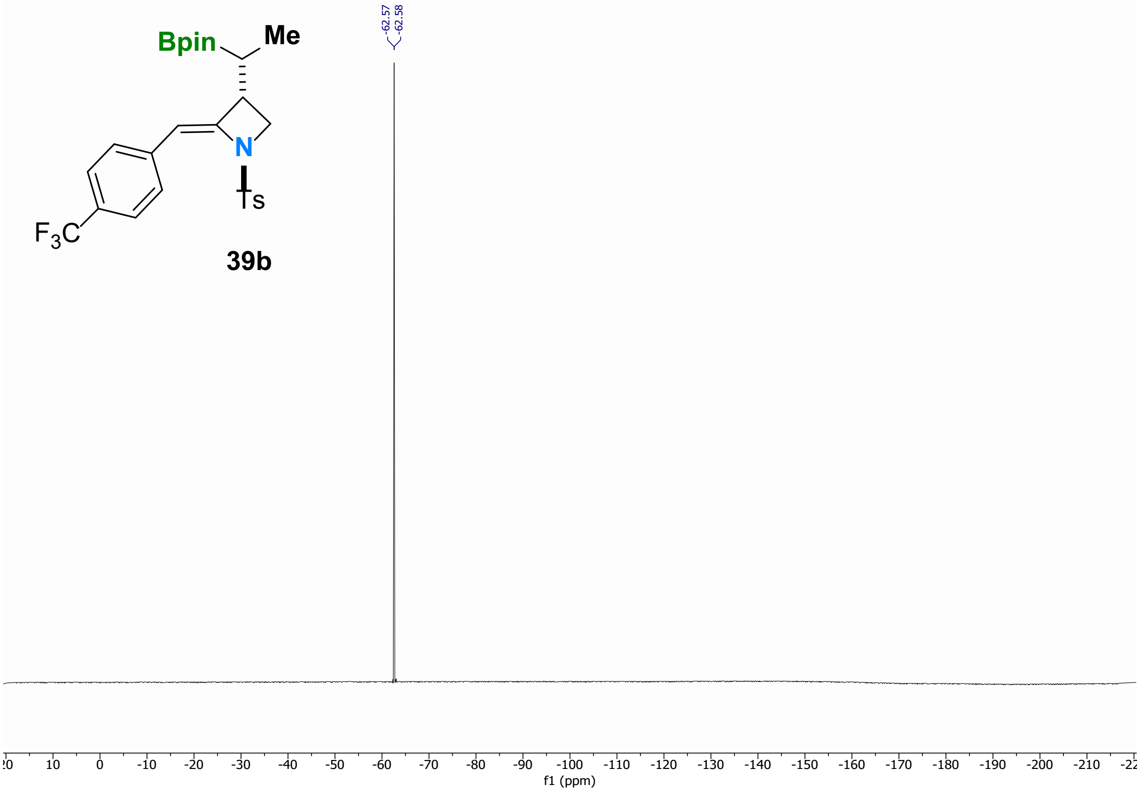

(*Z*)-1-(1-tosyl-2-(4-(trifluoromethyl)benzylidene)azetidin-3-yl)ethan-1-ol (40b)

^{1}H NMR (500 MHz, $CDCl_3$)

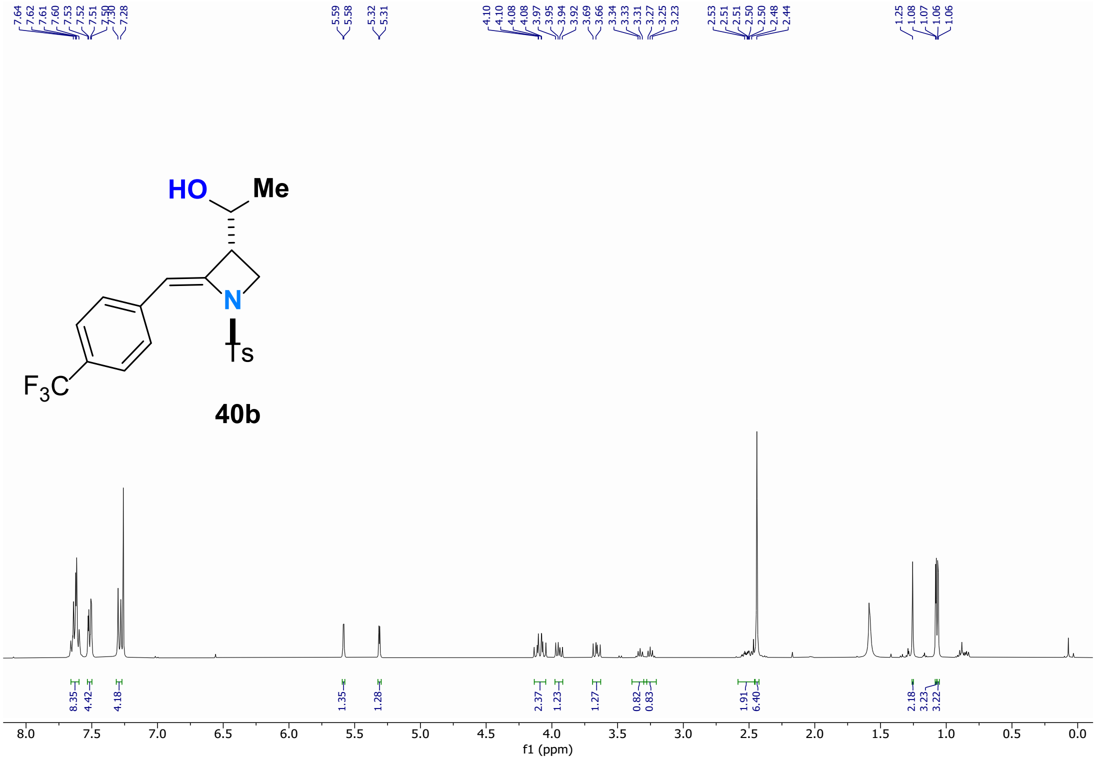

^{13}C NMR {^{1}H} (125 MHz, $CDCl_3$)

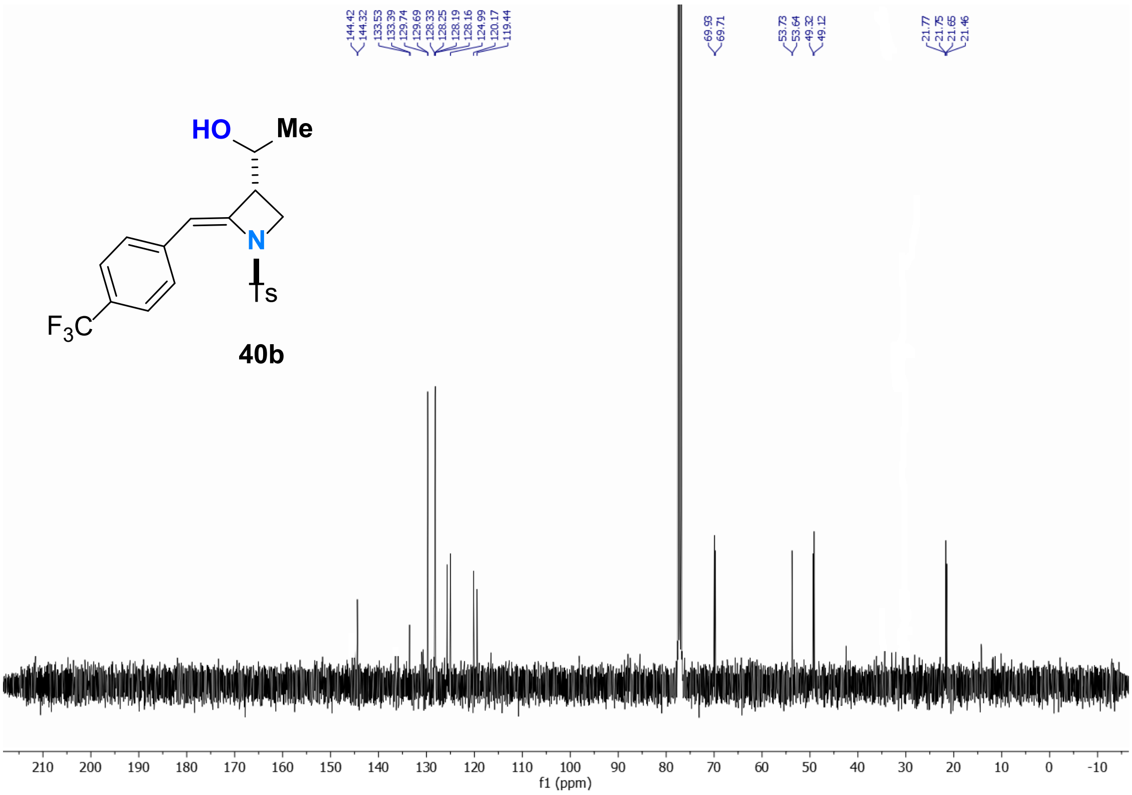

^{19}F NMR {^{1}H} (376.5 MHz, CDCl$_3$)

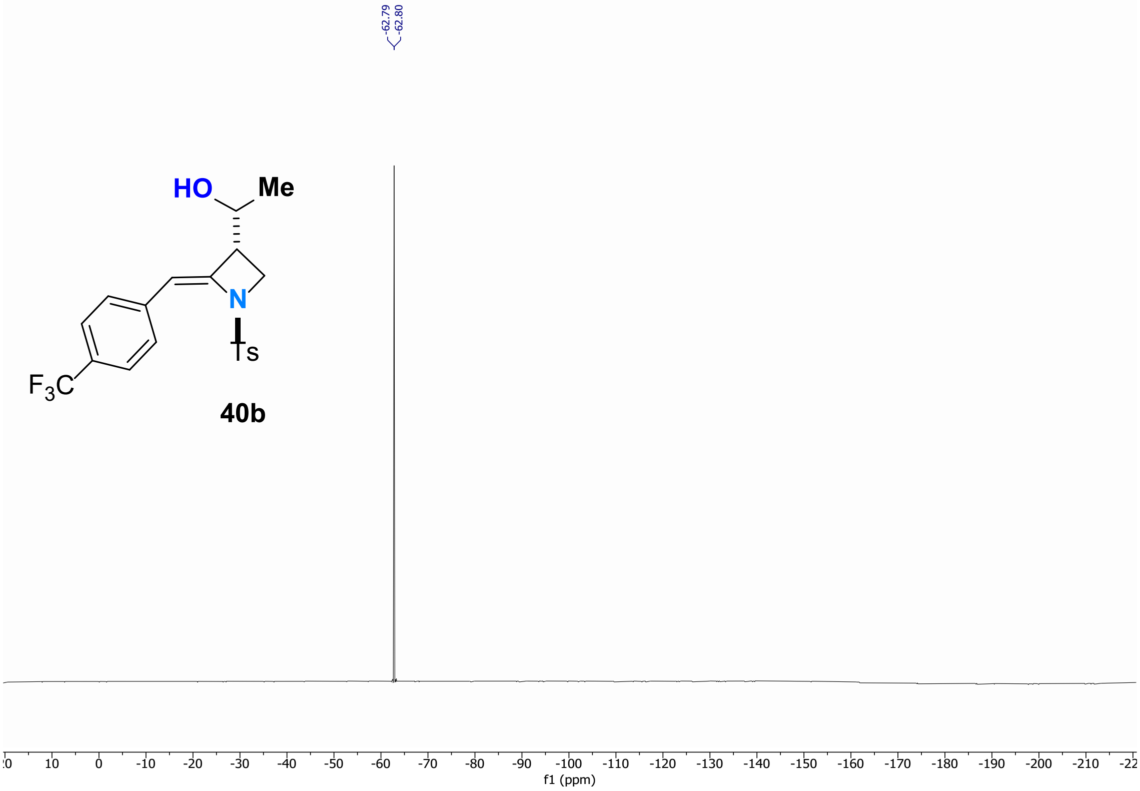

S18. X-ray single-crystal diffraction analysis for product 29e

Deposition Number CCDC 2363558

Method for crystal growth: A saturated solution of 10-15 mg of solid (compound **29e**) in 0.1 mL of dichloromethane was prepared in a 2mL GC-vial. Then, 1 mL of *n*-hexane was then carefully added with a syringe equipped with a needle forming two layers. The system was then allowed to slowly evaporate at room temperature with a needle in the cap until the crystals were obtained.

A suitable crystal was selected and measured on a Bruker APEX-II CCD diffractometer. The crystal was kept at 97.86 K during data collection. Using Olex2, the structure was solved with the SHELXT structure solution program using Intrinsic Phasing and refined with the XL refinement package using Least Squares minimisation.

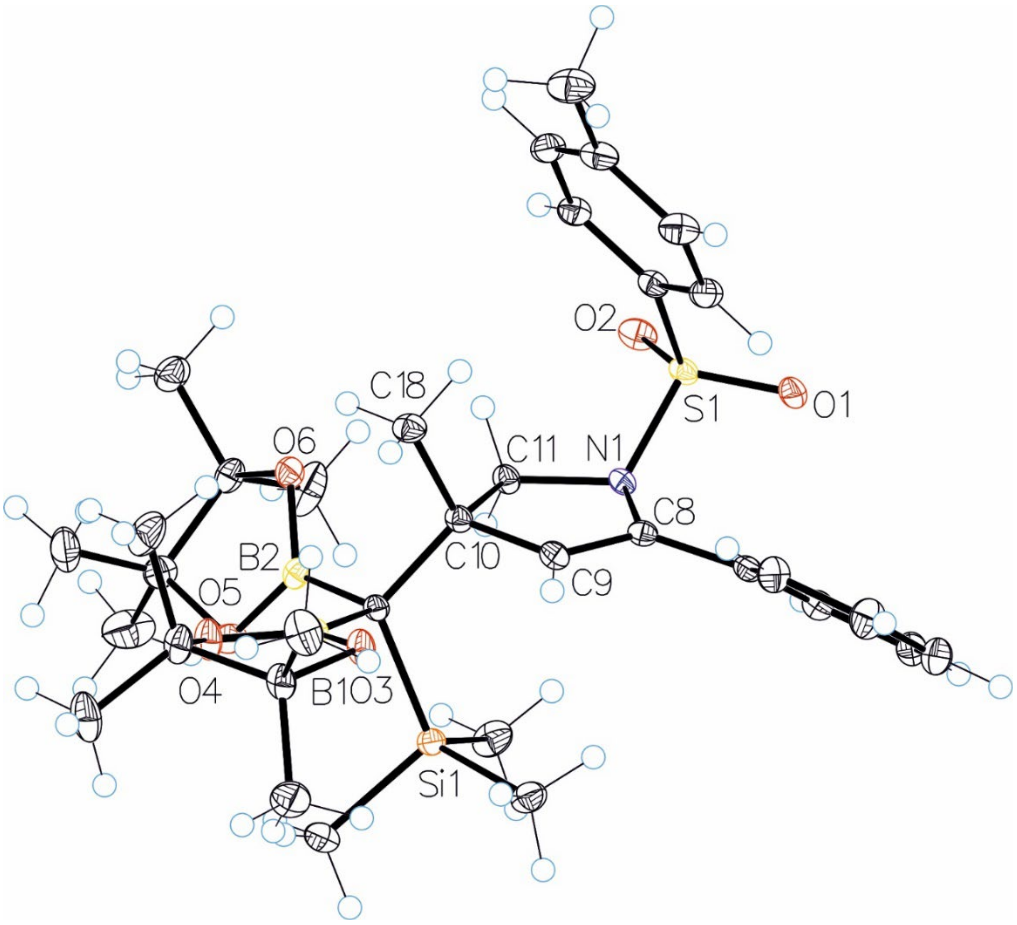

Crystal Data for $C_{34}H_{51}B_2NO_6SSi$ (M =651.52 g/mol): monoclinic, space group P2$_1$/n (no. 14), a = 11.2876(15) Å, b = 21.391(3) Å, c = 15.272(2) Å, β = 104.150(4)°, V = 3575.5(8) Å^3, Z = 4, T = 97.86 K, μ(MoKα) = 0.167 mm^{-1}, $Dcalc$ = 1.210 g/cm^3, 46325 reflections measured (3.346° ≤ 2Θ ≤ 63.116°), 11919 unique (R_{int} = 0.0765, R_{sigma} = 0.0695) which were used in all calculations. The final R_1 was 0.0483 (I > 2σ(I)) and wR_2 was 0.1341 (all data). Ellipsoids shown at 50 % probability level

Table S1 Crystal data and structure refinement for 29e

Identification code	mo_OS793f10_0m
Empirical formula	$C_{34}H_{51}B_2NO_6SSi$
Formula weight	651.52
Temperature/K	97.86
Crystal system	monoclinic
Space group	$P2_1/n$
a/Å	11.2876(15)
b/Å	21.391(3)
c/Å	15.272(2)
α/°	90
β/°	104.150(4)
γ/°	90
Volume/Å^3	3575.5(8)
Z	4
ρ_{calc}g/cm^3	1.210
μ/mm^{-1}	0.167
F(000)	1400.0
Crystal size/mm^3	0.2 × 0.15 × 0.1
Radiation	MoKα (λ = 0.71073)
2Θ range for data collection/°	3.346 to 63.116
Index ranges	$-12 \leq h \leq 16$, $-31 \leq k \leq 31$, $-17 \leq l \leq 22$
Reflections collected	46325
Independent reflections	11919 [R_{int} = 0.0765, R_{sigma} = 0.0695]
Data/restraints/parameters	11919/0/419
Goodness-of-fit on F^2	1.029
Final R indexes [I>=2σ (I)]	R_1 = 0.0483, wR_2 = 0.1230
Final R indexes [all data]	R_1 = 0.0660, wR_2 = 0.1341
Largest diff. peak/hole / e Å^{-3}	0.47/-0.46

Table S2 Fractional Atomic Coordinates (×10⁴) and Equivalent Isotropic Displacement Parameters (Å²×10³) for 29e. U_{eq} is defined as 1/3 of the trace of the orthogonalised U_{IJ} tensor.

Atom	*x*	*y*	*z*	U(eq)
S1	8253.3(3)	5782.4(2)	3581.9(2)	13.61(8)
Si1	4124.4(3)	7489.6(2)	3067.8(2)	13.48(8)
O1	8027.4(9)	5272.3(5)	2957.9(7)	19.1(2)
O2	9196.0(8)	6226.2(5)	3559.8(7)	18.7(2)
O3	3607.9(9)	6959.5(5)	5038.7(6)	16.68(19)
O4	4216.5(9)	7964.3(5)	5396.4(7)	18.8(2)
O5	5733.5(9)	8594.3(5)	4143.0(7)	19.2(2)
O6	7305.1(8)	7956.5(4)	4835.9(6)	16.24(19)
N1	6965.2(9)	6194.5(5)	3407.3(7)	12.4(2)
C1	8502.6(11)	5475.9(6)	4682.4(9)	14.0(2)
C2	9294.6(12)	5771.8(6)	5402.4(9)	16.7(3)
C3	9412.6(13)	5539.9(7)	6268.1(9)	19.2(3)
C4	8761.2(12)	5017.0(7)	6425.8(9)	19.4(3)
C5	7977.4(13)	4724.4(7)	5688.5(10)	20.4(3)
C6	7841.8(12)	4951.6(6)	4820.0(9)	17.7(3)
C7	8905.6(15)	4764.7(8)	7366.1(10)	27.3(3)
C8	5852.1(11)	5892.3(6)	3489.2(8)	12.8(2)
C9	5312.0(11)	6215.2(6)	4029.1(9)	13.6(2)
C10	6009.2(11)	6788.7(6)	4425.5(8)	12.6(2)
C11	7051.2(11)	6796.8(6)	3920.0(9)	13.4(2)
C12	5327.8(11)	5349.0(6)	2929.6(9)	14.0(2)
C13	5427.3(12)	5290.7(6)	2042.3(9)	16.7(3)
C14	4813.0(13)	4815.9(7)	1498.1(10)	19.7(3)
C15	4100.5(14)	4392.0(7)	1829.8(10)	23.0(3)
C16	4014.3(14)	4444.1(7)	2716.1(10)	23.6(3)
C17	4628.1(13)	4917.4(7)	3266.5(10)	19.2(3)
C18	6531.5(12)	6686.5(7)	5448.6(9)	16.9(3)
C19	5218.3(11)	7406.3(6)	4250.9(8)	11.9(2)
C20	4974.0(14)	7580.6(8)	2167.1(10)	22.9(3)
C21	3011.8(13)	6830.7(7)	2733.0(10)	20.3(3)
C22	3168.8(13)	8204.3(7)	3060.7(10)	22.2(3)
C23	2803.6(12)	7192.6(7)	5583.5(9)	17.3(3)
C24	2656.5(15)	6681.8(8)	6237.7(11)	25.7(3)
C25	4405.8(15)	7660.4(9)	6931.5(10)	29.2(3)
C26	1575.1(13)	7329.9(8)	4936.0(11)	25.3(3)
C27	3496.4(13)	7791.3(7)	6033.2(9)	19.0(3)
C28	2690.4(16)	8343.3(8)	6127.5(12)	28.8(3)
C29	6766.5(13)	9001.8(6)	4548.5(10)	19.0(3)
C30	7853.5(12)	8544.4(6)	4655.9(9)	16.6(3)
C31	6564.8(15)	9225.3(8)	5448.3(12)	27.9(3)
C32	6794.0(17)	9551.6(8)	3926.7(13)	37.1(4)

Atom	*x*	*y*	*z*	U(eq)
C33	8943.7(13)	8687.3(7)	5427.7(10)	22.8(3)
C34	8265.2(16)	8444.6(9)	3794.1(11)	32.5(4)
B1	4338.8(13)	7445.4(7)	4903.9(10)	13.5(3)
B2	6077.5(13)	7996.3(7)	4418.2(9)	13.6(3)

Table S3 Anisotropic Displacement Parameters (Å^2×10^3) for 29e. The Anisotropic displacement factor exponent takes the form: $-2\pi^2[h^2a^{*2}U_{11}+2hka^*b^*U_{12}+\ldots]$.

Atom	U_{11}	U_{22}	U_{33}	U_{23}	U_{13}	U_{12}
S1	11.84(14)	16.09(16)	13.99(14)	0.92(11)	5.24(11)	3.33(11)
Si1	12.34(16)	16.45(18)	11.82(16)	-0.40(12)	3.28(12)	1.27(12)
O1	19.0(5)	21.6(5)	17.0(5)	-3.9(4)	4.9(4)	7.5(4)
O2	13.1(4)	23.4(5)	21.9(5)	4.2(4)	8.4(4)	1.4(4)
O3	16.6(4)	18.3(5)	18.8(5)	-3.9(4)	11.4(4)	-1.6(4)
O4	21.0(5)	18.8(5)	21.2(5)	-5.6(4)	14.1(4)	-3.1(4)
O5	16.8(5)	16.0(5)	22.0(5)	3.6(4)	-0.5(4)	-2.5(4)
O6	14.4(4)	13.4(4)	20.5(5)	-2.1(4)	3.5(4)	-1.9(3)
N1	11.1(5)	12.8(5)	14.1(5)	0.2(4)	4.6(4)	2.2(4)
C1	12.8(5)	15.3(6)	14.7(6)	1.8(5)	4.8(4)	2.9(4)
C2	13.5(6)	17.4(6)	18.9(6)	1.9(5)	3.4(5)	0.9(5)
C3	16.4(6)	22.8(7)	16.8(6)	0.1(5)	1.0(5)	1.6(5)
C4	15.4(6)	25.8(7)	17.5(6)	5.1(5)	4.7(5)	5.0(5)
C5	16.8(6)	21.7(7)	22.4(7)	7.1(5)	4.5(5)	-0.6(5)
C6	15.8(6)	17.0(6)	19.0(6)	1.4(5)	1.9(5)	0.1(5)
C7	24.8(7)	38.7(9)	19.0(7)	9.5(6)	6.0(6)	4.2(6)
C8	12.1(5)	13.7(6)	12.7(5)	2.5(4)	3.0(4)	1.1(4)
C9	12.5(5)	13.5(6)	15.9(6)	1.8(4)	5.2(4)	0.8(4)
C10	11.6(5)	14.4(6)	12.7(5)	0.1(4)	4.6(4)	1.2(4)
C11	11.8(5)	13.5(6)	16.3(6)	-1.0(4)	5.9(4)	0.3(4)
C12	12.2(5)	12.8(6)	16.6(6)	0.7(4)	2.6(4)	2.5(4)
C13	16.7(6)	15.7(6)	17.6(6)	1.3(5)	3.9(5)	1.7(5)
C14	23.3(7)	17.3(6)	18.0(6)	-1.5(5)	3.7(5)	2.6(5)
C15	24.3(7)	17.1(7)	25.8(7)	-4.3(5)	2.6(6)	-2.0(5)
C16	25.4(7)	18.2(7)	27.5(7)	0.2(6)	7.3(6)	-6.1(5)
C17	21.0(7)	17.2(6)	20.3(6)	0.0(5)	7.0(5)	-1.9(5)
C18	16.9(6)	20.5(7)	13.3(6)	2.8(5)	3.8(5)	2.4(5)
C19	11.1(5)	13.8(6)	11.4(5)	-0.7(4)	4.1(4)	1.6(4)
C20	22.1(7)	33.6(8)	14.2(6)	0.9(5)	6.4(5)	-2.0(6)
C21	16.1(6)	24.7(7)	18.6(6)	-1.5(5)	1.5(5)	-1.2(5)
C22	19.6(7)	22.9(7)	22.2(7)	0.1(5)	1.8(5)	7.1(5)
C23	16.7(6)	20.2(7)	18.7(6)	-3.2(5)	11.3(5)	-0.5(5)
C24	29.5(8)	26.3(8)	27.8(8)	1.3(6)	19.4(6)	-0.8(6)
C25	29.9(8)	41.7(10)	17.7(7)	-8.9(6)	8.9(6)	-5.1(7)
C26	15.9(6)	32.0(8)	29.1(8)	-3.0(6)	7.4(6)	-1.2(6)
C27	20.6(6)	22.5(7)	17.8(6)	-5.5(5)	12.0(5)	-3.1(5)
C28	32.7(8)	26.1(8)	34.6(8)	-9.2(7)	22.1(7)	0.4(6)
C29	20.4(6)	13.8(6)	21.0(6)	2.7(5)	1.7(5)	-3.5(5)
C30	17.1(6)	17.6(6)	15.9(6)	-2.6(5)	5.4(5)	-5.7(5)
C31	28.1(8)	21.3(7)	36.2(9)	-11.1(6)	11.6(7)	-1.2(6)
C32	35.9(9)	25.5(8)	42.1(10)	16.9(7)	-5.5(8)	-9.8(7)
C33	20.1(7)	21.3(7)	24.6(7)	-2.5(5)	0.9(5)	-5.2(5)

Atom	U_{11}	U_{22}	U_{33}	U_{23}	U_{13}	U_{12}
C34	31.8(8)	43.7(10)	28.1(8)	-16.4(7)	19.1(7)	-20.0(7)
B1	12.5(6)	15.8(7)	12.4(6)	-1.4(5)	3.5(5)	1.2(5)
B2	14.9(6)	16.6(7)	10.7(6)	-1.8(5)	5.5(5)	-1.0(5)

Table S4 Bond Lengths for 29e.

Atom	Atom	Length/Å	Atom	Atom	Length/Å
S1	O1	1.4300(10)	C8	C9	1.3320(18)
S1	O2	1.4326(10)	C8	C12	1.4779(18)
S1	N1	1.6650(11)	C9	C10	1.5026(18)
S1	C1	1.7614(13)	C10	C11	1.5568(18)
Si1	C19	1.9313(13)	C10	C18	1.5448(18)
Si1	C20	1.8693(16)	C10	C19	1.5802(17)
Si1	C21	1.8740(15)	C12	C13	1.3925(19)
Si1	C22	1.8695(15)	C12	C17	1.3926(19)
O3	C23	1.4610(16)	C13	C14	1.3861(19)
O3	B1	1.3736(18)	C14	C15	1.387(2)
O4	C27	1.4595(17)	C15	C16	1.385(2)
O4	B1	1.3665(17)	C16	C17	1.388(2)
O5	C29	1.4662(16)	C19	B1	1.572(2)
O5	B2	1.3725(18)	C19	B2	1.5741(19)
O6	C30	1.4571(16)	C23	C24	1.517(2)
O6	B2	1.3792(17)	C23	C26	1.522(2)
N1	C8	1.4452(16)	C23	C27	1.5686(19)
N1	C11	1.4984(16)	C25	C27	1.525(2)
C1	C2	1.3885(18)	C27	C28	1.519(2)
C1	C6	1.3911(19)	C29	C30	1.546(2)
C2	C3	1.3877(19)	C29	C31	1.524(2)
C3	C4	1.391(2)	C29	C32	1.517(2)
C4	C5	1.398(2)	C30	C33	1.5126(18)
C4	C7	1.505(2)	C30	C34	1.514(2)
C5	C6	1.385(2)			

Table S5 Bond Angles for 29e.

Atom	Atom	Atom	Angle/°	Atom	Atom	Atom	Angle/°
O1	S1	O2	120.16(6)	C17	C12	C8	119.40(12)
O1	S1	N1	106.90(6)	C14	C13	C12	120.10(13)
O1	S1	C1	108.23(6)	C13	C14	C15	120.64(14)
O2	S1	N1	105.66(6)	C16	C15	C14	119.33(13)
O2	S1	C1	108.84(6)	C15	C16	C17	120.43(14)
N1	S1	C1	106.22(6)	C16	C17	C12	120.27(14)
C20	Si1	C19	111.86(6)	C10	C19	Si1	116.14(8)
C20	Si1	C21	107.92(7)	B1	C19	Si1	103.30(8)
C20	Si1	C22	108.03(7)	B1	C19	C10	110.85(10)
C21	Si1	C19	114.73(6)	B1	C19	B2	108.30(11)
C22	Si1	C19	108.48(6)	B2	C19	Si1	107.79(9)
C22	Si1	C21	105.44(7)	B2	C19	C10	110.04(10)
B1	O3	C23	107.86(10)	O3	C23	C24	108.21(11)
B1	O4	C27	107.97(11)	O3	C23	C26	106.87(11)
B2	O5	C29	106.77(10)	O3	C23	C27	102.44(10)
B2	O6	C30	106.53(10)	C24	C23	C26	109.74(12)
C8	N1	S1	119.54(9)	C24	C23	C27	115.02(12)
C8	N1	C11	106.62(10)	C26	C23	C27	113.86(12)
C11	N1	S1	115.40(8)	O4	C27	C23	102.39(10)
C2	C1	S1	120.51(10)	O4	C27	C25	106.50(12)
C2	C1	C6	120.87(12)	O4	C27	C28	108.07(12)
C6	C1	S1	118.54(10)	C25	C27	C23	113.44(13)
C3	C2	C1	119.00(13)	C28	C27	C23	115.48(12)
C2	C3	C4	121.36(13)	C28	C27	C25	110.16(13)
C3	C4	C5	118.53(13)	O5	C29	C30	101.37(10)
C3	C4	C7	120.98(13)	O5	C29	C31	106.77(12)
C5	C4	C7	120.49(14)	O5	C29	C32	109.42(11)
C6	C5	C4	120.93(13)	C31	C29	C30	113.11(12)
C5	C6	C1	119.31(13)	C32	C29	C30	114.83(14)
N1	C8	C12	122.37(11)	C32	C29	C31	110.59(14)
C9	C8	N1	111.15(11)	O6	C30	C29	101.73(10)
C9	C8	C12	125.91(12)	O6	C30	C33	109.24(11)
C8	C9	C10	113.50(11)	O6	C30	C34	106.09(11)
C9	C10	C11	101.19(10)	C33	C30	C29	115.34(12)
C9	C10	C18	108.95(11)	C33	C30	C34	109.98(12)
C9	C10	C19	113.32(10)	C34	C30	C29	113.65(13)
C11	C10	C19	111.79(10)	O3	B1	C19	123.40(12)
C18	C10	C11	110.82(10)	O4	B1	O3	112.76(12)
C18	C10	C19	110.43(10)	O4	B1	C19	123.84(12)
N1	C11	C10	106.81(10)	O5	B2	O6	112.10(12)
C13	C12	C8	121.13(12)	O5	B2	C19	125.49(12)
C13	C12	C17	119.21(12)	O6	B2	C19	122.35(12)

Table S6 Torsion Angles for 29e.

A	B	C	D	Angle/°	A	B	C	D	Angle/°
S1	N1	C8	C9	129.07(10)	C11	N1	C8	C9	-4.06(13)
S1	N1	C8	C12	-59.13(14)	C11	N1	C8	C12	167.74(11)
S1	N1	C11	C10	-127.52(9)	C11	C10	C19	Si1	73.21(12)
S1	C1	C2	C3	176.06(11)	C11	C10	C19	B1	-169.33(10)
S1	C1	C6	C5	-176.54(11)	C11	C10	C19	B2	-49.56(13)
Si1	C19	B1	O3	76.72(13)	C12	C8	C9	C10	-173.11(11)
Si1	C19	B1	O4	-103.18(13)	C12	C13	C14	C15	-0.4(2)
Si1	C19	B2	O5	37.13(16)	C13	C12	C17	C16	-1.5(2)
Si1	C19	B2	O6	-139.90(11)	C13	C14	C15	C16	-0.5(2)
O1	S1	N1	C8	57.77(11)	C14	C15	C16	C17	0.4(2)
O1	S1	N1	C11	-172.96(9)	C15	C16	C17	C12	0.6(2)
O1	S1	C1	C2	147.75(11)	C17	C12	C13	C14	1.42(19)
O1	S1	C1	C6	-35.56(12)	C18	C10	C11	N1	107.16(11)
O2	S1	N1	C8	-173.14(9)	C18	C10	C19	Si1	-162.92(9)
O2	S1	N1	C11	-43.88(10)	C18	C10	C19	B1	-45.45(14)
O2	S1	C1	C2	15.59(13)	C18	C10	C19	B2	74.32(13)
O2	S1	C1	C6	-167.72(11)	C19	C10	C11	N1	-129.18(10)
O3	C23	C27	O4	25.07(13)	C23	O3	B1	O4	7.67(15)
O3	C23	C27	C25	-89.27(14)	C23	O3	B1	C19	-172.24(12)
O3	C23	C27	C28	142.22(12)	C24	C23	C27	O4	142.20(12)
O5	C29	C30	O6	-33.10(12)	C24	C23	C27	C25	27.86(17)
O5	C29	C30	C33	-151.19(12)	C24	C23	C27	C28	-100.65(15)
O5	C29	C30	C34	80.48(13)	C26	C23	C27	O4	-89.92(14)
N1	S1	C1	C2	-97.76(11)	C26	C23	C27	C25	155.74(13)
N1	S1	C1	C6	78.93(12)	C26	C23	C27	C28	27.24(18)
N1	C8	C9	C10	-1.66(15)	C27	O4	B1	O3	9.95(15)
N1	C8	C12	C13	-33.97(17)	C27	O4	B1	C19	-170.14(12)
N1	C8	C12	C17	151.91(12)	C29	O5	B2	O6	-10.40(15)
C1	S1	N1	C8	-57.63(10)	C29	O5	B2	C19	172.31(13)
C1	S1	N1	C11	71.64(10)	C30	O6	B2	O5	-12.36(15)
C1	C2	C3	C4	0.4(2)	C30	O6	B2	C19	165.03(12)
C2	C1	C6	C5	0.1(2)	C31	C29	C30	O6	80.84(13)
C2	C3	C4	C5	0.1(2)	C31	C29	C30	C33	-37.24(17)
C2	C3	C4	C7	179.32(14)	C31	C29	C30	C34	-165.58(12)

A	B	C	D	Angle/°	A	B	C	D	Angle/°
C3	C4	C5	C6	-0.5(2)	C32	C29	C30	O6	-150.93(12)
C4	C5	C6	C1	0.4(2)	C32	C29	C30	C33	90.99(16)
C6	C1	C2	C3	-0.5(2)	C32	C29	C30	C34	-37.35(17)
C7	C4	C5	C6	-179.74(14)	B1	O3	C23	C24	-142.22(12)
C8	N1	C11	C10	7.82(12)	B1	O3	C23	C26	99.66(13)
C8	C9	C10	C11	6.29(14)	B1	O3	C23	C27	-20.32(13)
C8	C9	C10	C18	-110.51(13)	B1	O4	C27	C23	-21.64(14)
C8	C9	C10	C19	126.12(12)	B1	O4	C27	C25	97.68(13)
C8	C12	C13	C14	-172.72(12)	B1	O4	C27	C28	-143.98(12)
C8	C12	C17	C16	172.70(12)	B1	C19	B2	O5	-74.01(16)
C9	C8	C12	C13	136.58(14)	B1	C19	B2	O6	108.97(14)
C9	C8	C12	C17	-37.54(19)	B2	O5	C29	C30	26.95(14)
C9	C10	C11	N1	-8.27(12)	B2	O5	C29	C31	-91.66(13)
C9	C10	C19	Si1	-40.36(14)	B2	O5	C29	C32	148.62(14)
C9	C10	C19	B1	77.10(13)	B2	O6	C30	C29	28.18(13)
C9	C10	C19	B2	-163.13(11)	B2	O6	C30	C33	150.56(12)
C10	C19	B1	O3	-48.35(16)	B2	O6	C30	C34	-90.93(13)
C10	C19	B1	O4	131.75(13)	B2	C19	B1	O3	-169.15(12)
C10	C19	B2	O5	164.69(12)	B2	C19	B1	O4	10.95(17)
C10	C19	B2	O6	-12.33(17)					

Table S7 Hydrogen Atom Coordinates (Å×10^4) and Isotropic Displacement Parameters (Å^2×10^3) for 29e

Atom	*x*	*y*	*z*	U(eq)
H2	9748.87	6127.54	5303.72	20
H3	9949.23	5742.34	6763.41	23
H5	7531.65	4364.83	5784.5	24
H6	7303.22	4751.29	4323.65	21
H7A	8211.97	4492.65	7380.31	41
H7B	8932.96	5112.68	7787.87	41
H7C	9666	4524.34	7542.17	41
H9	4558.73	6094.58	4151.68	16
H11A	6950.09	7156.61	3500.49	16
H11B	7856.76	6831.6	4356.33	16
H13	5917.06	5576.74	1809.54	20
H14	4880.42	4780.4	892.13	24
H15	3675.98	4069.37	1452.89	28
H16	3531.95	4153.98	2948.91	28
H17	4570.65	4947.04	3875.41	23
H18A	6992.7	7057.65	5711.8	25
H18B	7074.08	6322.09	5544.24	25
H18C	5859.14	6615.06	5738.89	25
H20A	4390.9	7642.16	1582.5	34
H20B	5458.8	7203.76	2143.24	34
H20C	5517.61	7943.63	2303.51	34
H21A	2400.73	6943.45	2180.97	30
H21B	2604.11	6749.89	3219.38	30
H21C	3448.58	6454.01	2623.59	30
H22A	2532.55	8221.51	2494.89	33
H22B	3685.92	8577.07	3111.69	33
H22C	2787.05	8188.91	3571.77	33
H24A	2202.78	6845.8	6658.99	39
H24B	3464.24	6539.5	6575.97	39
H24C	2207.51	6329.94	5900.89	39
H25A	3961.19	7556.81	7388.97	44
H25B	4910.93	8031.9	7122.42	44
H25C	4929.84	7308.2	6860.49	44
H26A	985.2	7452.32	5280.72	38
H26B	1280.88	6954.76	4580.82	38
H26C	1668.21	7670.97	4529.66	38
H28A	2161.34	8228.45	6524.02	43
H28B	2185.73	8458.65	5531.53	43
H28C	3204.07	8698.96	6387.94	43

Atom	*x*	*y*	*z*	U(eq)
H31A	7221.54	9513.3	5732.72	42
H31B	6566.24	8865.16	5845.31	42
H31C	5776.9	9441.02	5345.67	42
H32A	7540.48	9795	4158.3	56
H32B	6079.02	9817.52	3900.12	56
H32C	6780.2	9397.67	3319.89	56
H33A	9285.6	9094.39	5326.46	34
H33B	9565.67	8362.56	5461.14	34
H33C	8686.18	8697.45	5995.6	34
H34A	8670.03	8823.48	3652.93	49
H34B	7554	8352.75	3297.64	49
H34C	8838.12	8092.89	3874.09	49

S19. References

[S1] Mairena, M. A., Urbano, J., Carbajo, J., Maraver, J. J., Álvarez, E., Díaz-Requejo, M. M., and Pérez, P. J. *Inorg. Chem.* **2007**, *46*, 7428-7435. 10.1021/ic7007073.

[S2] Yamada, Y., Yamamoto, T., and Okawara, M. *Chem. Lett.* **1975**, 361-362. 10.1246/cl.1975.361.

[S3] a) Picher, M. I., and Plietker, B. *Org. Lett.* **2020***, 22*, 340-344. 10.1021/acs.orglett.9b04521. b) Barday, M., Ho, K. Y. T., Halsall, C. T., and Aïssa, C. *Org. Lett.* **2016***, 18*, 1756-1759. 10.1021/acs.orglett.6b00451.
c) Adamson, N. J., Jeddi, H., and Malcolmson, S. J. *J. Am. Chem. Soc.* **2019***, 141*, 8574-8583. 10.1021/jacs.9b02637. d) Wang, B., Li, Y., Pang, J. H., Watanabe, K., Takita, R., and Chiba, S. *Angew. Chem. Int.* **2021**, Ed. *60*, 217-221. 10.1002/anie.202012027. e) Zhu, Y., Li, T., Qu, X., Sun, P., Yang, H., and Mao, J. *Org. Biomol. Chem.* **2011***, 9*, 7309-7312. 10.1039/C1OB06210G.
